# Ligand-controlled palladium-catalyzed regiodivergent aminocarbonylation of *tert*-alcohols†

**DOI:** 10.1039/d4sc06011c

**Published:** 2024-11-15

**Authors:** Xing-Wei Gu, Yan-Hua Zhao, Xiao-Feng Wu

**Affiliations:** a Leibniz-Institut für Katalyse e.V. Albert-Einstein-Straße 29a 18059 Rostock Germany xiao-feng.wu@catalysis.de; b Dalian National Laboratory for Clean Energy, Dalian Institute of Chemical Physics, Chinese Academy of Sciences Dalian Liaoning 116023 China

## Abstract

Alcohols are widely available, abundant, and diverse in both commercial and natural resources. They possess low toxicity, making their use as reactants for carbonylation extremely promising. Herein, we present a robust ligand-controlled regioselective aminocarbonylation of *tert*-alcohols. Utilizing a commercially available palladium salt and ligand as the catalytic system, various amides containing an α-quaternary carbon or β-substituted amides can be selectively accessible. Notably, water is the only by-product of this reaction, which is consistent with the concept of green chemistry. This protocol offers a broad substrate scope, high regioselectivity, and excellent performance in scale-up reactions.

## Abstract 2

A robust ligand-controlled regioselective aminocarbonylation of *tert*-alcohols has been develope.

## Graphical abstract

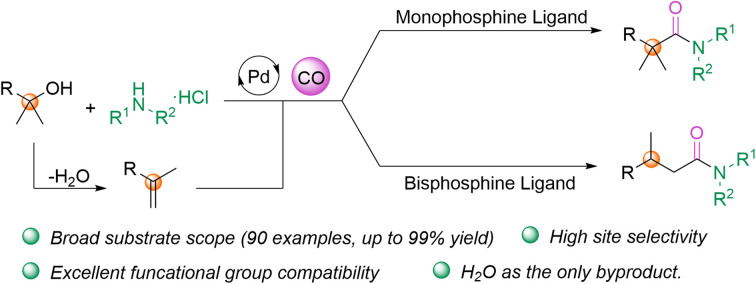

## Introduction

Amide skeletons are common building units in biologically active molecules.^1^ In 2020, a chemoinformatics analysis of the ChEMBL database of approximately 420 000 bioactive molecules was performed, and the investigation revealed that a staggering 40.3% of the molecules contain amide bonds.^2^ To date, amidation remains the most commonly utilized reaction in pharmaceutical synthesis, accounting for 16% of all reactions.^3^ Among the procedures for their synthesis, condensation of carboxylic acids with amines is the most straightforward method for preparing amides.^4^ However, the high activation barrier of the direct coupling of carboxylic acids and amines demands harsh reaction conditions to overcome.^5^ Laboratories typically pre-activate or *in situ* activate carboxylic acids (usually converting them to acyl chlorides) or use stoichiometric coupling reagents to achieve more efficient amide bond construction.^6^ Regrettably, these approaches generate a large amount of waste, and the pre-incorporation of the carboxylic acid carbonyls limits the scope of products to a certain extent.

**Scheme 1:**
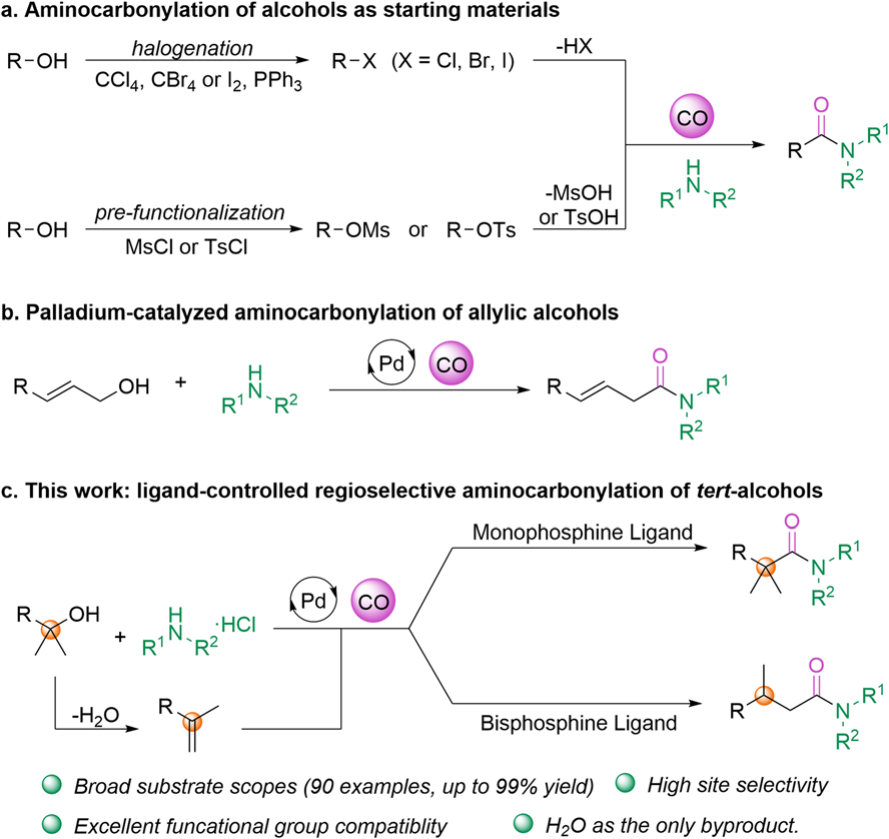
Aminocarbonylation of alcohols.

Carbon monoxide (CO) as a cheap and abundant source of C1 has attracted significant attention.^7^ Aminocarbonylation of alkyl halides is one of the main protocols for the synthesis of aliphatic amides (Scheme 1a). Based on the efforts of organic chemists, a variety of strategies have been established for the aminocarbonylation of alkyl halides, including transition metal catalysis,^8^ high-energy photocatalysis,^9^ and the recently popular visible-light-assisted photoredox catalysis.^10^ In addition, -OMs and -OTs, as excellent leaving groups, have been successively reported for carbonylation coupling reactions with amines.^11^ It is obvious that both the compounds are synthesized from alcohols and produced by halogenation (Appel reaction) as well as pre-functionalization.^12^ However, the preparation process typically involves and generates toxic and carcinogenic polyhalogenated compounds, along with wastes such as phosphine oxides, leading to serious environmental pollution.^13^ Not only that, the removal of the leaving functional group (-HX, TsOH, MsOH) during aminocarbonylation results in low step efficiency of the reaction. Indeed, as early as in 2007, a roundtable on green chemistry research areas identified “avoiding the formation of amides using poorly atomically economical reagents” as a high priority for organic chemistry.^14^ Therefore, exploring a rational and convenient manner of acquiring amides using readily available materials is still of great significance.

In this case, we pondered why not directly use alcohols as reaction partners for aminocarbonylation? Alcohols are widely available, abundant, and diverse in both commercial and natural resources. They are also inexpensive and possess low toxicity.^15^ However, aminocarbonylation of alcohols is challenging due to the poor ability to cleave the hydroxyl group.^16^ From a literature search, only one relevant instance has been reported: in 2016, a palladium-catalyzed aminocarbonylation of allyl alcohols was developed by Beller's group (Scheme 1b).^17^ It is worth mentioning that recently, Zhu's group reported the deoxyamidation reaction of alcohols with carbamoyl chloride having assembled carbonyl groups.^29^

On the other hand, the formation of olefins through alcohol dehydration is involved in hydroaminocarbonylation, producing only water as waste and aligning well with the principles of green chemistry. In particular, cases where some aliphatic alkenes can be produced from readily available alcohols are more attractive from a synthetic point of view. Additionally, utilizing olefins generated by alcohol dehydration in the reaction allows for regioselective control, enabling the production of different products from the same substrate, which is equally fascinating.^18^ Recently, the groups of Beller,^19^ Cole-Hamilton,^20^ Guan,^21^ Alper,^22^ Huang^23^ and several others^24^ have made significant contributions to palladium-catalyzed hydroaminocarbonylation. Inspired by this knowledge, we became interested in exploring the regioselective aminocarbonylation of alcohols (Scheme 1c).

## Results and discussion

Our studies started with the palladium-catalyzed aminocarbonylation of 2-phenylpropan-2-ol 1a and aniline hydrochloride 2a at 110 °C under 40 bar CO atmosphere in DCE. First, we tested several monophosphine ligands in the presence of PdCl_2_ and yielded the desired product 3a. The experiments show that the yield of 3a increased with decreasing ligand electron cloud density, reaching 54% when using (4-CF_3_C_6_H_4_)_3_P as the ligand (Table 1, entries 1–4). Subsequently, in the screening of palladium pre-catalysts, the best results were derived from [Pd(π-cinnamyl)Cl]_2_ as the catalyst (Table 1, entries 5–9). Moreover, changing the ligand to (3,5-CF_3_C_6_H_3_)_3_P afforded 3a in 67% yield (Table 1, entry 10). Finally, after constructing the optimal reaction conditions through appropriate adjustment of the reaction concentration and extension of the reaction time to 30 h, the yield of 3a was improved to 94% (Table 1, entries 11 and 12). A decreased amount of palladium pre-catalyst or lower CO pressure resulted in a decreased yield of the targeted amide product. It is worth mentioning that a trace amount of linear product 4a was detected during the optimization of the conditions, and thus we speculate that the reaction perhaps involves dehydration of alcohols to form olefins, followed by hydroaminocarbonylation regioselectively to afford branched and linear products.

**Table 1:** Optimization of the aminocarbonylation toward amides with a quaternary carbon^a^

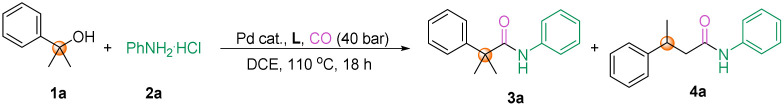				
Entry	[Pd]	Ligand	Yield^b^ (%)	3a : 4a
1	PdCl_2_	(4-MeOC_6_H_4_)_3_P	35	5 : 1
2	PdCl_2_	PPh_3_	47	6 : 1
3	PdCl_2_	(4-FC_6_H_4_)_3_P	49	6 : 1
4	PdCl_2_	(4-CF_3_C_6_H_4_)_3_P	54	6 : 1
5	Pd(OAc)_2_	(4-CF_3_C_6_H_4_)_3_P	55	4 : 1
6	Pd(acac)_2_	(4-CF_3_C_6_H_4_)_3_P	58	5 : 1
7	Pd(dba)_2_	(4-CF_3_C_6_H_4_)_3_P	57	5 : 1
8	Pd(MeCN)_2_Cl_2_	(4-CF_3_C_6_H_4_)_3_P	55	5 : 1
9	[Pd(π-cinnamyl)Cl]_2_	(4-CF_3_C_6_H_4_)_3_P	62	6 : 1
10	[Pd(π-cinnamyl)Cl]_2_	(3,5-CF_3_C_6_H_3_)_3_P	67	>20 : 1
11^c^	[Pd(π-cinnamyl)Cl]_2_	(3,5-CF_3_C_6_H_3_)_3_P	70	>20 : 1
12^d^	[Pd(π-cinnamyl)Cl]_2_	(3,5-CF_3_C_6_H_3_)_3_P	94(91)	>20 : 1

a Conditions: 1a (0.3 mmol), 2a (0.2 mmol), [Pd] (5 mol%), ligand (12 mol%), CO (40 bar), DCE (2.0 mL), stirred at 110 °C for 18 h.

b Yields were determined by GC with hexadecane as the internal standard.

c DCE (1.0 mL).

d 30 h isolated yield is shown in parentheses.

Considering the significance of linear amides, we attempted reaction optimization toward this product. As we know, the addition of an LPd-H complex to an alkene usually follows the Markovnikov rule and gives a branched product. However, the palladium complex will become bulky when coordinated with a bidentate ligand, which then leads to the Markovnikov addition intermediate not being stable; in comparison, the less steric palladium complex from anti-Markovnikov addition is much favored, which can finally give the linear product. Therefore, we screened a variety of bisphosphine ligands, and interestingly the bite angle of the bisphosphine ligands had a significant effect on this transformation (Table 2). The reaction could not proceed when DPPP with a bite angle of 91° was used as the ligand. In the presence of BINAP (bite angle: 93°) or DPPF (bite angle: 99°), the desired product 4a could be detected, albeit in low yield. Encouragingly, the application of Xantphos-type ligands such as DPEphos (bite angle: 104°), Xantphos (bite angle: 111°), and NIXantphos (bite angle: 114°) in the reaction resulted in significantly improved yields, with NIXantphos achieving the best results with 82% yield. Finally, reducing the pressure of CO to 20 bar further increased the yield to 98%. Notably, decreased yields were observed if we performed the reaction under lower or higher temperatures (for more details on optimization, please see the ESI†).

**Table 2:** Optimization of the aminocarbonylation toward β-substituted amide^a^

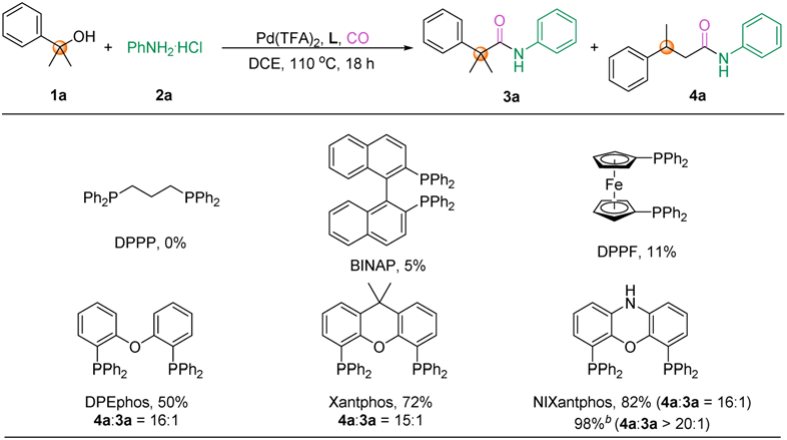

a Conditions: 1a (0.3 mmol), 2a (0.2 mmol), Pd(TFA)_2_ (1 mol%), ligand (1.2 mmol%), CO (40 bar), and DCE (2.0 ml), stirred at 110 °C for 18 h. Yields were determined by GC with hexadecane as the internal standard. CO (20 bar), isolated yield.

**Scheme 2:**
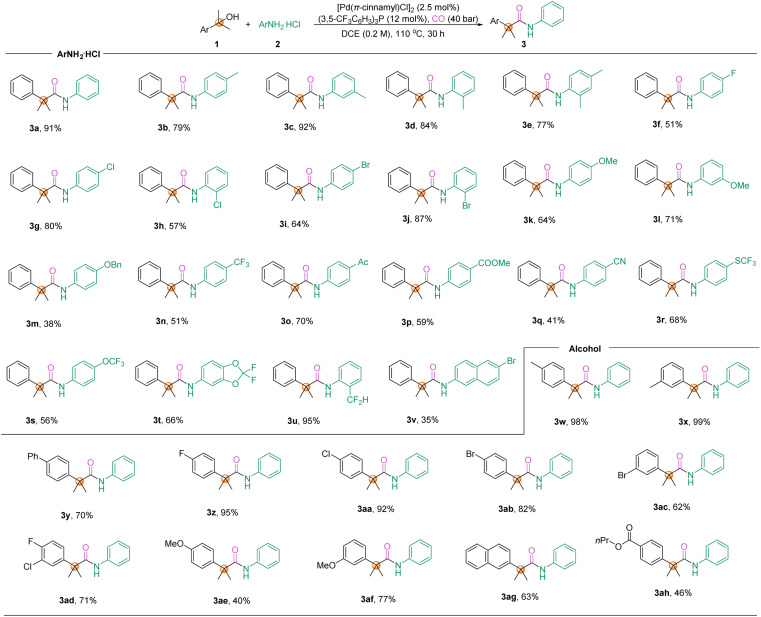
Scope of aminocarbonylation toward amides with a quaternary carbon. Reaction conditions: alcohols (0.3 mmol, 1.5 equiv.), ArNH_2_ HCl (0.2 mmol, 1.0 equiv.), [Pd(π-cinnamyl)Cl]_2_ (2.5 mmol%), (3,5-CF_3_C_6_H_3_)_3_P (12 mol%), CO (40 bar), 110 °C, 30 h.

With the optimized reaction conditions in hand, the product scope was subsequently explored. Initially, aminocarbonylation of tertiary alcohols with anilines was carried out to provide amides containing an α-quaternary carbon. As shown in Scheme 2, both *ortho*-, *meta*- and *para*-substituted anilines can be successfully applied to the reaction and provide the corresponding amides with an α quaternary carbon in moderate to excellent yields (3a–3v). Notably, the electronic nature of aniline hardly affects the conversion, and functional groups besides alkyl (3a–3e) and halogen groups (F, Cl, Br, 3f–3j), alkoxy (3k–3m), trifluoromethyl (3n), acyl (3o), ester (3p), and cyano (3q) groups are also perfectly tolerated. In addition, fluorine-containing functional groups including trifluoromethyl (3n), trifluoromethylsulfanyl (3r), trifluoromethoxy (3s), and fluorinated heterocycles (3t), as well as difluoromethyl groups (3u) were reserved under the reaction conditions and the corresponding amides were obtained in 51–95% yields. The naphthyl group (3v) was also tolerated in our reaction, although in low yield. Then, the compatibility of various alcohols in this reaction regime was explored. The vast majority of substituted alcohols can be converted to the corresponding products in good yields and with excellent regioselectivity (3w–3ah). Unfortunately, *ortho*-substituted alcohols were restricted and only trace amounts of linear amides were observed, which we attribute to the steric hindrance effect of the substrate inhibiting the Markovnikov addition of the L-Pd-H complex. The reaction failed when 3-aminophenol was tested under our standard conditions. Aliphatic tertiary alcohols were tested as well, but no desired product could be detected.

**Scheme 3:**
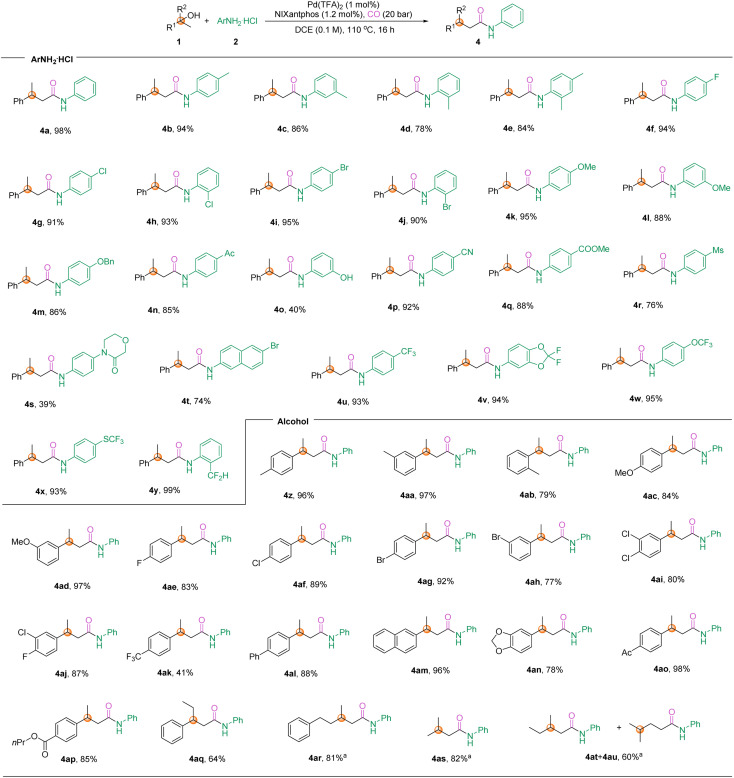
Scope of aminocarbonylation toward β-substituted amides. Reaction conditions: alcohols (0.3 mmol, 1.5 equiv.), ArNH_2_ HCl (0.2 mmol, 1.0 equiv.), Pd(TFA)_2_ (1 mmol%), NIXantphos (1.2 mol%), CO (20 bar), 110 °C, 18 h. [a] TsOH (5 mol%), alcohols (0.4 mmol, 2 equiv.).

Our next goal was to evaluate the preparation scope of linear amides. As shown in Scheme 3, various aryl amines were tested and provided the desired linear amides in satisfactory yields. Similarly, numerous functional groups can be accommodated, for instance anilines with electron-withdrawing groups including halogen (F, Cl, Br, 4f–4j), acyl (4n), cyano (4p), ester (4q), and methylsulfonyl (4r), as well as electron-donating groups such as alkyl (4a–4e) and alkoxy (4k–4m) can be successfully employed in the reaction and the desired linear amides were afforded in good to excellent yields. Pleasingly, phenol (4o) and amide (4s) were also tolerated under these conditions, with yields of 40% and 39%, respectively. Fluorine-containing molecules (4u–4y) also produced the desired amides in excellent yields. We then turned our attention to the substrate scope of alkyl alcohols. As expected, *para*-, *meta*-, and *ortho*-substituted alcohols can participate in the reaction and afford the corresponding amides (4z–4ap) without any problem. Not only that, 2-phenylbutan-2-ol (4aq) was also a suitable reaction partner to give the β-ethyl substituted amide in 64% yield. Furthermore, the participation of *tert*-butanol substrates in the reaction only requires the addition of 5 mol% of TsOH to promote the dehydration of alcohols to form olefins, which undergo hydroaminocarbonylation to access linear amides (4ar–4au). Especially in the cases of 4as and 4at, the alkenes are a gas, but can be produced from liquid alcohols and then transformed successfully in this system, which further proves the value of this procedure. The results show the excellent functional group compatibility proving the robustness of the protocol.

**Scheme 4:**
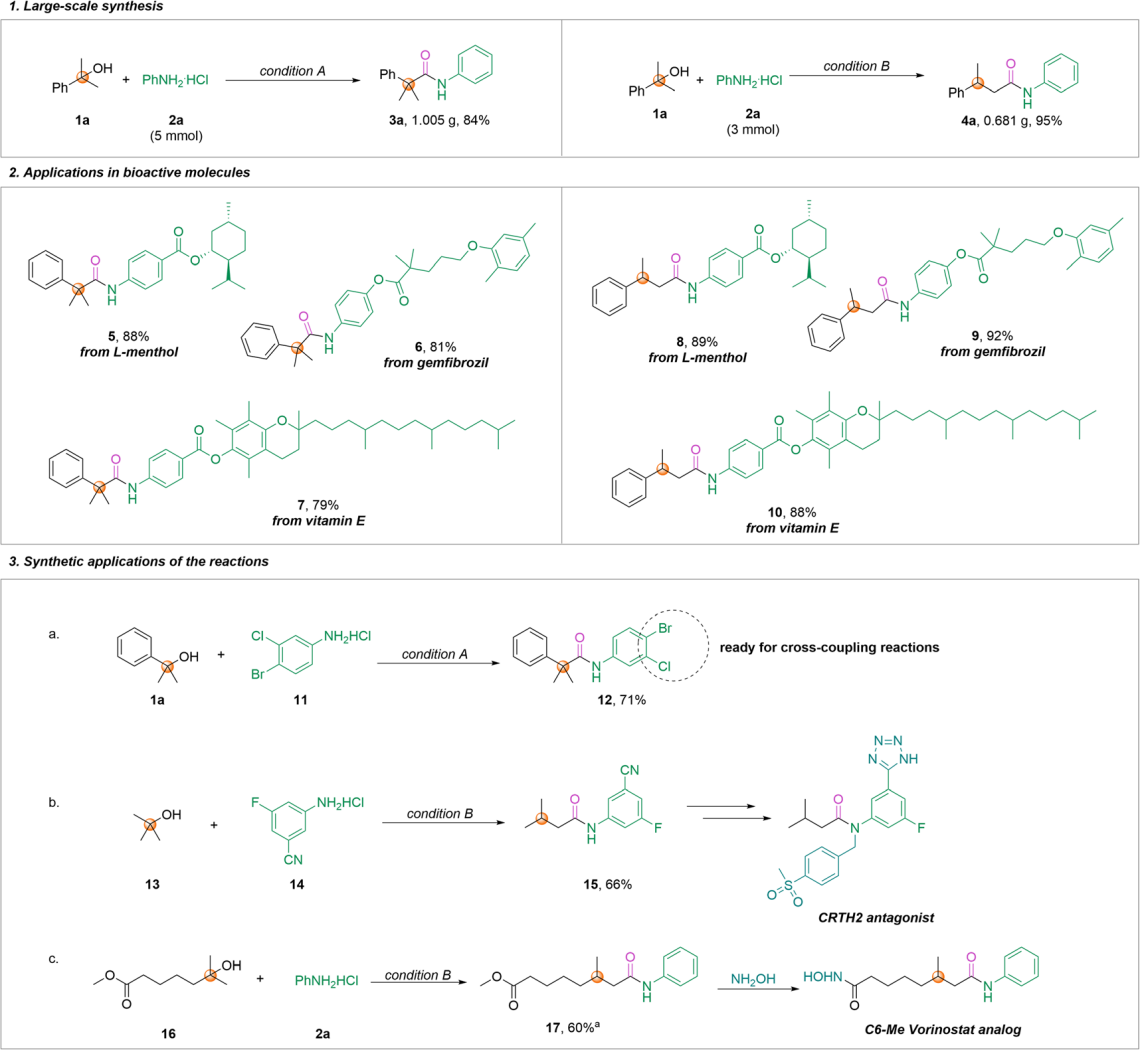
Synthetic applications of the aminocarbonylation.

To illustrate the potential value of this reaction in the field of synthesis, further large-scale synthesis as well as synthetic applications of bioactive molecules were accomplished. As shown in Scheme 4, when the reaction was expanded to 5 mmol under condition A, 1.005 g of product 3a was obtained in 84% yield. In addition, we also obtained amide 4a in 95% yield after scaling up the reaction 15-fold under condition B, providing a potential opportunity for application in large-scale production (Scheme 4, eqn(1)). Importantly, our aminocarbonylation allowed the doping of amides containing an α-quaternary carbon or β-substituted amide fragments into biologically active molecules, such as l-menthol, gemfibrozil, and vitamin E, and the desired products were successfully obtained in yields ranging from 79% to 92% (Scheme 4, eqn (2)). This ligand-controlled regioselective aminocarbonylation reaction demonstrates the utility of the approach through the formation of valuable chemical compounds (Scheme 4, eqn (3)). For instance, *tert*-alcohol 1a and aryl amine 11 can synthesize compound 12, which is ready for further modifications *via* cross-coupling reactions. Amide 15 is a critical synthetic intermediate for the synthesis of CRTH2 antagonist and was afforded in 66% yield under condition B.^25,26^ It is worth mentioning that vorinostat allows the introduction of methyl groups through this protocol, providing a great facility for modifying valuable pharmaceutical molecules.^27^ However, the reactions with 2-thiophene-substituted amine and alcohol failed. The attempts at using aniline trifluoromethanesulfonate and aliphatic amines as the substrates did not proceed either. In the case of secondary alcohols, such as cyclohexanol, only trace amounts of the desired products were detected under our conditions, even after increasing the reaction temperature to 140 °C.

**Scheme 5:**
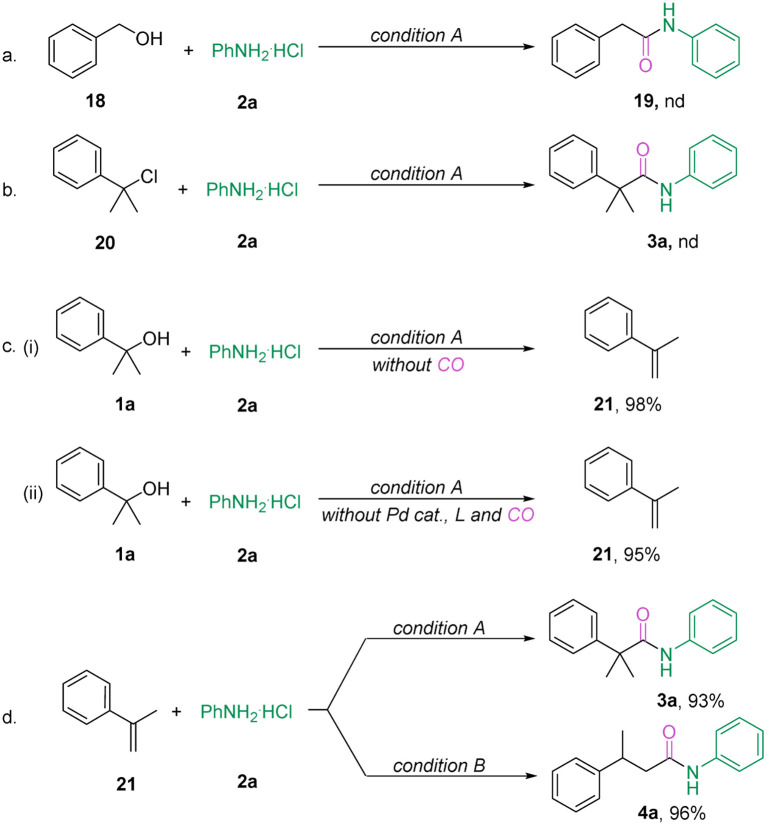
Control experiments and mechanistic studies.

To gain more insight into the mechanism of the reaction, several controlled experiments were performed. First, using benzyl alcohol 18 as a substrate did not yield the product 19, suggesting that the reaction did not proceed *via* hydronium ion dissociation (Scheme 5a).^17^ In addition, in order to exclude the possibility of carbon cation intermediate involvement (2-chloropropan-2-yl)benzene 20 as a reactive substrate was tested but did not react under condition A (Scheme 5b). Interestingly, a significant amount of olefin 21 was detected in the absence of CO and/or palladium catalysis participation (Scheme 5c). We therefore hypothesize that the reaction is achieved by dehydration of the alcohol to form an olefin, followed by hydroaminocarbonylation. As expected, the olefin 21 can yield the corresponding amides under condition A or condition B in excellent yields (Scheme 5d).

**Scheme 6:**
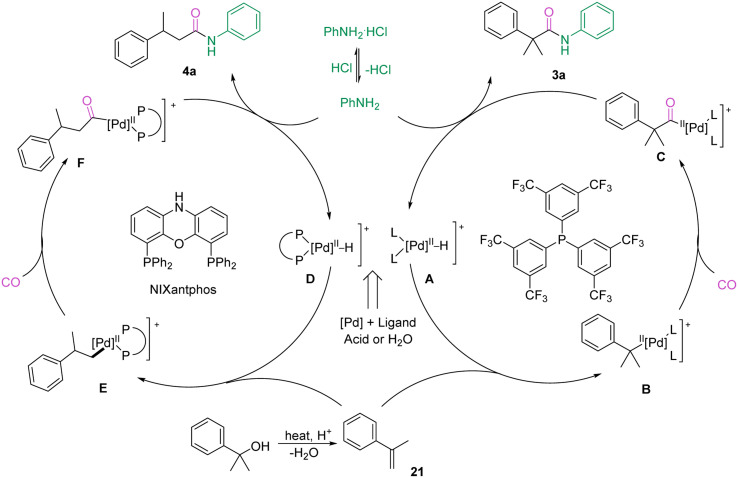
Mechanistic proposal.

A possible reaction mechanism to account for the aminocarbonylation of alcohols as well as the regioselectivity is proposed (Scheme 6).^19–24,28^ First, palladium salts and bidentate or monodentate ligands under acidic conditions generate Pd-H complexes A or D. Then, Markovnikov or anti-Markovnikov addition of the Pd-H complexes to the olefins formed by dehydration of the alcohols generates the corresponding branched or linear intermediates B or E, which are converted to acyl-palladium complexes C or F by coordination and insertion of CO. Finally, anilines attack the acyl-palladium complexes to afford the corresponding amides and regenerate the Pd-H complex for the next catalytic cycle.

## Conclusions

In conclusion, we have developed a novel ligand-controlled approach for regioselective aminocarbonylation of alcohols. A wide range of alcohols are readily available, and ligand modulation enables the direct production of a variety of desired amides containing an α-quaternary carbon or β-substituted amides with excellent regioselectivity. This protocol offers a broad substrate scope, high regioselectivity, and also excellent performance in scale-up reactions.

## Data availability

The data supporting this article have been included as part of the ESI.†

## Author contributions

XFW directed this project. XWG and YHZ performed all the experiments and prepared ESI.† XFW and XWG prepared and revised this manuscript.

## Conflicts of interest

There are no conflicts to declare.

## Supplementary Material

SC-015-D4SC06011C-s001
