# Supplementary material for: Ligand-controlled palladium-catalyzed regiodivergent aminocarbonylation of tert-alcohols
Source: Chem Sci. 2024 Nov 15;15(47):19970–6. doi: 10.1039/d4sc06011c (PMC11575597; doi:10.1039/d4sc06011c)
Supplement: SC-015-D4SC06011C-s001 [file SC-015-D4SC06011C-s001.pdf]

Supplementary Information (SI) for Chemical Science.
This journal is © The Royal Society of Chemistry 2024

# Supporting Information

## Ligand-Controlled Palladium-Catalyzed Regiodivergent Aminocarbonylation of *tert*-Alcohols

Xing-Wei Gu,[a] Yan-Hua Zhao,[a] and Xiao-Feng Wu*[a,b]

[a] X.-W. Gu, Y.-H. Zhao, Prof. Dr. X.-F. Wu, Leibniz-Institut für Katalyse e.V., Albert-Einstein-Straße 29a, 18059 Rostock, Germany, E-mail: xiao-feng.wu@catalysis.de, xwu2020@dicp.ac.cn

[b] Prof. Dr. X.-F. Wu, Dalian National Laboratory for Clean Energy, Dalian Institute of Chemical Physics, Chinese Academy of Sciences, 116023, Dalian, Liaoning, China

# Contents


**1. General Experimental.** ........ **S3**

**2. Optimization studies** ........ **S4**

**3. General Procedure for the aminocarbonylation.** ........ **S6**

**3.1 General Procedure I the aminocarbonylation toward amide with quaternary carbon.** ........ **S6**

**3.2 General Procedure II: the aminocarbonylation toward *β*-substituted amide.** ........ **S6**

**4. Characterization of the Products.** ........ **S7**

**5. Intermediate verification experiment** ........ **S38**

**6. Reference** ........ **S39**

**7. NMR spectra of products** ........ **S40**

1. General Experimental.

**Reagents and solvents:** Unless otherwise noted, the chemicals were commercially available from Sigma-Aldrich, TCI, BLD or Alfa Aesar and were used without further purification. The reaction does not require the glovebox.

**Purification:** The products were isolated from the reaction mixture by column chromatography on silica gel 60, 0.063-0.2 mm, 70-230 mesh (Merck). Gradient flash chromatography was conducted eluting with PE/EA, PE refers to pentane and EA refers to ethyl acetate, they were listed as volume/volume ratios.

**Data collection:** GC-yields were calculated using hexadecane as internal standard. GC analysis was performed on an Agilent HP-7890A instrument with FID detector and HP-5 capillary column (polydimethylsiloxane with 5% phenyl groups, 30 m, 0.32 mm i.d., 0.25 µm film thickness) using argon as carrier gas. High resolution mass spectra (HRMS) were recorded on Agilent 6210. NMR spectra were recorded on Bruker Avance 300 and Bruker ARX 400 spectrometers. Chemical shifts (ppm) are given relative to solvent: references for CDCl3 were 7.26 ppm ($^1$H NMR) and 77.00 ppm ($^{13}$C NMR). All measurements were carried out at room temperature unless otherwise stated.

2. Optimization studies

**Table 1.** Optimization of the aminocarbonylation toward amide with quaternary carbon.[a]

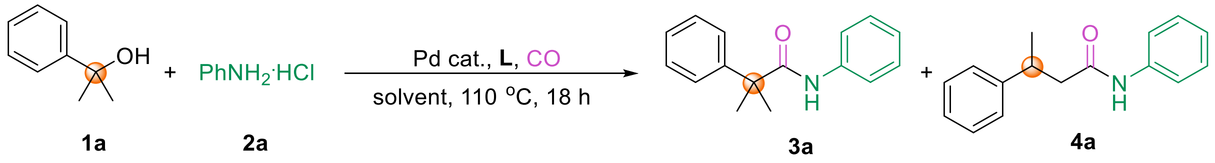


| entry | Pd cat. | ligand | solvent | concentration | Yield (%)[b] | **3a**:**4a** |
|---|---|---|---|---|---|---|
| 1 | $PdCl_2$ | $PPh_3$ | DCE | 0.1 | 47 | 6:1 |
| 2 | $PdCl_2$ | $(4\text{-}OMeC_6H_4)_3P$ | DCE | 0.1 | 35 | 5:1 |
| 3 | $PdCl_2$ | $(4\text{-}FC_6H_4)_3P$ | DCE | 0.1 | 49 | 6:1 |
| 4 | $PdCl_2$ | $(4\text{-}CF_3C_6H_4)_3P$ | DCE | 0.1 | 54 | 6:1 |
| 5 | $PdCl_2$ | *n*BuPAd$_2$ | DCE | 0.1 | 35 | 10:1 |
| 6 | $PdCl_2$ | X-Phos | DCE | 0.1 | 17 | > 20:1 |
| 7 | $Pd(OAc)_2$ | $(4\text{-}CF_3C_6H_4)_3P$ | DCE | 0.1 | 55 | 4:1 |
| 8 | $[Pd(allyl)Cl]_2$ | $(4\text{-}CF_3C_6H_4)_3P$ | DCE | 0.1 | 62 | 6:1 |
| 9 | $[Pd(cinnamyl)Cl]_2$ | $(4\text{-}CF_3C_6H_4)_3P$ | DCE | 0.1 | 62 | 6:1 |
| 10 | $Pd_2dba_3$ | $(4\text{-}CF_3C_6H_4)_3P$ | DCE | 0.1 | 55 | 5:1 |
| 11 | $Pd(TFA)_2$ | $(4\text{-}CF_3C_6H_4)_3P$ | DCE | 0.1 | 49 (17)[c] | 3:1 |
| 12 | $Pd(MeCN)_2Cl_2$ | $(4\text{-}CF_3C_6H_4)_3P$ | DCE | 0.1 | 43 | 5:1 |
| 13 | $Pd(acac)_2$ | $(4\text{-}CF_3C_6H_4)_3P$ | DCE | 0.1 | 58 | 5:1 |
| 14 | $Pd(dba)_2$ | $(4\text{-}CF_3C_6H_4)_3P$ | DCE | 0.1 | 57 | 5:1 |
| 15 | $[Pd(cinnamyl)Cl]_2$ | $(3,5\text{-}CF_3\ C_6H_3)_3P$ | DCE | 0.1 | 67 | > 20:1 |
| 16 | $[Pd(cinnamyl)Cl]_2$ | $(3,5\text{-}CF_3\ C_6H_3)_3P$ | MeCN | 0.1 | 17 | 1:1 |
| 17 | $[Pd(cinnamyl)Cl]_2$ | $(3,5\text{-}CF_3\ C_6H_3)_3P$ | 1,4-dioxane | 0.1 | 33 | 13:1 |
| 18 | $[Pd(cinnamyl)Cl]_2$ | $(3,5\text{-}CF_3\ C_6H_3)_3P$ | Toluene | 0.1 | nd | - |
| 19 | $[Pd(cinnamyl)Cl]_2$ | $(3,5\text{-}CF_3\ C_6H_3)_3P$ | anisole | 0.1 | 26 | > 20:1 |
| 20 | $[Pd(cinnamyl)Cl]_2$ | $(3,5\text{-}CF_3\ C_6H_3)_3P$ | DCE | 0.2 | 70 | > 20:1 |
| 21 | $[Pd(cinnamyl)Cl]_2$ | $(3,5\text{-}CF_3\ C_6H_3)_3P$ | DCE | 0.4 | 64 | > 20:1 |
| 22[d] | $[Pd(cinnamyl)Cl]_2$ | $(3,5\text{-}CF_3\ C_6H_3)_3P$ | DCE | 0.2 | 94 | > 20:1 |

[a] Conditions: **1a** (0.3 mmol), **2a** (0.2 mmol), [Pd] (5 mol%), ligand (12 mol%), CO (40 bar), solvent (X mL), stirred at 110°C for 18 h. [b] Yields were determined by GC with hexadecane as the internal standard. [c] Yield of **4a** is shown in parentheses. [d] 30 h.

**Table 2.** Optimization of the aminocarbonylation toward *β*-substituted amide.[a]

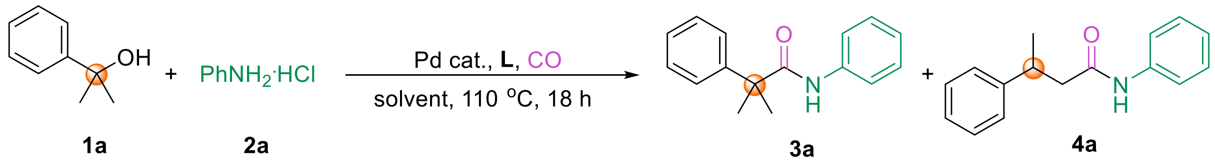


| entry | Pd cat. | [Pd]/L | ligand | CO | Yield (%)[b] | **4a**:**3a** |
|---|---|---|---|---|---|---|
| 1 | $Pd(TFA)_2$ | 5%/6% | NIXantphos | 40 bar | 85 | 17:1 |
| 2 | $Pd(OAc)_2$ | 5%/6% | NIXantphos | 40 bar | 82 | 15:1 |
| 3 | $[Pd(cinnamyl)Cl]_2$ | 5%/6% | NIXantphos | 40 bar | 78 | 15:1 |
| 4 | $PdCl_2$ | 5%/6% | NIXantphos | 40 bar | 75 | 16:1 |
| 5 | $Pd(dba)_2$ | 5%/6% | NIXantphos | 40 bar | 83 | 13:1 |
| 6 | $Pd(acac)_2$ | 5%/6% | NIXantphos | 40 bar | 84 | 15:1 |
| 7 | $Pd(TFA)_2$ | 3%/3.6% | NIXantphos | 40 bar | 86 | 17:1 |
| 8 | $Pd(TFA)_2$ | 1%/1.2% | NIXantphos | 40 bar | 82 | 19:1 |
| 9 | $Pd(TFA)_2$ | 1%/1.2% | DPPP | 40 bar | nd | - |
| 10 | $Pd(TFA)_2$ | 1%/1.2% | DPEPhos | 40 bar | 50 | 16:1 |
| 11 | $Pd(TFA)_2$ | 1%/1.2% | Xantphos | 40 bar | 72 | 15:1 |
| 12 | $Pd(TFA)_2$ | 1%/1.2% | Dppf | 40 bar | 11 | - |
| 13 | $Pd(TFA)_2$ | 1%/1.2% | Binap | 40 bar | 5 | - |
| 14 | $Pd(TFA)_2$ | 1%/1.2% | NIXantphos | 20 bar | 98 | > 20:1 |
| 15 | $Pd(TFA)_2$ | 1%/1.2% | NIXantphos | 1 bar | 34 | > 20:1 |

[a] Conditions: **1a** (0.3 mmol), **2a** (0.2 mmol), $Pd(TFA)_2$ (X mol%), ligand (Y mol%), CO (Z bar), DCE (2.0 mL), stirred at 110°C for 18 h. Yields were determined by GC with hexadecane as the internal standard. [b] Yields were determined by GC with hexadecane as the internal standard.

3. General Procedure for the aminocarbonylation.

3.1 General Procedure I the aminocarbonylation toward amide with quaternary carbon.

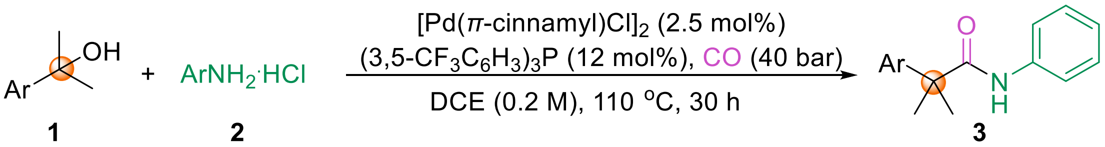

A 4 mL screw-cap vial was charged with $[Pd(cinnamyl)Cl]_2$ (2.6 mg, 2.5 mol%), $(4\text{-}CF_3C_6H_4)_3P$ (16.1 mg, 12 mol%), $ArNH_2{\cdot}HCl$ (0.2 mmol, 1.0 equiv.), alcohols (0.3 mmol, 1.5 equiv.) and an oven-dried stirring bar. The vial was closed with a Teflon septum and cap and connected to the atmosphere via a needle. After DCE (1.0 mL) was added with a syringe under argon atmosphere, the vial was moved to an alloy plate and put into a Parr 4560 series autoclave (300 mL) under argon atmosphere. At room temperature, the autoclave was flushed three times with $N_2$ and CO, respectively, and finally charged with 40 atm CO. The autoclave was placed on a heating plate equipped with a magnetic stirrer and an aluminum block. The reaction mixture was heated to 110 °C for 30 h. After reaction, cooling to room temperature. The crude product was purified by silica gel chromatography (pentane/EA) to afford the corresponding product.

3.2 General Procedure II: the aminocarbonylation toward *β*-substituted amide.

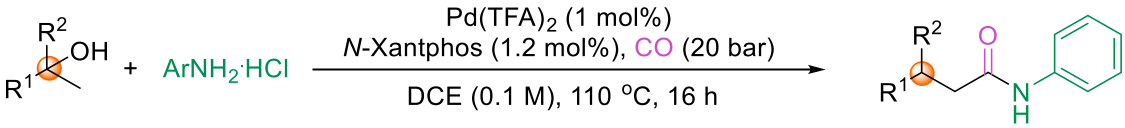

A 4 mL screw-cap vial was charged with $Pd(TFA)_2$ (0.7 mg, 1 mol%), NiXantphos (1.4 mg, 1.2 mol%), $ArNH_2{\cdot}HCl$ (0.2 mmol, 1.0 equiv.), alcohols (0.3 mmol, 1.5 equiv.) and an oven-dried stirring bar. The vial was closed with a Teflon septum and cap and connected to the atmosphere via a needle. After DCE (2.0 mL) was added with a syringe, the vial was moved to an alloy plate and put into a Parr 4560 series autoclave (300 mL). At room temperature, the autoclave was flushed three times with $N_2$ and CO, respectively, and finally charged with 20 atm CO. The autoclave was placed on a heating plate equipped with a magnetic stirrer and an aluminum block. The reaction mixture was heated to 110 °C for 18 h. After reaction, cooling to room temperature. The crude product was purified by silica gel chromatography (pentane/EA) to afford the corresponding product.

4. Characterization of the Products.

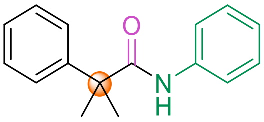

**3a**

**2-methyl-*N*,2-diphenylpropanamide (3a) [1]**

Chromatography Pentane/EA = 15:1 (v/v), 43.4 mg (91%).

$^{1}$H NMR (300 MHz, $CDCl_3$) δ 7.42 – 7.08 (m, 9H), 7.05 – 6.91 (m, 1H), 6.73 (s, 1H), 1.58 (s, 6H).

$^{13}$C NMR (75 MHz, $CDCl_3$) δ 175.5, 144.5, 137.9, 129.0, 128.8, 127.3, 126.4, 124.1, 119.6, 48.0, 27.0.

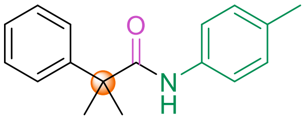

**3b**

**2-methyl-2-phenyl-*N*-(p-tolyl)propanamide (3b) [1]**

Chromatography Pentane/EA = 15:1 (v/v), 40.2 mg (79%).

$^{1}$H NMR (300 MHz, $CDCl_3$) δ 7.40 – 7.27 (m, 4H), 7.25 – 7.19 (m, 1H), 7.18 – 7.11 (m, 2H), 7.00 – 6.92 (m, 2H), 6.67 (s, 1H), 2.19 (s, 3H), 1.57 (s, 6H).

$^{13}$C NMR (75 MHz, $CDCl_3$) δ 175.4, 144.7, 135.4, 133.7, 129.3, 128.9, 127.3, 126.4, 119.7, 47.9, 27.0, 20.7.

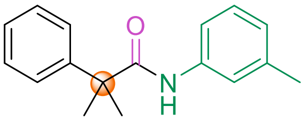

**3c**

**2-methyl-2-phenyl-*N*-(*m*-tolyl)propanamide (3c) [1]**

Chromatography Pentane/EA = 15:1 (v/v), 46.5 mg (92%).

$^{1}$H NMR (300 MHz, $CDCl_3$) δ 7.40 – 7.27 (m, 4H), 7.25 – 7.19 (m, 1H), 7.16 – 7.13 (m, 1H), 7.12 – 6.98 (m, 2H), 6.84 – 6.73 (m, 2H), 6.69 (s, 1H), 2.20 (s, 3H), 1.57 (s, 6H).

$^{13}$C NMR (75 MHz, $CDCl_3$) δ 175.5, 144.5, 138.7, 137.8, 128.9, 128.6, 127.3, 126.4, 124.8, 120.2, 116.6, 48.0, 27.0, 21.3.

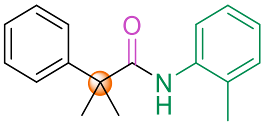

**3d**

**2-methyl-2-phenyl-*N*-(*o*-tolyl)propanamide (3d) [1]**

Chromatography Pentane/EA = 15:1 (v/v), 42.7 mg (84%).

$^1$H NMR (300 MHz, $CDCl_3$) δ 7.91 – 7.81 (m, 1H), 7.57 – 7.48 (m, 2H), 7.47 – 7.40 (m, 2H), 7.38 – 7.30 (m, 1H), 7.24 – 7.14 (m, 1H), 7.12 – 6.94 (m, 2H), 6.72 (s, 1H), 1.81 (s, 3H), 1.72 (s, 6H).

$^{13}$C NMR (75 MHz, $CDCl_3$) δ 175.5, 144.6, 135.9, 130.2, 129.0, 127.9, 127.4, 126.7, 126.6, 124.5, 121.8, 48.0, 26.8, 16.9.

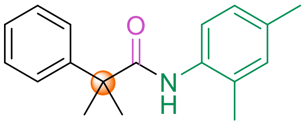

**3e**

***N*-(2,4-dimethylphenyl)-2-methyl-2-phenylpropanamide (3e)**

Chromatography Pentane/EA = 15:1 (v/v), 40.9 mg (77%).

$^1$H NMR (300 MHz, $CDCl_3$) δ 7.59 (d, *J* = 8.2 Hz, 1H), 7.43 – 7.38 (m, 2H), 7.37 – 7.29 (m, 2H), 7.28 – 7.19 (m, 1H), 6.94 – 6.84 (m, 1H), 6.83 – 6.76 (m, 1H), 6.54 (s, 1H), 2.16 (s, 3H), 1.70 (s, 3H), 1.61 (s, 6H).

$^{13}$C NMR (75 MHz, $CDCl_3$) δ 175.5, 144.8, 134.2, 133.2, 130.9, 128.9, 128.3, 127.3, 127.1, 126.6, 122.1, 47.9, 26.9, 20.7, 16.9.

HRMS (ES-TOF): m/z calcd. for $C_{18}H_{21}NONa^+$ [M+Na$^+$] 290.1515, found 290.1518.

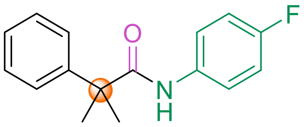

**3f**

***N*-(4-fluorophenyl)-2-methyl-2-phenylpropanamide (3f)**

Chromatography Pentane/EA = 15:1 (v/v), 26.1 mg (51%).

$^1$H NMR (300 MHz, $CDCl_3$) δ 7.41 – 7.29 (m, 4H), 7.28 – 7.16 (m, 3H), 6.94 – 6.79 (m, 2H), 6.71 (s, 1H), 1.58 (s, 6H).

$^{13}$C NMR (75 MHz, $CDCl_3$) δ 175.6, 159.2 (d, *J* = 243.3 Hz), 144.4, 133.9 (d, *J* = 2.7 Hz), 129.0, 127.4, 126.4, 121.5 (d, *J* = 7.9 Hz), 115.4 (d, *J* = 22.4 Hz), 47.9, 27.0.

$^{19}$F NMR (282 MHz, $CDCl_3$) δ -118.2 – -118.3 (m).

HRMS (ES-TOF): m/z calcd. for $C_{16}H_{16}FNONa^+$ [M+Na$^+$] 280.1108, found 280.1111.

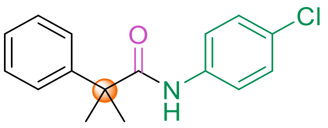

**3g**

**N-(4-chlorophenyl)-2-methyl-2-phenylpropanamide (3g)** [1]

Chromatography Pentane/EA = 15:1 (v/v), 43.7 mg (80%).

$^{1}$H NMR (300 MHz, $CDCl_3$) δ 7.40 – 7.18 (m, 7H), 7.14 – 7.03 (m, 2H), 6.75 (s, 1H), 1.57 (s, 6H).

$^{13}$C NMR (75 MHz, $CDCl_3$) δ 175.6, 144.3, 136.5, 129.0, 128.8, 127.4, 126.4, 120.9, 48.0, 26.9.

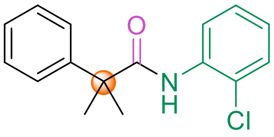

**3h**

**N-(2-chlorophenyl)-2-methyl-2-phenylpropanamide (3h)** [1]

Chromatography Pentane/EA = 15:1 (v/v), 31.0 mg (57%).

$^{1}$H NMR (300 MHz, $CDCl_3$) δ 8.32 – 8.23 (m, 1H), 7.45 – 7.31 (m, 5H), 7.28 – 7.24 (m, 1H), 7.19 – 7.15 (m, 2H), 6.93 – 6.85 (m, 1H), 1.63 (s, 6H).

$^{13}$C NMR (75 MHz, $CDCl_3$) δ 175.7, 144.0, 134.7, 129.0, 128.8, 127.6, 127.5, 126.5, 124.3, 122.8, 121.1, 48.3, 26.8.

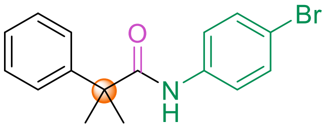

**3i**

**N-(4-bromophenyl)-2-methyl-2-phenylpropanamide (3i)** [1]

Chromatography Pentane/EA = 15:1 (v/v), 40.8 mg (64%).

$^{1}$H NMR (300 MHz, $CDCl_3$) δ 7.40 – 7.11 (m, 1H), 6.72 (s, 0H), 1.58 (s, 1H).

$^{13}$C NMR (75 MHz, $CDCl_3$) δ 175.6, 144.3, 137.0, 131.7, 129.0, 127.5, 126.4, 121.2, 116.6, 48.1, 26.9.

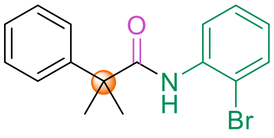


**3j**

**_N_-(2-chlorophenyl)-2-methyl-2-phenylpropanamide (3j) [1]**

Chromatography Pentane/EA = 15:1 (v/v), 55.4 mg (87%).

$^{1}$H NMR (300 MHz, $CDCl_3$) δ 8.26 (dd, *J* = 8.3, 1.6 Hz, 1H), 7.44 – 7.33 (m, 5H), 7.29 – 7.15 (m, 3H), 6.90 – 6.77 (m, 1H), 1.64 (s, 6H).

$^{13}$C NMR (75 MHz, $CDCl_3$) δ 175.8, 144.0, 135.9, 132.1, 129.1, 128.2, 127.5, 126.6, 124.8, 121.3, 113.3, 48.3, 26.8.

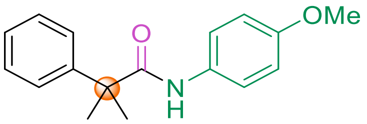


**3k**

**_N_-(4-methoxyphenyl)-2-methyl-2-phenylpropanamide (3k) [1]**

Chromatography Pentane/EA = 10:1 (v/v), 34.5 mg (64%).

$^{1}$H NMR (300 MHz, $CDCl_3$) δ 7.43 – 7.29 (m, 4H), 7.26 – 7.12 (m, 3H), 6.75 – 6.68 (m, 2H), 6.64 (s, 1H), 3.67 (s, 3H), 1.58 (s, 6H).

$^{13}$C NMR (75 MHz, $CDCl_3$) δ 175.4, 156.3, 144.7, 131.0, 128.9, 127.3, 126.5, 121.5, 114.0, 55.4, 47.8, 27.0.

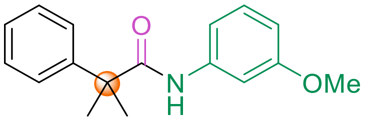


**3l**

**_N_-(3-methoxyphenyl)-2-methyl-2-phenylpropanamide (3l) [1]**

Chromatography Pentane/EA = 10:1 (v/v), 38.0 mg (71%).

$^{1}$H NMR (300 MHz, $CDCl_3$) δ 7.39 – 7.28 (m, 4H), 7.26 – 7.20 (m, 1H), 7.18 – 7.13 (m, 1H), 7.04 (t, *J* = 8.1 Hz, 1H), 6.73 (s, 1H), 6.69 – 6.59 (m, 1H), 6.58 – 6.46 (m, 1H), 3.68 (s, 3H), 1.58 (s, 6H).

$^{13}$C NMR (75 MHz, $CDCl_3$) δ 175.6, 160.1, 144.4, 139.2, 129.4, 129.0, 127.4, 126.4, 111.5, 110.2, 105.0, 55.2, 48.1, 27.0.

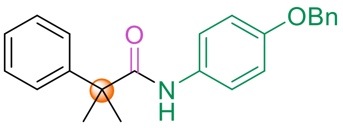

**3m**

***N*-(4-(benzyloxy)phenyl)-2-methyl-2-phenylpropanamide (3m)**

Chromatography Pentane/EA = 10:1 (v/v), 26.4 mg (38%).

$^{1}$H NMR (300 MHz, $CDCl_3$) δ 7.42 – 7.12 (m, 12H), 6.85 – 6.74 (m, 2H), 6.63 (s, 1H), 4.94 (s, 2H), 1.58 (s, 6H).

$^{13}$C NMR (75 MHz, $CDCl_3$) δ 175.4, 155.4, 144.7, 136.9, 131.3, 128.9, 128.5, 127.9, 127.4, 127.3, 126.5, 121.5, 115.1, 70.2, 47.8, 27.0.

HRMS (ES-TOF): m/z calcd. for $C_{23}H_{23}NO_2Na^+$ [M+Na$^+$] 368.1621, found 368.1623.

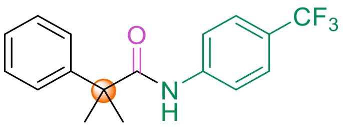

**3n**

**2-methyl-2-phenyl-*N*-(4-(trifluoromethyl)phenyl)propanamide (3n) [1]**

Chromatography Pentane/EA = 15:1 (v/v), 31.5 mg (51%).

$^{1}$H NMR (300 MHz, $CDCl_3$) δ 7.45 – 7.21 (m, 9H), 6.86 (s, 1H), 1.59 (s, 6H).

$^{13}$C NMR (75 MHz, $CDCl_3$) δ 175.9, 144.1, 141.0, 129.1, 127.6, 126.4, 126.1 (q, $J$ = 3.8 Hz), 124.0 (q, $J$ = 270.0 Hz), 119.1, 48.2, 26.9.

$^{19}$F NMR (282 MHz, $CDCl_3$) δ -62.10.

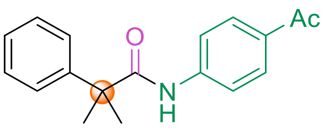

**3o**

***N*-(4-acetylphenyl)-2-methyl-2-phenylpropanamide (3o)**

Chromatography Pentane/EA = 3:1 (v/v), 39.4 mg (70%).

$^{1}$H NMR (300 MHz, $CDCl_3$) δ 7.82 – 7.71 (m, 2H), 7.41 – 7.20 (m, 7H), 7.08 – 6.98 (m, 1H), 2.43 (d, $J$ = 0.7 Hz, 3H), 1.59 (s, 6H).

$^{13}$C NMR (75 MHz, $CDCl_3$) δ 196.8, 175.8, 144.0, 142.3, 132.6, 129.5, 129.0, 127.5, 126.3, 118.7, 48.2, 26.8, 26.3.

HRMS (ES-TOF): m/z calcd. for $C_{18}H_{19}NO_2Na^+$ [M+Na$^+$] 304.1308, found 304.1313.

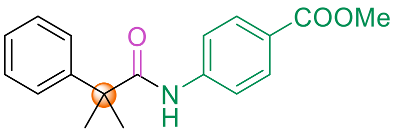


**3p**

**methyl 4-(2-methyl-2-phenylpropanamido)benzoate (3p)** [1]

Chromatography Pentane/EA = 8:1 (v/v), 35.1 mg (59%).

$^{1}$H NMR (300 MHz, $CDCl_3$) δ 7.91 – 7.79 (m, 0H), 7.44 – 7.21 (m, 1H), 6.90 (s, 0H), 3.79 (s, 1H), 1.59 (s, 1H).

$^{13}$C NMR (75 MHz, $CDCl_3$) δ 175.8, 166.5, 144.1, 142.1, 130.7, 129.1, 127.6, 126.4, 125.4, 118.6, 51.9, 48.2, 26.9.

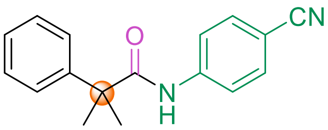


**3q**

***N*-(4-cyanophenyl)-2-methyl-2-phenylpropanamide (3q)** [1]

Chromatography Pentane/EA = 5:1 (v/v), 21.4 mg (41%).

$^{1}$H NMR (300 MHz, $CDCl_3$) δ 7.58 – 7.46 (m, 4H), 7.44 – 7.30 (m, 5H), 6.97 (s, 1H), 1.67 (s, 6H).

$^{13}$C NMR (75 MHz, $CDCl_3$) δ 175.9, 143.8, 141.9, 133.1, 129.2, 127.7, 126.4, 119.3, 118.8, 106.9, 48.3, 26.8.

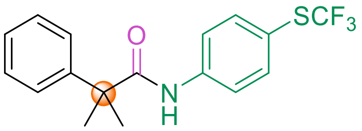


**3r**

**2-methyl-2-phenyl-*N*-(4-((trifluoromethyl)thio)phenyl)propanamide (3r)**

Chromatography Pentane/EA = 8:1 (v/v), 45.9 mg (68%).

$^{1}$H NMR (300 MHz, $CDCl_3$) δ 7.50 – 7.40 (m, 2H), 7.38 – 7.21 (m, 7H), 6.84 (s, 1H), 1.58 (s, 6H).

$^{13}$C NMR (75 MHz, $CDCl_3$) δ 175.8, 144.0, 140.5, 137.3, 129.5 (q, *J* = 307.5 Hz), 129.1, 127.6, 126.4, 120.0, 118.5 (q, *J* = 2.0 Hz), 48.2, 26.9.

$^{19}$F NMR (282 MHz, $CDCl_3$) δ -43.38.

HRMS (ES-TOF): m/z calcd. for $C_{17}H_{16}F_3NOSH^+$ [M+H $^+$] 340.0978, found 340.0980.

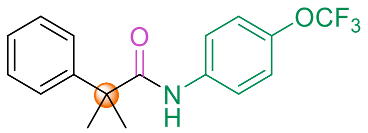


**3s**

**2-methyl-2-phenyl-*N*-(4-(trifluoromethoxy)phenyl)propanamide (3s)**

Chromatography Pentane/EA = 8:1 (v/v), 36.1 mg (56%).

$^{1}$H NMR (300 MHz, CDCl$_3$) δ 7.44 – 7.20 (m, 7H), 7.08 – 6.98 (m, 2H), 6.76 (s, 1H), 1.59 (s, 6H).

$^{13}$C NMR (75 MHz, CDCl$_3$) δ 175.7, 145.1, 144.3, 136.6, 129.1, 127.5, 126.4, 122.1, 121.6, 120.8, 48.0, 26.9.

$^{19}$F NMR (282 MHz, CDCl$_3$) δ -58.19.

HRMS (ES-TOF): m/z calcd. for $C_{17}H_{16}F_3NO_2H^+$ [M+H $^+$] 324.1206, found 324.1203.

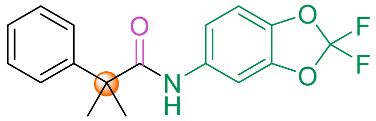


**3t**

***N*-(2,2-difluorobenzo[*d*][1,3]dioxol-5-yl)-2-methyl-2-phenylpropanamide (3t)**

Chromatography Pentane/EA = 5:1 (v/v), 42.2 mg (66%).

$^{1}$H NMR (300 MHz, CDCl$_3$) δ 7.49 (d, *J* = 2.1 Hz, 1H), 7.45 – 7.29 (m, 5H), 6.88 (dd, *J* = 8.6, 0.4 Hz, 1H), 6.84 (s, 1H), 6.72 (dd, *J* = 8.6, 2.1 Hz, 1H), 1.66 (s, 6H).

$^{13}$C NMR (75 MHz, CDCl$_3$) δ 175.7, 144.2, 143.8, 140.0, 134.0, 131.7 (t, *J* = 255.0 Hz), 129.1, 127.5, 126.4, 114.4, 109.0, 103.0, 48.0, 26.9.

$^{19}$F NMR (282 MHz, CDCl$_3$) δ -50.05.

HRMS (ES-TOF): m/z calcd. for $C_{17}H_{15}F_2NO_3H^+$ [M+H $^+$] 320.1093, found 320.1100.

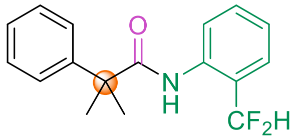


**3u**

***N*-(2-(difluoromethyl)phenyl)-2-methyl-2-phenylpropanamide (3u)**

Chromatography Pentane/EA = 15:1 (v/v), 54.8 mg (95%).

$^{1}$H NMR (300 MHz, CDCl$_3$) δ 8.27 (d, *J* = 8.2 Hz, 1H), 7.43 – 7.02 (m, 7H), 6.95 – 6.87 (m, 2H), 6.08 (t, *J* = 73.5 Hz, 1H), 1.60 (s, 6H).

$^{13}$C NMR (75 MHz, CDCl$_3$) δ 175.6, 144.0, 139.9, 130.4, 129.0, 127.4, 126.4, 126.2, 123.8, 121.0, 118.9, 115.9 (t, *J* = 255.0 Hz), 48.2, 26.7.

$^{19}$F NMR (282 MHz, CDCl$_3$) δ -80.24 (d, *J* = 73.6 Hz).

HRMS (ES-TOF): m/z calcd. for $C_{17}H_{18}F_2NO^+$ [M+H $^+$] 290.1351, found 290.1355.

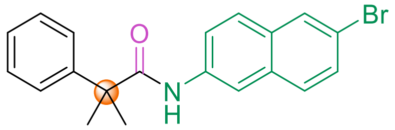

**3v**

***N*-(6-bromonaphthalen-2-yl)-2-methyl-2-phenylpropanamide (3v)**

Chromatography Pentane/EA = 15:1 (v/v), 25.6 mg (35%).

$^1$H NMR (300 MHz, $CDCl_3$) δ 8.13 (d, *J* = 2.2 Hz, 1H), 7.88 (d, *J* = 1.9 Hz, 1H), 7.61 (d, *J* = 8.7 Hz, 2H), 7.52 – 7.30 (m, 6H), 7.21 (dd, *J* = 8.8, 2.2 Hz, 1H), 6.96 (s, 1H), 1.71 (s, 6H).

$^{13}$C NMR (75 MHz, $CDCl_3$) δ 175.9, 144.4, 135.8, 132.3, 131.5, 129.9, 129.5, 129.3, 129.1, 127.7, 127.6, 126.5, 120.7, 118.7, 116.2, 48.2, 27.1.

HRMS (ES-TOF): m/z calcd. for $C_{20}H_{18}BrNONa^+$ [M+Na $^+$] 390.0464, found 390.0473.

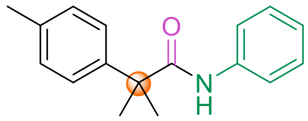

**3w**

**2-methyl-*N*-phenyl-2-(*p*-tolyl)propanamide (3w) [1]**

Chromatography Pentane/EA = 15:1 (v/v), 49.7 mg (98%).

$^1$H NMR (400 MHz, $CDCl_3$) δ 7.43 – 7.34 (m, 4H), 7.32 – 7.20 (m, 4H), 7.11 – 7.05 (m, 1H), 6.89 (s, 1H), 2.41 (s, 3H), 1.69 (s, 6H).

$^{13}$C NMR (101 MHz, $CDCl_3$) δ 175.8, 141.5, 138.0, 137.0, 129.6, 128.8, 126.3, 124.0, 119.5, 47.6, 27.0, 20.9.

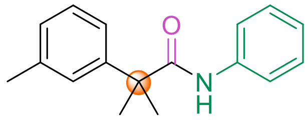

**3x**

**2-methyl-*N*-phenyl-2-(m-tolyl)propanamide (3x) [1]**

Chromatography Pentane/EA = 15:1 (v/v), 49.9 mg (99%).

$^1$H NMR (400 MHz, $CDCl_3$) δ 7.45 – 7.37 (m, 2H), 7.36 – 7.24 (m, 5H), 7.21 – 7.13 (m, 1H), 7.12 – 7.04 (m, 1H), 6.88 (s, 1H), 2.42 (s, 3H), 1.69 (s, 6H).

$^{13}$C NMR (101 MHz, $CDCl_3$) δ 175.6, 144.4, 138.6, 138.0, 128.8, 128.1, 127.1, 124.0, 123.4, 119.6, 47.9, 27.0, 21.6.

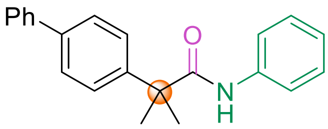


**3y**

**2-([1,1'-biphenyl]-4-yl)-2-methyl-*N*-phenylpropanamide (3y)** [1]

Chromatography Pentane/EA = 15:1 (v/v), 44.2 mg (70%).

$^{1}$H NMR (300 MHz, $CDCl_3$) δ 7.61 – 7.49 (m, 4H), 7.47 – 7.34 (m, 4H), 7.33 – 7.25 (m, 3H), 7.24 – 7.12 (m, 2H), 7.04 – 6.92 (m, 1H), 6.80 (s, 1H), 1.62 (s, 6H).

$^{13}$C NMR (75 MHz, $CDCl_3$) δ 175.4, 143.5, 140.3, 140.2, 137.9, 128.9, 128.8, 127.6, 127.5, 127.0, 126.9, 124.2, 119.7, 47.8, 27.0.

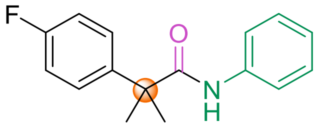


**3z**

**2-(4-fluorophenyl)-2-methyl-*N*-phenylpropanamide (3z)** [1]

Chromatography Pentane/EA = 15:1 (v/v), 48.8 mg (95%).

$^{1}$H NMR (300 MHz, $CDCl_3$) δ 7.42 – 7.21 (m, 1H), 7.20 – 7.12 (m, 0H), 7.08 – 6.91 (m, 1H), 6.75 (s, 0H), 1.56 (s, 1H).

$^{13}$C NMR (75 MHz, $CDCl_3$) δ 175.1, 161.8 (d, *J* = 246.7 Hz), 140.4 (d, *J* = 3.3 Hz), 137.8, 128.8, 128.1 (d, *J* = 8.0 Hz), 124.2, 119.7, 115.7 (d, *J* = 21.3 Hz), 47.5, 27.1.

$^{19}$F NMR (282 MHz, $CDCl_3$) δ -114.99 – -115.21 (m).

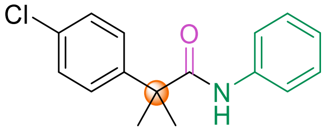


**3aa**

**2-(4-chlorophenyl)-2-methyl-*N*-phenylpropanamide (3aa)** [1]

Chromatography Pentane/EA = 15:1 (v/v), 50.5 mg (92%).

$^{1}$H NMR (300 MHz, $CDCl_3$) δ 7.34 – 7.24 (m, 6H), 7.22 – 7.14 (m, 2H), 7.05 – 6.92 (m, 1H), 6.75 (s, 1H), 1.55 (s, 6H).

$^{13}$C NMR (75 MHz, $CDCl_3$) δ 174.8, 143.1, 137.7, 133.2, 129.0, 128.8, 127.8, 124.3, 119.7, 47.6, 26.9.

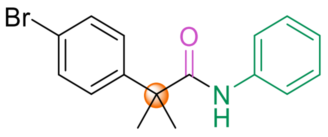

**3ab**

**2-(4-bromophenyl)-2-methyl-*N*-phenylpropanamide (3ab)** [1]

Chromatography Pentane/EA = 15:1 (v/v), 51.8 mg (82%).

$^{1}$H NMR (300 MHz, $CDCl_3$) δ 7.48 – 7.36 (m, 2H), 7.33 – 7.11 (m, 6H), 7.05 – 6.92 (m, 1H), 6.75 (s, 1H), 1.54 (s, 6H).

$^{13}$C NMR (75 MHz, $CDCl_3$) δ 174.7, 143.7, 137.7, 132.0, 128.8, 128.2, 124.3, 121.3, 119.7, 47.7, 26.9.

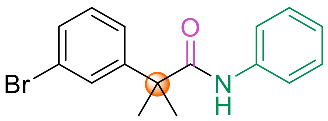

**3ac**

**2-(3-bromophenyl)-2-methyl-*N*-phenylpropanamide (3ac)** [1]

Chromatography Pentane/EA = 15:1 (v/v), 39.4 mg (62%).

$^{1}$H NMR (300 MHz, $CDCl_3$) δ 7.55 – 7.47 (m, 1H), 7.42 – 7.32 (m, 1H), 7.32 – 7.25 (m, 3H), 7.23 – 7.14 (m, 3H), 7.05 – 6.94 (m, 1H), 6.73 (s, 1H), 1.56 (s, 6H).

$^{13}$C NMR (75 MHz, $CDCl_3$) δ 174.6, 147.1, 137.8, 130.6, 129.4, 129.0, 125.4, 124.4, 123.2, 119.9, 48.0, 27.0.

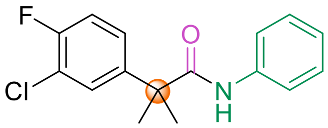

**3ad**

**2-(3-chloro-4-fluorophenyl)-2-methyl-*N*-phenylpropanamide (3ad)**

Chromatography Pentane/EA = 15:1 (v/v), 41.6 mg (71%).

$^{1}$H NMR (300 MHz, $CDCl_3$) δ 7.39 (dd, *J* = 6.9, 2.4 Hz, 0H), 7.33 – 7.26 (m, 0H), 7.24 – 7.16 (m, 1H), 7.12 – 6.93 (m, 0H), 6.82 – 6.73 (m, 0H), 1.56 (s, 1H).

$^{13}$C NMR (75 MHz, $CDCl_3$) δ 174.3, 157.2 (d, *J* = 249.5 Hz), 141.9 (d, *J* = 4.0 Hz), 137.6, 128.9, 128.6, 126.4 (d, *J* = 7.1 Hz), 124.4, 121.3 (d, *J* = 17.8 Hz), 119.8, 117.0 (d, *J* = 21.1 Hz), 47.4, 27.0.

$^{19}$F NMR (282 MHz, $CDCl_3$) δ -107.73 – -125.80 (m).

HRMS (ES-TOF): m/z calcd. for $C_{16}H_{15}ClFNONa^{+}$ [M+Na $^{+}$] 314.0718, found 314.0717.

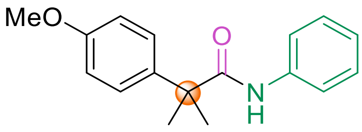

**3ae**

**2-(4-methoxyphenyl)-2-methyl-*N*-phenylpropanamide (3ae)** [1]

Chromatography Pentane/EA = 10:1 (v/v), 21.6 mg (40%).

$^{1}$H NMR (300 MHz, $CDCl_3$) δ 7.34 – 7.24 (m, 4H), 7.21 – 7.13 (m, 2H), 7.03 – 6.91 (m, 1H), 6.91 – 6.81 (m, 2H), 6.76 (s, 1H), 3.75 (s, 3H), 1.57 (s, 6H).

$^{13}$C NMR (75 MHz, $CDCl_3$) δ 175.9, 158.7, 138.0, 136.5, 128.8, 127.7, 124.0, 119.5, 114.3, 55.3, 47.4, 27.1.

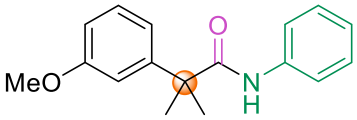

**3af**

**2-(3-methoxyphenyl)-2-methyl-*N*-phenylpropanamide (3af)** [1]

Chromatography Pentane/EA = 10:1 (v/v), 41.7 mg (77%).

$^{1}$H NMR (300 MHz, $CDCl_3$) δ 7.33 – 7.10 (m, 2H), 7.02 – 6.86 (m, 1H), 6.82 – 6.72 (m, 1H), 3.73 (s, 1H), 1.57 (s, 2H).

$^{13}$C NMR (75 MHz, $CDCl_3$) δ 175.3, 159.9, 146.2, 137.9, 130.0, 128.8, 124.0, 119.6, 118.8, 112.8, 112.1, 55.2, 48.0, 26.9.

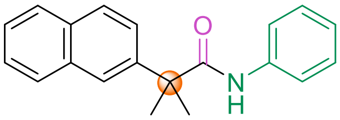

**3ag**

**2-methyl-2-(naphthalen-2-yl)-*N*-phenylpropanamide (3ag)** [1]

Chromatography Pentane/EA = 10:1 (v/v), 36.4 mg (63%).

$^{1}$H NMR (300 MHz, $CDCl_3$) δ 7.89 – 7.70 (m, 4H), 7.50 – 7.35 (m, 3H), 7.29 – 7.19 (m, 2H), 7.18 – 7.06 (m, 2H), 7.00 – 6.86 (m, 1H), 6.74 (s, 1H), 1.67 (s, 6H).

$^{13}$C NMR (75 MHz, $CDCl_3$) δ 175.4, 142.0, 137.9, 133.3, 132.4, 128.8, 128.0, 127.6, 126.5, 126.3, 125.3, 124.5, 124.1, 119.6, 48.2, 26.9.

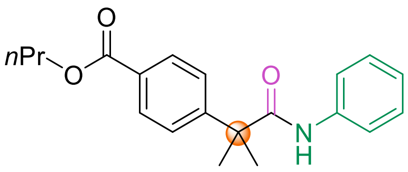

**3ah**

**propyl 4-(2-methyl-1-oxo-1-(phenylamino)propan-2-yl)benzoate (3ah)**

Chromatography Pentane/EA = 6:1 (v/v), 30.1 mg (46%).

$^{1}$H NMR (300 MHz, $CDCl_3$) δ 8.04 – 7.94 (m, 1H), 7.50 – 7.39 (m, 1H), 7.33 – 7.25 (m, 1H), 7.23 – 7.14 (m, 1H), 7.05 – 6.93 (m, 0H), 6.70 (s, 1H), 4.21 (t, *J* = 6.6 Hz, 1H), 1.79 – 1.66 (m, 1H), 1.61 (s, 3H), 0.96 (t, *J* = 7.4 Hz, 1H).

$^{13}$C NMR (75 MHz, $CDCl_3$) δ 174.6, 166.2, 149.7, 137.7, 130.1, 129.6, 128.9, 126.4, 124.3, 119.7, 66.6, 48.3, 26.9, 22.1, 10.5.

HRMS (ES-TOF): m/z calcd. for $C_{20}H_{23}NO_3Na^+$ [M+Na$^+$] 348.1570, found 348.1578.

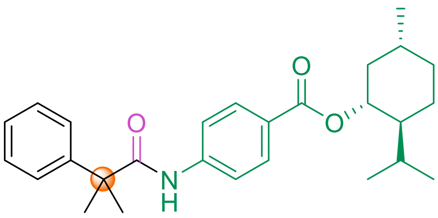

**5**

**(1*R*,2*S*,5*R*)-2-isopropyl-5-methylcyclohexyl 4-(2-methyl-2-phenylpropanamido)benzoate (5)**

Chromatography Pentane/EA = 10:1 (v/v), 73.8 mg (88%).

$^{1}$H NMR (300 MHz, $CDCl_3$) δ 8.01 – 7.89 (m, 2H), 7.65 – 7.32 (m, 7H), 7.03 (s, 1H), 4.98 – 4.84 (m, 1H), 2.18 – 2.04 (m, 1H), 2.02 – 1.85 (m, 1H), 1.77 – 1.66 (s, 8H), 1.59 – 1.47 (m, 2H), 1.20 – 1.03 (m, 2H), 0.99 – 0.83 (m, 7H), 0.79 (d, *J* = 7.0 Hz, 3H).

$^{13}$C NMR (75 MHz, $CDCl_3$) δ 175.7, 165.5, 144.1, 141.9, 130.6, 129.0, 127.5, 126.3, 126.1, 118.6, 74.6, 48.2, 47.2, 40.9, 34.2, 31.4, 26.9, 26.5, 23.7, 22.0, 20.6, 16.5.

HRMS (ES-TOF): m/z calcd. for $C_{27}H_{35}NO_3Na^+$ [M+Na$^+$] 444.2509, found 444.2515.

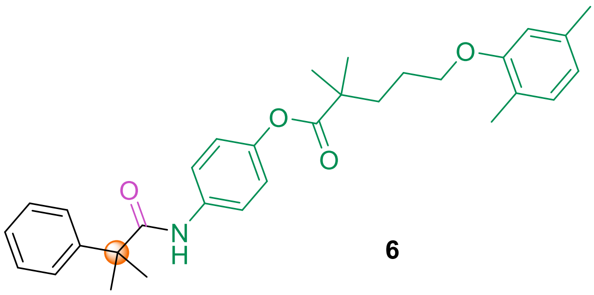

**6**

4-(2-methyl-2-phenylpropanamido)phenyl 5-(2,5-dimethylphenoxy)-2,2-dimethylpentanoate (6)

Chromatography Pentane/EA = 8:1 (v/v), 78.5 mg (81%).

$^{1}$H NMR (300 MHz, $CDCl_3$) δ 7.53 – 7.30 (m, 7H), 7.04 (dd, *J* = 7.4, 0.9 Hz, 1H), 7.00 – 6.88 (m, 3H), 6.76 – 6.63 (m, 2H), 4.09 – 3.95 (m, 2H), 2.35 (s, 3H), 2.22 (s, 3H), 1.91 (d, *J* = 2.9 Hz, 4H), 1.71 (s, 6H), 1.40 (s, 6H).

$^{13}C$ NMR (75 MHz, $CDCl_3$) δ 176.4, 175.5, 156.8, 147.0, 144.5, 136.4, 135.5, 130.3, 128.9, 127.3, 126.4, 123.5, 121.7, 120.7, 120.5, 111.9, 67.7, 47.9, 42.3, 37.0, 26.9, 25.2, 25.1, 21.3, 15.7.

HRMS (ES-TOF): m/z calcd. for $C_{31}H_{37}NO_4Na^+$ [M+Na$^+$] 510.2614, found 510.2619.

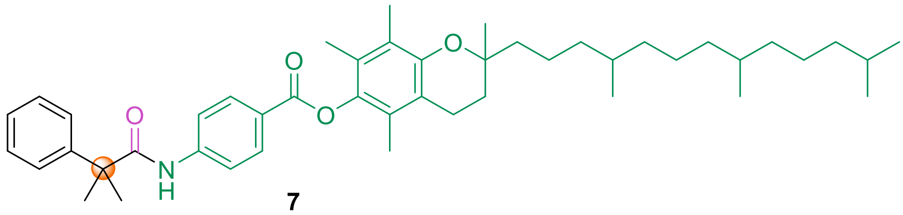

**7**

**2,5,7,8-tetramethyl-2-(4,8,12-trimethyltridecyl)chroman-6-yl 4-(2-methyl-2-phenylpropanamido)benzoate (7)**

Chromatography Pentane/EA = 10:1 (v/v), 110.0 mg (79%).

$^1H$ NMR (300 MHz, $CDCl_3$) δ 8.20 – 8.12 (m, 2H), 7.59 – 7.30 (m, 7H), 7.07 (s, 1H), 2.63 (t, *J* = 6.8 Hz, 2H), 2.14 (s, 3H), 2.03 (d, *J* = 12.8 Hz, 6H), 1.82 (q, *J* = 8.1 Hz, 2H), 1.71 (s, 6H), 1.63 – 1.05 (m, 24H), 0.96 – 0.82 (m, 12H).

$^{13}C$ NMR (75 MHz, $CDCl_3$) δ 175.8, 164.6, 149.4, 144.1, 142.6, 140.5, 131.3, 129.1, 127.6, 126.8, 126.3, 125.1, 124.8, 123.0, 118.8, 117.4, 75.0, 48.3, 39.3, 37.4, 37.3, 37.2, 32.7, 32.6, 27.9, 26.9, 24.8, 24.4, 22.7, 22.6, 21.0, 20.6, 19.7, 19.6, 19.6, 19.6, 19.6, 13.0, 12.1, 11.8.

HRMS (ES-TOF): m/z calcd. for $C_{46}H_{65}NO_4Na^+$ [M+Na$^+$] 718.4805, found 718.4818.

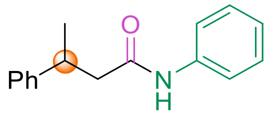

**4a**

***N*,3-diphenylbutanamide (4a)**

Chromatography Pentane/EA = 8:1 (v/v), 46.7 mg (98%).

$^1H$ NMR (300 MHz, $CDCl_3$) δ 7.57 – 7.47 (m, 1H), 7.33 – 7.22 (m, 2H), 7.21 – 7.05 (m, 7H), 7.02 – 6.91 (m, 1H), 3.26 (h, *J* = 7.1 Hz, 1H), 2.60 – 2.39 (m, 2H), 1.24 (d, *J* = 7.0 Hz, 3H).

$^{13}C$ NMR (75 MHz, $CDCl_3$) δ 170.4, 145.6, 137.7, 128.8, 128.6, 126.7, 126.5, 124.2, 120.1, 46.4, 36.9, 21.6.

HRMS (ES-TOF): m/z calcd. for $C_{16}H_{17}NONa^+$ [M+Na$^+$] 262.1202, found 262.1203.

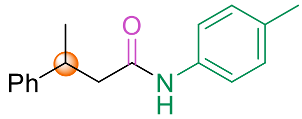

**4b**

**3-phenyl-*N*-(p-tolyl)butanamide (4b)[2]**

Chromatography Pentane/EA = 10:1 (v/v), 47.6 mg (94%).

$^1H$ NMR (300 MHz, $CDCl_3$) δ 7.49 – 7.43 (m, 1H), 7.39 – 7.19 (m, 7H), 7.08 (d, *J* = 8.1 Hz, 2H), 3.39 (h, *J* = 7.1 Hz, 1H), 2.71 – 2.51 (m, 2H), 2.32 (s, 3H), 1.38 (d, *J* = 7.0 Hz, 3H).

$^{13}C$ NMR (75 MHz, $CDCl_3$) δ 170.2, 145.7, 135.1, 133.8, 129.2, 128.6, 126.7, 126.4, 120.3, 46.3, 36.9, 21.5, 20.8.

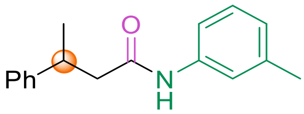

**4c**

**3-phenyl-*N*-(*m*-tolyl)butanamide (4c)**

Chromatography Pentane/EA = 8:1 (v/v), 43.4 mg (86%).

$^{1}$H NMR (300 MHz, $CDCl_3$) δ 7.51 (s, 1H), 7.41 – 7.22 (m, 6H), 7.20 – 7.13 (m, 2H), 6.97 – 6.86 (m, 1H), 3.40 (h, *J* = 7.1 Hz, 1H), 2.73 – 2.52 (m, 2H), 2.31 (s, 3H), 1.38 (d, *J* = 7.0 Hz, 3H).

$^{13}$C NMR (75 MHz, $CDCl_3$) δ 170.2, 145.6, 138.7, 137.6, 128.6, 126.7, 126.5, 125.0, 120.8, 117.2, 46.4, 36.9, 21.3.

HRMS (ES-TOF): m/z calcd. for $C_{17}H_{19}NONa^+$ [M+Na $^+$] 276.1359, found 276.1356.

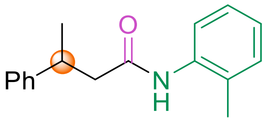

**4d**

**3-phenyl-*N*-(o-tolyl)butanamide (4d)**

Chromatography Pentane/EA = 8:1 (v/v), 39.7 mg (78%).

$^{1}$H NMR (300 MHz, $CDCl_3$) δ 7.60 (d, *J* = 7.9 Hz, 1H), 7.41 – 7.21 (m, 5H), 7.21 – 6.99 (m, 4H), 3.39 (h, *J* = 7.1 Hz, 1H), 2.76 – 2.57 (m, 2H), 1.98 (s, 3H), 1.40 (d, *J* = 7.0 Hz, 3H).

$^{13}$C NMR (75 MHz, $CDCl_3$) δ 170.1, 145.5, 135.4, 130.3, 129.7, 128.7, 126.8, 126.5, 126.4, 125.3, 123.6, 46.3, 37.2, 22.0, 17.4.

HRMS (ES-TOF): m/z calcd. for $C_{17}H_{19}NONa^+$ [M+Na $^+$] 276.1359, found 276.1364.

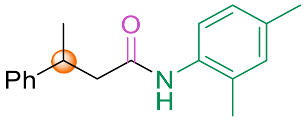

**4e**

***N*-(2,4-dimethylphenyl)-3-phenylbutanamide (4e)**

Chromatography Pentane/EA = 8:1 (v/v), 45.0 mg (84%).

$^{1}$H NMR (300 MHz, $CDCl_3$) δ 7.32 – 7.06 (m, 6H), 6.83 (d, *J* = 7.9 Hz, 3H), 3.26 (h, *J* = 7.2 Hz, 1H), 2.64 – 2.43 (m, 2H), 2.16 (s, 3H), 1.82 (s, 3H), 1.27 (d, *J* = 7.0 Hz, 3H).

$^{13}$C NMR (75 MHz, $CDCl_3$) δ 170.2, 145.6, 135.0, 132.7, 130.9, 130.1, 128.7, 127.0, 126.8, 126.5, 123.9, 46.2, 37.2, 22.0, 20.8, 17.3.

HRMS (ES-TOF): m/z calcd. for $C_{18}H_{21}NONa^+$ [M+Na $^+$] 290.1515, found 290.1522.

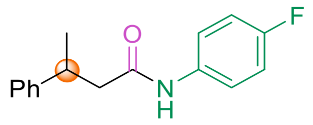


**4f**

***N*-(4-fluorophenyl)-3-phenylbutanamide (4f)**

Chromatography Pentane/EA = 8:1 (v/v), 48.3 mg (94%).

$^{1}$H NMR (300 MHz, $CDCl_3$) δ 7.32 – 7.05 (m, 8H), 6.90 – 6.76 (m, 2H), 3.26 (h, *J* = 7.1 Hz, 1H), 2.59 – 2.40 (m, 2H), 1.26 (d, *J* = 7.0 Hz, 3H).

$^{13}$C NMR (75 MHz, $CDCl_3$) δ 170.2, 159.3 (d, *J* = 243.5 Hz), 145.5, 133.6 (d, *J* = 2.8 Hz), 128.7, 126.7, 126.6, 122.0 (d, *J* = 8.0 Hz), 115.4 (d, *J* = 22.4 Hz), 46.4, 37.0, 21.6.

19F NMR (282 MHz, CDCl3) δ -117.77 – -118.01 (m).

HRMS (ES-TOF): m/z calcd. for $C_{16}H_{16}FNONa^{+}$ [M+Na $^{+}$] 280.1108, found 280.1114.

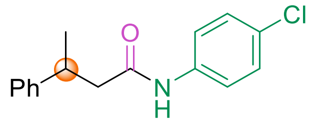


**4g**

***N*-(4-chlorophenyl)-3-phenylbutanamide (4g)**

Chromatography Pentane/EA = 8:1 (v/v), 49.7 mg (91%).

$^{1}$H NMR (300 MHz, $CDCl_3$) δ 7.44 – 6.53 (m, 10H), 3.26 (h, *J* = 7.1 Hz, 1H), 2.60 – 2.41 (m, 2H), 1.27 (d, *J* = 6.9 Hz, 3H).

$^{13}$C NMR (75 MHz, $CDCl_3$) δ 170.2, 145.4, 136.1, 129.2, 128.8, 128.7, 126.7, 126.7, 121.3, 46.5, 37.0, 21.6.

HRMS (ES-TOF): m/z calcd. for $C_{16}H_{16}ClNONa^{+}$ [M+Na $^{+}$] 296.0812, found 296.0820.

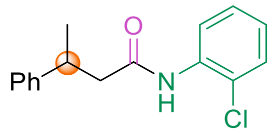


**4h**

***N*-(2-chlorophenyl)-3-phenylbutanamide (4h)**

Chromatography Pentane/EA = 8:1 (v/v), 50.6 mg (93%).

$^{1}$H NMR (400 MHz, $CDCl_3$) δ 8.34 (d, *J* = 8.3 Hz, 1H), 7.51 (s, 1H), 7.43 – 7.21 (m, 7H), 7.08 – 6.99 (m, 1H), 3.43 (h, *J* = 7.1 Hz, 1H), 2.76 – 2.63 (m, 2H), 1.42 (d, *J* = 6.9 Hz, 3H).

$^{13}$C NMR (101 MHz, $CDCl_3$) δ 169.9, 145.3, 134.4, 128.8, 128.7, 127.5, 126.7, 126.6, 124.5, 121.7, 46.7, 36.9, 21.8.

HRMS (ES-TOF): m/z calcd. for $C_{16}H_{16}ClNONa^{+}$ [M+Na $^{+}$] 296.0812, found 296.0817.

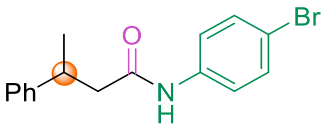

**4i**

***N*-(4-bromophenyl)-3-phenylbutanamide (4ai)**

Chromatography Pentane/EA = 10:1 (v/v), 60.5 mg (95%).

$^{1}$H NMR (300 MHz, $CDCl_3$) δ 7.32 – 7.20 (m, 4H), 7.21 – 7.09 (m, 5H), 6.98 (s, 1H), 3.27 (h, *J* = 7.1 Hz, 1H), 2.61 – 2.42 (m, 2H), 1.28 (d, *J* = 7.0 Hz, 3H).

$^{13}$C NMR (75 MHz, $CDCl_3$) δ 170.1, 145.4, 136.6, 131.8, 128.8, 126.7, 126.7, 121.5, 116.8, 46.7, 37.0, 21.7.

HRMS (ES-TOF): m/z calcd. for $C_{16}H_{16}BrNONa^{+}$ [M+Na$^{+}$] 340.0307, found 340.0315.

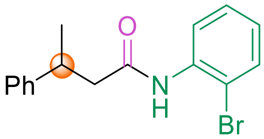

**4j**

***N*-(4-bromophenyl)-3-phenylbutanamide (4j)**

Chromatography Pentane/EA = 8:1 (v/v), 57.0 mg (90%).

$^{1}$H NMR (300 MHz, $CDCl_3$) δ 8.23 – 8.14 (m, 1H), 7.43 – 7.34 (m, 2H), 7.25 – 7.01 (m, 6H), 6.90 – 6.79 (m, 1H), 3.31 (h, 1H), 2.71 – 2.48 (m, 2H), 1.30 (d, *J* = 7.0 Hz, 3H).

$^{13}$C NMR (75 MHz, $CDCl_3$) δ 169.9, 145.4, 135.5, 132.1, 128.7, 128.2, 126.7, 126.6, 125.1, 122.0, 113.3, 46.7, 36.9, 21.8.

HRMS (ES-TOF): m/z calcd. for $C_{16}H_{16}BrNONa^{+}$ [M+Na$^{+}$] 340.0307, found 340.0311.

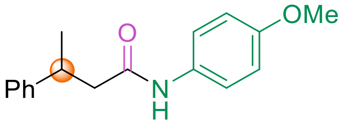

**4k**

***N*-(4-methoxyphenyl)-3-phenylbutanamide (4k)**

Chromatography Pentane/EA = 5:1 (v/v), 51.4 mg (95%).

$^{1}$H NMR (300 MHz, $CDCl_3$) δ 7.53 – 7.38 (m, 1H), 7.27 – 7.05 (m, 1H), 6.78 – 6.62 (m, 2H), 3.65 (s, 3H), 3.26 (h, *J* = 7.1 Hz, 1H), 2.57 – 2.38 (m, 2H), 1.24 (d, *J* = 7.0 Hz, 3H).

$^{13}$C NMR (75 MHz, $CDCl_3$) δ 170.2, 156.3, 145.7, 130.7, 128.6, 126.7, 126.4, 122.1, 113.9, 55.3, 46.2, 37.0, 21.5.

HRMS (ES-TOF): m/z calcd. for $C_{17}H_{19}NO_2Na^{+}$ [M+Na$^{+}$] 292.1308, found 292.1316.

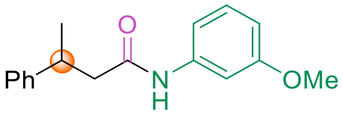

**4l**

***N*-(3-methoxyphenyl)-3-phenylbutanamide (4l)**

Chromatography Pentane/EA = 5:1 (v/v), 47.1 mg (88%).

$^{1}$H NMR (300 MHz, $CDCl_3$) δ 7.34 (s, 1H), 7.27 – 6.99 (m, 7H), 6.80 – 6.70 (m, 1H), 6.58 – 6.48 (m, 1H), 3.64 (s, 3H), 3.27 (h, *J* = 7.1 Hz, 1H), 2.60 – 2.40 (m, 2H), 1.25 (d, *J* = 6.9 Hz, 3H).

$^{13}$C NMR (75 MHz, $CDCl_3$) δ 170.3, 159.9, 145.6, 138.9, 129.5, 128.6, 126.7, 126.5, 112.1, 110.0, 105.7, 55.1, 46.5, 36.9, 21.6.

HRMS (ES-TOF): m/z calcd. for $C_{17}H_{19}NO_2Na^+$ [M+Na $^+$] 292.1308, found 292.1310.

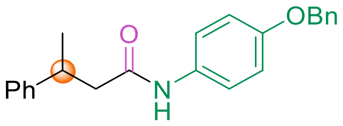

**4m**

***N*-(4-(benzyloxy)phenyl)-3-phenylbutanamide (4m)**

Chromatography Pentane/EA = 5:1 (v/v), 59.7 mg (86%).

$^{1}$H NMR (300 MHz, $CDCl_3$) δ 7.35 – 7.05 (m, 13H), 6.81 – 6.69 (m, 2H), 4.89 (s, 2H), 3.26 (h, *J* = 7.1 Hz, 1H), 2.57 – 2.38 (m, 2H), 1.18 (d, 3H).

$^{13}$C NMR (75 MHz, $CDCl_3$) δ 170.2, 155.6, 145.6, 136.9, 130.9, 128.6, 128.5, 127.9, 127.4, 126.8, 126.5, 122.1, 115.0, 70.2, 46.3, 37.0, 21.6.

HRMS (ES-TOF): m/z calcd. for $C_{23}H_{23}NO_2Na^+$ [M+Na $^+$] 368.1612, found 368.1621.

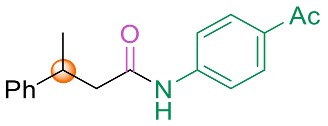

**4n**

***N*-(4-acetylphenyl)-3-phenylbutanamide (4n)**

Chromatography Pentane/EA = 3:1 (v/v), 48.0 mg (85%).

$^{1}$H NMR (300 MHz, $CDCl_3$) δ 7.89 (s, 1H), 7.82 – 7.72 (m, 2H), 7.47 – 7.37 (m, 2H), 7.26 – 7.06 (m, 5H), 3.29 (h, *J* = 7.1 Hz, 1H), 2.67 – 2.47 (m, 2H), 2.45 (s, 3H), 1.27 (d, *J* = 7.0 Hz, 3H).

$^{13}$C NMR (75 MHz, $CDCl_3$) δ 197.3, 170.7, 145.4, 142.3, 132.5, 129.6, 128.7, 126.7, 126.6, 119.0, 46.5, 36.9, 26.4, 21.6.

HRMS (ES-TOF): m/z calcd. for $C_{18}H_{19}NO_2Na^+$ [M+Na $^+$] 304.1308, found 304.1316.

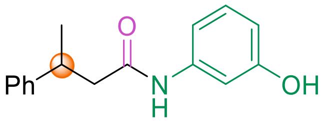


**4o**

***N*-(3-hydroxyphenyl)-3-phenylbutanamide (4o)**

Chromatography Pentane/EA = 2:1 (v/v), 20.6 mg (40%).

$^{1}$H NMR (300 MHz, $CDCl_3$) δ 7.77 (t, *J* = 2.2 Hz, 1H), 7.30 – 7.09 (m, 5H), 7.00 (t, *J* = 8.1 Hz, 1H), 6.92 (s, 1H), 6.61 – 6.51 (m, 1H), 6.19 – 6.10 (m, 1H), 3.27 (h, *J* = 7.1 Hz, 1H), 2.67 – 2.44 (m, 2H), 1.29 (d, *J* = 6.9 Hz, 3H).

$^{13}$C NMR (75 MHz, $CDCl_3$) δ 171.2, 157.6, 145.0, 138.2, 129.6, 128.9, 126.8, 126.8, 112.0, 110.4, 107.6, 47.1, 37.2, 21.6.

HRMS (ES-TOF): m/z calcd. for $C_{16}H_{17}NO_2Na^+$ [M+Na $^+$] 278.1151, found 278.1156.

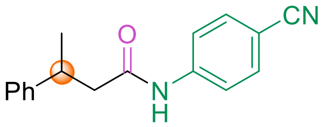


**4p**

***N*-(4-cyanophenyl)-3-phenylbutanamide (4p)**

Chromatography Pentane/EA = 3:1 (v/v), 48.8 mg (92%).

$^{1}$H NMR (300 MHz, $CDCl_3$) δ 7.51 (s, 1H), 7.46 – 7.39 (m, 4H), 7.28 – 7.19 (m, 2H), 7.19 – 7.11 (m, 3H), 3.28 (h, *J* = 7.1 Hz, 1H), 2.67 – 2.47 (m, 2H), 1.29 (d, *J* = 7.0 Hz, 3H).

$^{13}$C NMR (75 MHz, $CDCl_3$) δ 170.6, 145.2, 141.9, 133.1, 128.8, 126.7, 126.7, 119.5, 118.9, 106.6, 46.6, 36.9, 21.7.

HRMS (ES-TOF): m/z calcd. for $C_{17}H_{16}N_2ONa^+$ [M+Na $^+$] 287.1154, found 287.1161.

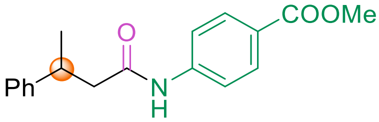


**4q**

**methyl 4-(3-phenylbutanamido)benzoate (4q)**

Chromatography Pentane/EA = 5:1 (v/v), 52.2 mg (88%).

$^{1}$H NMR (300 MHz, $CDCl_3$) δ 7.90 – 7.81 (m, 2H), 7.71 – 7.60 (m, 1H), 7.43 – 7.33 (m, 2H), 7.27 – 7.06 (m, 5H), 3.78 (s, 3H), 3.28 (h, *J* = 7.1 Hz, 1H), 2.65 – 2.44 (m, 2H), 1.26 (d, *J* = 7.0 Hz, 3H).

$^{13}$C NMR (75 MHz, $CDCl_3$) δ 170.5, 166.7, 145.3, 142.0, 130.6, 128.7, 126.7, 126.6, 125.3, 118.9, 52.0, 46.5, 36.9, 21.6.

HRMS (ES-TOF): m/z calcd. for $C_{18}H_{19}NO_3Na^+$ [M+Na $^+$] 320.1257, found 320.1263.

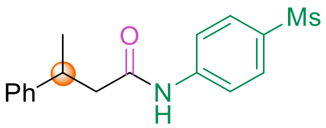


**4r**

***N*-(4-(methylsulfonyl)phenyl)-3-phenylbutanamide (4r)**

Chromatography Pentane/EA = 2:1 (v/v), 48.1 mg (76%).

$^1$H NMR (300 MHz, $CDCl_3$) δ 7.85 – 7.45 (m, 5H), 7.38 – 7.18 (m, 5H), 3.45 – 3.26 (m, 1H), 3.03 (s, 3H), 2.74 – 2.58 (m, 2H), 1.42 – 1.25 (m, 3H).

$^{13}$C NMR (75 MHz, $CDCl_3$) δ 170.7, 145.3, 142.8, 134.8, 128.7, 128.4, 126.7, 126.7, 119.6, 46.5, 44.6, 36.9, 21.7.

HRMS (ES-TOF): m/z calcd. for $C_{17}H_{19}NO_3SNa^+$ [M+Na $^+$] 340.0977, found 340.0979.

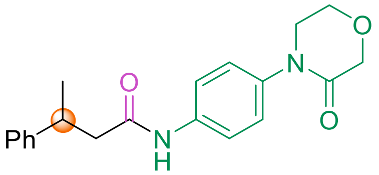


**4s**

***N*-(4-(3-oxomorpholino)phenyl)-3-phenylbutanamide (4s)**

Chromatography Pentane/EA = 2:1 (v/v), 26.6 mg (39%).

$^1$H NMR (300 MHz, $CDCl_3$) δ 8.00 (s, 1H), 7.29 – 7.09 (m, 7H), 6.98 – 6.88 (m, 2H), 4.23 (s, 2H), 3.97 – 3.88 (m, 2H), 3.63 – 3.54 (m, 2H), 3.28 (h, *J* = 7.1 Hz, 1H), 2.56 – 2.36 (m, 2H), 1.26 (d, *J* = 7.0 Hz, 3H).

$^{13}$C NMR (75 MHz, $CDCl_3$) δ 170.4, 167.1, 145.9, 137.1, 136.5, 128.6, 126.8, 126.5, 125.9, 121.2, 68.4, 64.0, 49.9, 46.1, 36.8, 21.5.

HRMS (ES-TOF): m/z calcd. for $C_{20}H_{22}N_2O_3Na^+$ [M+Na $^+$] 361.1523, found 361.1519.

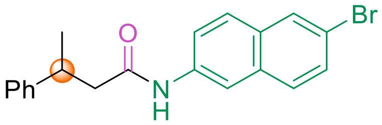


**4t**

***N*-(6-bromonaphthalen-2-yl)-3-phenylbutanamide (4t)**

Chromatography Pentane/EA = 10:1 (v/v), 54.2 mg (74%).

$^1$H NMR (300 MHz, $CDCl_3$) δ 7.92 (d, *J* = 2.2 Hz, 1H), 7.81 – 7.69 (m, 1H), 7.47 – 7.03 (m, 10H), 3.30 (h, *J* = 7.1 Hz, 1H), 2.65 – 2.45 (m, 2H), 1.28 (d, *J* = 7.0 Hz, 3H).

$^{13}$C NMR (75 MHz, $CDCl_3$) δ 170.4, 145.5, 135.4, 132.1, 129.7, 129.4, 129.1, 128.7, 127.6, 126.7, 126.6, 120.9, 119.0, 118.7, 116.7, 46.6, 37.0, 21.7.

HRMS (ES-TOF): m/z calcd. for $C_{20}H_{18}BrNONa^+$ [M+Na $^+$] 390.0464, found 390.0467.

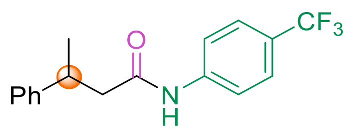

**4u**

**3-phenyl-*N*-(4-(trifluoromethyl)phenyl)butanamide (4u)**

Chromatography Pentane/EA = 10:1 (v/v), 56.9 mg (93%).

$^{1}$H NMR (300 MHz, $CDCl_3$) δ 7.54 – 7.44 (m, 1H), 7.43 – 7.30 (m, 4H), 7.26 – 7.17 (m, 2H), 7.17 – 7.09 (m, 3H), 3.26 (h, *J* = 7.1 Hz, 1H), 2.64 – 2.44 (m, 2H), 1.26 (d, *J* = 7.0 Hz, 3H).

$^{13}$C NMR (75 MHz, $CDCl_3$) δ 170.6, 145.3, 140.6, 128.8, 126.7, 126.7, 126.0 (q, *J* = 3.8 Hz), 124.0 (q, *J* = 270.0 Hz), 119.6, 46.6, 37.0, 21.7.

$^{19}$F NMR (282 MHz, $CDCl_3$) δ -62.11.

HRMS (ES-TOF): m/z calcd. for $C_{17}H_{16}F_3NONa^+$ [M+Na $^+$] 330.1076, found 330.1078.

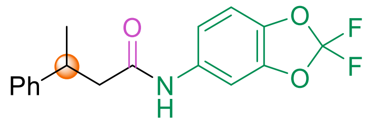

**4v**

***N*-(2,2-difluorobenzo[*d*][1,3]dioxol-5-yl)-3-phenylbutanamide (4v)**

Chromatography Pentane/EA = 5:1 (v/v), 60.2 mg (94%).

$^{1}$H NMR (300 MHz, $CDCl_3$) δ 7.32 (s, 1H), 7.27 – 7.18 (m, 3H), 7.18 – 7.08 (m, 3H), 6.78 (d, *J* = 8.6 Hz, 1H), 6.65 (dd, *J* = 8.6, 2.1 Hz, 1H), 3.26 (h, *J* = 7.1 Hz, 1H), 2.51 (dd, *J* = 7.4, 1.0 Hz, 2H), 1.27 (d, *J* = 7.0 Hz, 3H).

13C NMR (75 MHz, CDCl3) δ 170.5, 145.3, 143.6, 140.1, 133.6, 131.7 (t, *J* = 253.5 Hz), 128.7, 126.7, 126.7, 115.1, 109.0, 103.4, 46.4, 37.1, 21.6.

$^{19}$F NMR (282 MHz, $CDCl_3$) δ -49.98.

HRMS (ES-TOF): m/z calcd. for $C_{17}H_{15}F_2NO_3Na^+$ [M+Na $^+$] 342.0912, found 342.0916.

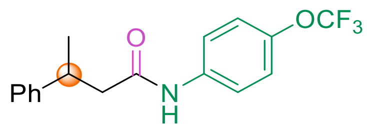

**4w**

**3-phenyl-*N*-(4-(trifluoromethoxy)phenyl)butanamide (4w)**

Chromatography Pentane/EA = 5:1 (v/v), 61.4 mg (95%).

$^{1}$H NMR (300 MHz, $CDCl_3$) δ 7.44 (s, 1H), 7.32 – 7.07 (m, 7H), 7.04 – 6.93 (m, 2H), 3.26 (h, *J* = 7.1 Hz, 1H), 2.61 – 2.42 (m, 2H), 1.26 (d, *J* = 7.0 Hz, 3H).

$^{13}$C NMR (75 MHz, $CDCl_3$) δ 170.4, 145.4, 145.2, 136.2, 128.7, 126.7, 126.7, 121.5, 121.3, 120.4 (q, *J* = 255.0 Hz), 46.4, 37.0, 21.6.

$^{19}F$ NMR (282 MHz, $CDCl_3$) δ -58.14.

HRMS (ES-TOF): m/z calcd. for $C_{17}H_{16}F_3NO_2Na^+$ [M+Na$^+$] 346.1025, found 346.1032.

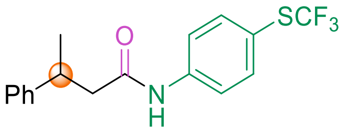

**4x**

**3-phenyl-*N*-(4-((trifluoromethyl)thio)phenyl)butanamide (4x)**

Chromatography Pentane/EA = 5:1 (v/v), 63.1 mg (93%).

$^1H$ NMR (300 MHz, $CDCl_3$) δ 7.51 (s, 1H), 7.46 – 7.39 (m, 2H), 7.36 – 7.29 (m, 2H), 7.25 – 7.08 (m, 5H), 3.26 (h, *J* = 7.1 Hz, 1H), 2.64 – 2.44 (m, 2H), 1.26 (d, *J* = 7.0 Hz, 3H).

$^{13}C$ NMR (75 MHz, $CDCl_3$) δ 170.6, 145.3, 140.2, 137.3, 129.5 (q, *J* = 306.8 Hz), 128.8, 126.7, 126.7, 120.4, 118.7, 46.5, 36.9, 21.7.

$^{19}F$ NMR (282 MHz, $CDCl_3$) δ -43.25.

HRMS (ES-TOF): m/z calcd. for $C_{17}H_{16}F_3NOSNa^+$ [M+Na$^+$] 362.0797, found 362.0802.

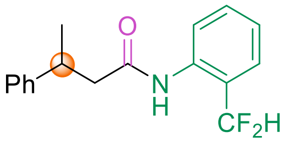

**4y**

***N*-(2-(difluoromethyl)phenyl)-3-phenylbutanamide (4y)**

Chromatography Pentane/EA = 10:1 (v/v), 57.2 mg (99%).

$^1H$ NMR (300 MHz, $CDCl_3$) δ 8.27 – 8.18 (m, 1H), 7.33 (s, 1H), 7.28 – 7.01 (m, 6H), 7.00 – 6.90 (m, 2H), 6.28 (t, *J* = 73.5 Hz, 1H), 3.29 (h, *J* = 7.1 Hz, 1H), 2.68 – 2.46 (m, 2H), 1.28 (d, *J* = 7.0 Hz, 3H).

$^{13}C$ NMR (75 MHz, $CDCl_3$) δ 170.0, 145.4, 140.0, 129.8, 128.6, 126.7, 126.5, 126.1, 124.1, 121.7, 118.5, 116.2 (t, *J* = 259.5 Hz), 46.6, 36.9, 21.8.

$^{19}F$ NMR (282 MHz, $CDCl_3$) δ -79.89 (d, *J* = 73.5 Hz).

HRMS (ES-TOF): m/z calcd. for $C_{17}H_{18}F_2NO^+$ [M+H$^+$] 290.1351, found 290.1355.

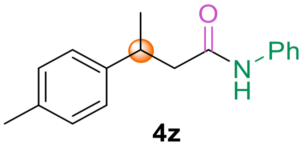

**4z**

***N*-phenyl-3-(*p*-tolyl)butanamide (4z)**

Chromatography Pentane/EA = 10:1 (v/v), 48.5 mg (96%).

$^1H$ NMR (400 MHz, $CDCl_3$) δ 7.39 (s, 1H), 7.32 – 7.24 (m, 2H), 7.21 – 7.10 (m, 2H), 7.07 – 6.91 (m, 5H), 3.23 (h, *J* = 7.1 Hz, 1H), 2.57 – 2.40 (m, 2H), 2.22 (s, 3H), 1.23 (d, *J* = 7.0 Hz, 3H).

$^{13}C$ NMR (101 MHz, $CDCl_3$) δ 170.4, 142.6, 137.7, 136.0, 129.3, 128.8, 126.6, 124.2, 120.1, 46.5, 36.5, 21.7, 20.9.

HRMS (ES-TOF): m/z calcd. for $C_{17}H_{19}NONa^+$ [M+Na$^+$] 276.1359, found 276.1362.

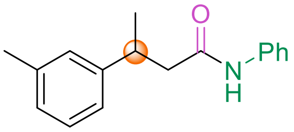

**4aa**

**_N_-phenyl-3-(_m_-tolyl)butanamide (4aa)**

Chromatography Pentane/EA = 10:1 (v/v), 49.0 mg (97%).

$^1$H NMR (400 MHz, $CDCl_3$) δ 7.35 (s, 1H), 7.31 – 7.23 (m, 2H), 7.18 – 7.07 (m, 3H), 7.02 – 6.89 (m, 4H), 3.23 (h, *J* = 7.1 Hz, 1H), 2.58 – 2.41 (m, 2H), 2.21 (d, *J* = 0.7 Hz, 3H), 1.24 (d, *J* = 7.0 Hz, 3H).

$^{13}$C NMR (101 MHz, $CDCl_3$) δ 170.3, 145.6, 138.2, 137.7, 128.8, 128.5, 127.6, 127.3, 124.2, 123.6, 120.1, 46.5, 36.9, 21.6, 21.4.

HRMS (ES-TOF): m/z calcd. for $C_{17}H_{19}NONa^+$ [M+Na $^+$] 276.1359, found 276.1363.

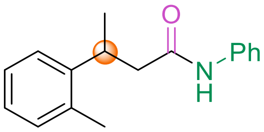

**4ab**

**_N_-phenyl-3-(_o_-tolyl)butanamide (4ab)**

Chromatography Pentane/EA = 10:1 (v/v), 39.9 mg (79%).

$^1$H NMR (300 MHz, $CDCl_3$) δ 7.48 – 6.63 (m, 10H), 3.64 – 3.46 (m, 1H), 2.61 – 2.38 (m, 2H), 2.26 (s, 3H), 1.22 (d, *J* = 6.9 Hz, 3H).

$^{13}$C NMR (75 MHz, $CDCl_3$) δ 170.3, 143.8, 137.7, 135.6, 130.6, 128.8, 126.4, 126.2, 125.0, 124.2, 120.0, 45.8, 31.9, 21.3, 19.4.

HRMS (ES-TOF): m/z calcd. for $C_{17}H_{19}NONa^+$ [M+Na $^+$] 276.1359, found 276.1364.

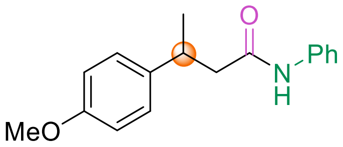

**4ac**

**3-(4-methoxyphenyl)-_N_-phenylbutanamide (4ac)[3]**

Chromatography Pentane/EA = 5:1 (v/v), 45.0 mg (84%).

$^1$H NMR (300 MHz, $CDCl_3$) δ 7.35 (s, 1H), 7.31 – 7.25 (m, 2H), 7.20 – 7.12 (m, 2H), 7.09 – 7.03 (m, 2H), 7.01 – 6.94 (m, 1H), 6.79 – 6.70 (m, 2H), 3.68 (s, 3H), 3.23 (h, *J* = 7.1 Hz, 1H), 2.57 – 2.38 (m, 2H), 1.23 (d, *J* = 7.0 Hz, 3H).

$^{13}$C NMR (75 MHz, $CDCl_3$) δ 170.3, 158.1, 137.6, 128.8, 127.7, 124.2, 120.0, 114.0, 55.2, 46.7, 36.1, 21.8.

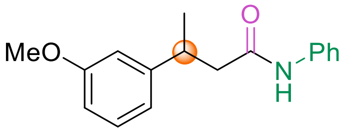

**4ad**

**3-(3-methoxyphenyl)-*N*-phenylbutanamide (4ad)**

Chromatography Pentane/EA = 5:1 (v/v), 52.1 mg (97%).

$^{1}$H NMR (300 MHz, $CDCl_3$) δ 7.41 (s, 1H), 7.33 – 7.23 (m, 2H), 7.21 – 7.05 (m, 3H), 7.02 – 6.91 (m, 1H), 6.78 – 6.61 (m, 3H), 3.65 (s, 3H), 3.24 (h, *J* = 7.1 Hz, 1H), 2.59 – 2.38 (m, 2H), 1.24 (d, *J* = 7.0 Hz, 3H).

$^{13}$C NMR (75 MHz, $CDCl_3$) δ 170.2, 159.7, 147.3, 137.7, 129.6, 128.8, 124.2, 120.1, 119.0, 112.7, 111.6, 55.1, 46.4, 37.0, 21.5.

HRMS (ES-TOF): m/z calcd. for $C_{17}H_{19}NO_2Na^+$ [M+Na $^+$] 292.1308, found 292.1316.

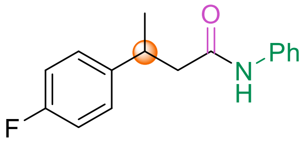

**4ae**

**3-(4-fluorophenyl)-*N*-phenylbutanamide (4ae)[4]**

Chromatography Pentane/EA = 10:1 (v/v), 42.8 mg (83%).

$^{1}$H NMR (300 MHz, $CDCl_3$) δ 7.75 – 6.55 (m, 3H), 3.29 (h, *J* = 7.1 Hz, 0H), 2.48 (d, *J* = 7.1 Hz, 0H), 1.25 (d, *J* = 7.0 Hz, 1H).

$^{13}$C NMR (75 MHz, $CDCl_3$) δ 169.9, 161.4 (d, *J* = 244.5 Hz), 141.3 (d, *J* = 3.1 Hz), 137.6, 128.9, 128.2 (d, *J* = 7.8 Hz), 124.4, 120.0, 115.4 (d, *J* = 21.0 Hz), 46.7, 36.2, 21.7.

$^{19}$F NMR (282 MHz, $CDCl_3$) δ -116.35 – -116.63 (m).

HRMS (ES-TOF): m/z calcd. for $C_{16}H_{15}FNONa^+$ [M+Na $^+$] 314.0718, found 314.0721.

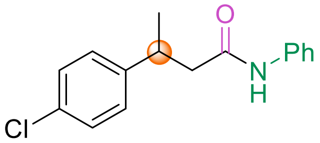

**4af**

**3-(4-chlorophenyl)-*N*-phenylbutanamide (4af)**

Chromatography Pentane/EA = 10:1 (v/v), 48.8 mg (89%).

$^{1}$H NMR (400 MHz, $CDCl_3$) δ 7.34 – 7.28 (m, 2H), 7.26 – 7.14 (m, 5H), 7.12 – 7.04 (m, 2H), 7.03 – 6.96 (m, 1H), 3.28 (h, *J* = 7.1 Hz, 1H), 2.54 – 2.40 (m, 2H), 1.24 (d, *J* = 7.0 Hz, 3H).

$^{13}$C NMR (101 MHz, $CDCl_3$) δ 169.7, 144.1, 137.5, 132.1, 128.9, 128.7, 128.1, 124.4, 120.0, 46.4, 36.3, 21.5.

HRMS (ES-TOF): m/z calcd. for $C_{16}H_{16}ClNONa^+$ [M+Na $^+$] 296.0812, found 296.0817.

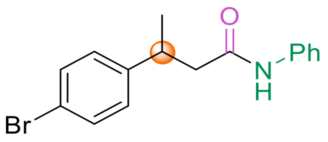

**4ag**

**3-(4-bromophenyl)-*N*-phenylbutanamide (4ag)**

Chromatography Pentane/EA = 10:1 (v/v), 58.3 mg (92%).

$^{1}$H NMR (300 MHz, $CDCl_3$) δ 7.44 – 7.13 (m, 7H), 7.08 – 6.92 (m, 3H), 3.26 (h, *J* = 7.1 Hz, 1H), 2.55 – 2.37 (m, 2H), 1.22 (d, *J* = 7.0 Hz, 3H).

$^{13}$C NMR (75 MHz, $CDCl_3$) δ 169.8, 144.6, 137.5, 131.6, 128.9, 128.5, 124.4, 120.1, 46.2, 36.3, 21.4.

HRMS (ES-TOF): m/z calcd. for $C_{16}H_{16}BrNONa^+$ [M+Na$^+$] 340.0307, found 340.0310.

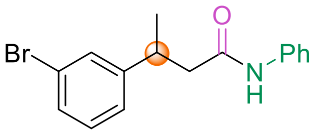

**4ah**

**3-(3-bromophenyl)-*N*-phenylbutanamide (4ah)**

Chromatography Pentane/EA = 10:1 (v/v), 48.6 mg (77%).

$^{1}$H NMR (300 MHz, $CDCl_3$) δ 7.47 (s, 1H), 7.37 – 7.12 (m, 6H), 7.11 – 6.93 (m, 3H), 3.25 (h, *J* = 7.1 Hz, 1H), 2.56 – 2.36 (m, 2H), 1.22 (d, *J* = 7.0 Hz, 3H).

$^{13}$C NMR (75 MHz, $CDCl_3$) δ 169.8, 148.1, 137.5, 130.2, 129.7, 129.6, 128.9, 125.7, 124.4, 122.6, 120.2, 46.0, 36.5, 21.4.

HRMS (ES-TOF): m/z calcd. for $C_{16}H_{16}BrNONa^+$ [M+Na$^+$] 340.0307, found 340.0313.

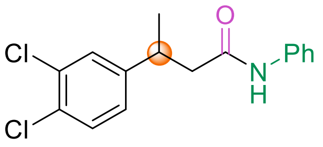

**4ai**

**phenylbutanamide (4ai)**

Chromatography Pentane/EA = 10:1 (v/v), 49.2 mg (80%).

$^{1}$H NMR (300 MHz, $CDCl_3$) δ 7.55 (s, 1H), 7.38 – 7.29 (m, 2H), 7.27 – 7.11 (m, 4H), 7.07 – 6.89 (m, 2H), 3.25 (h, *J* = 7.1 Hz, 1H), 2.53 – 2.34 (m, 2H), 1.20 (d, *J* = 7.0 Hz, 3H).

$^{13}$C NMR (75 MHz, $CDCl_3$) δ 169.6, 146.0, 137.4, 132.4, 130.4, 130.2, 128.9, 128.6, 126.4, 124.6, 120.2, 45.8, 36.0, 21.3.

HRMS (ES-TOF): m/z calcd. for $C_{16}H_{15}Cl_2NONa^+$ [M+Na$^+$] 330.0423, found 330.0426.

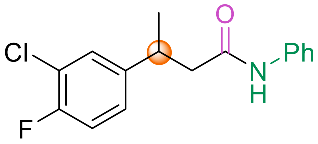

**4aj**

**3-(3-chloro-4-fluorophenyl)-*N*-phenylbutanamide (4aj)**

Chromatography Pentane/EA = 10:1 (v/v), 50.8 mg (87%).

$^1$H NMR (300 MHz, $CDCl_3$) δ 7.52 (s, 1H), 7.38 – 7.27 (m, 2H), 7.25 – 7.10 (m, 3H), 7.06 – 6.87 (m, 3H), 3.26 (h, *J* = 7.1 Hz, 1H), 2.54 – 2.35 (m, 2H), 1.21 (d, *J* = 7.0 Hz, 3H).

$^{13}$C NMR (75 MHz, $CDCl_3$) δ 169.7, 156.6 (d, *J* = 247.3 Hz), 142.7 (d, *J* = 4.0 Hz), 137.5, 128.9, 128.7, 126.6 (d, *J* = 6.9 Hz), 124.5, 120.7 (d, *J* = 17.6 Hz), 120.2, 116.5 (d, *J* = 20.8 Hz), 46.1, 35.9, 21.5.

$^{19}$F NMR (282 MHz, $CDCl_3$) δ -118.67 – -118.87 (m).

HRMS (ES-TOF): m/z calcd. for $C_{16}H_{16}ClFNO^+$ [M+H $^+$] 292.0899, found 292.0901.

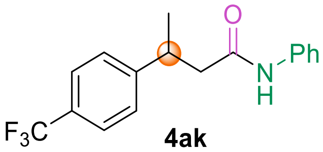

**4ak**

***N*-phenyl-3-(4-(trifluoromethyl)phenyl)butanamide (4ak)**

Chromatography Pentane/EA = 10:1 (v/v), 25.4 mg (41%).

$^1$H NMR (300 MHz, $CDCl_3$) δ 7.60 – 7.14 (m, 9H), 7.07 – 6.95 (m, 1H), 3.39 (h, *J* = 7.1 Hz, 1H), 2.52 (dd, *J* = 7.4, 2.3 Hz, 2H), 1.28 (d, *J* = 7.0 Hz, 3H).

$^{13}$C NMR (75 MHz, $CDCl_3$) δ 169.5, 149.8, 137.5, 129.0, 127.2, 125.5 (q, *J* = 3.5 Hz), 124.5, 124.2 (q, *J* = 270.0 Hz), 120.0, 46.1, 36.6, 21.3.

$^{19}$F NMR (282 MHz, $CDCl_3$) δ -62.37.

HRMS (ES-TOF): m/z calcd. for $C_{17}H_{16}F_3NONa^+$ [M+Na $^+$] 330.1076, found 330.1084.

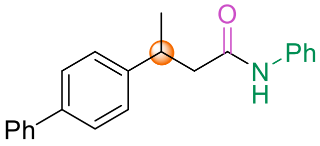

**4al**

**3-([1,1'-biphenyl]-4-yl)-*N*-phenylbutanamide (4al)**

Chromatography Pentane/EA = 10:1 (v/v), 55.3 mg (88%).

$^1$H NMR (300 MHz, $CDCl_3$) δ 7.53 – 7.40 (m, 4H), 7.39 – 7.12 (m, 10H), 7.04 – 6.92 (m, 1H), 3.34 (h, *J* = 7.1 Hz, 1H), 2.64 – 2.44 (m, 2H), 1.31 (d, *J* = 6.9 Hz, 3H).

$^{13}$C NMR (75 MHz, $CDCl_3$) δ 170.1, 144.7, 140.8, 139.5, 137.6, 128.9, 128.7, 127.4, 127.2, 127.1, 126.9, 124.3, 120.0, 46.6, 36.6, 21.6.

HRMS (ES-TOF): m/z calcd. for $C_{22}H_{21}NONa^+$ [M+Na $^+$] 338.1515, found 338.1518.

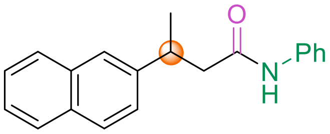

**4am**

**3-(naphthalen-2-yl)-*N*-phenylbutanamide (4am)**

Chromatography Pentane/EA = 10:1 (v/v), 55.5 mg (96%).

$^{1}$H NMR (400 MHz, $CDCl_3$) δ 7.73 – 7.59 (m, 3H), 7.54 (d, *J* = 1.7 Hz, 1H), 7.41 (s, 1H), 7.38 – 7.29 (m, 2H), 7.27 – 7.21 (m, 3H), 7.15 – 7.07 (m, 2H), 6.94 (t, *J* = 7.3 Hz, 1H), 3.43 (h, *J* = 7.1 Hz, 1H), 2.85 – 2.34 (m, 2H), 1.31 (d, *J* = 7.0 Hz, 3H).

$^{13}$C NMR (101 MHz, $CDCl_3$) δ 170.2, 143.0, 137.6, 133.5, 132.3, 128.8, 128.3, 127.6, 127.5, 126.0, 125.4, 125.3, 125.0, 124.3, 120.1, 46.3, 37.0, 21.5.

HRMS (ES-TOF): m/z calcd. for $C_{20}H_{19}NONa^+$ [M+Na$^+$] 312.1359, found 312.1356.

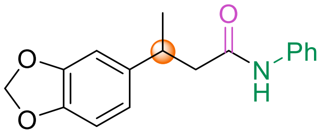

**4an**

**3-(benzo[*d*][1,3]dioxol-5-yl)-*N*-phenylbutanamide (4an)**

Chromatography Pentane/EA = 5:1 (v/v), 44.3 mg (78%).

$^{1}$H NMR (300 MHz, $CDCl_3$) δ 7.38 (s, 1H), 7.35 – 7.27 (m, 2H), 7.24 – 7.11 (m, 2H), 7.04 – 6.93 (m, 1H), 6.69 – 6.49 (m, 3H), 5.87 – 5.74 (m, 2H), 3.21 (h, *J* = 7.1 Hz, 1H), 2.55 – 2.36 (m, 2H), 1.21 (d, *J* = 7.0 Hz, 3H).

$^{13}$C NMR (75 MHz, $CDCl_3$) δ 170.1, 147.7, 146.0, 139.6, 137.7, 128.8, 124.3, 120.1, 119.7, 108.3, 107.0, 100.8, 46.6, 36.7, 21.9.

HRMS (ES-TOF): m/z calcd. for $C_{17}H_{17}NO_3Na^+$ [M+Na$^+$] 306.1100, found 306.1102.

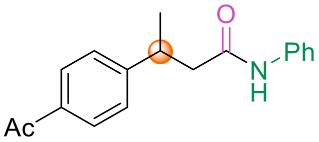

**4ao**

**3-(4-acetylphenyl)-*N*-phenylbutanamide (4ao)**

Chromatography Pentane/EA = 3:1 (v/v), 55.1 mg (98%).

$^{1}$H NMR (300 MHz, $CDCl_3$) δ 7.95 (s, 1H), 7.81 – 7.70 (m, 2H), 7.44 – 7.31 (m, 2H), 7.25 – 7.10 (m, 4H), 7.04 – 6.89 (m, 1H), 3.37 (h, *J* = 7.1 Hz, 1H), 2.54 (dd, *J* = 7.4, 4.3 Hz, 2H), 2.47 (s, 3H), 1.24 (d, *J* = 7.0 Hz, 3H).

$^{13}$C NMR (75 MHz, $CDCl_3$) δ 198.1, 169.8, 151.6, 137.8, 135.3, 128.8, 128.7, 127.0, 124.2, 120.0, 45.6, 36.7, 26.5, 21.3.

HRMS (ES-TOF): m/z calcd. for $C_{18}H_{19}NO_2Na^+$ [M+Na$^+$] 304.1308, found 304.1313.

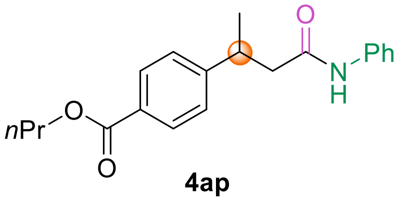

**4ap**

**propyl 4-(4-oxo-4-(phenylamino)butan-2-yl)benzoate (4ap)**

Chromatography Pentane/EA = 5:1 (v/v), 55.0 mg (85%).

$^{1}$H NMR (300 MHz, $CDCl_3$) δ 7.91 – 7.81 (m, 2H), 7.78 – 7.72 (m, 1H), 7.39 – 7.27 (m, 2H), 7.23 – 7.09 (m, 4H), 7.04 – 6.90 (m, 1H), 4.17 (t, *J* = 6.6 Hz, 2H), 3.36 (h, *J* = 7.1 Hz, 1H), 2.62 – 2.42 (m, 2H), 1.78 – 1.60 (m, 2H), 1.24 (d, *J* = 7.0 Hz, 3H), 0.92 (t, *J* = 7.4 Hz, 3H).

$^{13}$C NMR (75 MHz, $CDCl_3$) δ 169.8, 166.7, 151.1, 137.7, 129.9, 128.8, 128.6, 126.8, 124.3, 120.0, 66.5, 45.8, 36.7, 22.0, 21.3, 10.4.

HRMS (ES-TOF): m/z calcd. for $C_{20}H_{23}NO_3Na^+$ [M+Na$^+$] 348.1570, found 348.1569.

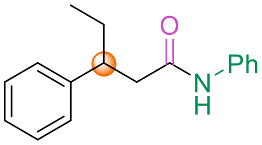

**4aq**

***N*,3-diphenylpentanamide (4aq)**

Chromatography Pentane/EA = 10:1 (v/v), 32.2 mg (64%).

$^{1}$H NMR (300 MHz, $CDCl_3$) δ 7.28 – 7.04 (m, 3H), 7.02 – 6.86 (m, 0H), 3.06 – 2.89 (m, 0H), 2.67 – 2.39 (m, 1H), 1.80 – 1.46 (m, 1H), 0.71 (t, *J* = 7.3 Hz, 1H).

$^{13}$C NMR (75 MHz, $CDCl_3$) δ 170.3, 143.8, 137.6, 128.8, 128.6, 127.5, 126.6, 124.2, 120.0, 45.2, 44.5, 29.0, 11.9.

HRMS (ES-TOF): m/z calcd. for $C_{17}H_{19}NONa^+$ [M+Na$^+$] 276.1359, found 276.1363.

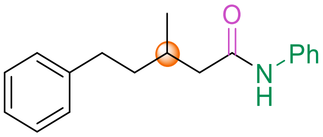

**4ar**

**3-methyl-*N*,5-diphenylpentanamide (4ar)**

Chromatography Pentane/EA = 10:1 (v/v), 43.1 mg (81%).

$^{1}$H NMR (300 MHz, $CDCl_3$) δ 7.48 – 7.36 (m, 2H), 7.29 – 6.87 (m, 9H), 2.70 – 2.44 (m, 2H), 2.39 – 2.24 (m, 1H), 2.16 – 1.93 (m, 2H), 1.78 – 1.56 (m, 1H), 1.55 – 1.37 (m, 1H), 0.98 (d, *J* = 6.3 Hz, 3H).

$^{13}$C NMR (75 MHz, $CDCl_3$) δ 170.8, 142.3, 137.8, 128.9, 128.3, 128.3, 125.7, 124.2, 119.9, 45.3, 38.5, 33.3, 30.6, 19.6.

HRMS (ES-TOF): m/z calcd. for $C_{18}H_{21}NONa^+$ [M+Na$^+$] 290.1515, found 290.1518.

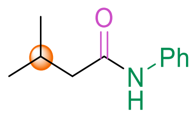


**4as**

**3-methyl-*N*-phenylbutanamide (4as)[5]**

Chromatography Pentane/EA = 10:1 (v/v), 29.1 mg (82%).

$^{1}$H NMR (300 MHz, $CDCl_3$) δ 7.67 (s, 1H), 7.52 – 7.40 (m, 2H), 7.27 – 7.14 (m, 2H), 7.06 – 6.95 (m, 1H), 2.20 – 2.04 (m, 3H), 0.99 – 0.83 (m, 6H).

$^{13}$C NMR (75 MHz, $CDCl_3$) δ 171.2, 137.9, 128.8, 124.2, 120.0, 46.9, 26.2, 22.4.

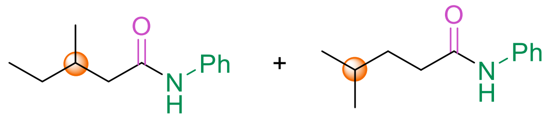


**4at+4au**

**3-methyl-*N*-phenylpentanamide (4at), 4-methyl-*N*-phenylpentanamide (4au)**

Chromatography Pentane/EA = 10:1 (v/v), 22.9 mg (60%).

$^{1}$H NMR (400 MHz, $CDCl_3$) δ 7.51 – 7.20 (m, 5.06H), 7.06 – 6.98 (m, 1H), 2.29 (dd, *J* = 13.9, 6.2 Hz, 1.29H), 2.04 (dd, *J* = 13.9, 8.2 Hz, 0.84H), 1.97 – 1.87 (m, 0.82H), 1.63 – 1.51 (m, 0.66H), 1.44 – 1.29 (m, 0.86H), 1.29 – 1.10 (m, 1.23H), 0.91 (d, *J* = 6.6 Hz, 2.51H), 0.85 (d, *J* = 7.2 Hz, 3.59H).

$^{13}$C NMR (101 MHz, $CDCl_3$) δ 171.7, 171.1, 138.0, 137.9, 128.9, 124.2, 124.1, 119.9, 119.8, 45.1, 35.8, 34.4, 32.4, 29.4, 27.8, 22.3, 19.2, 11.3.

HRMS (ES-TOF): m/z calcd. for $C_{12}H_{18}NO^{+}$ [M+H $^{+}$] 192.1383, found 192.1388.

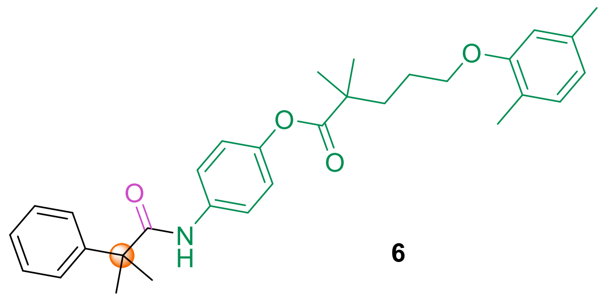


**6**

**4-(2-methyl-2-phenylpropanamido)phenyl 5-(2,5-dimethylphenoxy)-2,2-dimethylpentanoate (6)**

Chromatography Pentane/EA = 8:1 (v/v), 78.5 mg (81%).

$^{1}$H NMR (300 MHz, $CDCl_3$) δ 7.53 – 7.30 (m, 7H), 7.04 (dd, *J* = 7.4, 0.9 Hz, 1H), 7.00 – 6.88 (m, 3H), 6.76 – 6.63 (m, 2H), 4.09 – 3.95 (m, 2H), 2.35 (s, 3H), 2.22 (s, 3H), 1.91 (d, *J* = 2.9 Hz, 4H), 1.71 (s, 6H), 1.40 (s, 6H).

$^{13}$C NMR (75 MHz, $CDCl_3$) δ 176.4, 175.5, 156.8, 147.0, 144.5, 136.4, 135.5, 130.3, 128.9, 127.3, 126.4, 123.5, 121.7, 120.7, 120.5, 111.9, 67.7, 47.9, 42.3, 37.0, 26.9, 25.2, 25.1, 21.3, 15.7.

HRMS (ES-TOF): m/z calcd. for $C_{31}H_{38}NO_4^{+}$ [M+H $^{+}$] 488.2795, found 488.2799.

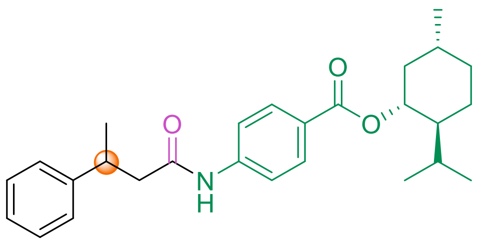

**8**

**(1*R*,2*S*,5*R*)-2-isopropyl-5-methylcyclohexyl 4-(3-phenylbutanamido)benzoate (8)**

Chromatography Pentane/EA = 6:1 (v/v), 74.8 mg (89%).

$^{1}$H NMR (300 MHz, $CDCl_3$) δ 7.91 – 7.81 (m, 2H), 7.65 (d, *J* = 3.8 Hz, 1H), 7.44 – 7.33 (m, 2H), 7.26 – 7.16 (m, 2H), 7.15 – 7.05 (m, 3H), 4.88 – 4.74 (m, 1H), 3.37 – 3.19 (m, 1H), 2.65 – 2.44 (m, 2H), 2.07 – 1.93 (m, 1H), 1.92 – 1.76 (m, 1H), 1.70 – 1.54 (m, 2H), 1.53 – 1.35 (m, 2H), 1.26 (d, *J* = 7.0 Hz, 3H), 1.12 – 0.92 (m, 2H), 0.90 – 0.75 (m, 7H), 0.68 (d, *J* = 6.9 Hz, 3H).

$^{13}$C NMR (75 MHz, $CDCl_3$) δ 170.4, 165.7, 145.4, 141.9, 130.6, 128.7, 126.7, 126.6, 126.0, 118.9, 74.8, 47.2, 46.6, 40.9, 36.9, 36.9, 34.2, 31.4, 26.5, 23.6, 22.0, 21.6, 20.7, 16.5.

HRMS (ES-TOF): m/z calcd. for $C_{27}H_{35}NO_3Na^+$ [M+Na$^+$] 444.2509, found 444.2506.

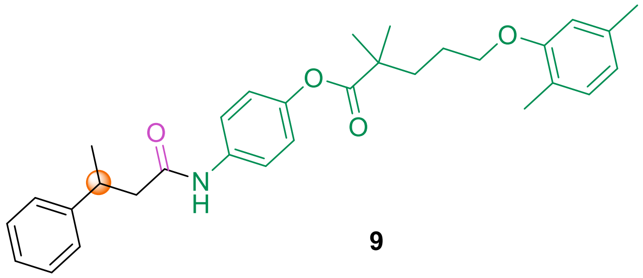

**9**

**4-(3-phenylbutanamido)phenyl 5-(2,5-dimethylphenoxy)-2,2-dimethylpentanoate (9)**

Chromatography Pentane/EA = 5:1 (v/v), 89.8 mg (92%).

$^{1}$H NMR (300 MHz, $CDCl_3$) δ 7.52 (s, 1H), 7.41 – 7.20 (m, 7H), 7.06 (dd, *J* = 7.4, 0.9 Hz, 1H), 6.98 – 6.87 (m, 2H), 6.77 – 6.65 (m, 2H), 4.10 – 3.98 (m, 2H), 3.40 (q, *J* = 7.1 Hz, 1H), 2.69 – 2.49 (m, 2H), 2.36 (d, *J* = 0.9 Hz, 3H), 2.23 (s, 3H), 1.92 (t, *J* = 2.1 Hz, 4H), 1.57 – 1.11 (m, 9H).

$^{13}$C NMR (75 MHz, $CDCl_3$) δ 176.8, 170.1, 156.8, 146.9, 145.6, 136.4, 135.3, 130.3, 128.6, 126.7, 126.5, 123.5, 121.6, 121.0, 120.7, 111.9, 67.6, 46.3, 42.3, 37.0, 36.8, 25.2, 25.1, 21.5, 21.3, 15.7.

HRMS (ES-TOF): m/z calcd. for $C_{31}H_{37}NO_4Na^+$ [M+Na$^+$] 510.2614, found 510.2618.

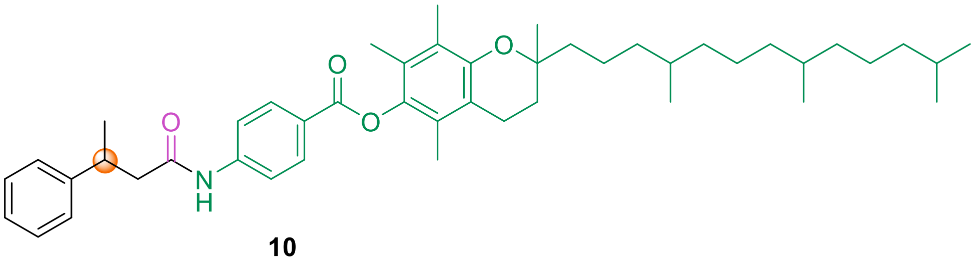

**10**

**2,5,7,8-tetramethyl-2-(4,8,12-trimethyltridecyl)chroman-6-yl 4-(3-phenylbutanamido)benzoate (10)**

Chromatography Pentane/EA = 6:1 (v/v), 122.4 mg (88%).

$^{1}$H NMR (300 MHz, $CDCl_3$) δ 8.24 – 8.13 (m, 2H), 8.01 (s, 1H), 7.57 (d, *J* = 8.7 Hz, 2H), 7.39 – 7.30 (m, 2H), 7.29 – 7.21 (m, 3H), 3.59 – 3.27 (m, 1H), 2.73 – 2.53 (m, 4H), 2.30 – 2.02 (m, 9H), 1.94 – 1.73 (m, 2H), 1.69 – 1.09 (m, 27H), 1.00 – 0.88 (m, 12H).

$^{13}$C NMR (75 MHz, $CDCl_3$) δ 170.6, 165.1, 149.4, 145.4, 142.7, 140.5, 131.2, 128.6, 126.8, 126.7, 126.6, 125.0, 124.4, 123.1, 119.1, 117.4, 75.0, 46.3, 39.3, 37.5, 37.4, 37.3, 37.3, 37.2, 36.8, 32.7, 27.9, 24.7, 24.4, 22.7, 22.6, 21.6, 21.0, 20.5, 19.7, 19.6, 19.6, 19.6, 19.5, 13.0, 12.1, 11.8.

HRMS (ES-TOF): m/z calcd. for $C_{46}H_{66}NO_4^+$ [M+H $^+$] 696.4986, found 696.4989.

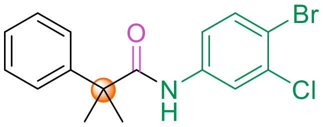

**12**

***N*-(4-bromo-3-chlorophenyl)-2-methyl-2-phenylpropanamide (12)**

Chromatography Pentane/EA = 20:1 (v/v), 49.5 mg (71%).

$^{1}$H NMR (300 MHz, $CDCl_3$) δ 7.53 (d, *J* = 2.5 Hz, 1H), 7.40 – 7.22 (m, 6H), 7.02 (dd, *J* = 8.7, 2.5 Hz, 1H), 6.72 (s, 1H), 1.58 (s, 6H).

$^{13}$C NMR (75 MHz, $CDCl_3$) δ 175.7, 144.0, 138.1, 134.6, 133.5, 129.1, 127.6, 126.4, 121.2, 119.0, 116.5, 48.2, 26.9.

HRMS (ES-TOF): m/z calcd. for $C_{16}H_{15}BrClNONa^+$ [M+Na $^+$] 352.0099, found 352.0108.

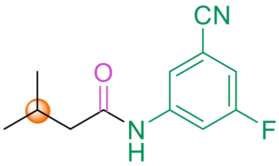

**15**

***N*-(3-cyano-5-fluorophenyl)-3-methylbutanamide (15)**

Chromatography Pentane/EA = 6:1 (v/v), 29.1 mg (66%).

$^{1}$H NMR (300 MHz, $CDCl_3$) δ 7.83 – 7.73 (m, 1H), 7.71 (s, 1H), 7.61 – 7.53 (m, 1H), 7.12 – 7.03 (m, 1H), 2.31 – 2.08 (m, 3H), 1.01 (d, *J* = 6.5 Hz, 6H).

$^{13}$C NMR (75 MHz, $CDCl_3$) δ 171.4, 162.5 (d, *J* = 249.0 Hz), 140.6 (d, *J* = 11.4 Hz), 118.5 (d, *J* = 3.3 Hz), 117.4 (d, *J* = 3.8 Hz), 114.2 (d, *J* = 25.1 Hz), 113.7 (d, *J* = 11.1 Hz), 111.7 (d, *J* = 26.2 Hz), 46.8, 26.2, 22.4.

$^{19}$F NMR (282 MHz, $CDCl_3$) δ -107.74 – -107.93 (m).

HRMS (ES-TOF): m/z calcd. for $C_{12}H_{13}FN_2ONa^+$ [M+Na$^+$] 221.1085, found 221.1083.

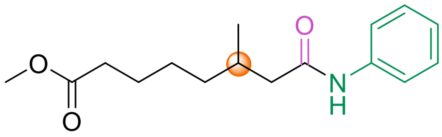


**17**

**methyl 6-methyl-8-oxo-8-(phenylamino)octanoate (17)**

Chromatography Pentane/EA = 6:1 (v/v), 33.4 mg (60%).

$^{1}$H NMR (300 MHz, $CDCl_3$) δ 7.53 – 7.40 (m, 1H), 7.28 – 7.16 (m, 1H), 7.07 – 6.96 (m, 0H), 3.59 (s, 1H), 2.36 – 2.18 (m, 1H), 2.11 – 1.94 (m, 1H), 1.62 – 1.46 (m, 1H), 1.39 – 1.08 (m, 1H), 0.91 (d, *J* = 6.2 Hz, 1H).

$^{13}$C NMR (75 MHz, $CDCl_3$) δ 174.25, 170.97, 137.98, 128.89, 124.12, 119.84, 51.46, 45.21, 36.22, 33.90, 30.63, 26.32, 24.87, 19.63.

HRMS (ES-TOF): m/z calcd. for $C_{16}H_{23}NO_3Na^+$ [M+Na$^+$] 300.1570, found 300.1578.

## 5. Intermediate verification experiments

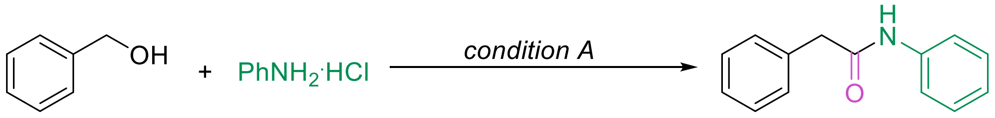


**18** **2a** **19,** nd

A 4 mL screw-cap vial was charged with $[Pd(cinnamyl)Cl]_2$ (2.6 mg, 2.5 mol%), $(4\text{-}CF_3C_6H_4)_3P$ (16.1 mg, 12 mol%), $ArNH_2$·HCl (0.2 mmol, 1.0 equiv.), alcohol **18** (0.3 mmol, 1.5 equiv.) and an oven-dried stirring bar. The vial was closed with a Teflon septum and cap and connected to the atmosphere via a needle. After DCE (1.0 mL) was added with a syringe, the vial was moved to an alloy plate and put into a Parr 4560 series autoclave (300 mL). At room temperature, the autoclave was flushed three times with $N_2$ and CO, respectively, and finally charged with 40 atm CO. The autoclave was placed on a heating plate equipped with a magnetic stirrer and an aluminum block. The reaction mixture was heated to 110 °C for 30 h. After reaction, cooling to room temperature. Results were observed by TLC and GC-MS.

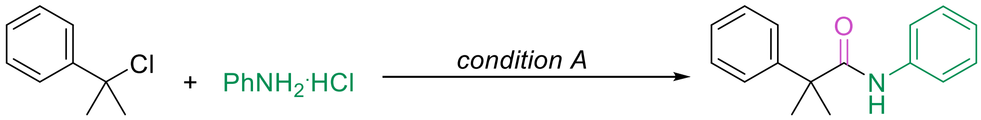


**20** **2a** **3a,** nd

A 4 mL screw-cap vial was charged with $[Pd(cinnamyl)Cl]_2$ (2.6 mg, 2.5 mol%), $(4\text{-}CF_3C_6H_4)_3P$ (16.1 mg, 12 mol%), $ArNH_2$·HCl (0.2 mmol, 1.0 equiv.), alkyl chloride **20** (0.3 mmol, 1.5 equiv.) and an oven-dried stirring bar. The vial was closed with a Teflon septum and cap and connected to the atmosphere via a needle. After DCE (1.0 mL) was added with a syringe, the vial was moved to an alloy plate and put into a Parr 4560 series autoclave (300 mLAt room temperature, the autoclave was flushed three times with $N_2$ and CO, respectively, and finally charged with 40 atm CO. The autoclave was placed on a heating plate equipped with a magnetic stirrer and an aluminum block. The reaction mixture was heated to 110 °C for 30 h. After reaction, cooling to room temperature. Results were observed by TLC and GC-MS.

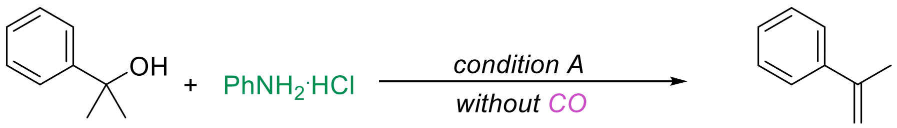


**1a** **2a** **21, detected by GC-MS**

A 5 mL vial was charged with $[Pd(cinnamyl)Cl]_2$ (2.6 mg, 2.5 mol%), $(4\text{-}CF_3C_6H_4)_3P$ (16.1 mg, 12 mol%), $ArNH_2$·HCl (0.2 mmol, 1.0 equiv.), alcohol **1a** (0.3 mmol, 1.5 equiv.) and an oven-dried stirring bar. The vial was closed with a Teflon septum and cap and connected to the atmosphere via a needle. After DCE (1.0 mL) was added with a syringe. The reaction mixture was heated to 110 °C for 18 h. After reaction, cooling to room temperature. Results were observed by TLC and GC-MS.

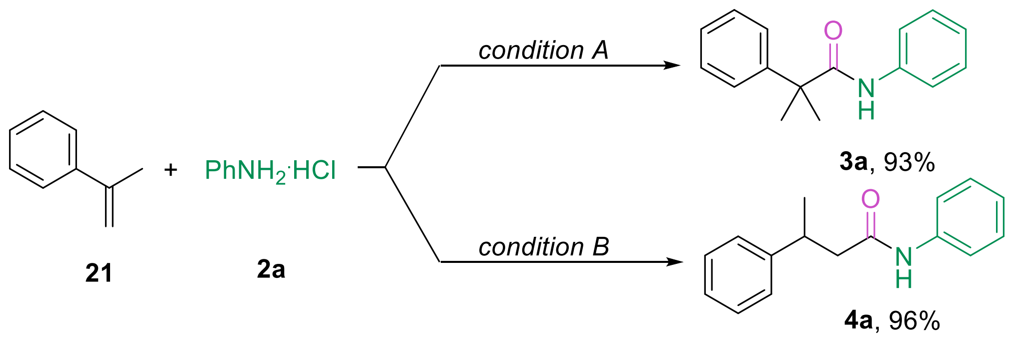

A 4 mL screw-cap vial was charged with Pd cat., ligand, $PhNH_2{\cdot}HCl$, cyclobutene **26** and an oven-dried stirring bar. The vial was closed with a Teflon septum and cap and connected to the atmosphere via a needle. After DCE (1.0 or 2.0 mL) was added with a syringe, the vial was moved to an alloy plate and put into a Parr 4560 series autoclave (300 mL). At room temperature, the autoclave was flushed three times with $N_2$ and CO, respectively, and finally charged with 40 or 20 atm CO. The autoclave was placed on a heating plate equipped with a magnetic stirrer and an aluminum block. After reaction, cooling to room temperature. The crude product was purified by silica gel chromatography (pentane/EA) to afford the corresponding product **3a** (condition A) or **4a** (condition B).

## 6. Reference


[1] H. Y. Yang, Y. H. Yao, M. Chen, Z. H. Ren, Z. H. Guan, *J. Am. Chem. Soc.* **2021**, *143*, 7298-7305.

[2] P. Yuan, J. Chen, J. Zhao, Y. Huang. *Angew. Chem. Int. Ed.* **2018**, 57, 8503-8507.

[3] Z. Wu, J. D. Laffoon, T. T. Nguyen, J. D. Mcalpin, K. L. Hull. *Angew. Chem. Int. Ed.* **2017**, 56, 1371-1375.

[4] G. A. Molander, S. R. Wisniewski, M. Hosseini-Sarvari. *Adv. Synth. Catal.* **2013**, 355, 3037-3057.

[5] P. Cooper, G. E. M. Crisenza, L. J. Feron, J. F. Bower. *Angew. Chem. Int. Ed.* **2018**, 57, 14198-14202.

# 7. NMR spectra of products

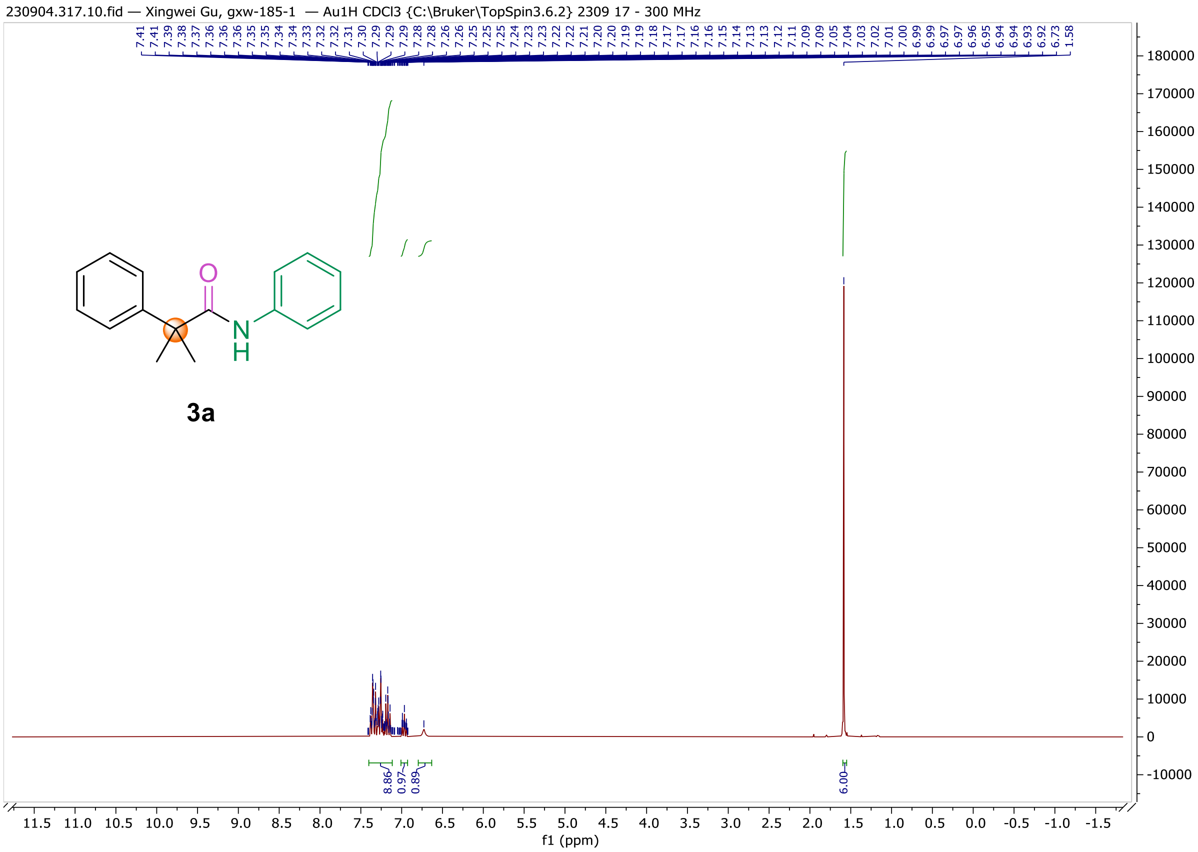


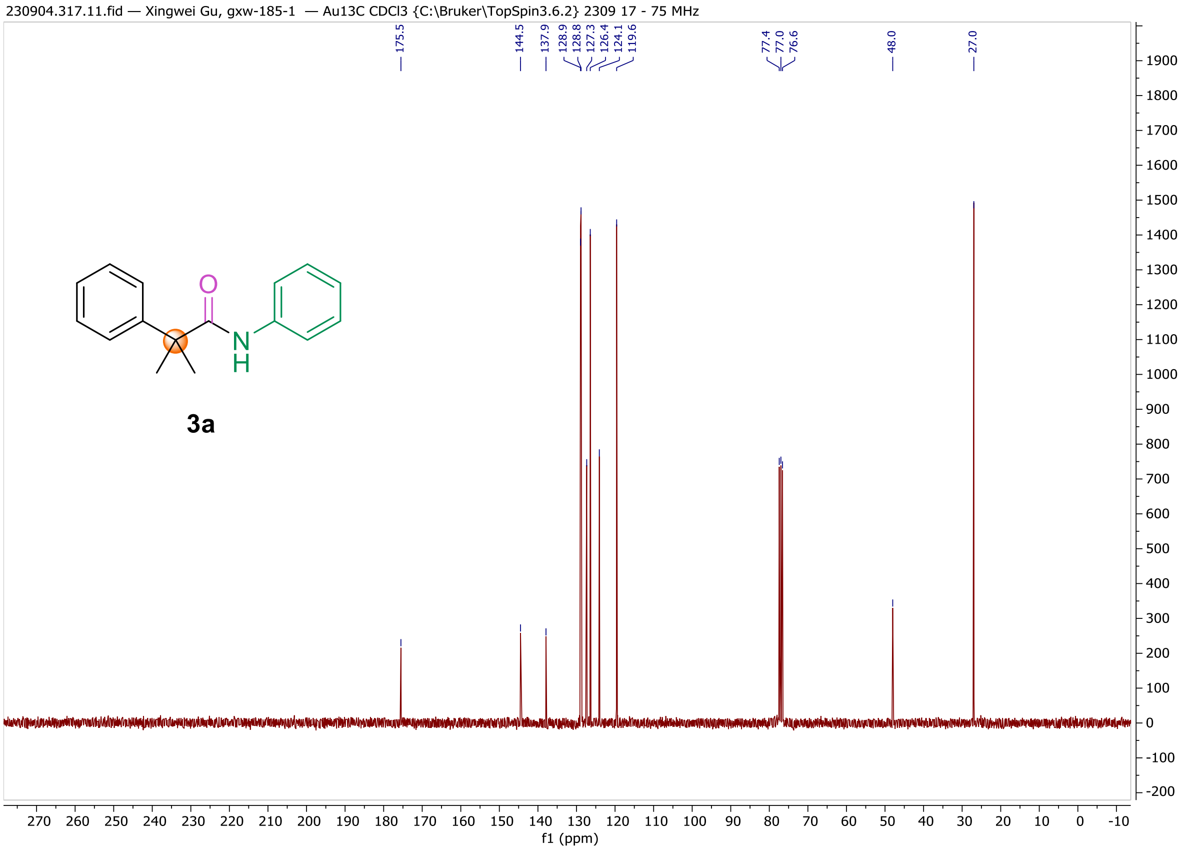

231010.f304.10.fid — Xingwei Gu gxw-195-2 — Au1H CDCl3 {C:\Bruker\TopSpin3.6.2} 2310 4 - 300 MHz

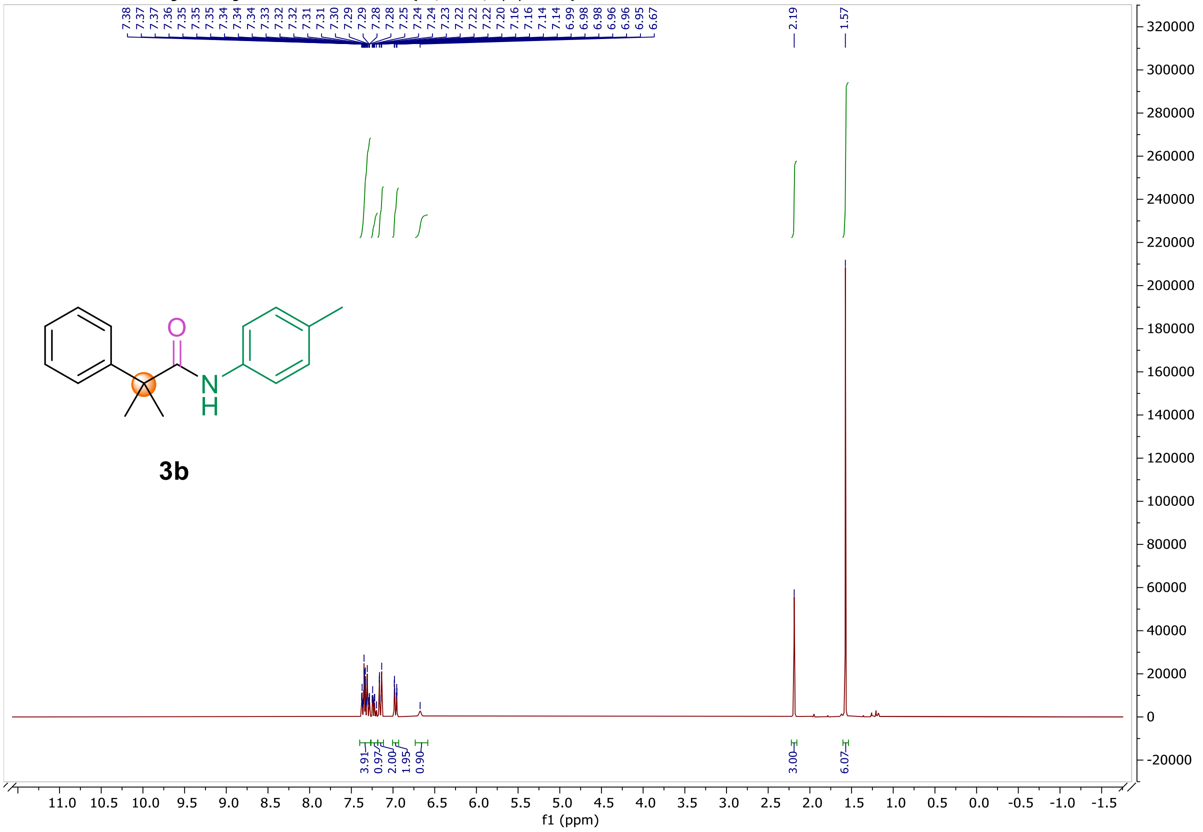


231010.f304.11.fid — Xingwei Gu gxw-195-2 — Au13C CDCl3 {C:\Bruker\TopSpin3.6.2} 2310 4 - 75 MHz

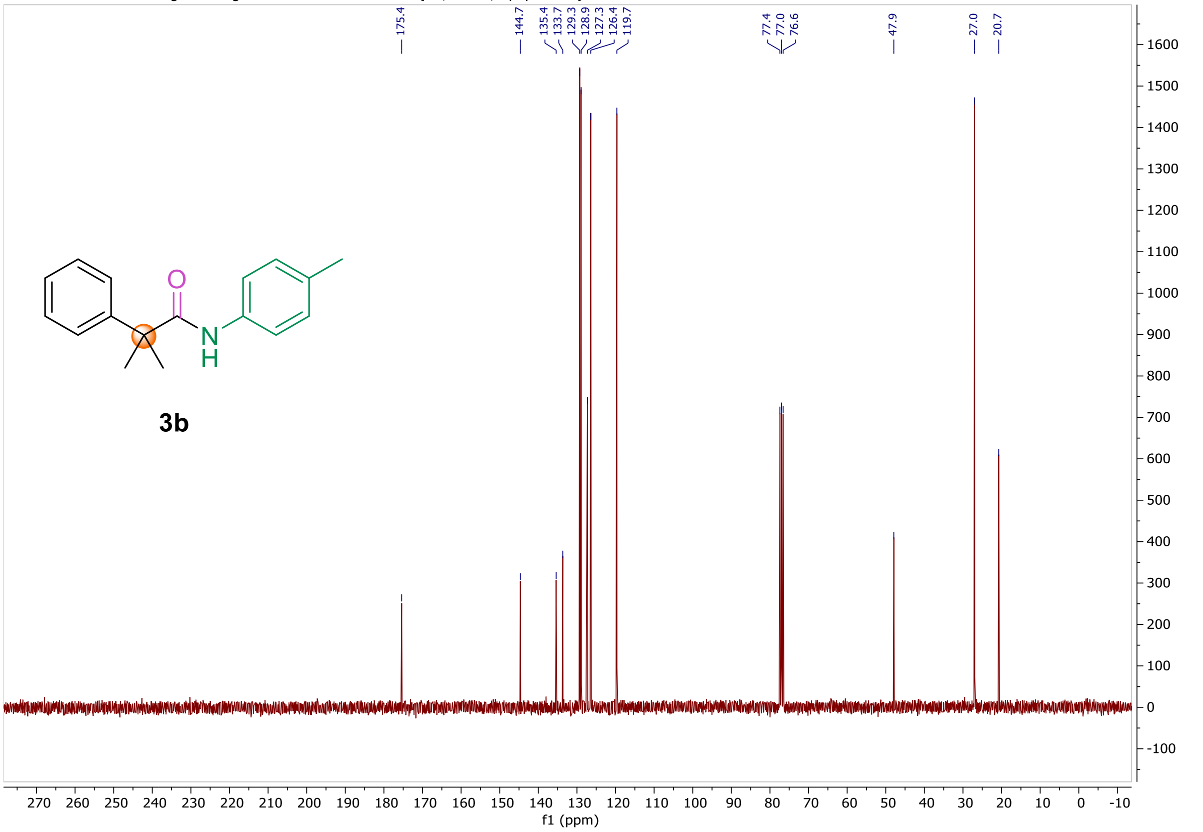

230919.f314.10.fid — Xingwei Gu gxw-185-19 — PROTON CDCl3 {C:\Bruker\TopSpin3.6.2} 2309 14 - 300 MHz

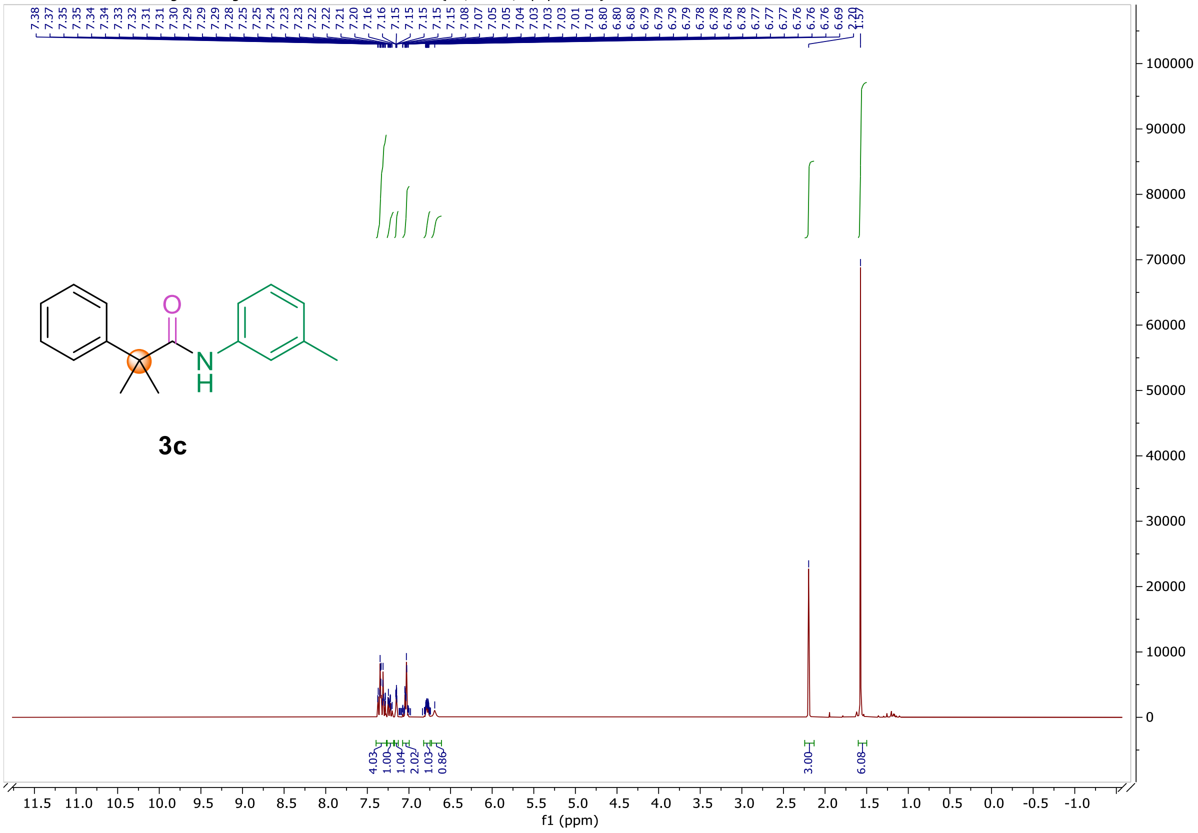


230919.f314.11.fid — Xingwei Gu gxw-185-19 — Au13C CDCl3 {C:\Bruker\TopSpin3.6.2} 2309 14 - 75 MHz

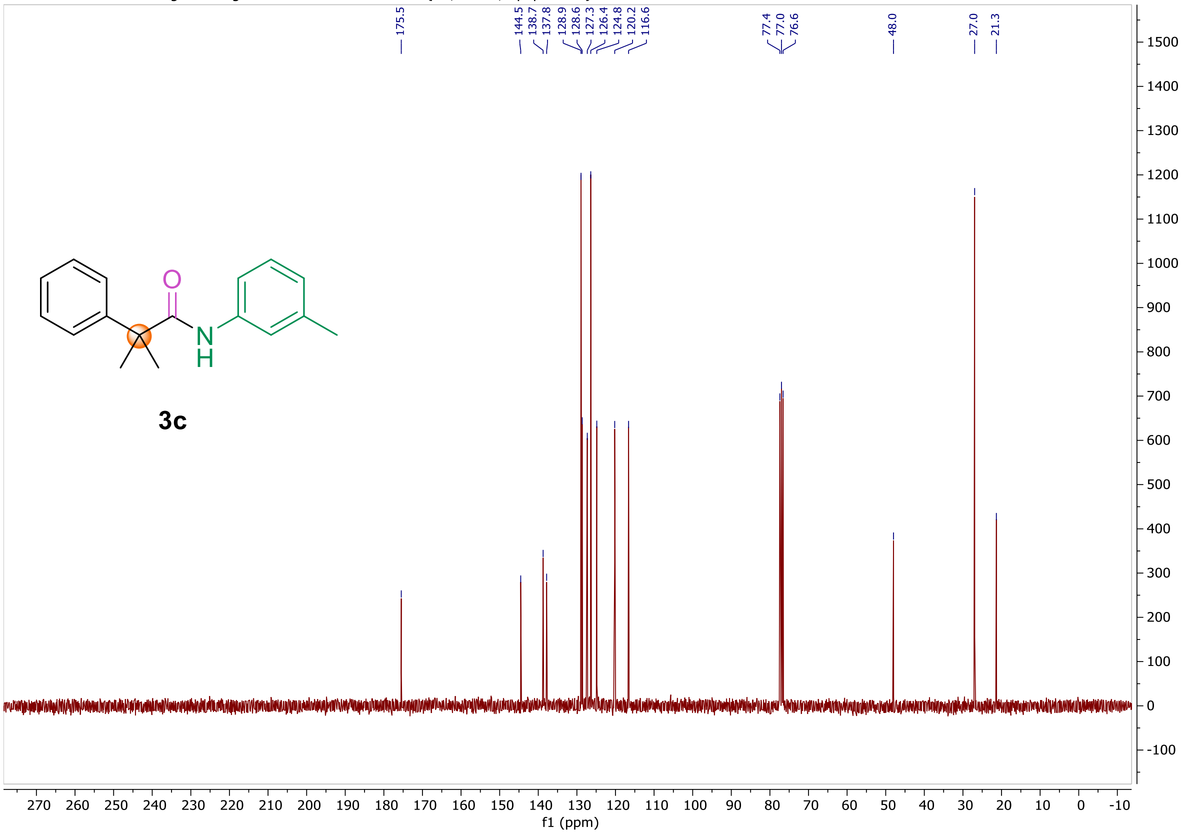

230919.f312.10.fid — Xingwei Gu gxw-185-16 — PROTON CDCl3 {C:\Bruker\TopSpin3.6.2} 2309 12 - 300 MHz

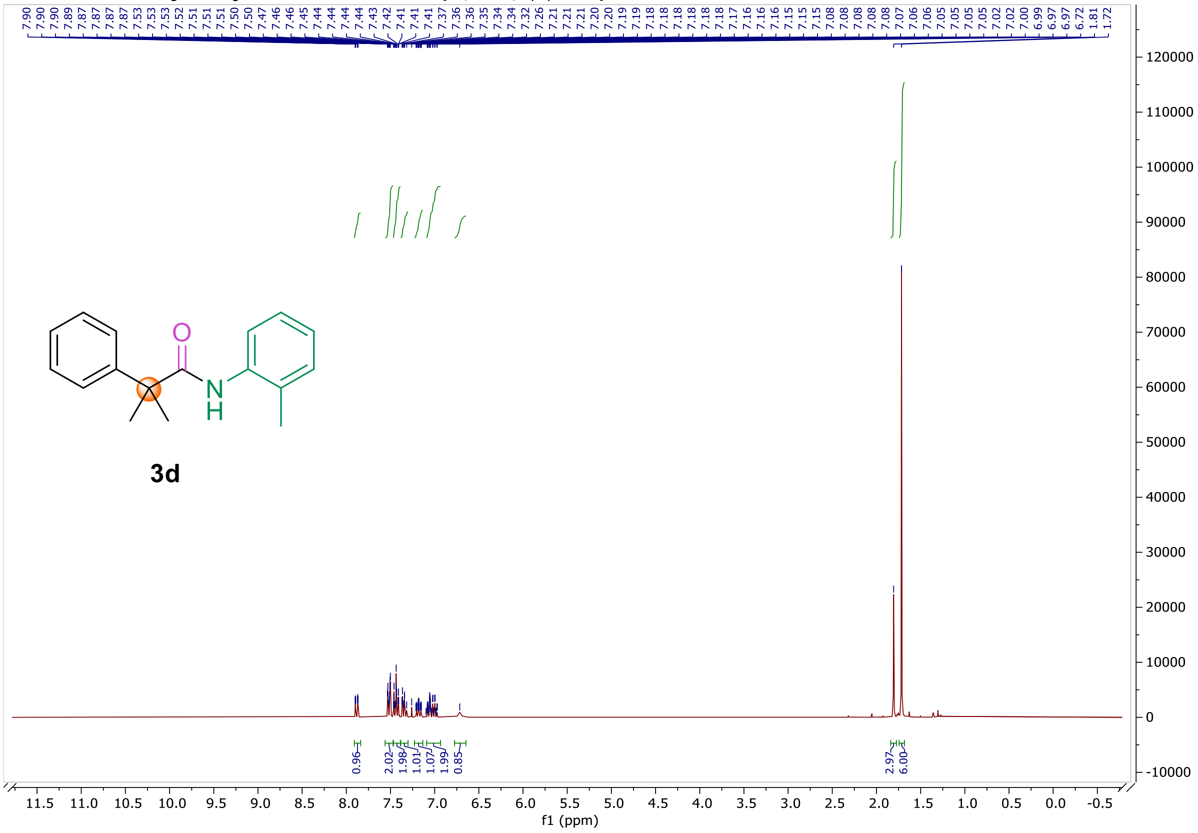


230919.f312.11.fid — Xingwei Gu gxw-185-16 — Au13C CDCl3 {C:\Bruker\TopSpin3.6.2} 2309 12 - 75 MHz

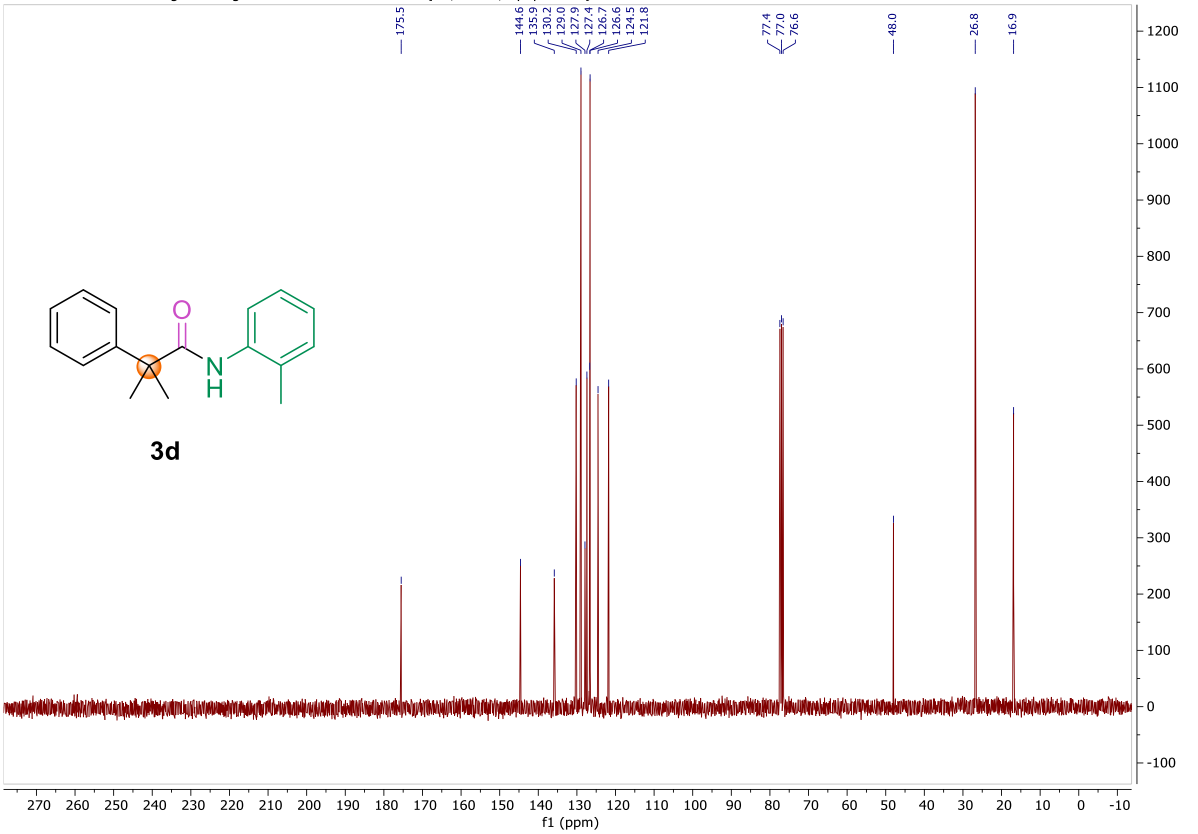

230904.318.10.fid — Xingwei Gu, gxw-185-2 — Au1H CDCl3 {C:\Bruker\TopSpin3.6.2} 2309 18 - 300 MHz

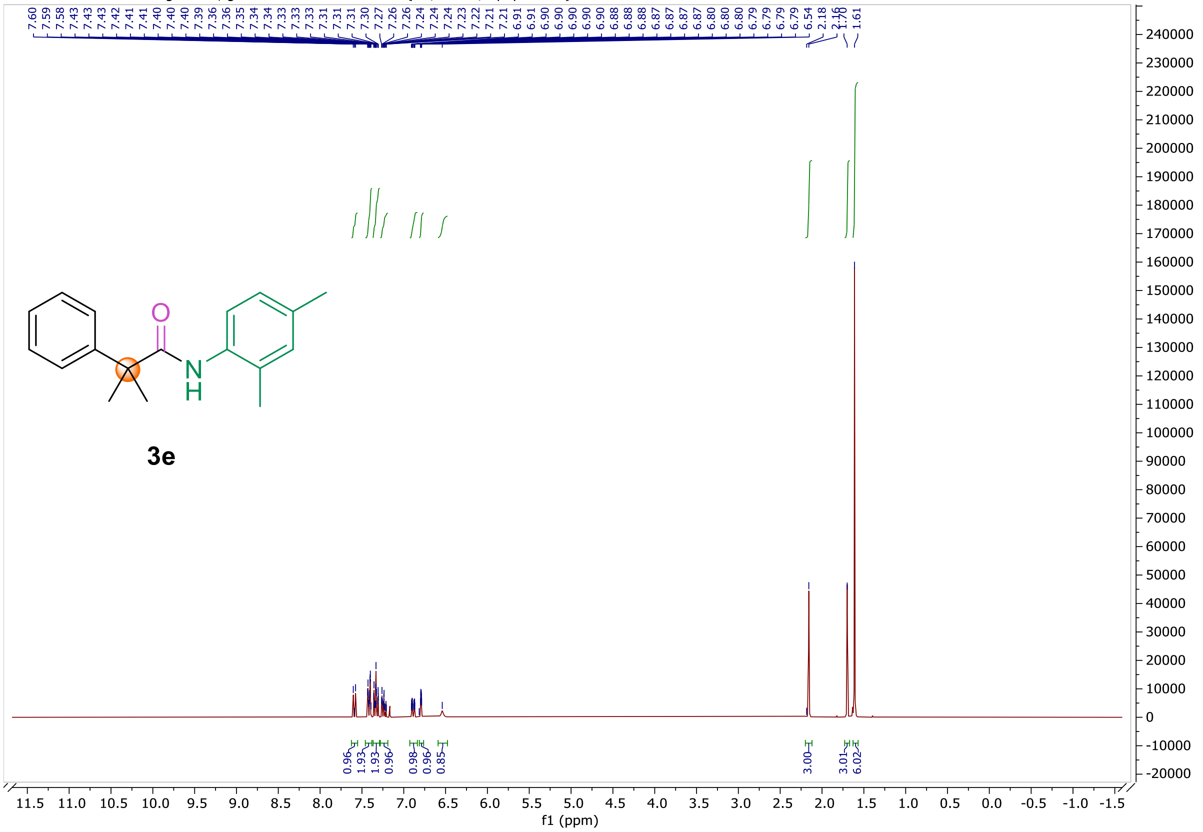


230904.318.11.fid — Xingwei Gu, gxw-185-2 — Au13C CDCl3 {C:\Bruker\TopSpin3.6.2} 2309 18 - 75 MHz

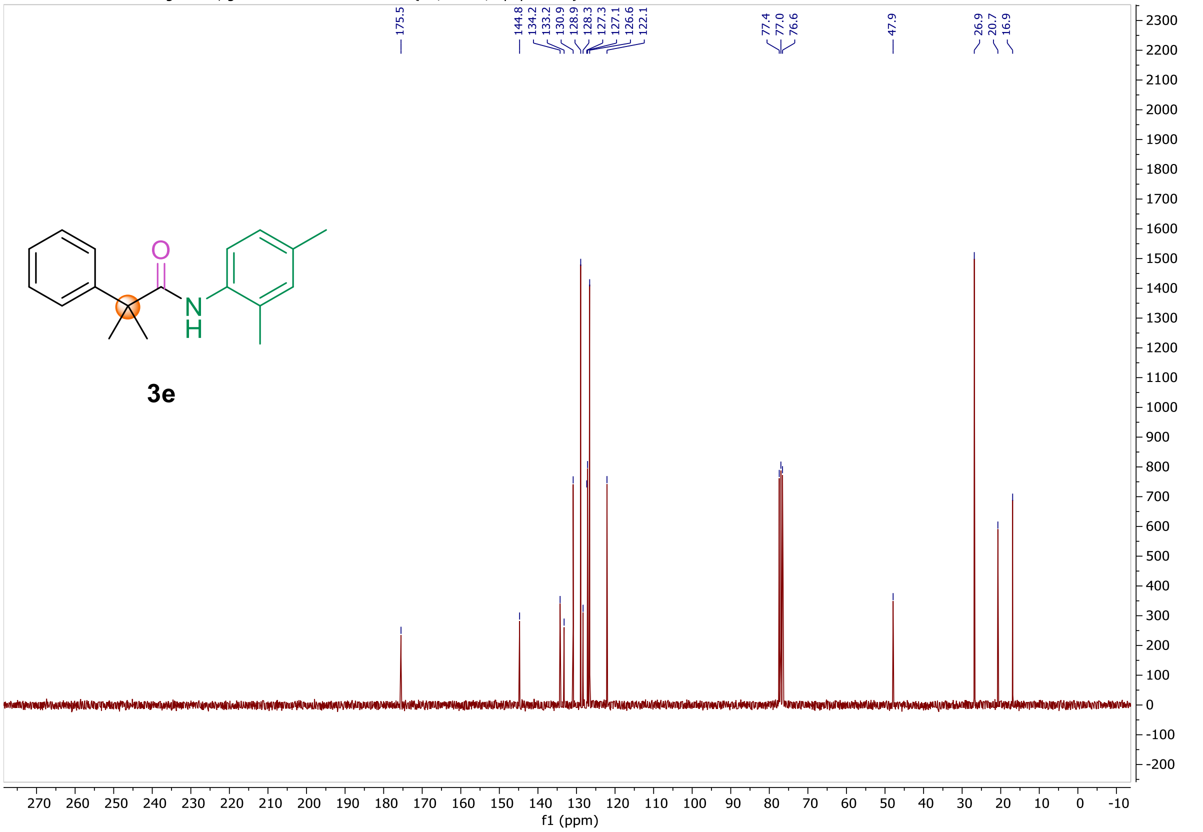

230905.323.10.fid — Xingwei Gu, gxw-185-9 — Au1H CDCl3 {C:\Bruker\TopSpin3.6.2} 2309 23 - 300 MHz

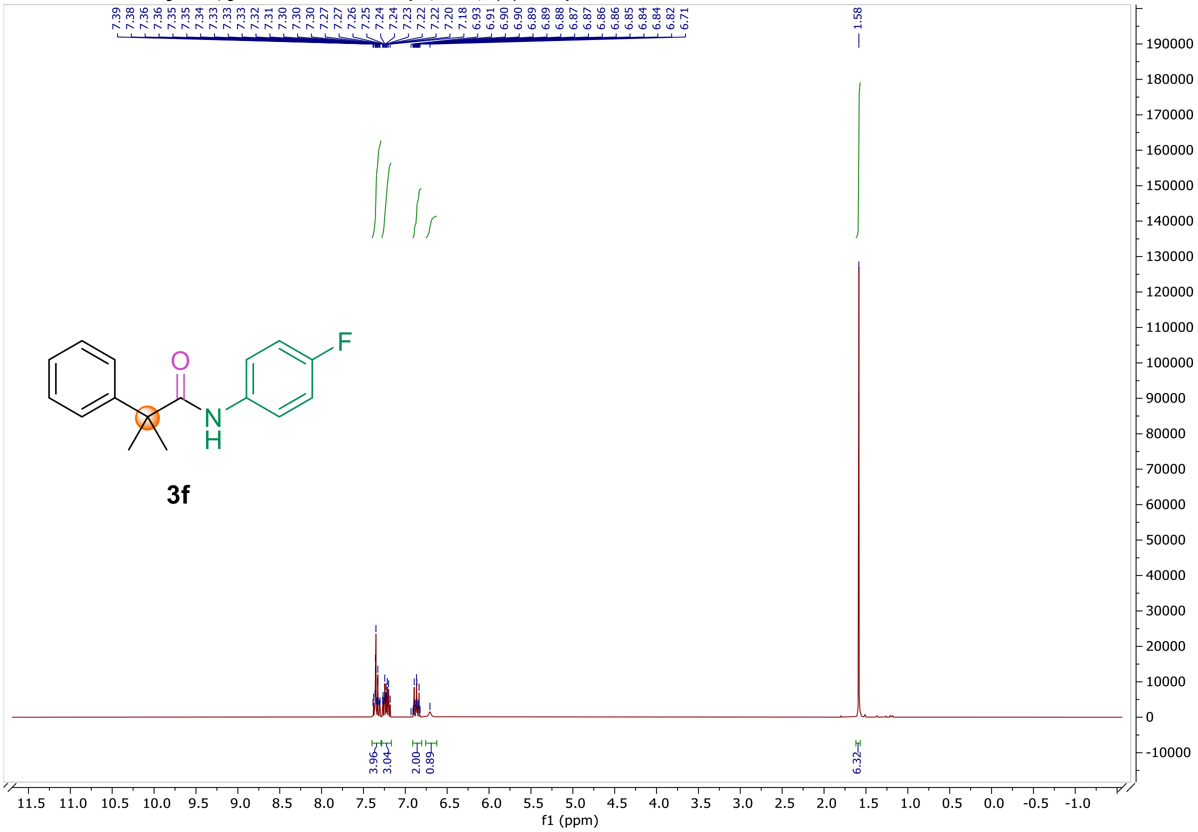


230905.323.11.fid — Xingwei Gu, gxw-185-9 — Au13C CDCl3 {C:\Bruker\TopSpin3.6.2} 2309 23 - 75 MHz

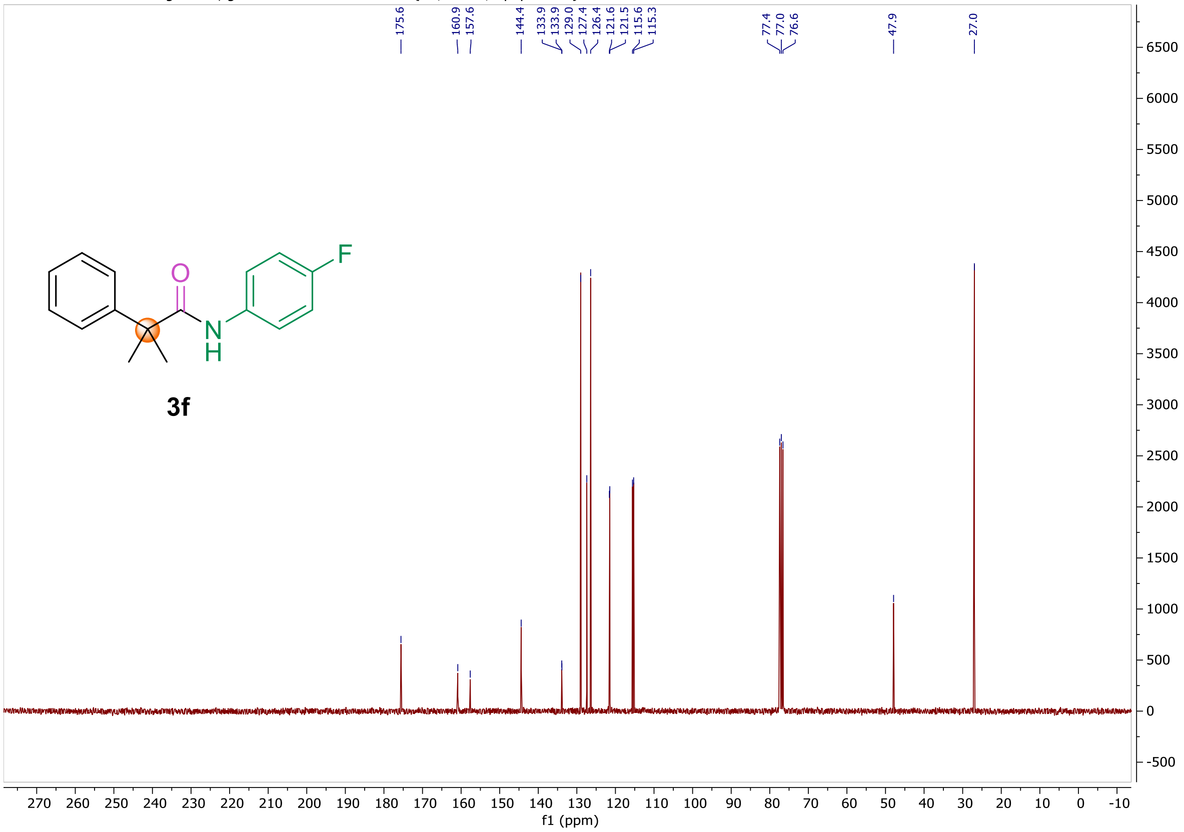

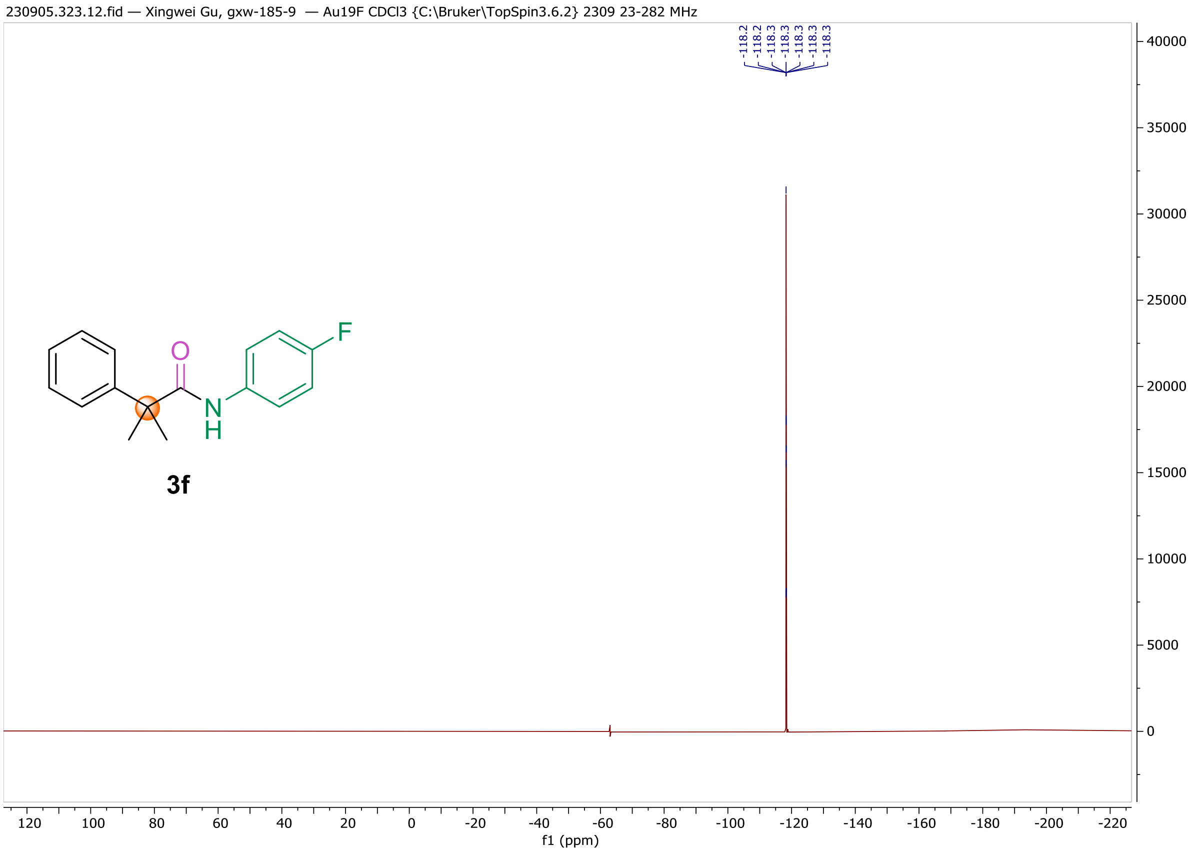
230905.323.12.fid — Xingwei Gu, gxw-185-9 — Au19F CDCl3 {C:\Bruker\TopSpin3.6.2} 2309 23-282 MHz
-118.2
-118.2
-118.3
-118.3
-118.3
-118.3
-118.3
O
N
H
F
3f
40000
35000
30000
25000
20000
15000
10000
5000
0
120 100 80 60 40 20 0 -20 -40 -60 -80 -100 -120 -140 -160 -180 -200 -220
f1 (ppm)

230914.328.10.fid — Xingwei Gu, gxw-184-14 — Au1H CDCl3 {C:\Bruker\TopSpin3.6.2} 2309 28 - 300 MHz

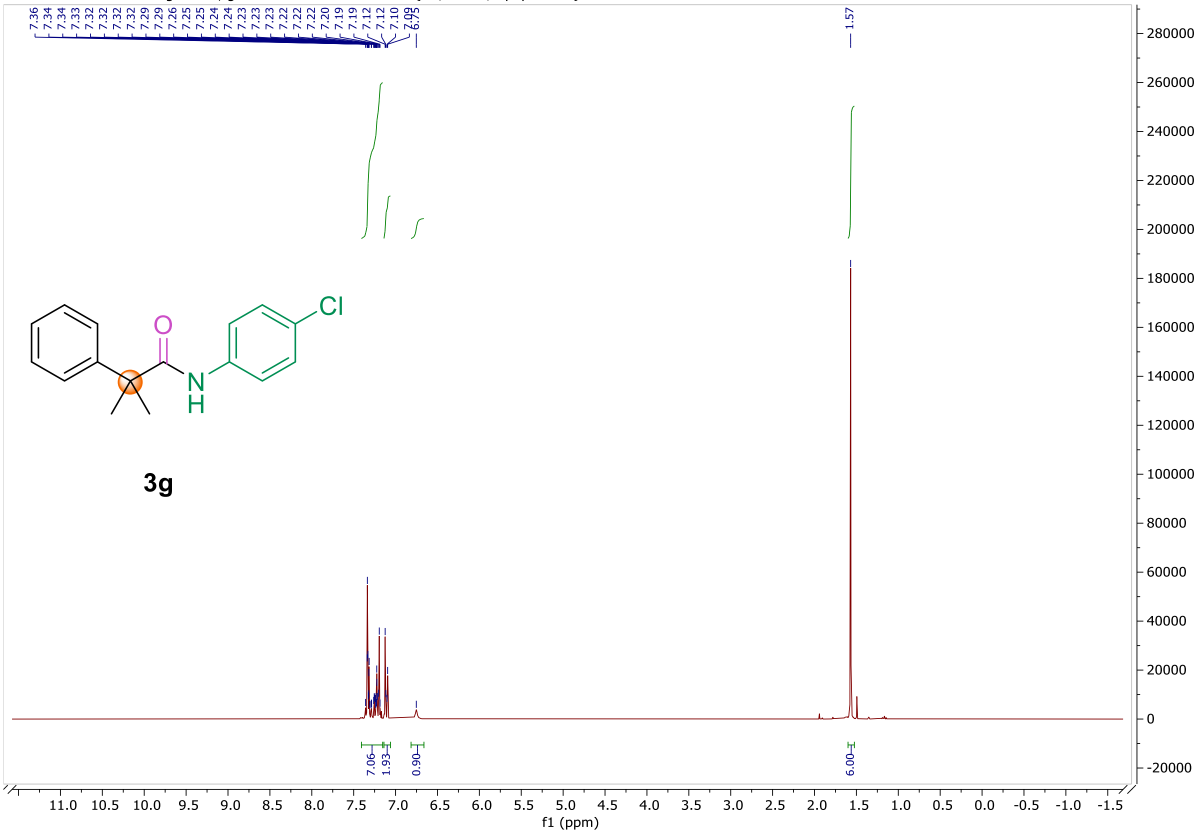


230914.328.11.fid — Xingwei Gu, gxw-184-14 — Au13C CDCl3 {C:\Bruker\TopSpin3.6.2} 2309 28 - 75 MHz

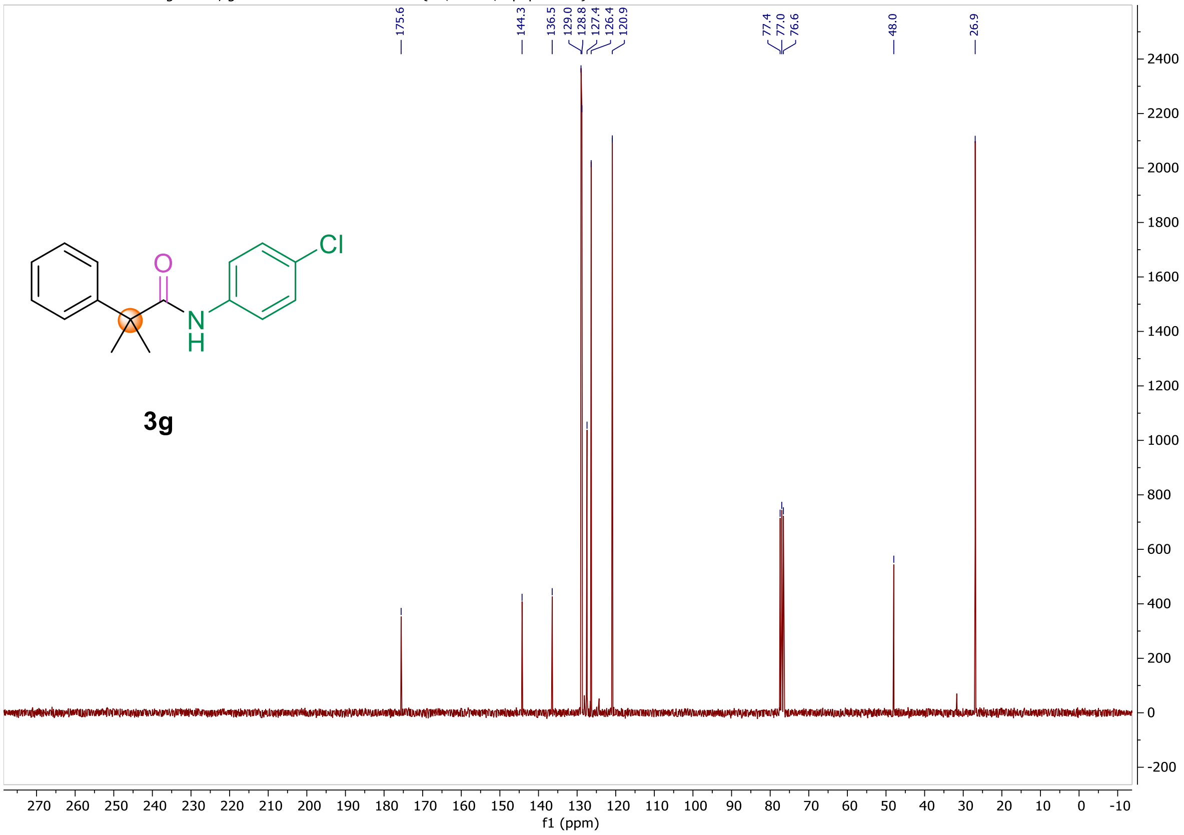

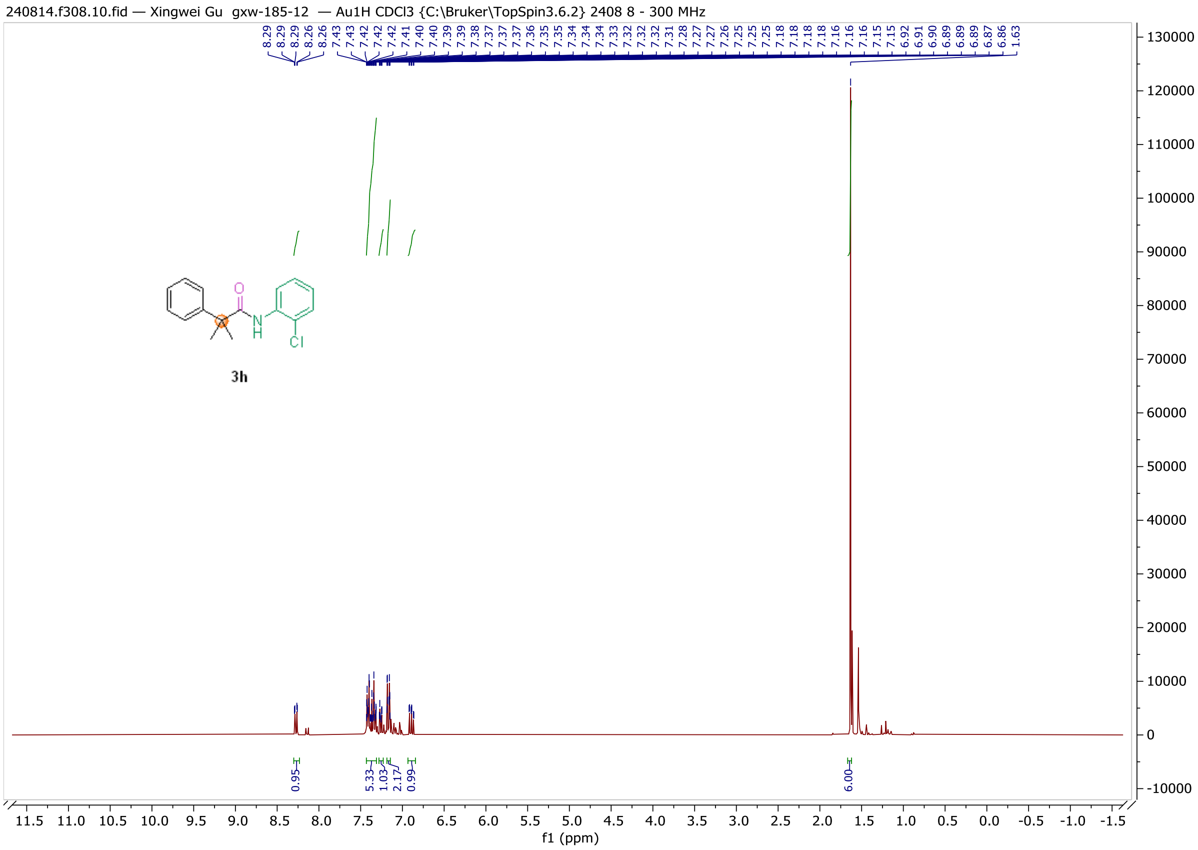
240814.f308.10.fid — Xingwei Gu gxw-185-12 — Au1H CDCl3 {C:\Bruker\TopSpin3.6.2} 2408 8 - 300 MHz
8.29 8.29 8.29 8.26 8.26 7.43 7.43 7.42 7.42 7.42 7.41 7.40 7.40 7.39 7.39 7.38 7.37 7.37 7.37 7.36 7.35 7.35 7.34 7.34 7.34 7.33 7.32 7.32 7.32 7.32 7.31 7.28 7.27 7.27 7.26 7.25 7.25 7.25 7.18 7.18 7.18 7.18 7.16 7.16 7.16 7.15 6.92 6.91 6.90 6.89 6.89 6.89 6.87 6.86 1.63
O
N
H
Cl
3h
0.95
5.33
1.03
2.17
0.99
6.00
f1 (ppm)

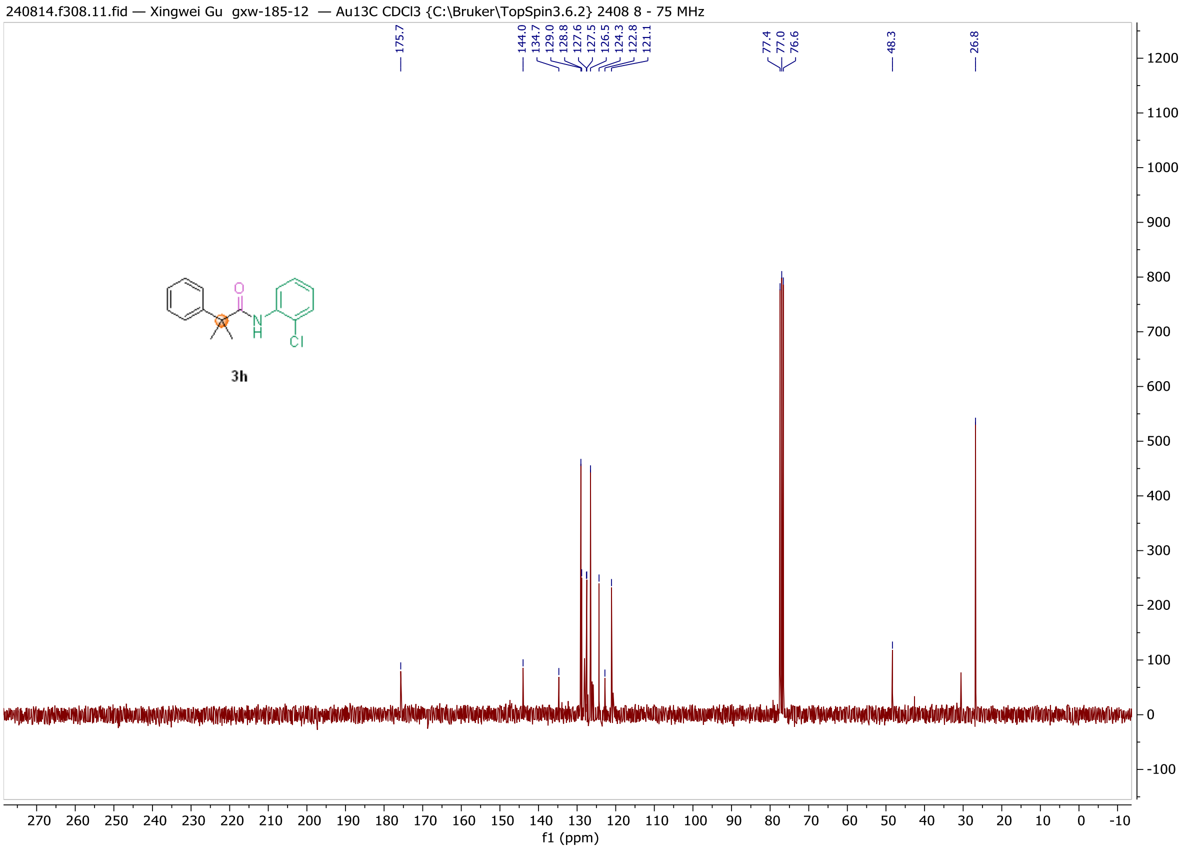
240814.f308.11.fid — Xingwei Gu gxw-185-12 — Au13C CDCl3 {C:\Bruker\TopSpin3.6.2} 2408 8 - 75 MHz
175.7
144.0 134.7 129.0 128.8 127.6 127.5 126.5 124.3 122.8 121.1
77.4 77.0 76.6
48.3
26.8
O
N
H
Cl
3h
f1 (ppm)

231010.f303.10.fid — Xingwei Gu gxw-195-1 — Au1H CDCl3 {C:\Bruker\TopSpin3.6.2} 2310 3 - 300 MHz

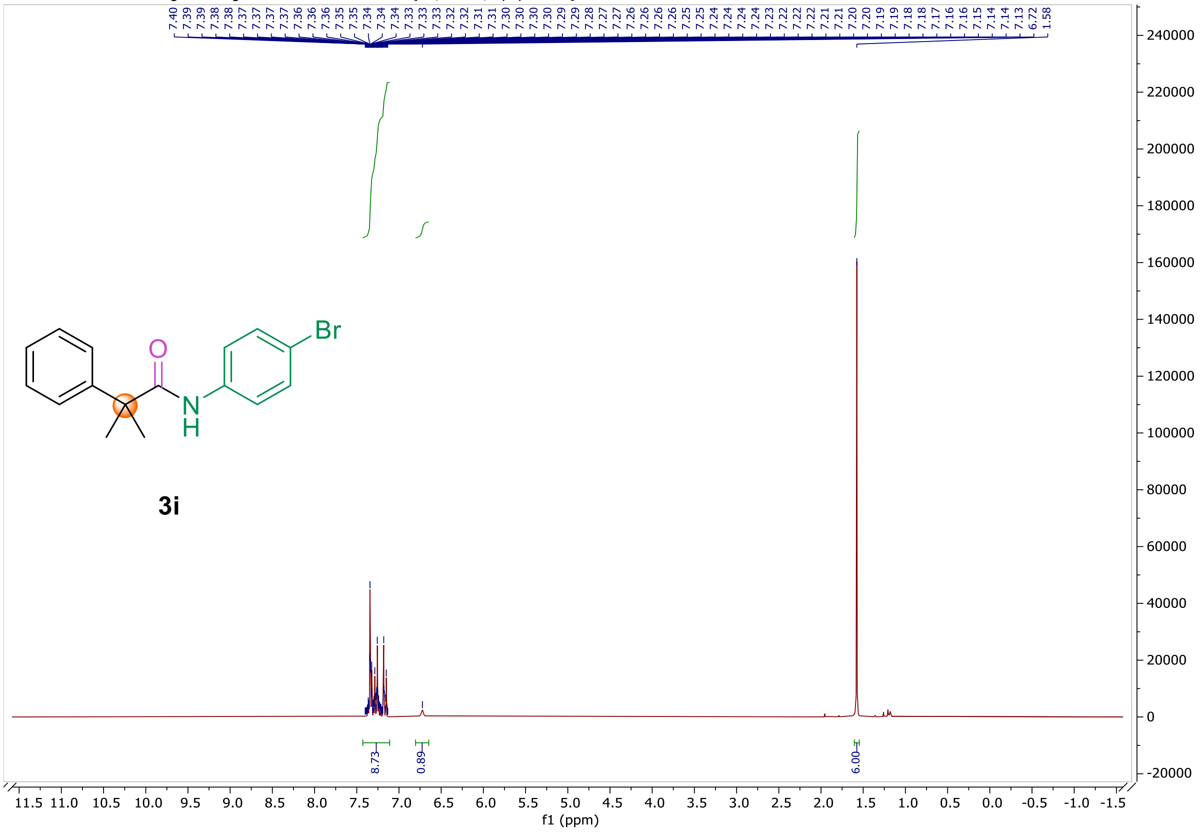

231010.f303.11.fid — Xingwei Gu gxw-195-1 — Au13C CDCl3 {C:\Bruker\TopSpin3.6.2} 2310 3 - 75 MHz

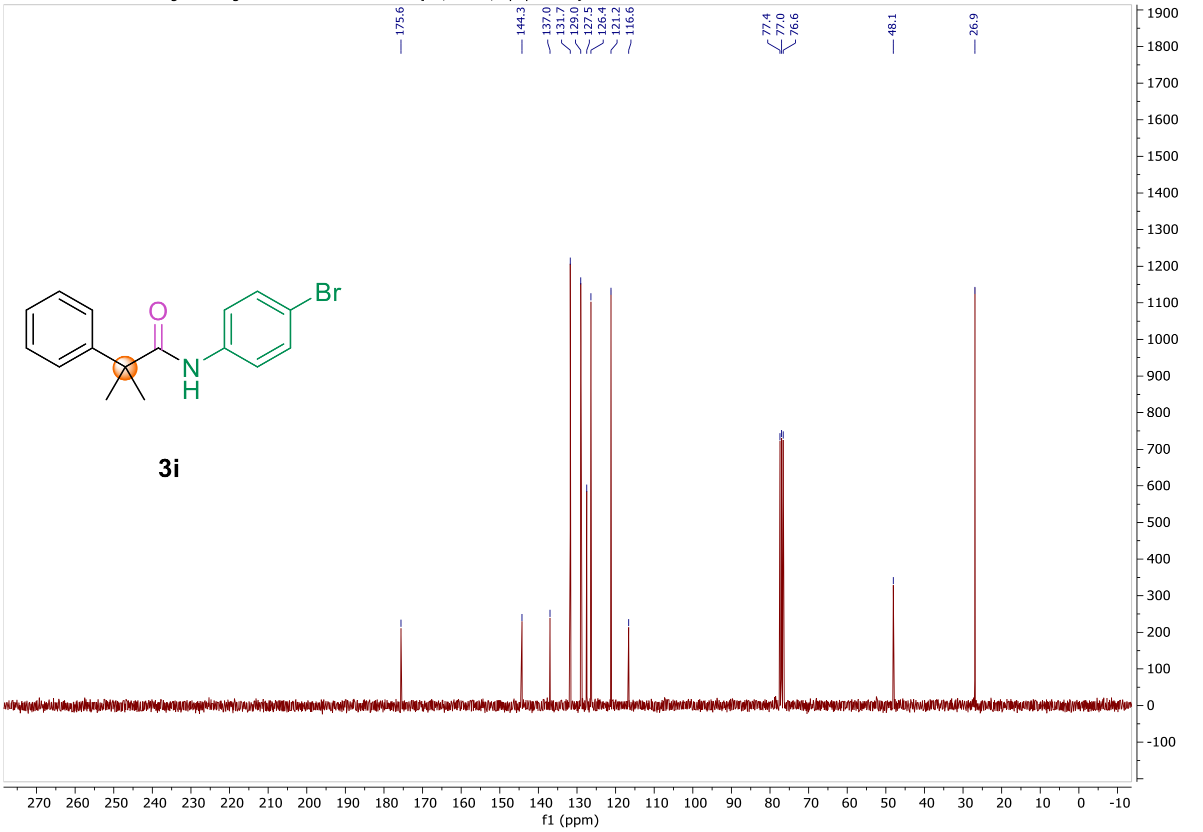

240814.f307.10.fid — Xingwei Gu gxw-185-4 — Au1H CDCl3 {C:\Bruker\TopSpin3.6.2} 2408 7 - 300 MHz

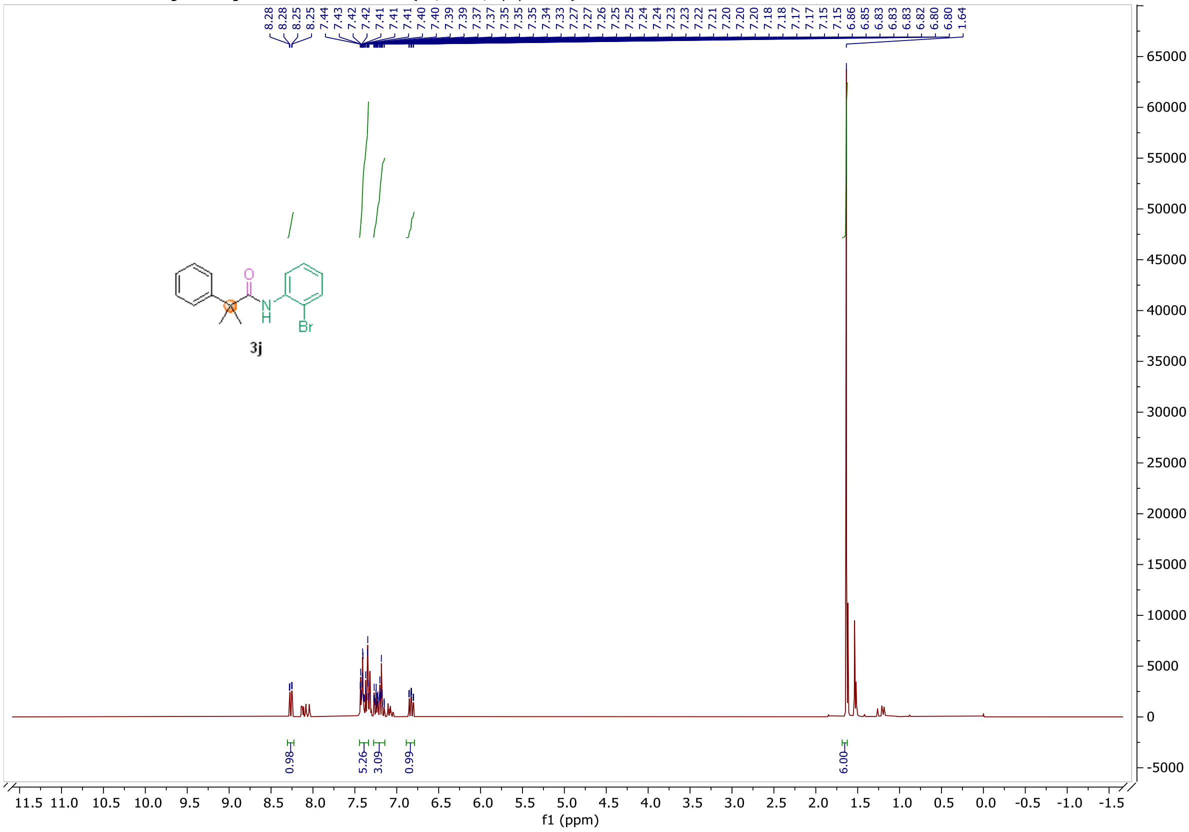


240814.f307.11.fid — Xingwei Gu gxw-185-4 — Au13C CDCl3 {C:\Bruker\TopSpin3.6.2} 2408 7 - 75 MHz

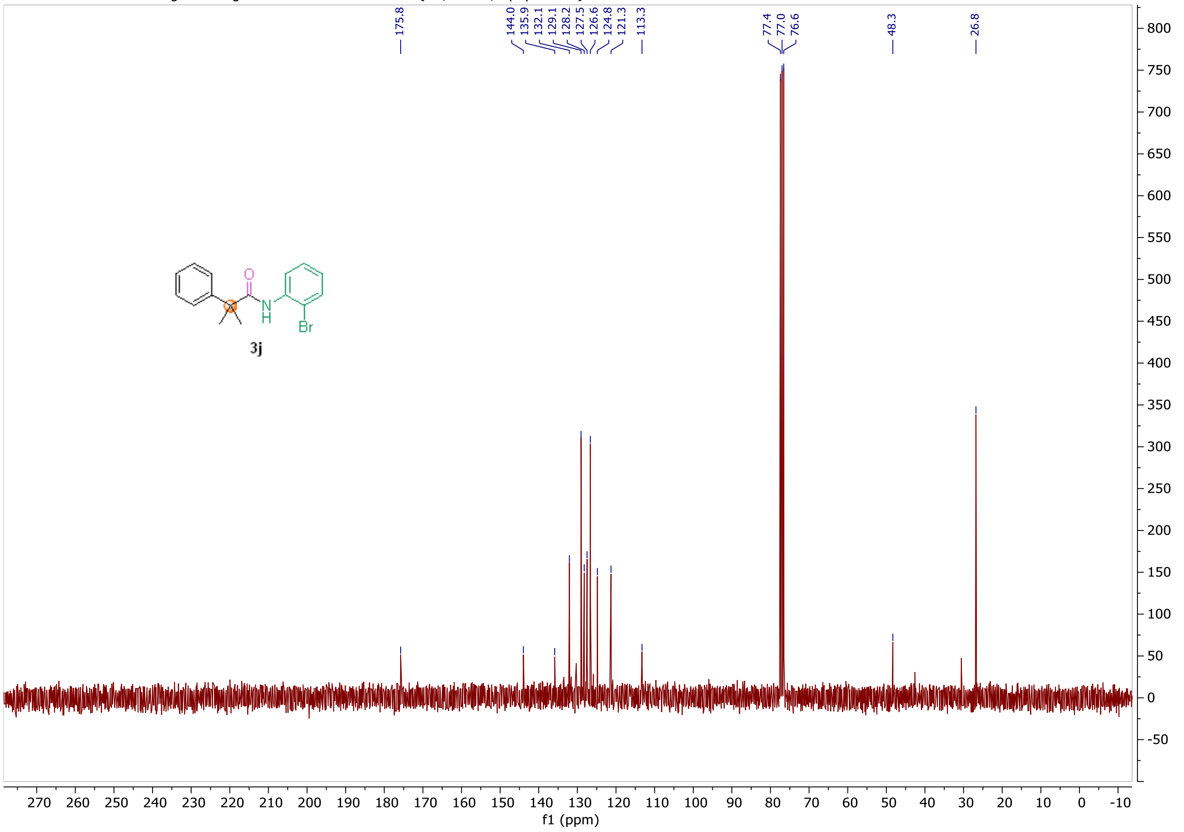

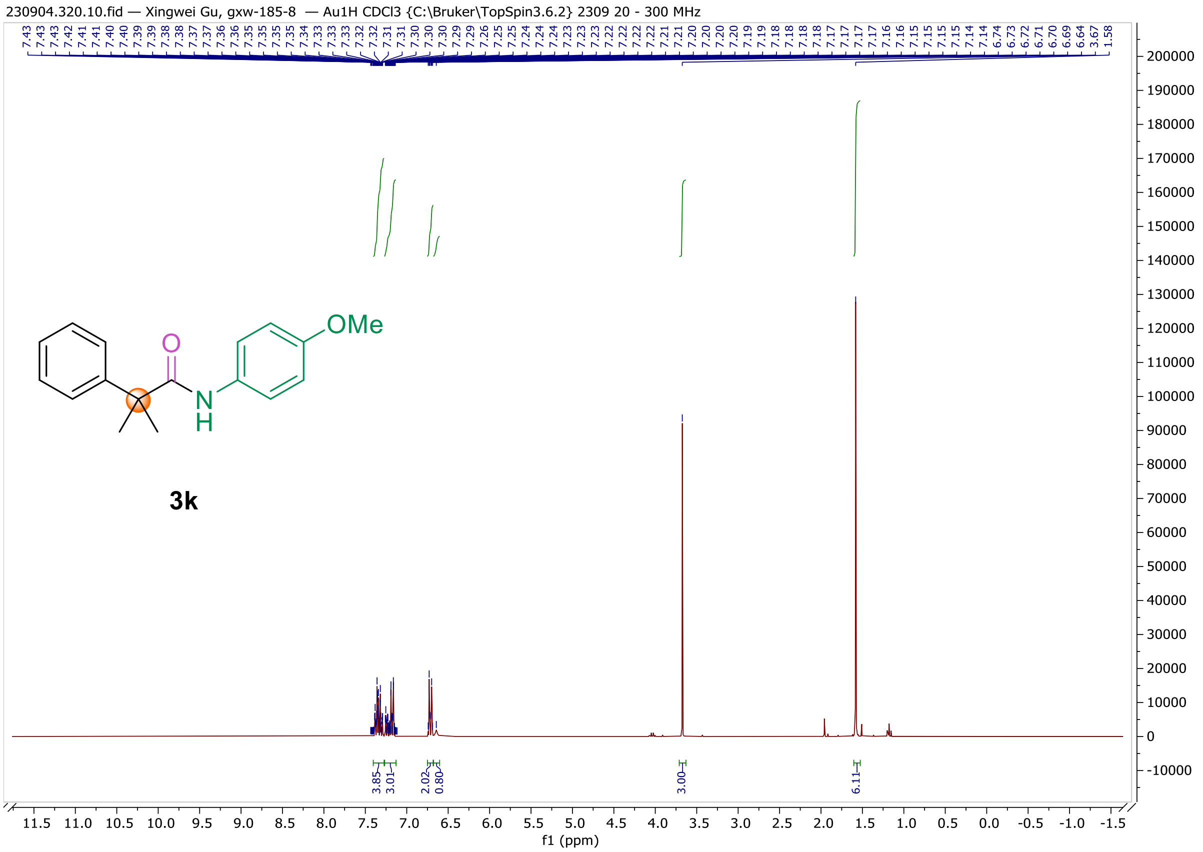
230904.320.10.fid — Xingwei Gu, gxw-185-8 — Au1H CDCl3 {C:\Bruker\TopSpin3.6.2} 2309 20 - 300 MHz
OMe
O
N
H
3k
f1 (ppm)

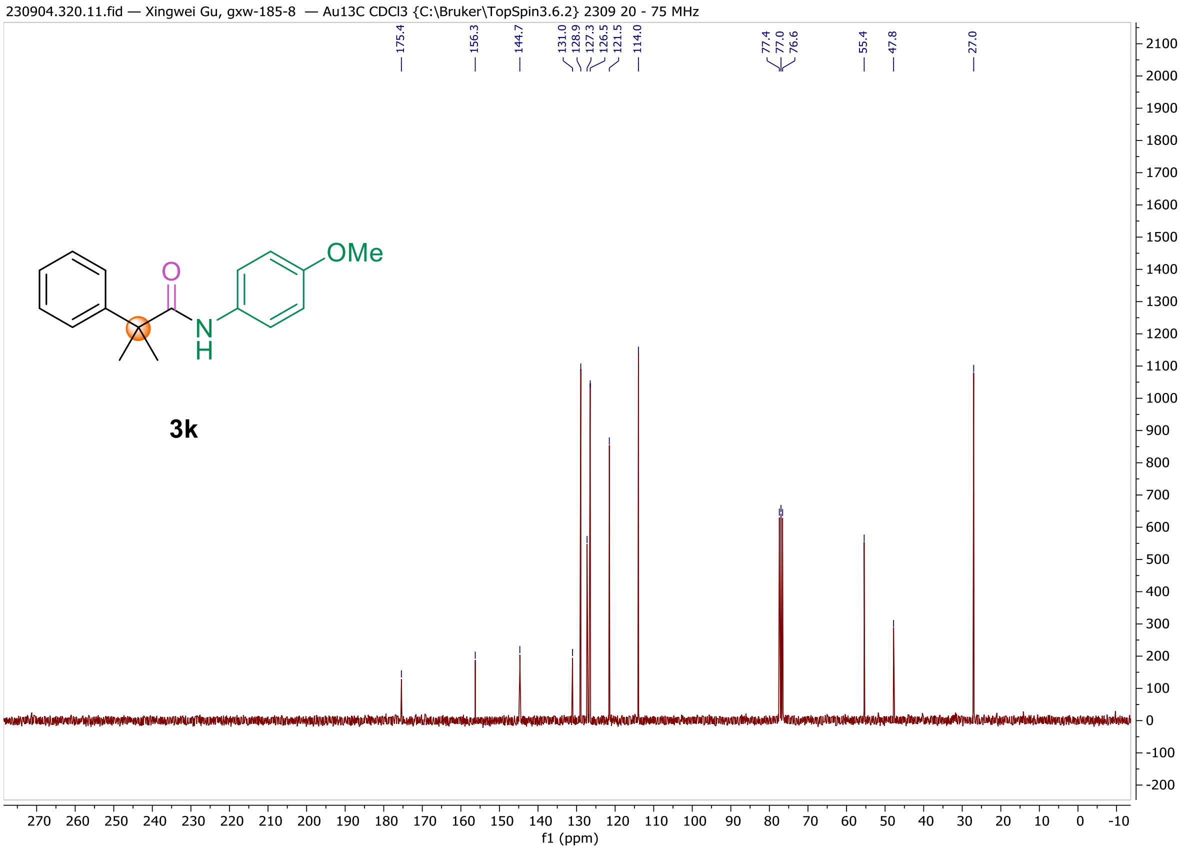
230904.320.11.fid — Xingwei Gu, gxw-185-8 — Au13C CDCl3 {C:\Bruker\TopSpin3.6.2} 2309 20 - 75 MHz
175.4
156.3
144.7
131.0
128.9
127.3
126.5
121.5
114.0
77.4
77.0
76.6
55.4
47.8
27.0
OMe
O
N
H
3k
f1 (ppm)

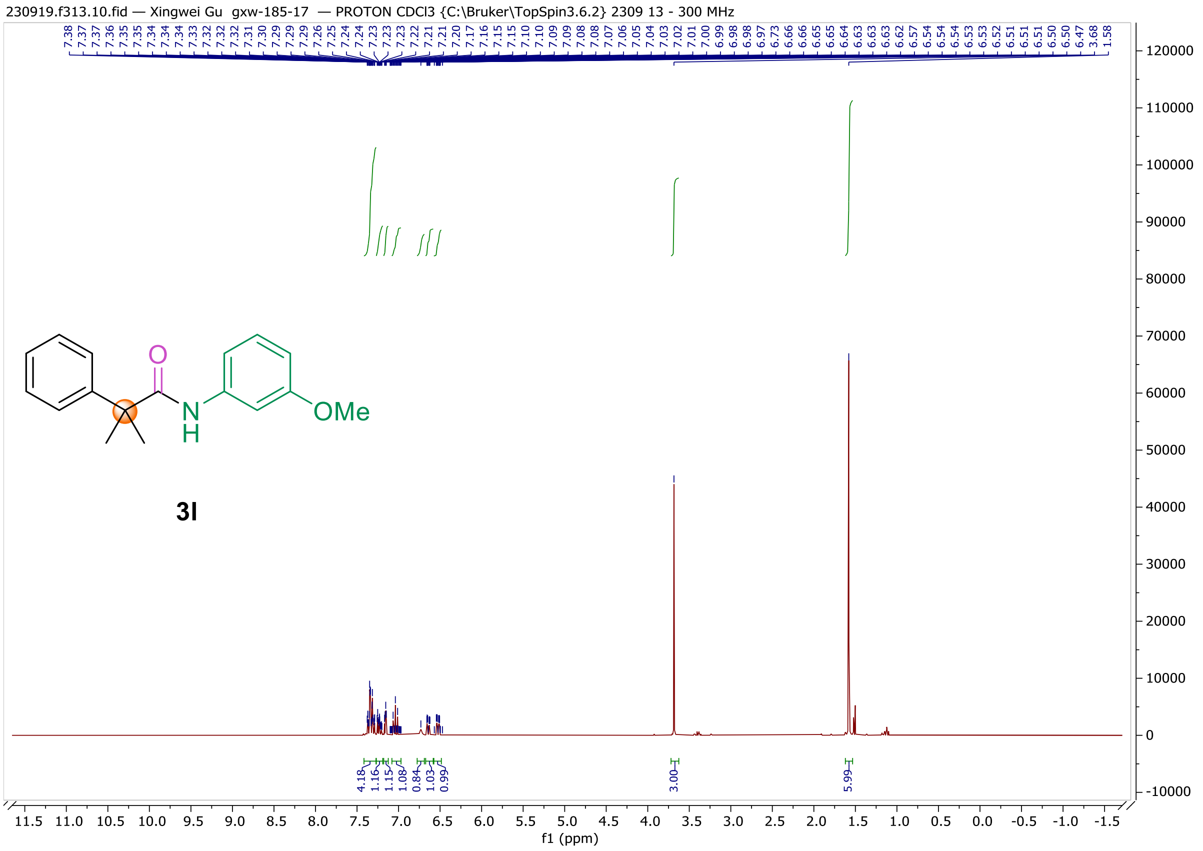
230919.f313.10.fid — Xingwei Gu gxw-185-17 — PROTON CDCl3 {C:\Bruker\TopSpin3.6.2} 2309 13 - 300 MHz
OMe
3l
f1 (ppm)

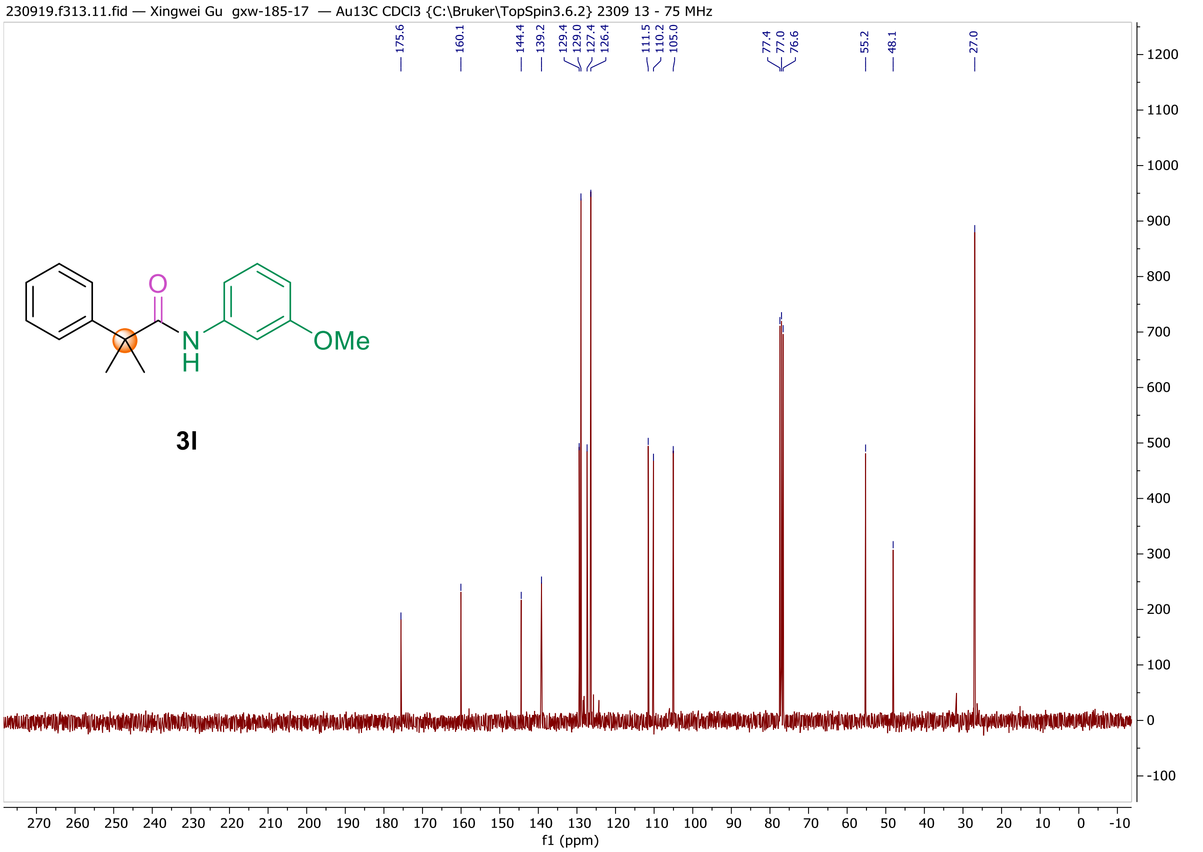
230919.f313.11.fid — Xingwei Gu gxw-185-17 — Au13C CDCl3 {C:\Bruker\TopSpin3.6.2} 2309 13 - 75 MHz
175.6
160.1
144.4
139.2
129.4
129.0
127.4
126.4
111.5
110.2
105.0
77.4
77.0
76.6
55.2
48.1
27.0
OMe
3l
f1 (ppm)

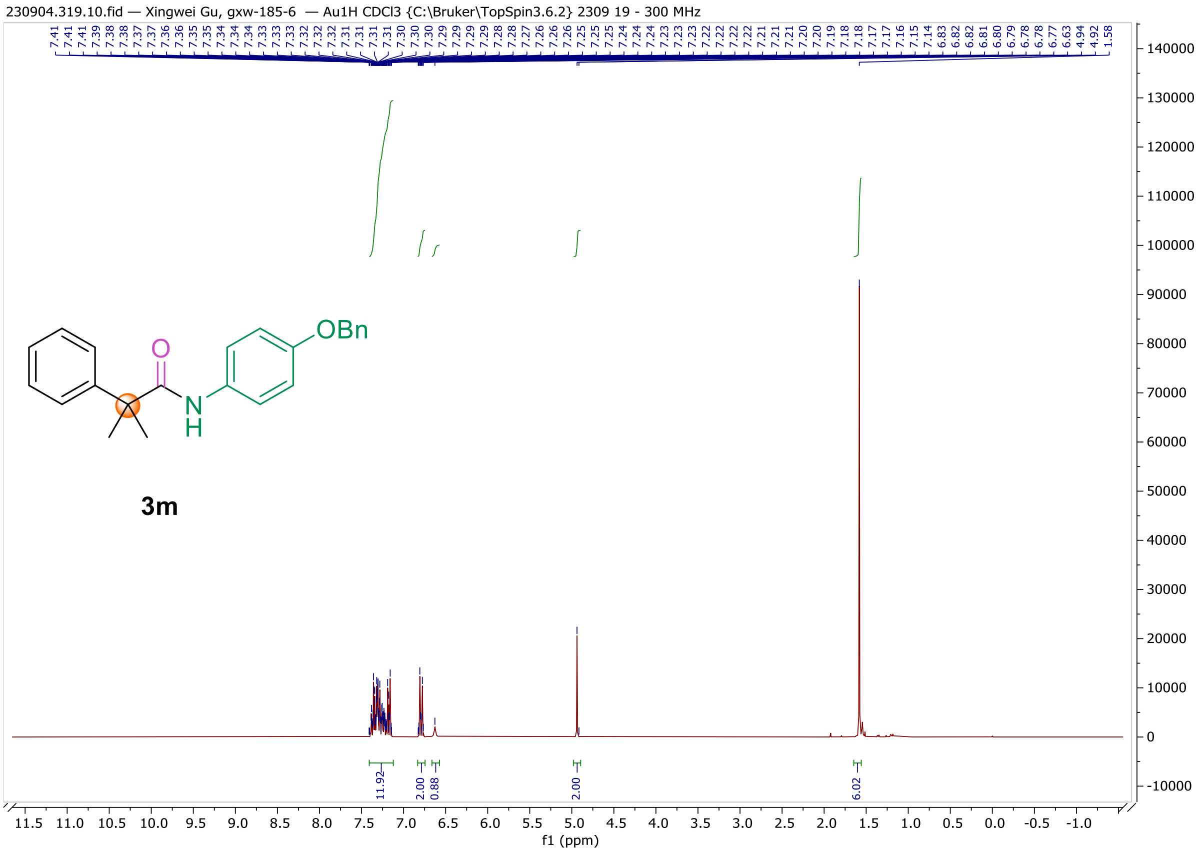
230904.319.10.fid — Xingwei Gu, gxw-185-6 — Au1H CDCl3 {C:\Bruker\TopSpin3.6.2} 2309 19 - 300 MHz
OBn
O
N
H
f1 (ppm)

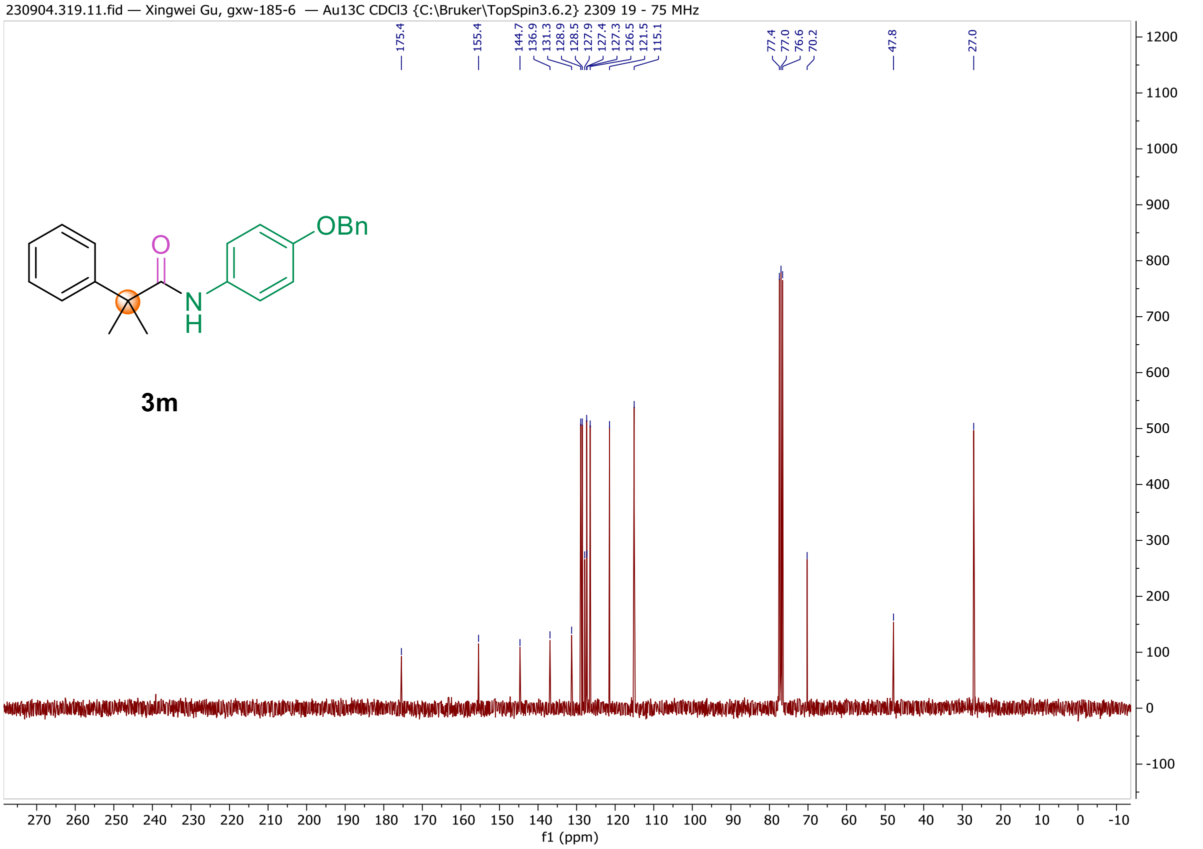
230904.319.11.fid — Xingwei Gu, gxw-185-6 — Au13C CDCl3 {C:\Bruker\TopSpin3.6.2} 2309 19 - 75 MHz
175.4
155.4
144.7
136.9
131.3
128.9
128.5
127.9
127.4
127.3
126.5
121.5
115.1
77.4
77.0
76.6
70.2
47.8
27.0
OBn
O
N
H
f1 (ppm)

230914.330.10.fid — Xingwei Gu, gxw-184-15 — Au1H CDCl3 {C:\Bruker\TopSpin3.6.2} 2309 30 - 300 MHz

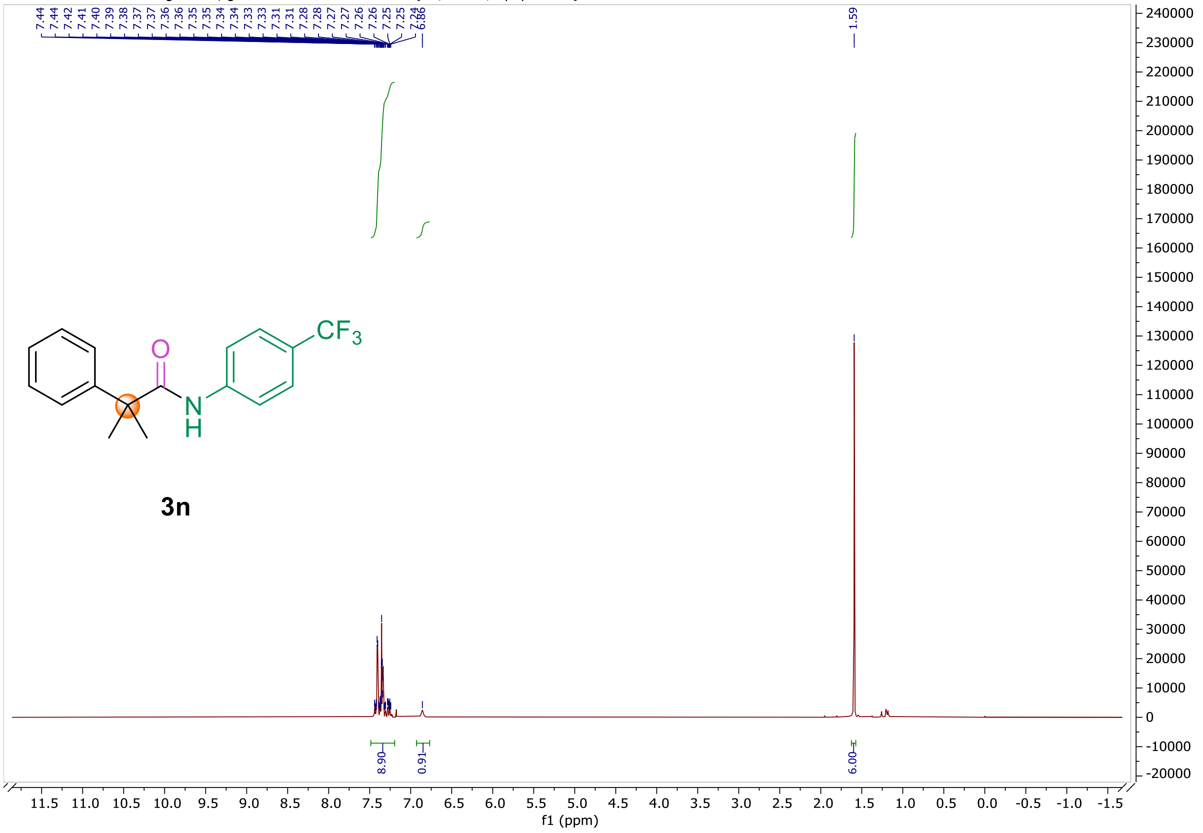


230914.330.11.fid — Xingwei Gu, gxw-184-15 — Au13C CDCl3 {C:\Bruker\TopSpin3.6.2} 2309 30 - 75 MHz

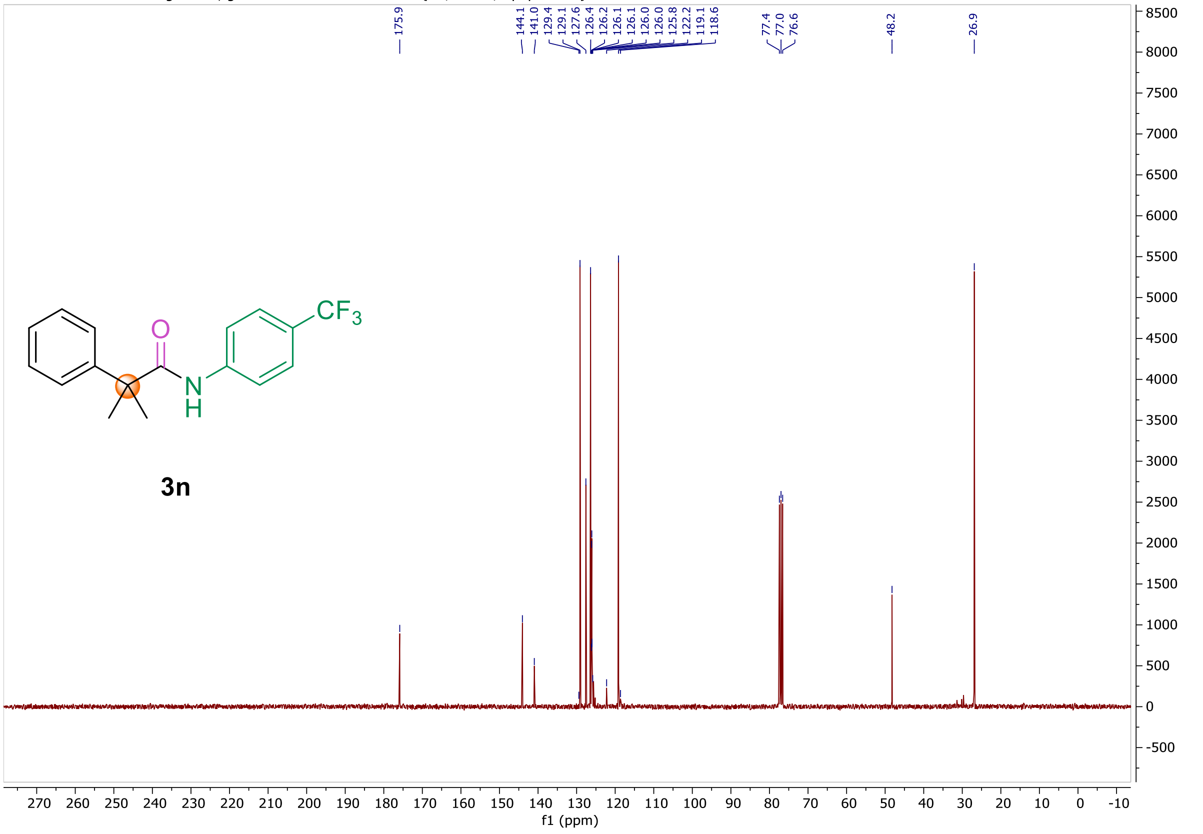

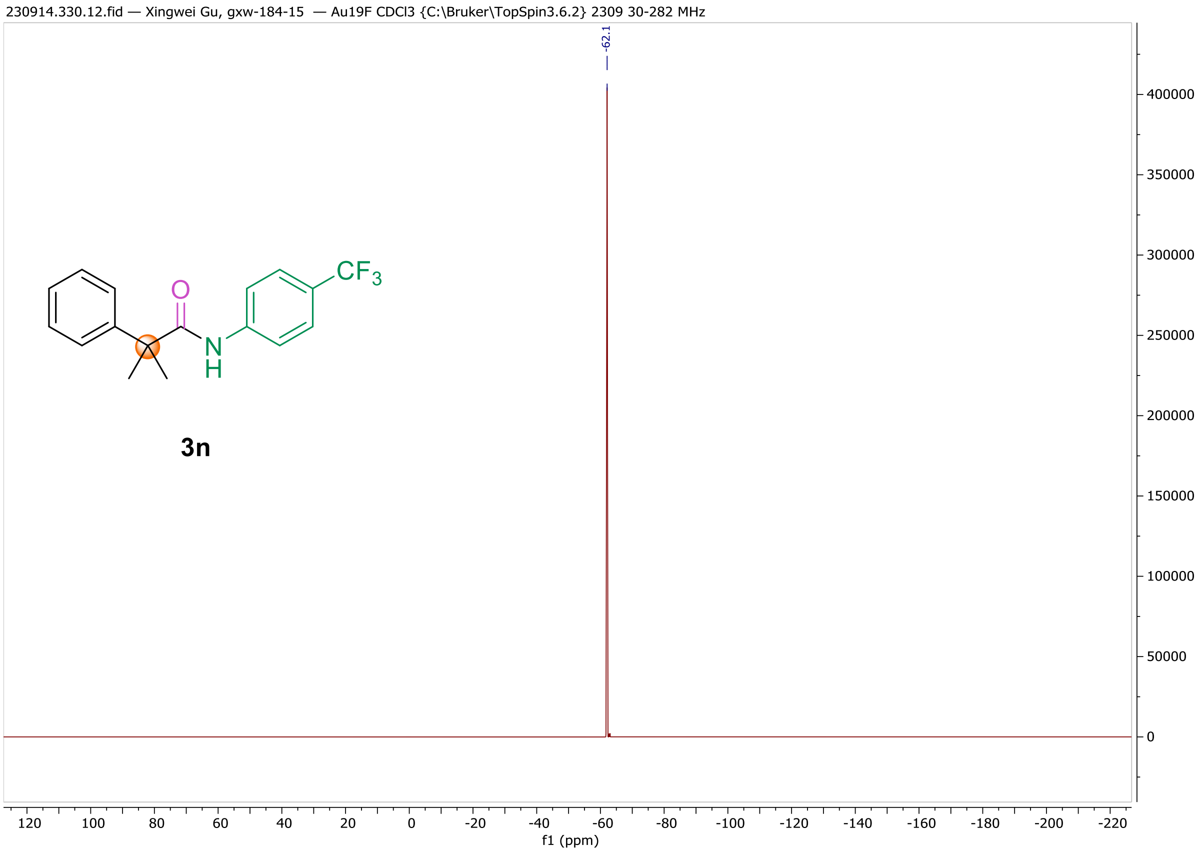
230914.330.12.fid — Xingwei Gu, gxw-184-15 — Au19F CDCl3 {C:\Bruker\TopSpin3.6.2} 2309 30-282 MHz
-62.1
CF3
O
N
H
3n
400000
350000
300000
250000
200000
150000
100000
50000
0
120 100 80 60 40 20 0 -20 -40 -60 -80 -100 -120 -140 -160 -180 -200 -220
f1 (ppm)

231006.f331.10.fid — Xingwei Gu gxw-185-18 — Au1H CDCl3 {C:\Bruker\TopSpin3.6.2} 2310 31 - 300 MHz

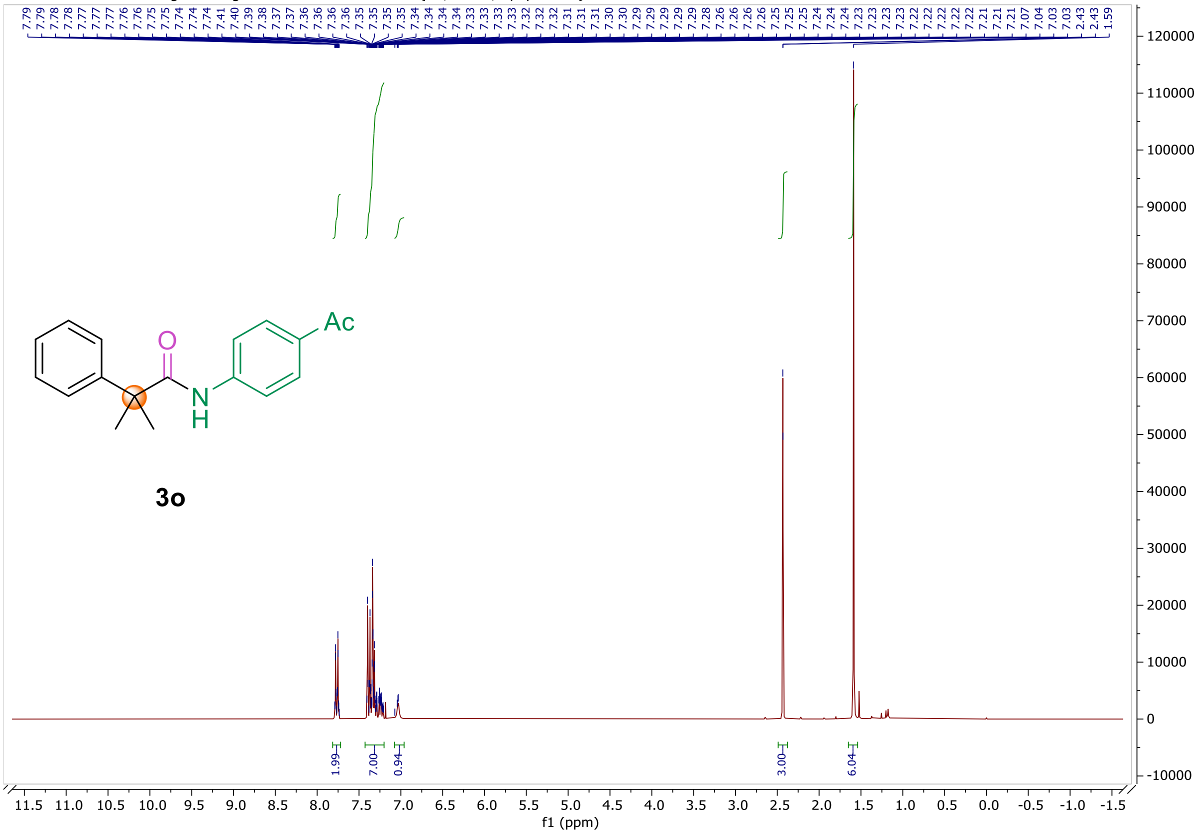


231006.f331.11.fid — Xingwei Gu gxw-185-18 — Au13C CDCl3 {C:\Bruker\TopSpin3.6.2} 2310 31 - 75 MHz

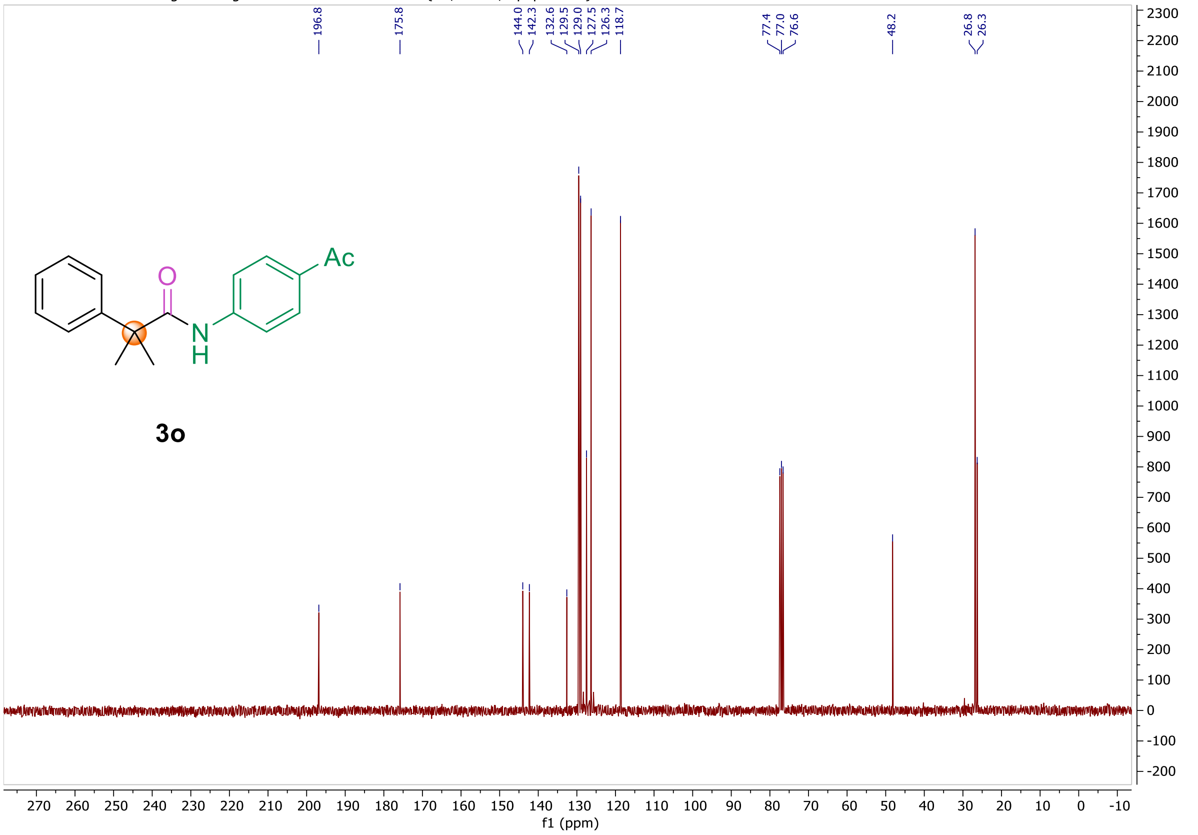

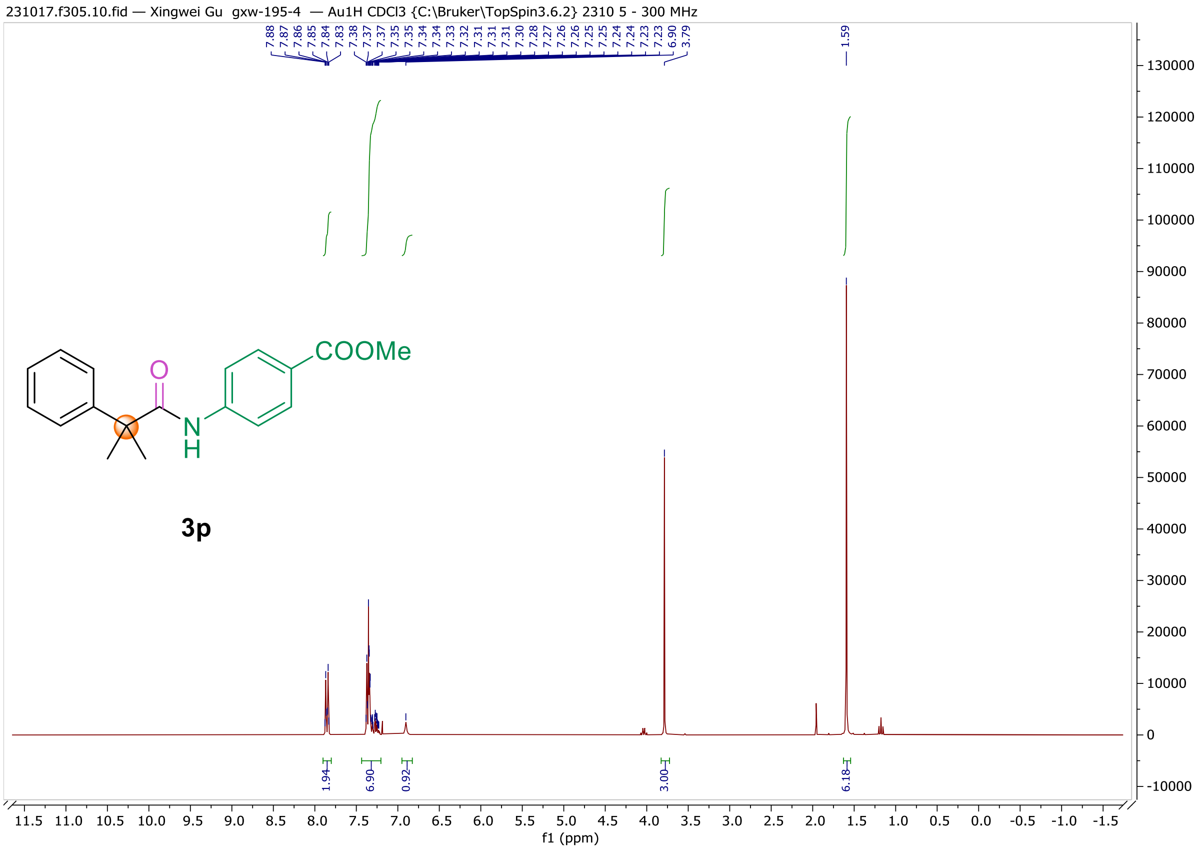
231017.f305.10.fid — Xingwei Gu gxw-195-4 — Au1H CDCl3 {C:\Bruker\TopSpin3.6.2} 2310 5 - 300 MHz
7.88
7.87
7.86
7.85
7.84
7.83
7.38
7.37
7.37
7.35
7.35
7.34
7.34
7.33
7.32
7.31
7.31
7.30
7.28
7.27
7.26
7.26
7.25
7.25
7.24
7.24
7.23
7.23
6.90
3.79
1.59
COOMe
O
N
H
3p
1.94
6.90
0.92
3.00
6.18
f1 (ppm)

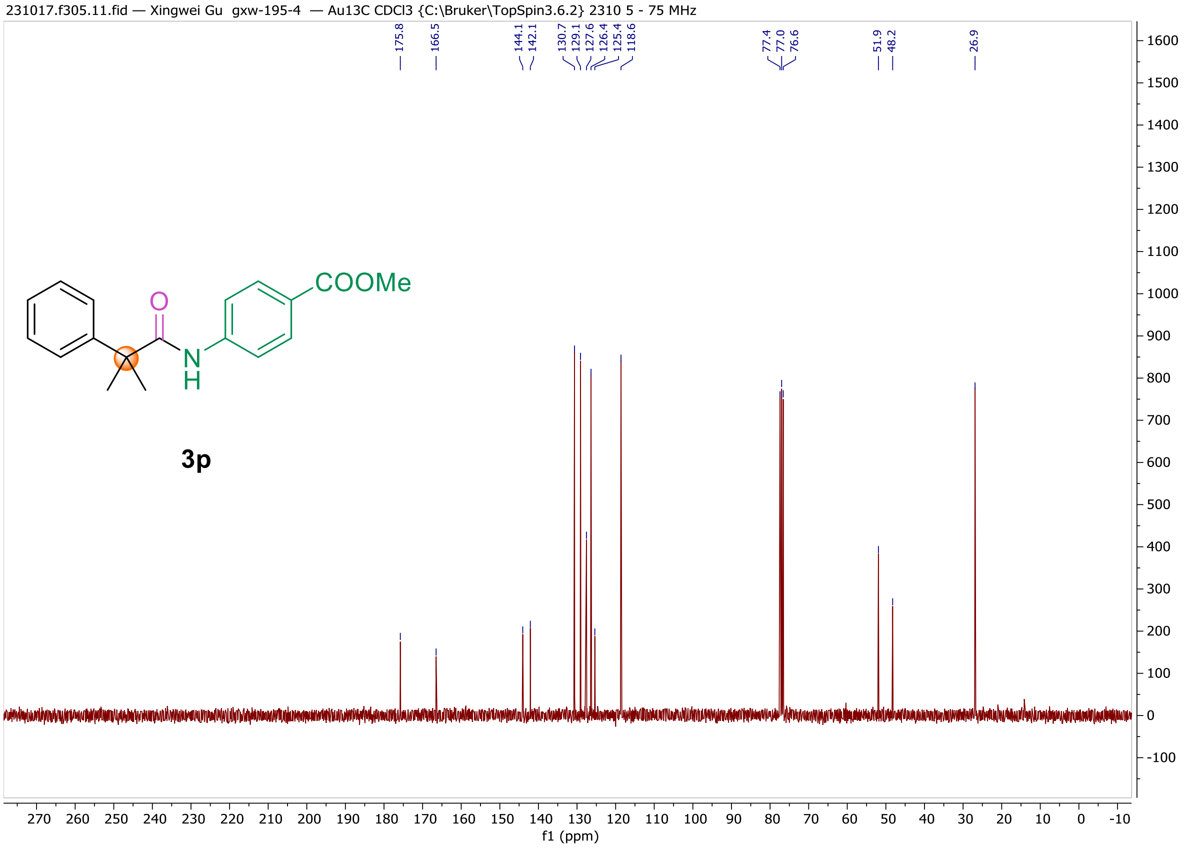
231017.f305.11.fid — Xingwei Gu gxw-195-4 — Au13C CDCl3 {C:\Bruker\TopSpin3.6.2} 2310 5 - 75 MHz
175.8
166.5
144.1
142.1
130.7
129.1
127.6
126.4
125.4
118.6
77.4
77.0
76.6
51.9
48.2
26.9
COOMe
O
N
H
3p
f1 (ppm)

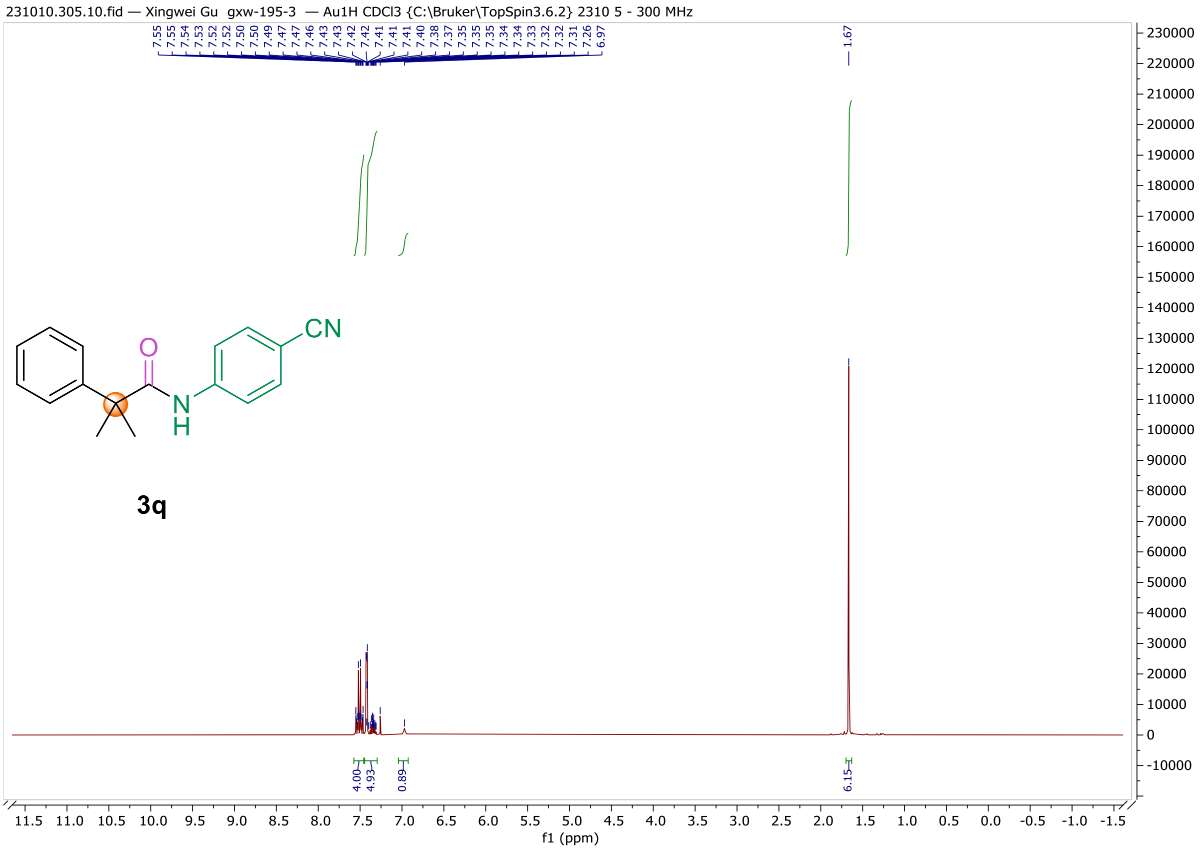
231010.305.10.fid — Xingwei Gu gxw-195-3 — Au1H CDCl3 {C:\Bruker\TopSpin3.6.2} 2310 5 - 300 MHz
7.55 7.55 7.54 7.53 7.52 7.52 7.50 7.50 7.49 7.47 7.47 7.46 7.43 7.43 7.42 7.42 7.41 7.41 7.41 7.40 7.38 7.37 7.35 7.35 7.35 7.34 7.34 7.33 7.32 7.32 7.31 7.26 6.97
1.67
CN
O
N
H
3q
4.00
4.93
0.89
6.15
f1 (ppm)

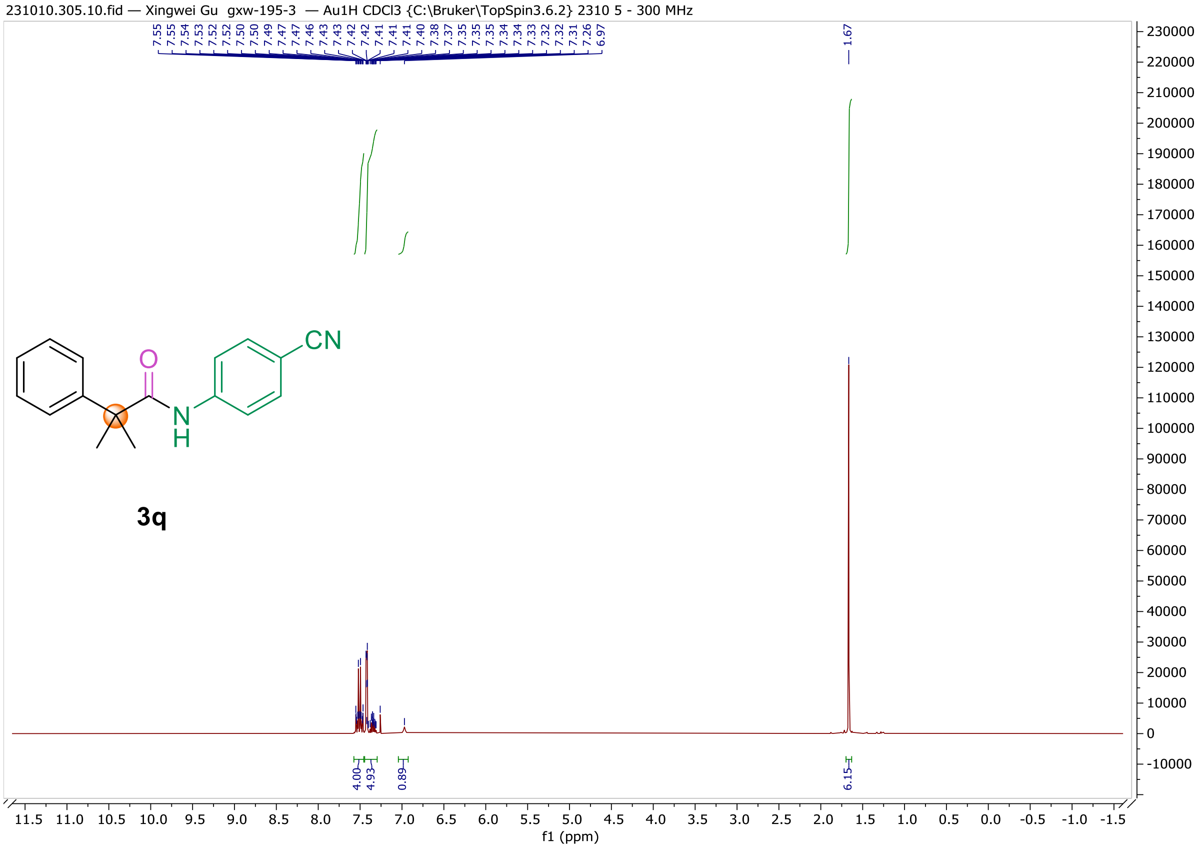
231010.305.10.fid — Xingwei Gu gxw-195-3 — Au1H CDCl3 {C:\Bruker\TopSpin3.6.2} 2310 5 - 300 MHz
7.55 7.55 7.54 7.53 7.52 7.52 7.50 7.50 7.49 7.47 7.47 7.46 7.43 7.43 7.42 7.42 7.41 7.41 7.41 7.40 7.38 7.37 7.35 7.35 7.35 7.34 7.34 7.33 7.32 7.32 7.31 7.26 6.97
1.67
CN
O
N
H
3q
4.00
4.93
0.89
6.15
f1 (ppm)

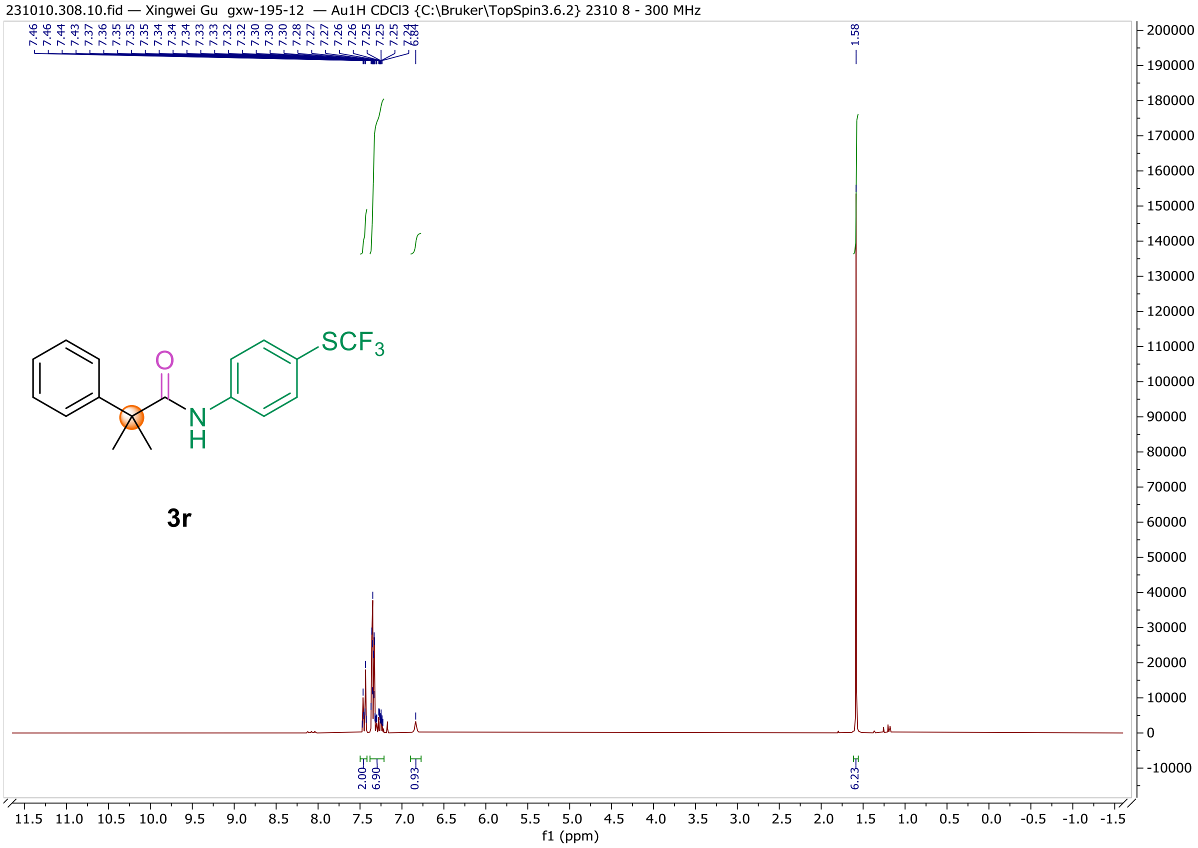
231010.308.10.fid — Xingwei Gu gxw-195-12 — Au1H CDCl3 {C:\Bruker\TopSpin3.6.2} 2310 8 - 300 MHz
3r
f1 (ppm)

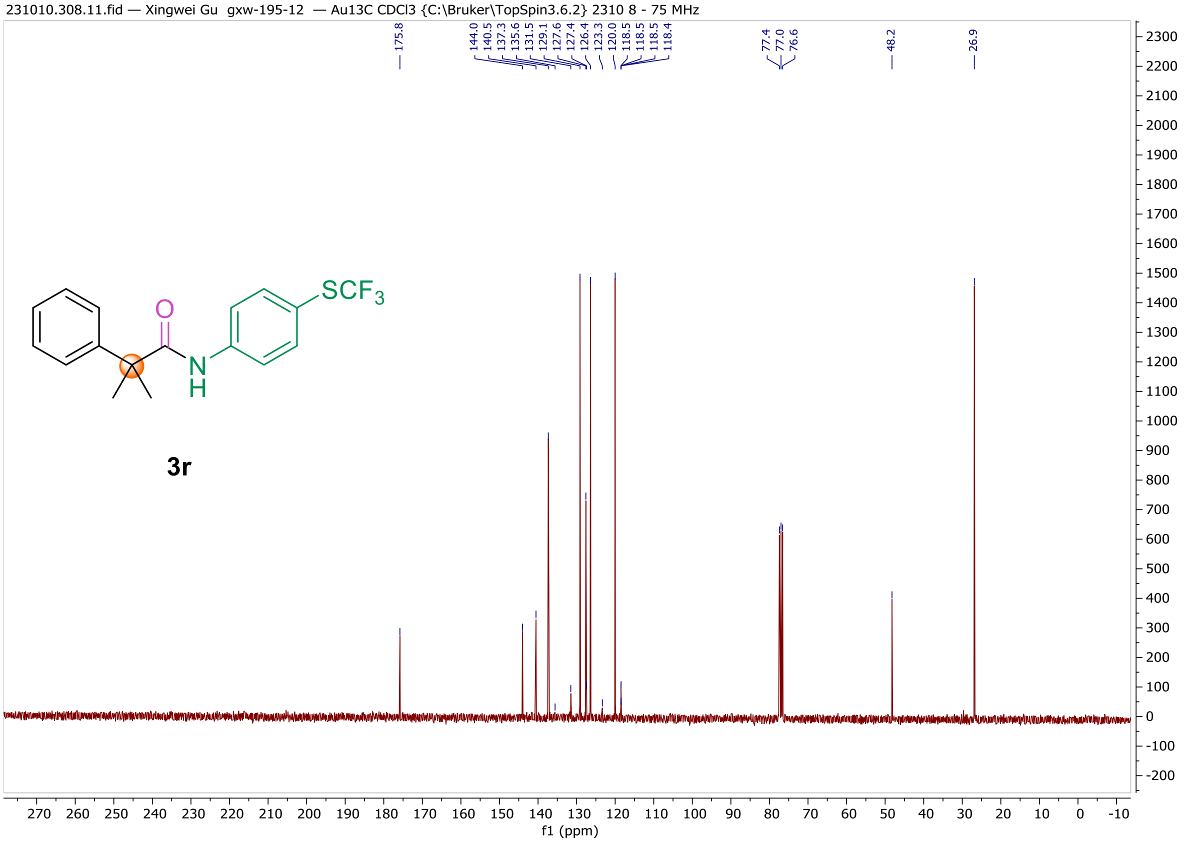
231010.308.11.fid — Xingwei Gu gxw-195-12 — Au13C CDCl3 {C:\Bruker\TopSpin3.6.2} 2310 8 - 75 MHz
175.8
144.0
140.5
137.3
135.6
131.5
129.1
127.6
127.4
126.4
123.3
120.0
118.5
118.5
118.4
77.4
77.0
76.6
48.2
26.9
3r
f1 (ppm)

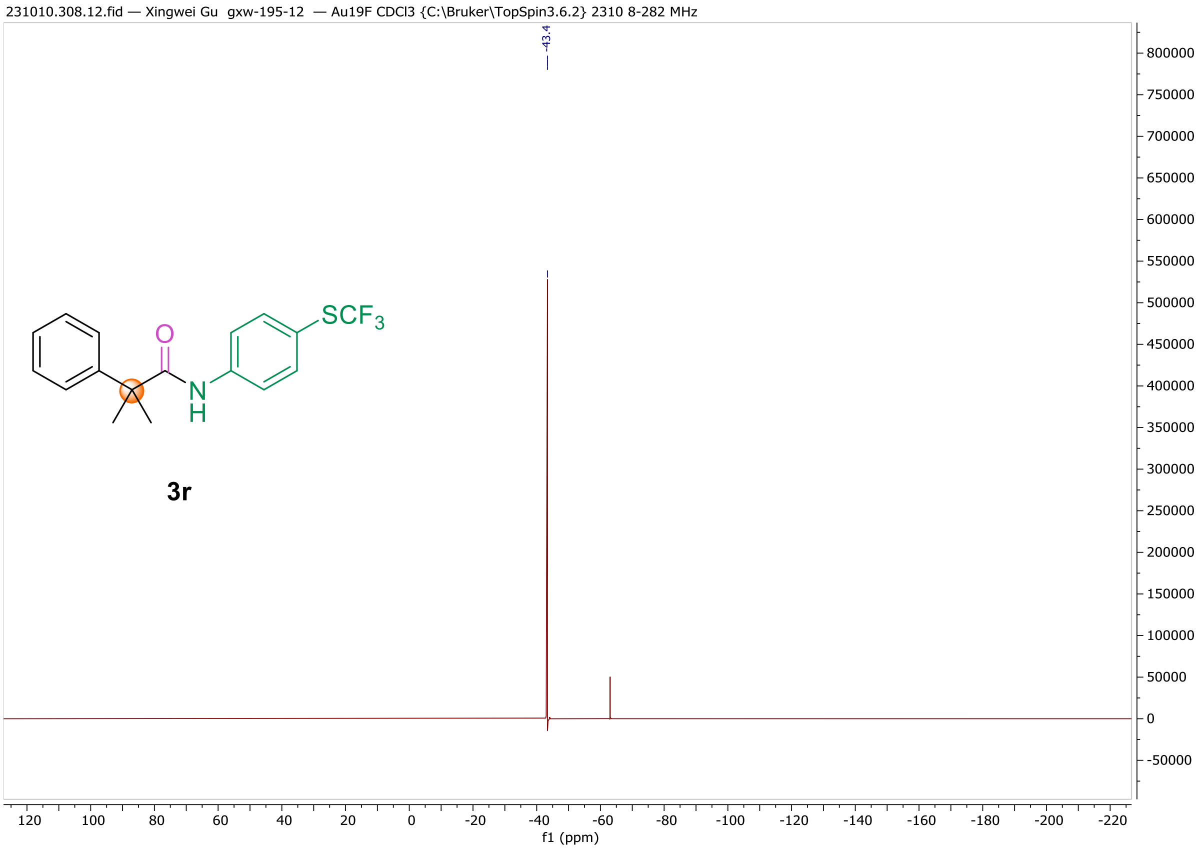
231010.308.12.fid — Xingwei Gu gxw-195-12 — Au19F CDCl3 {C:\Bruker\TopSpin3.6.2} 2310 8-282 MHz
-43.4
SCF3
O
N
H
3r
800000
750000
700000
650000
600000
550000
500000
450000
400000
350000
300000
250000
200000
150000
100000
50000
0
-50000
120 100 80 60 40 20 0 -20 -40 -60 -80 -100 -120 -140 -160 -180 -200 -220
f1 (ppm)

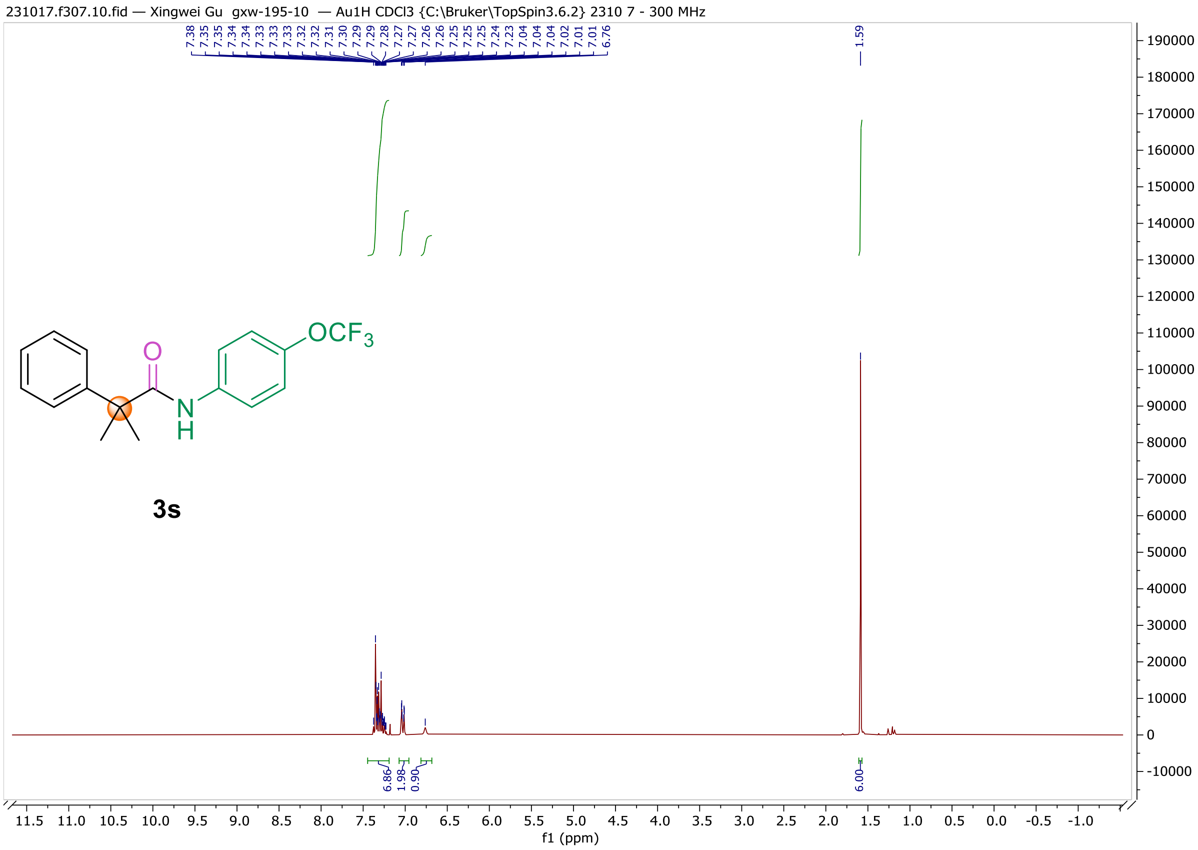
231017.f307.10.fid — Xingwei Gu gxw-195-10 — Au1H CDCl3 {C:\Bruker\TopSpin3.6.2} 2310 7 - 300 MHz
7.38 7.35 7.35 7.34 7.34 7.33 7.33 7.33 7.32 7.32 7.32 7.31 7.30 7.29 7.29 7.28 7.27 7.27 7.26 7.26 7.25 7.25 7.25 7.24 7.23 7.04 7.04 7.04 7.02 7.01 7.01 6.76
1.59
OCF3
O
N
H
3s
6.86
1.98
0.90
6.00
f1 (ppm)

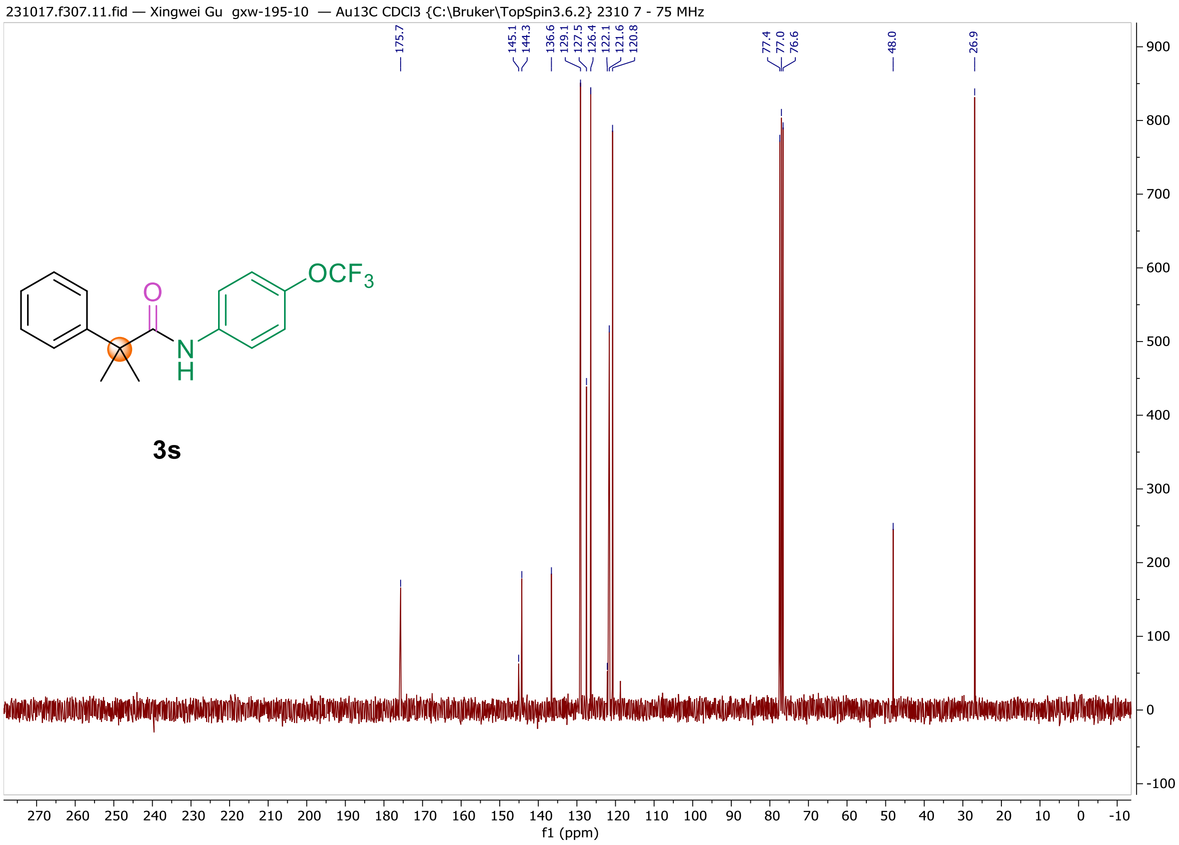
231017.f307.11.fid — Xingwei Gu gxw-195-10 — Au13C CDCl3 {C:\Bruker\TopSpin3.6.2} 2310 7 - 75 MHz
175.7
145.1 144.3
136.6 129.1 127.5 126.4 122.1 121.6 120.8
77.4 77.0 76.6
48.0
26.9
OCF3
O
N
H
3s
f1 (ppm)

231017.f307.12.fid — Xingwei Gu gxw-195-10 — Au19F(H-gek) CDCl3 {C:\Bruker\TopSpin3.6.2} 2310 7-282 MHz

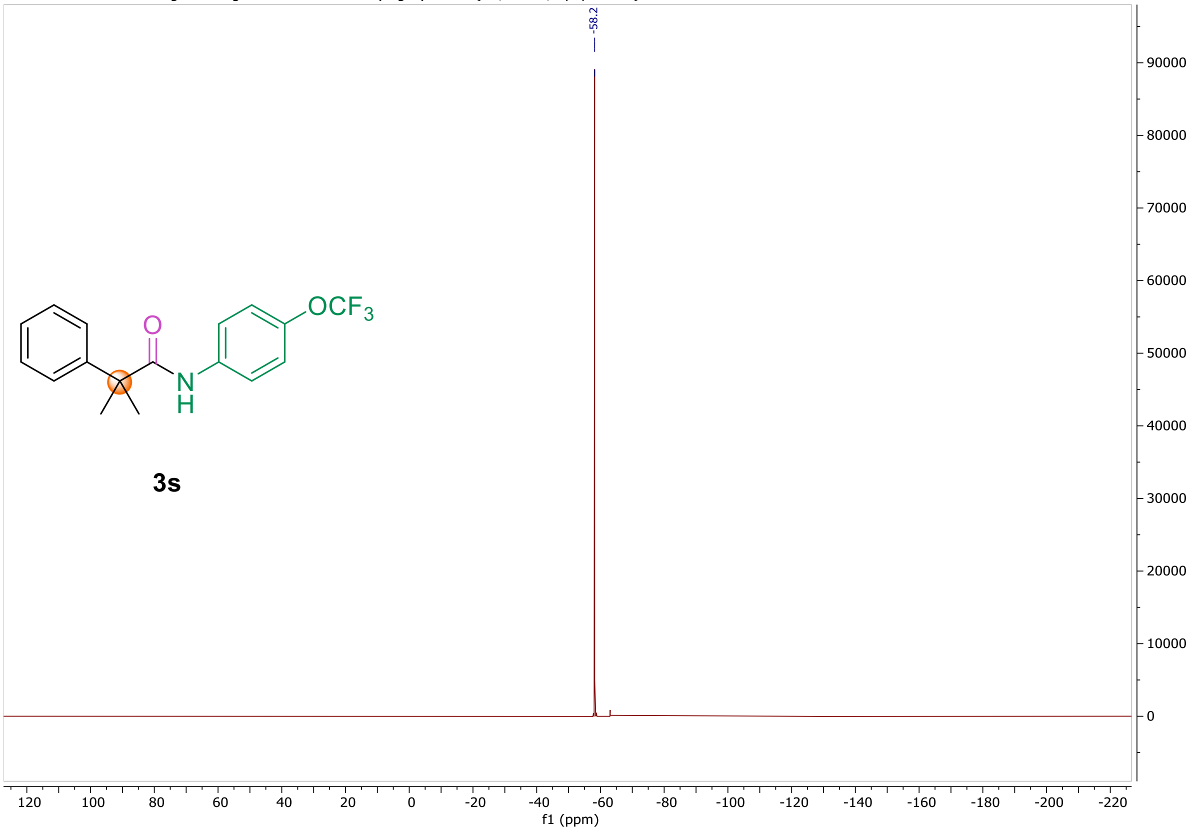


**3s**

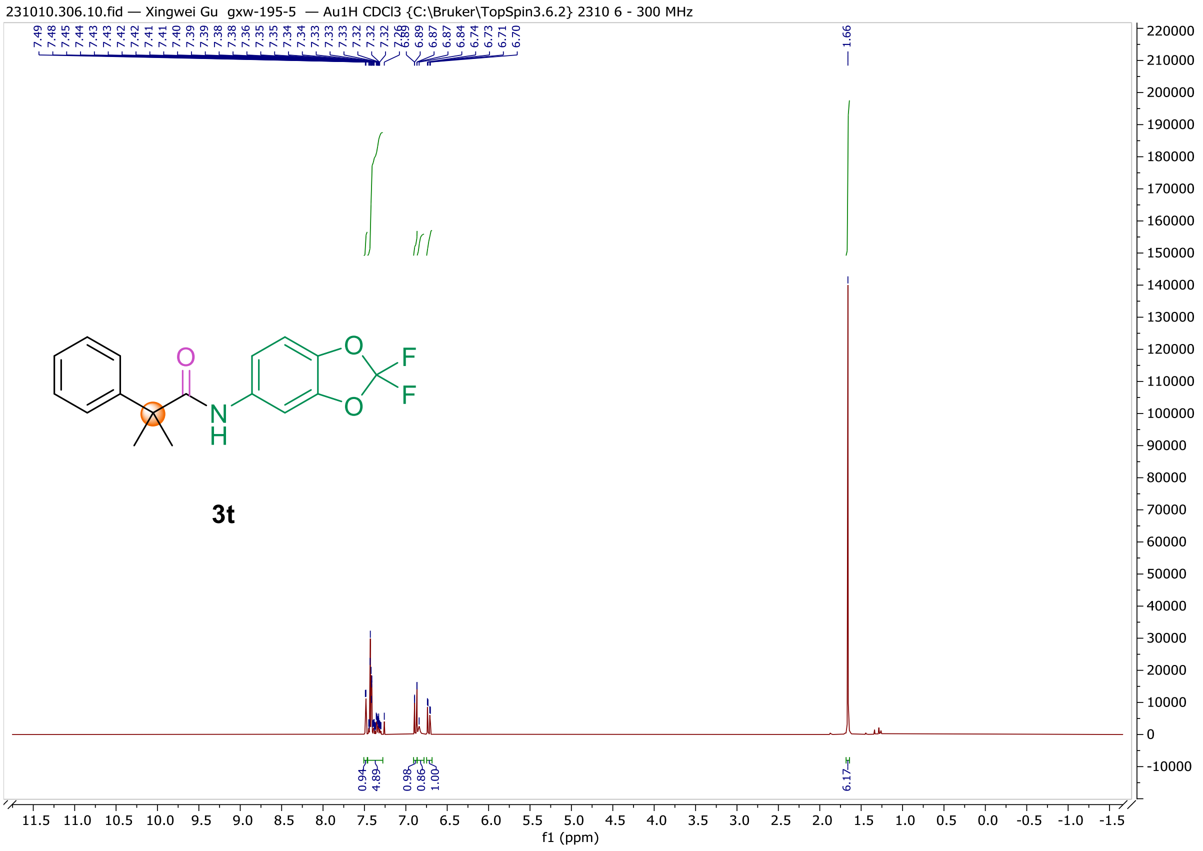
231010.306.10.fid — Xingwei Gu gxw-195-5 — Au1H CDCl3 {C:\Bruker\TopSpin3.6.2} 2310 6 - 300 MHz
7.49 7.48 7.45 7.44 7.43 7.43 7.42 7.42 7.41 7.40 7.39 7.39 7.38 7.38 7.36 7.35 7.35 7.34 7.34 7.33 7.33 7.33 7.32 7.32 7.32 7.26 6.89 6.89 6.87 6.84 6.74 6.73 6.71 6.70
1.66
O
N
H
O
O
F
F
3t
0.94 4.89 0.98 0.86 1.00
6.17
f1 (ppm)

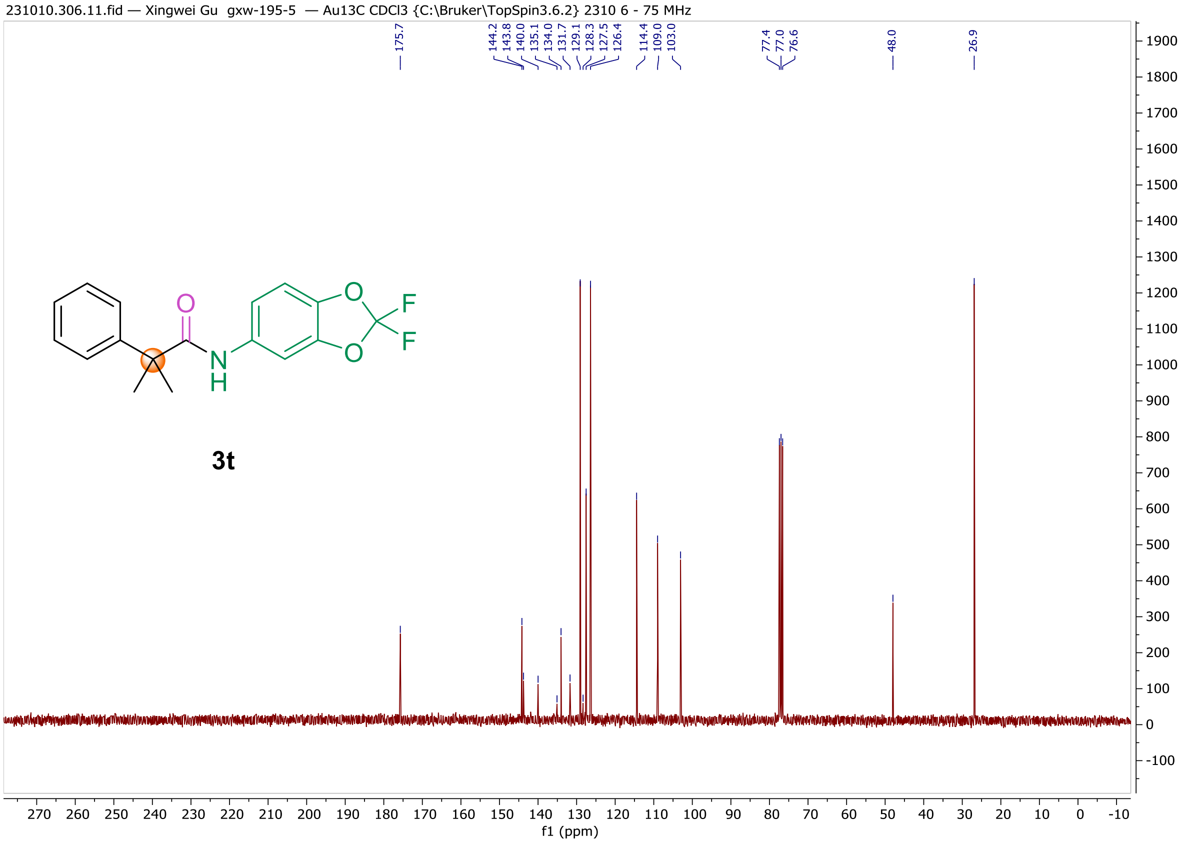
231010.306.11.fid — Xingwei Gu gxw-195-5 — Au13C CDCl3 {C:\Bruker\TopSpin3.6.2} 2310 6 - 75 MHz
175.7
144.2 143.8 140.0 135.1 134.0 131.7 129.1 128.3 127.5 126.4
114.4 109.0 103.0
77.4 77.0 76.6
48.0
26.9
3t
f1 (ppm)

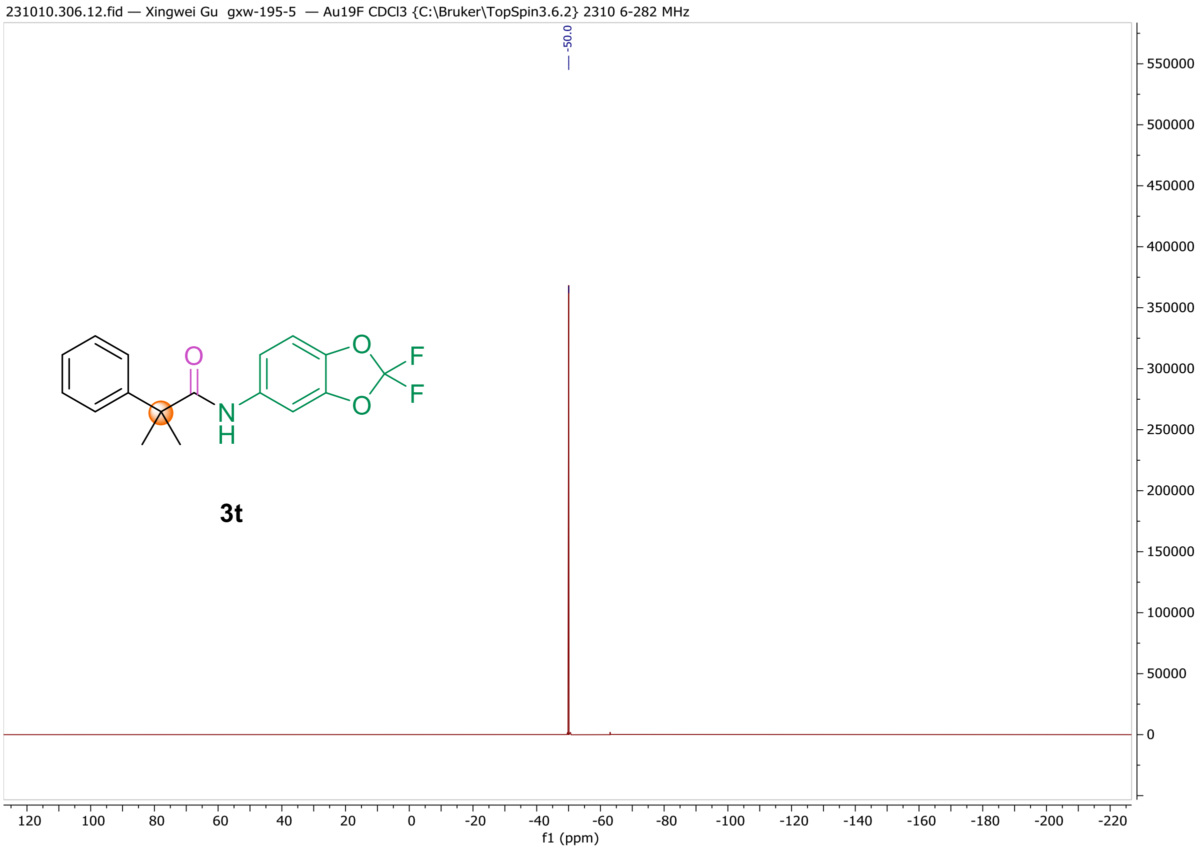
231010.306.12.fid — Xingwei Gu gxw-195-5 — Au19F CDCl3 {C:\Bruker\TopSpin3.6.2} 2310 6-282 MHz
-50.0
O
N
H
O
O
F
F
3t
550000
500000
450000
400000
350000
300000
250000
200000
150000
100000
50000
0
120 100 80 60 40 20 0 -20 -40 -60 -80 -100 -120 -140 -160 -180 -200 -220
f1 (ppm)

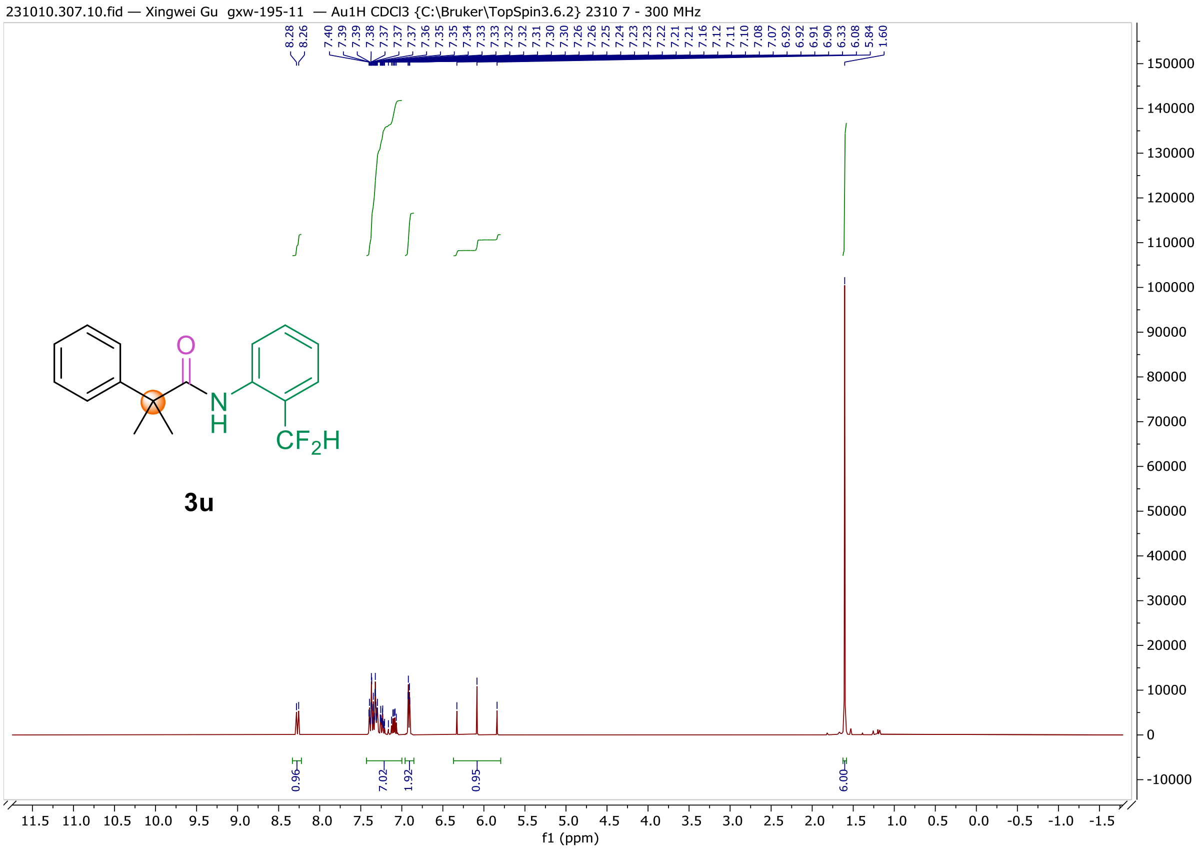
231010.307.10.fid — Xingwei Gu gxw-195-11 — Au1H CDCl3 {C:\Bruker\TopSpin3.6.2} 2310 7 - 300 MHz
8.28
8.26
7.40
7.39
7.39
7.38
7.37
7.37
7.37
7.36
7.35
7.35
7.34
7.33
7.33
7.33
7.32
7.32
7.31
7.30
7.30
7.26
7.26
7.25
7.24
7.23
7.23
7.22
7.21
7.21
7.16
7.12
7.11
7.10
7.08
7.07
6.92
6.92
6.91
6.90
6.33
6.08
5.84
1.60
O
N
H
$CF_2H$
3u
0.96
7.02
1.92
0.95
6.00
f1 (ppm)

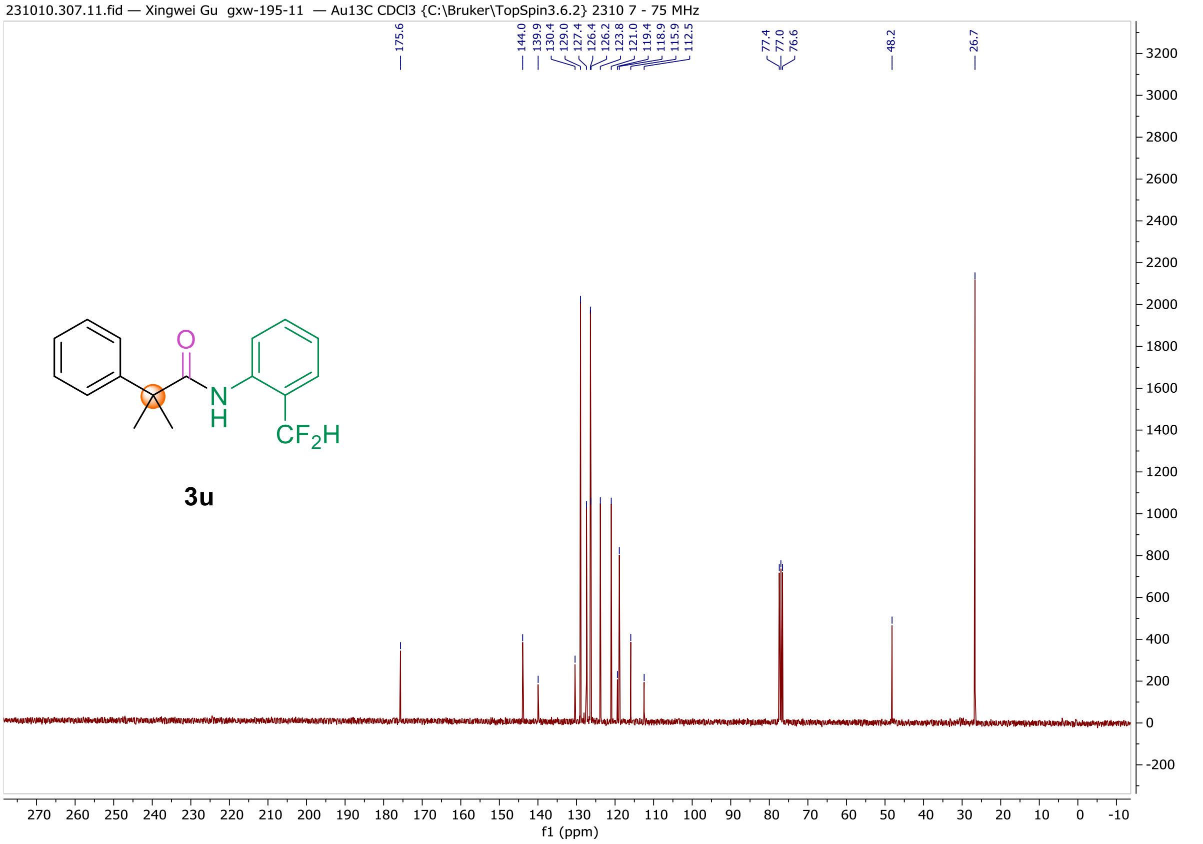
231010.307.11.fid — Xingwei Gu gxw-195-11 — Au13C CDCl3 {C:\Bruker\TopSpin3.6.2} 2310 7 - 75 MHz
175.6
144.0
139.9
130.4
129.0
127.4
126.4
126.2
123.8
121.0
119.4
118.9
115.9
112.5
77.4
77.0
76.6
48.2
26.7
O
N
H
$CF_2H$
3u
f1 (ppm)

231010.307.12.fid — Xingwei Gu gxw-195-11 — Au19F CDCl3 {C:\Bruker\TopSpin3.6.2} 2310 7-282 MHz

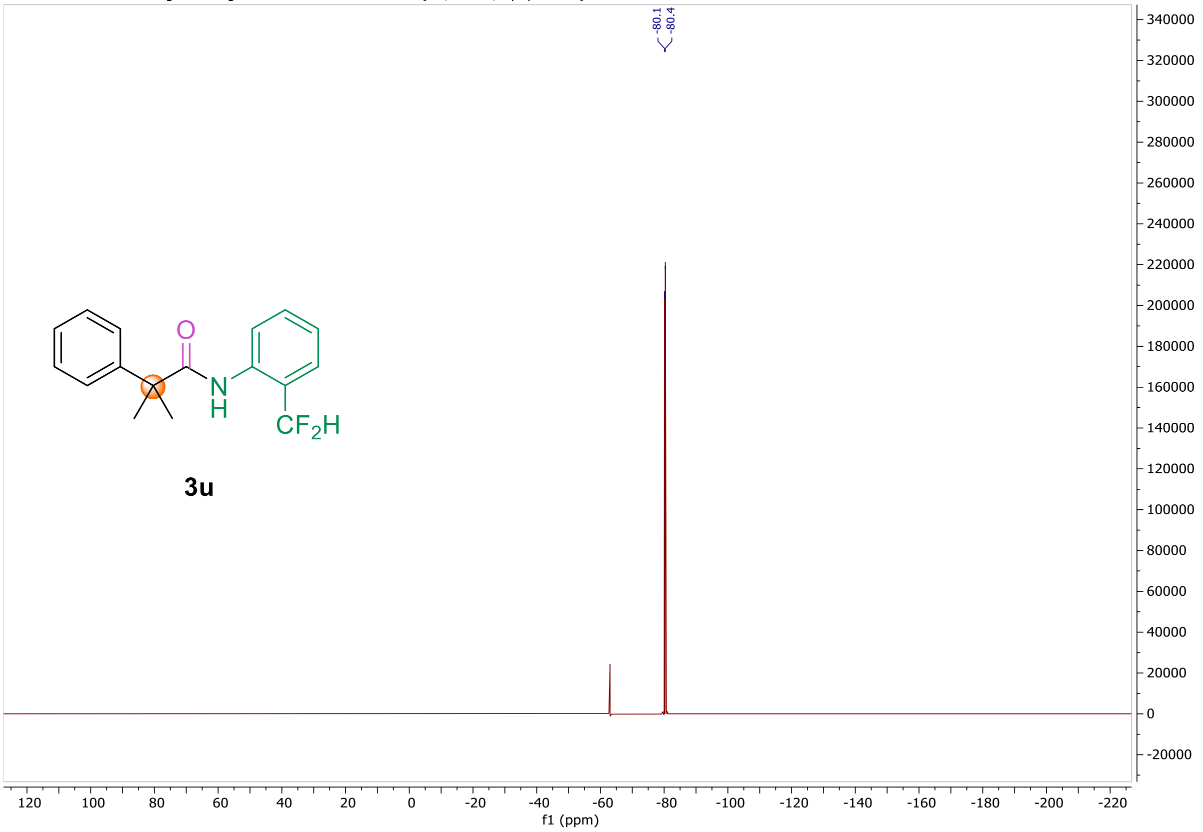


**3u**

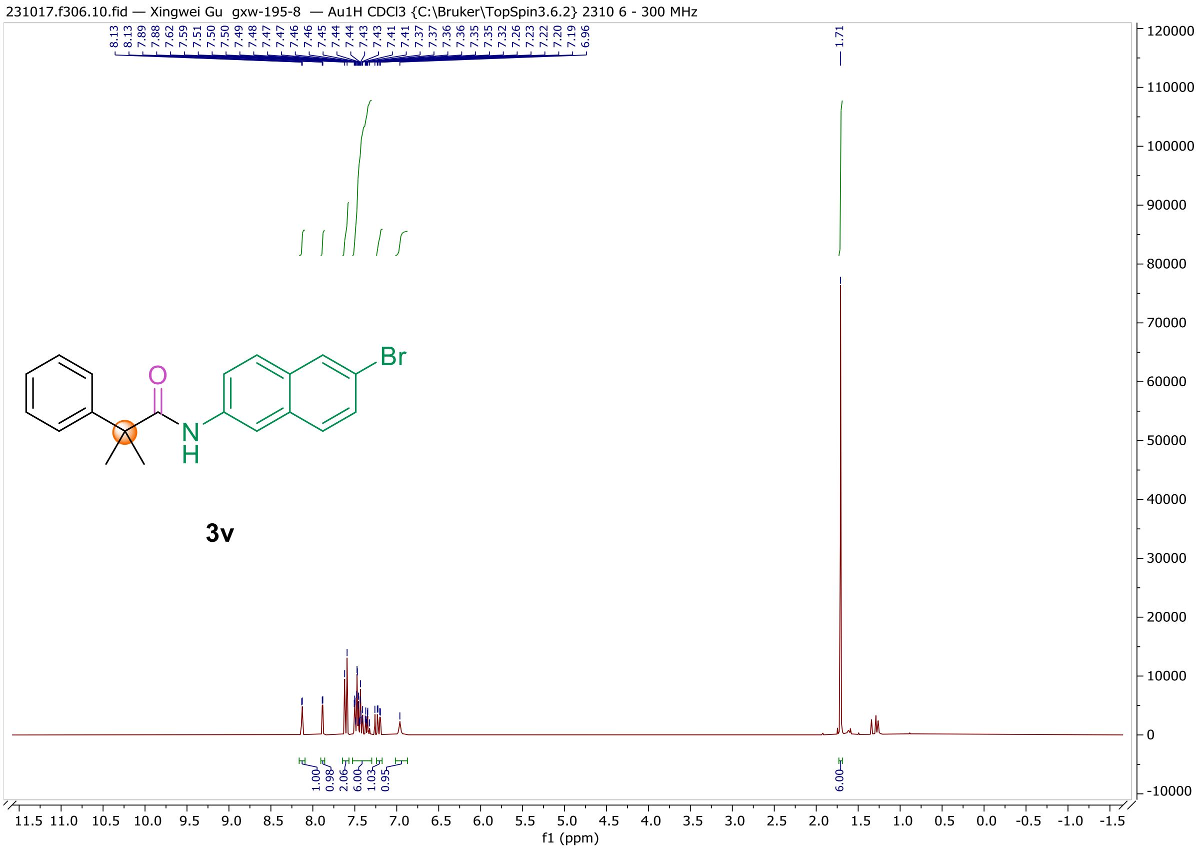
231017.f306.10.fid — Xingwei Gu gxw-195-8 — Au1H CDCl3 {C:\Bruker\TopSpin3.6.2} 2310 6 - 300 MHz
8.13 8.13 7.89 7.88 7.62 7.59 7.51 7.50 7.49 7.48 7.47 7.47 7.46 7.46 7.45 7.44 7.44 7.43 7.43 7.41 7.41 7.37 7.37 7.36 7.36 7.35 7.35 7.32 7.26 7.23 7.22 7.20 7.19 6.96
1.71
Br
O
N
H
3v
1.00 0.98 2.06 6.00 1.03 0.95
6.00
f1 (ppm)

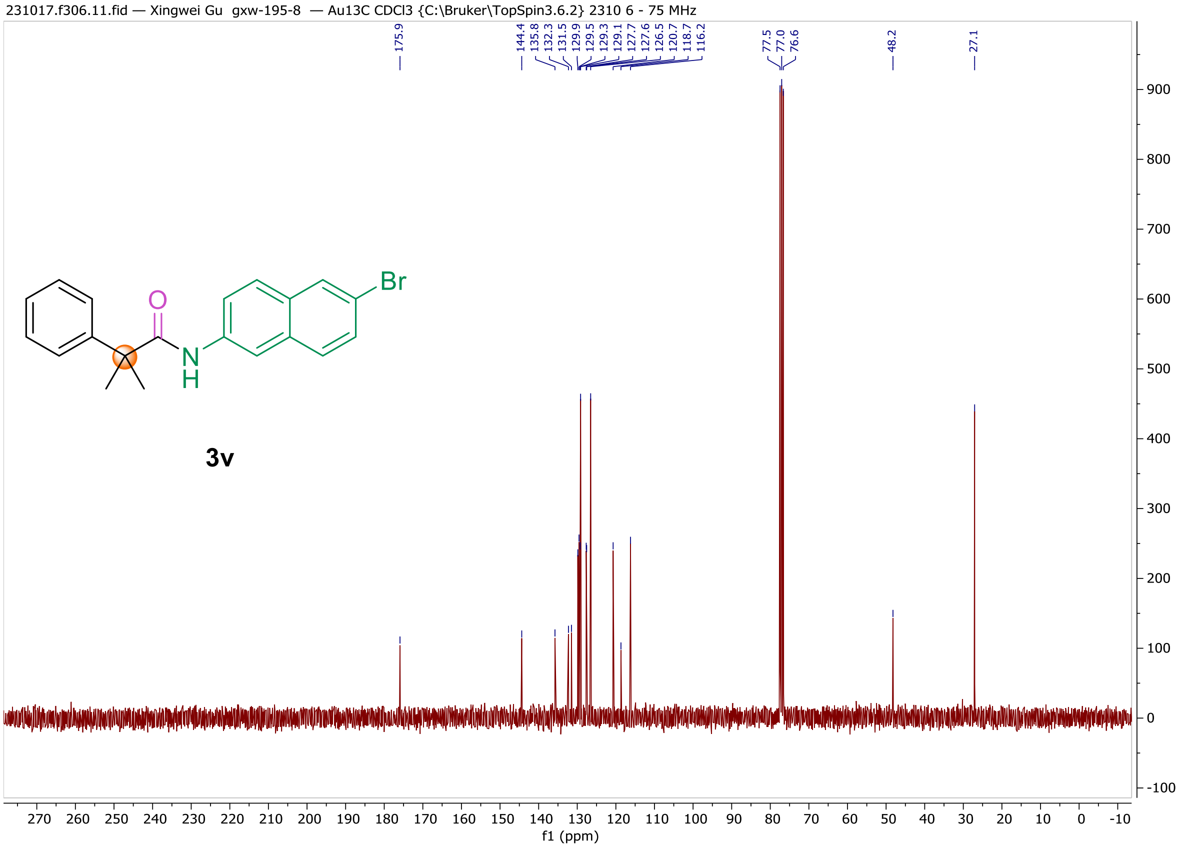
231017.f306.11.fid — Xingwei Gu gxw-195-8 — Au13C CDCl3 {C:\Bruker\TopSpin3.6.2} 2310 6 - 75 MHz
175.9
144.4 135.8 132.3 131.5 129.9 129.5 129.3 129.1 127.7 127.6 126.5 120.7 118.7 116.2
77.5 77.0 76.6
48.2
27.1
Br
O
N
H
3v
f1 (ppm)

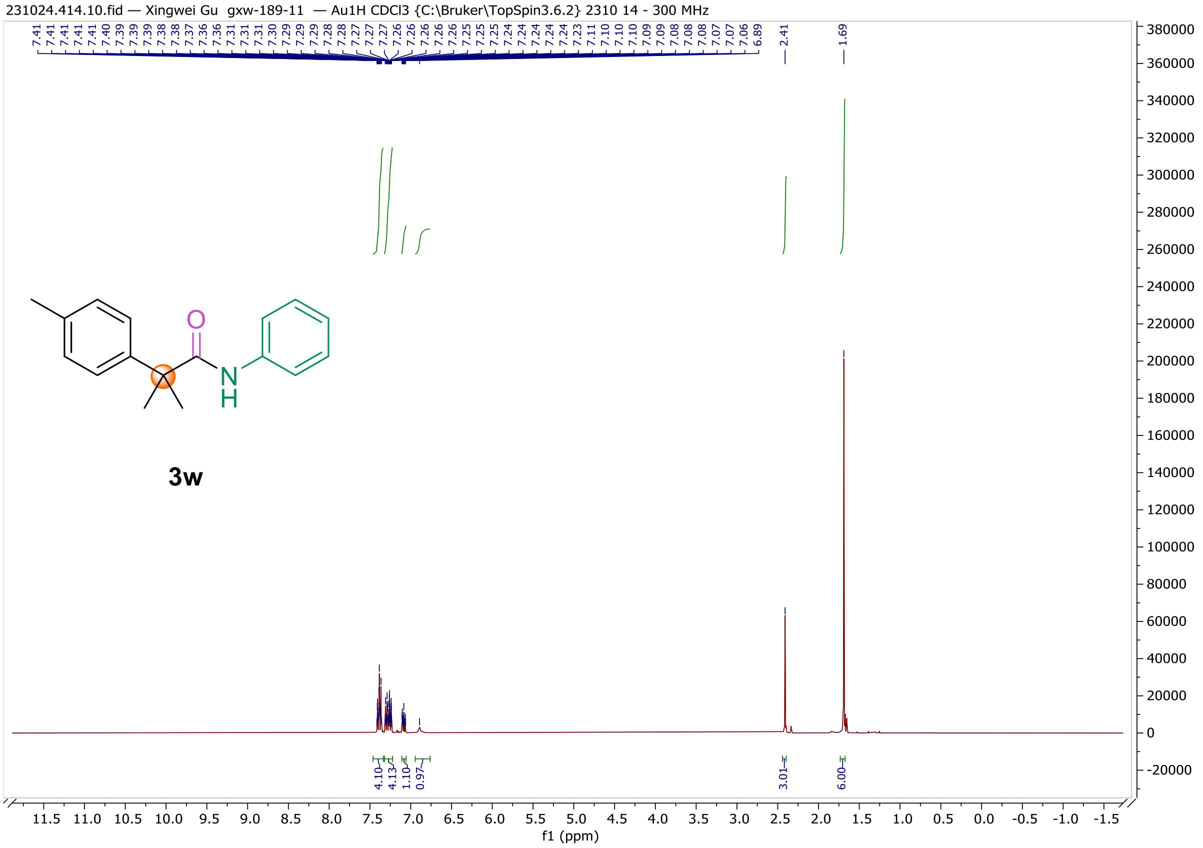
231024.414.10.fid — Xingwei Gu gxw-189-11 — Au1H CDCl3 {C:\Bruker\TopSpin3.6.2} 2310 14 - 300 MHz
3w
f1 (ppm)

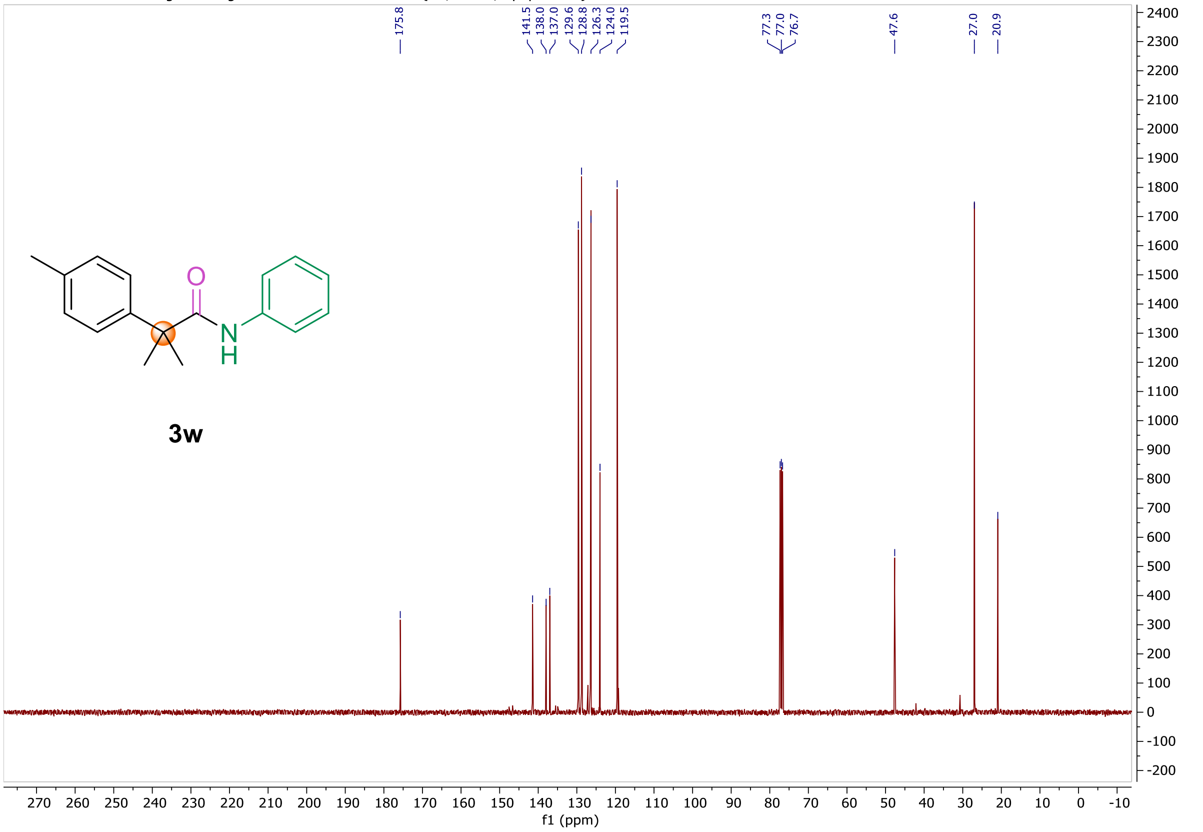
231024.414.11.fid — Xingwei Gu gxw-189-11 — Au13C CDCl3 {C:\Bruker\TopSpin3.6.2} 2310 14 - 75 MHz
175.8
141.5
138.0
137.0
129.6
128.8
126.3
124.0
119.5
77.3
77.0
76.7
47.6
27.0
20.9
3w
f1 (ppm)

231024.415.10.fid — Xingwei Gu gxw-189-12 — Au1H CDCl3 {C:\Bruker\TopSpin3.6.2} 2310 15 - 300 MHz

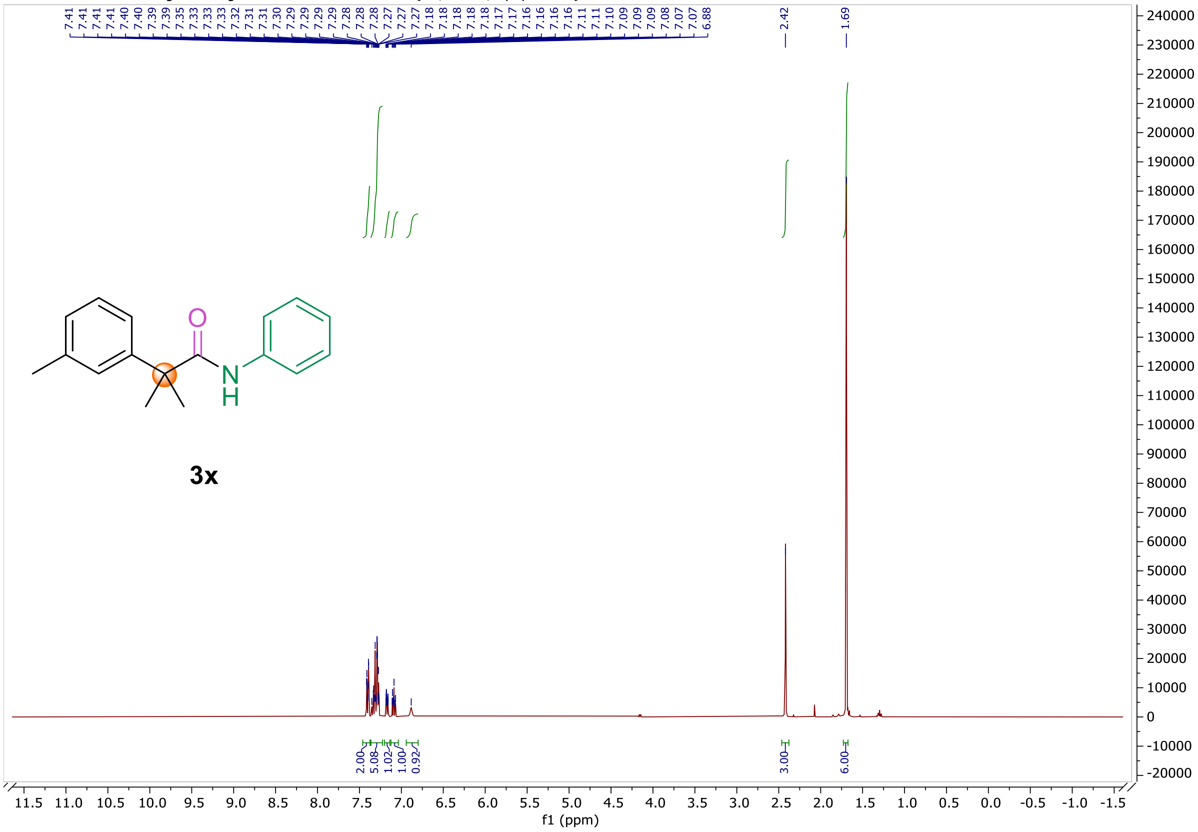


231024.415.11.fid — Xingwei Gu gxw-189-12 — Au13C CDCl3 {C:\Bruker\TopSpin3.6.2} 2310 15 - 75 MHz

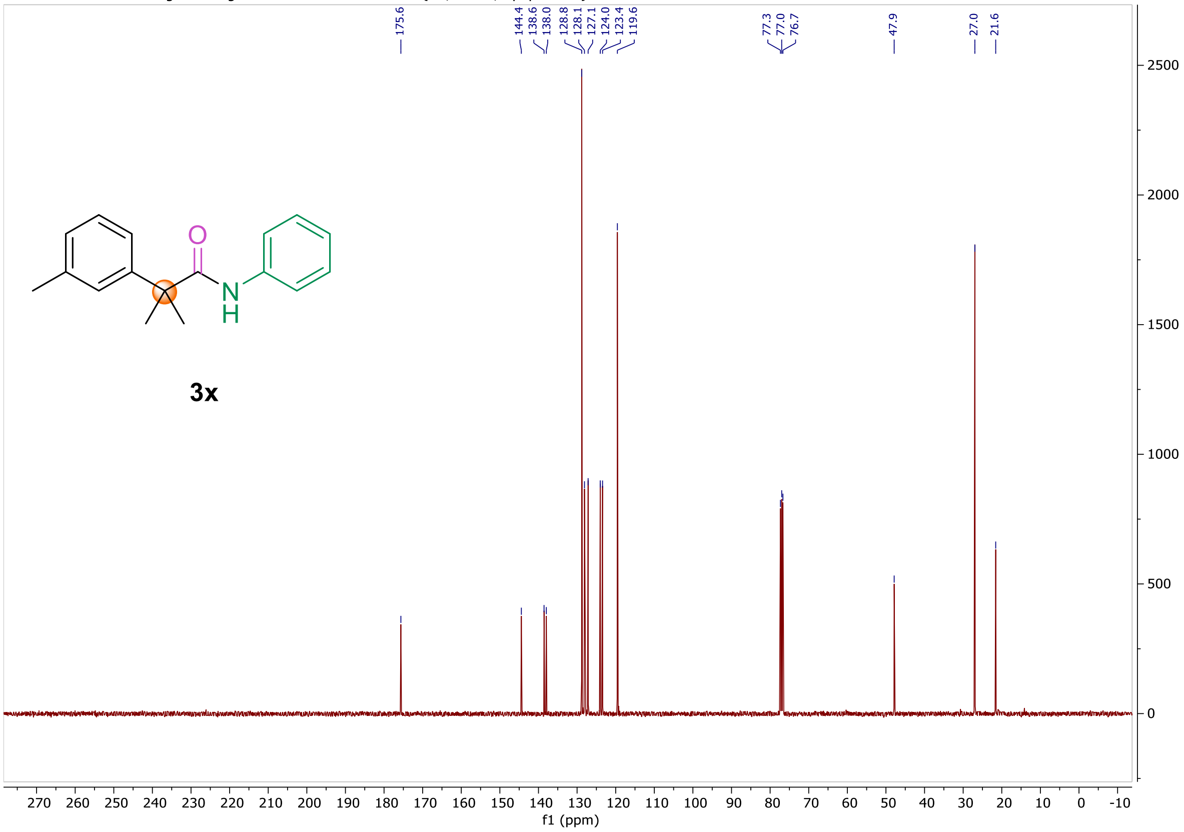

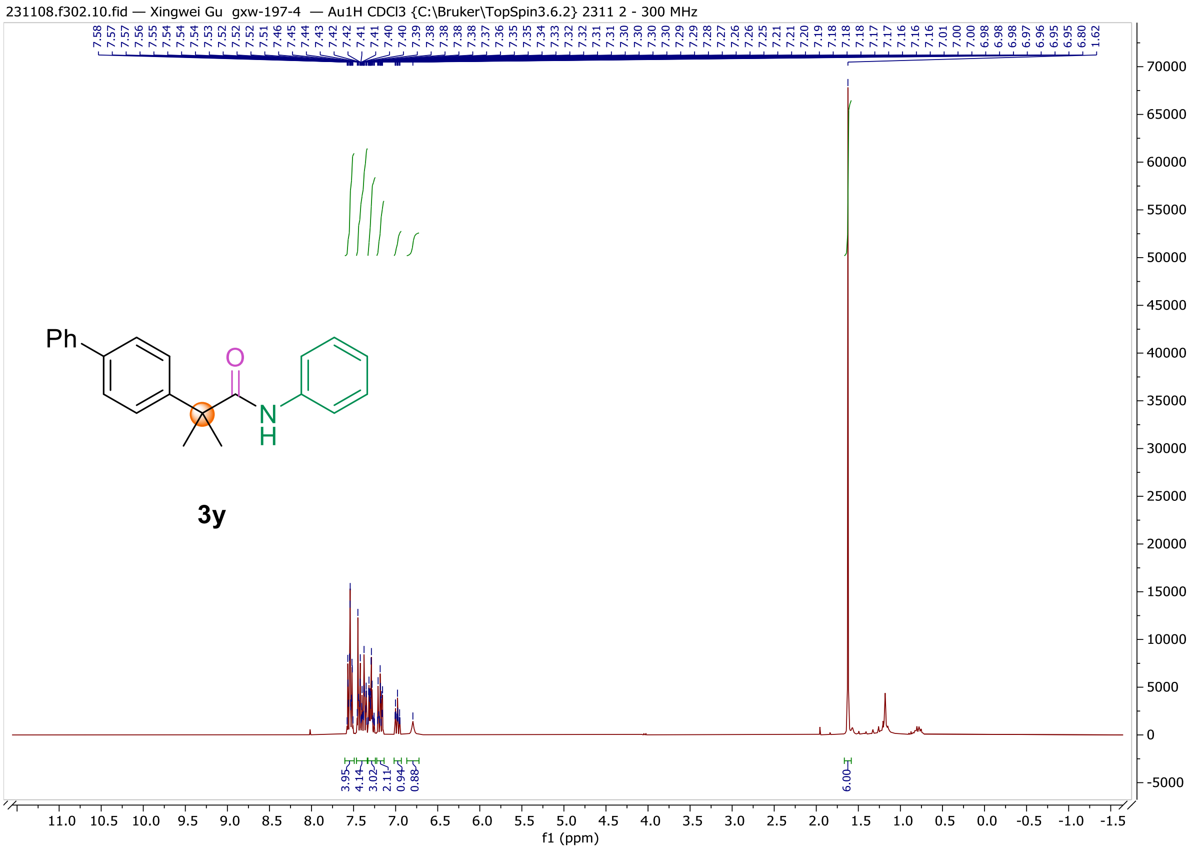
231108.f302.10.fid — Xingwei Gu gxw-197-4 — Au1H CDCl3 {C:\Bruker\TopSpin3.6.2} 2311 2 - 300 MHz
Ph
O
N
H
3y
3.95
4.14
3.02
2.11
0.94
0.88
6.00
f1 (ppm)

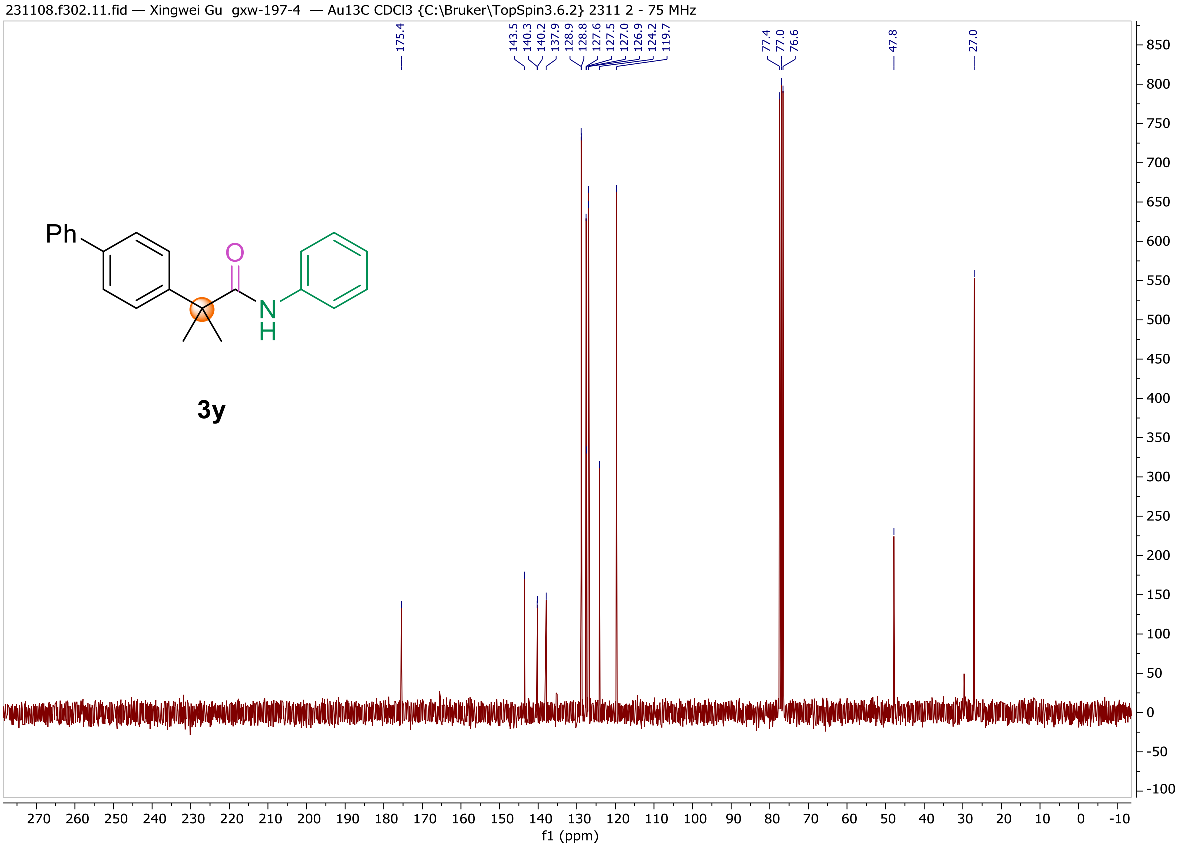
231108.f302.11.fid — Xingwei Gu gxw-197-4 — Au13C CDCl3 {C:\Bruker\TopSpin3.6.2} 2311 2 - 75 MHz
175.4
143.5
140.3
140.2
137.9
128.9
128.8
127.6
127.5
127.0
126.9
124.2
119.7
77.4
77.0
76.6
47.8
27.0
Ph
O
N
H
3y
f1 (ppm)

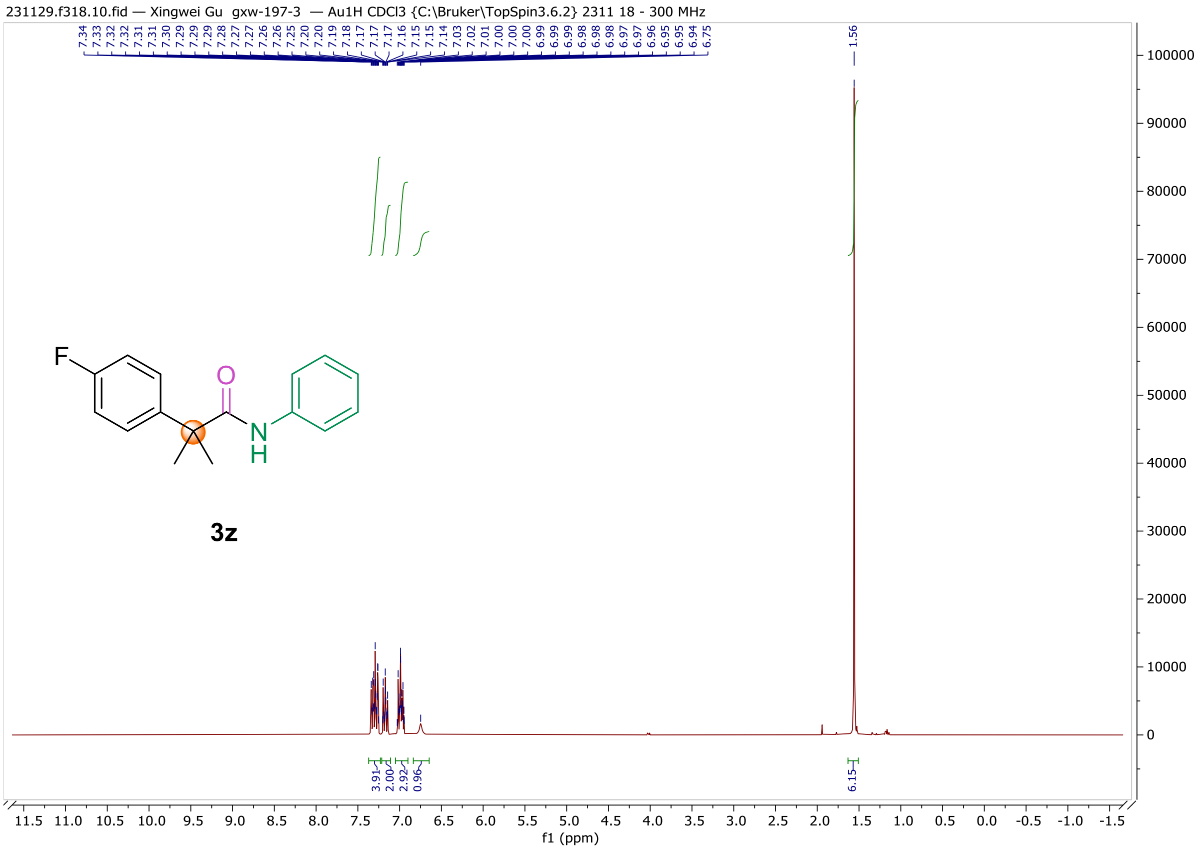
231129.f318.10.fid — Xingwei Gu gxw-197-3 — Au1H CDCl3 {C:\Bruker\TopSpin3.6.2} 2311 18 - 300 MHz
7.34 7.33 7.32 7.32 7.31 7.31 7.30 7.29 7.29 7.29 7.28 7.27 7.27 7.26 7.26 7.25 7.20 7.20 7.19 7.18 7.17 7.17 7.17 7.16 7.15 7.15 7.14 7.03 7.02 7.01 7.00 7.00 7.00 6.99 6.99 6.99 6.98 6.98 6.98 6.97 6.97 6.96 6.95 6.95 6.94 6.75
1.56
3.91
2.00
2.92
0.96
6.15
F
O
N
H
3z
f1 (ppm)

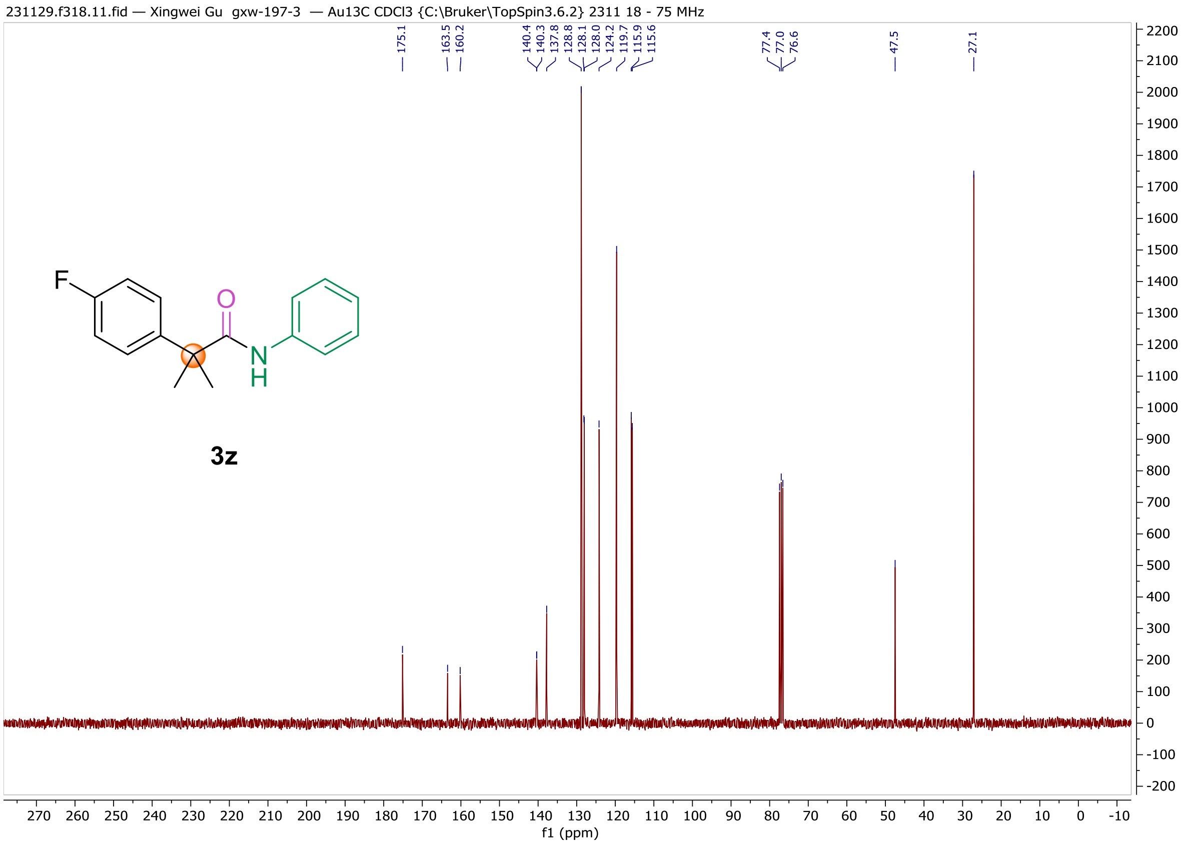
231129.f318.11.fid — Xingwei Gu gxw-197-3 — Au13C CDCl3 {C:\Bruker\TopSpin3.6.2} 2311 18 - 75 MHz
175.1
163.5 160.2
140.4 140.3 137.8 128.8 128.1 128.0 124.2 119.7 115.9 115.6
77.4 77.0 76.6
47.5
27.1
F
O
N
H
3z
f1 (ppm)

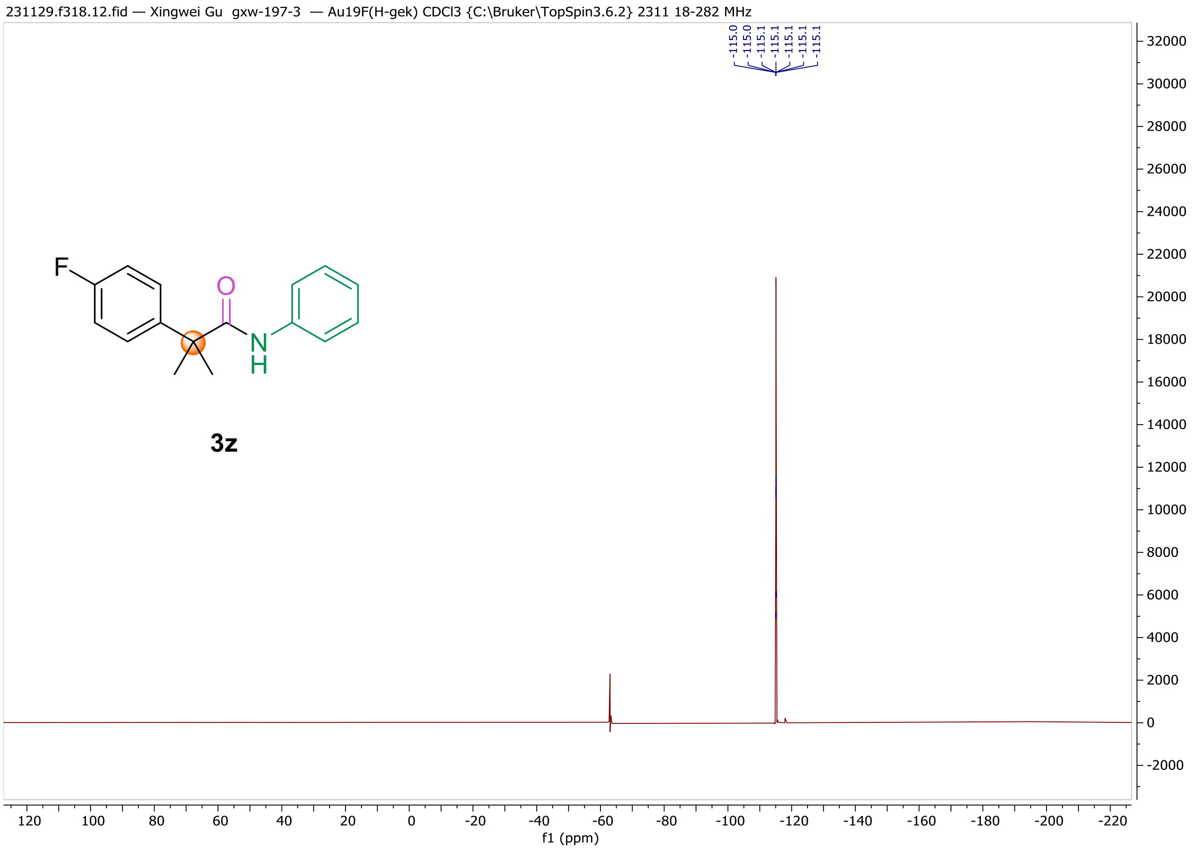

231129.f318.12.fid — Xingwei Gu gxw-197-3 — Au19F(H-gek) CDCl3 {C:\Bruker\TopSpin3.6.2} 2311 18-282 MHz
-115.0
-115.0
-115.1
-115.1
-115.1
-115.1
-115.1
F
O
N
H
32000
30000
28000
26000
24000
22000
20000
18000
16000
14000
12000
10000
8000
6000
4000
2000
0
-2000
120 100 80 60 40 20 0 -20 -40 -60 -80 -100 -120 -140 -160 -180 -200 -220
f1 (ppm)


3z

230926.320.10.fid — Xingwei Gu, gxw-189-7 — Au1H CDCl3 {C:\Bruker\TopSpin3.6.2} 2309 20 - 300 MHz

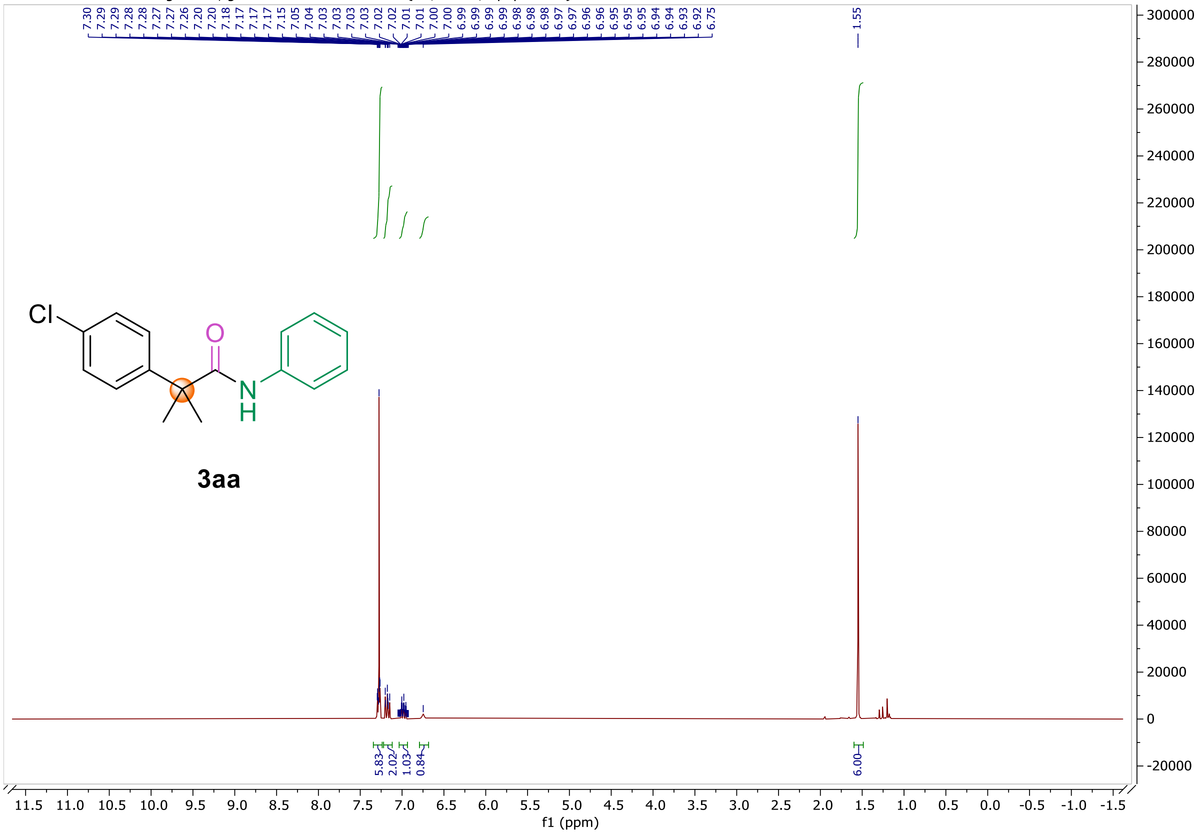


230926.320.11.fid — Xingwei Gu, gxw-189-7 — Au13C CDCl3 {C:\Bruker\TopSpin3.6.2} 2309 20 - 75 MHz

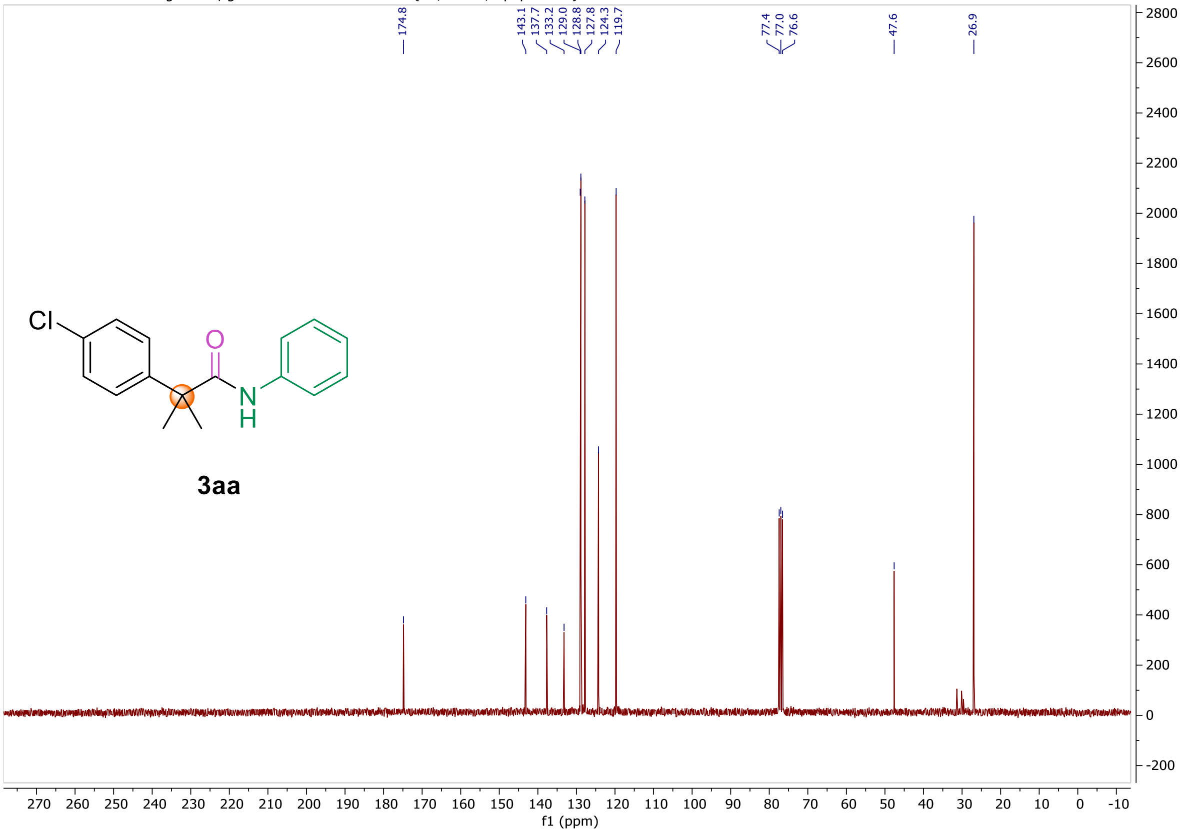

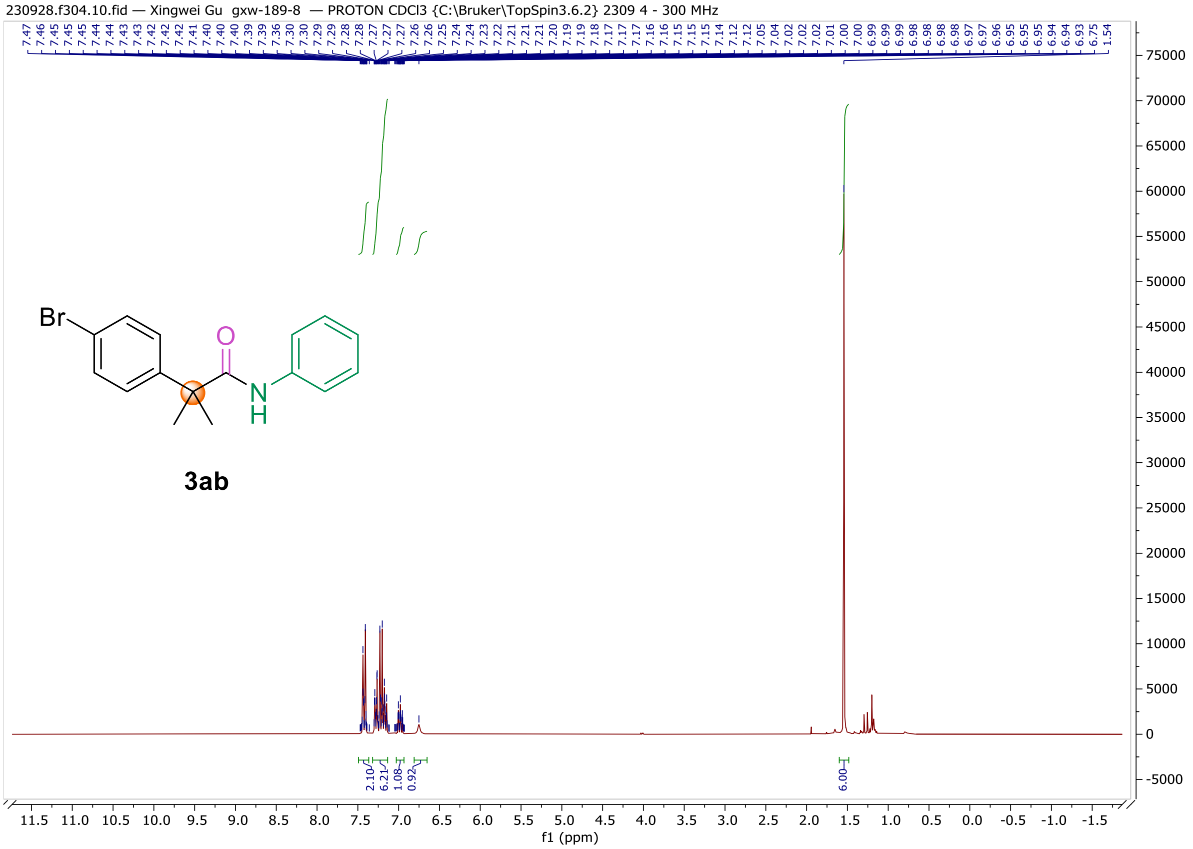
230928.f304.10.fid — Xingwei Gu gxw-189-8 — PROTON CDCl3 {C:\Bruker\TopSpin3.6.2} 2309 4 - 300 MHz
3ab
f1 (ppm)

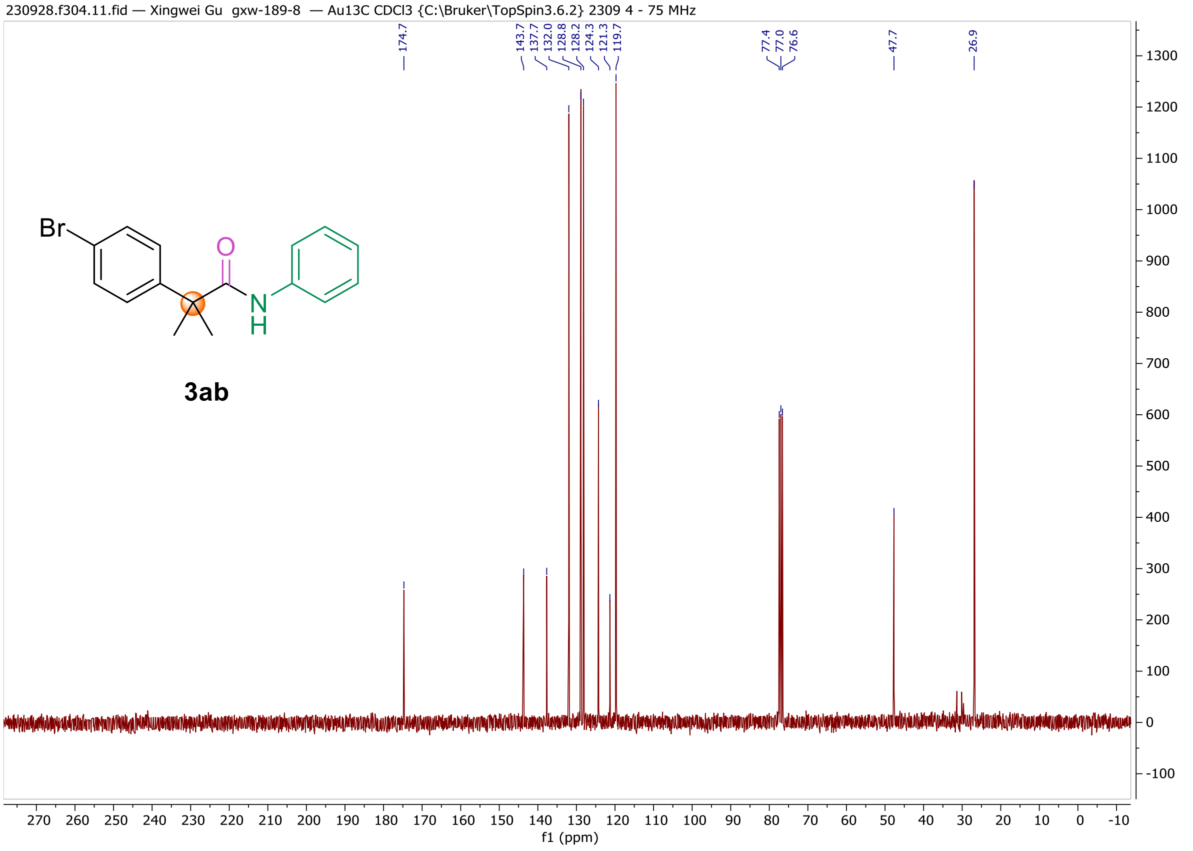
230928.f304.11.fid — Xingwei Gu gxw-189-8 — Au13C CDCl3 {C:\Bruker\TopSpin3.6.2} 2309 4 - 75 MHz
174.7
143.7
137.7
132.0
128.8
128.2
124.3
121.3
119.7
77.4
77.0
76.6
47.7
26.9
3ab
f1 (ppm)

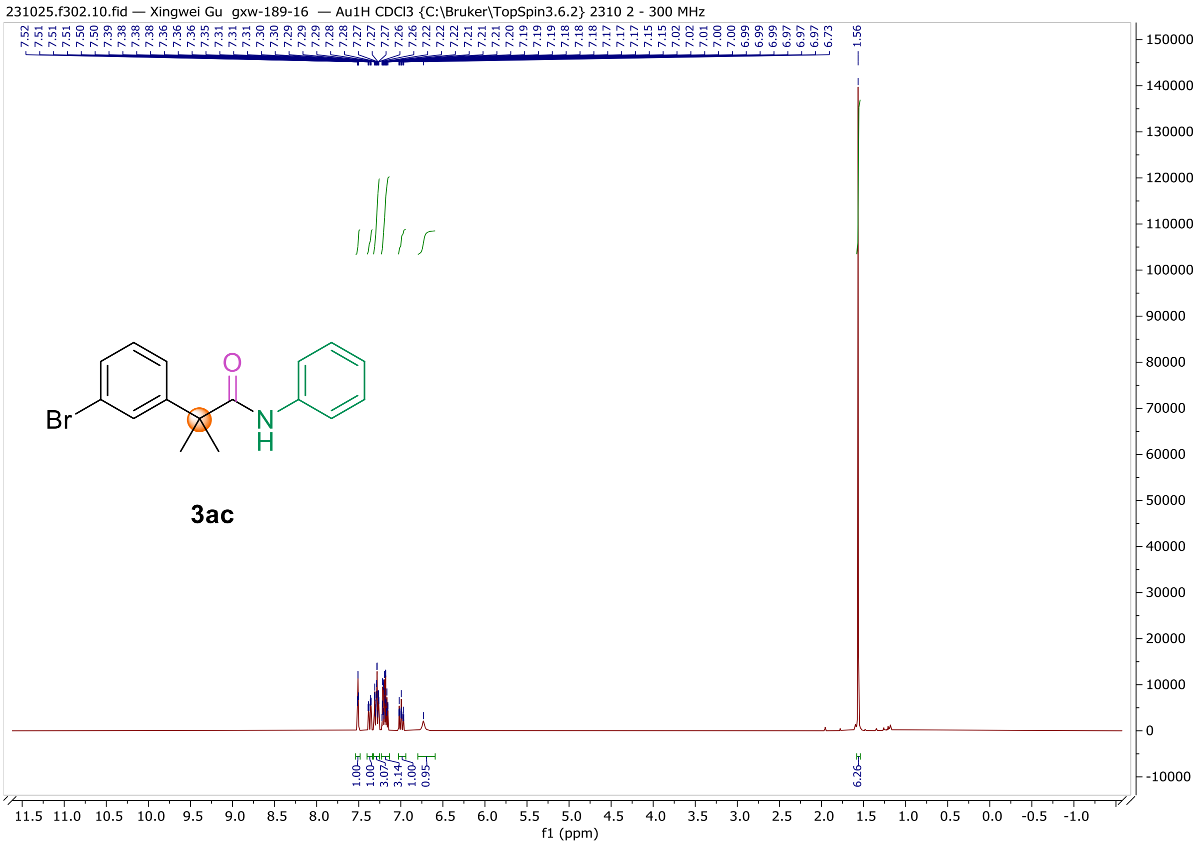
231025.f302.10.fid — Xingwei Gu gxw-189-16 — Au1H CDCl3 {C:\Bruker\TopSpin3.6.2} 2310 2 - 300 MHz
7.52 7.51 7.51 7.51 7.50 7.50 7.39 7.38 7.38 7.38 7.36 7.36 7.36 7.35 7.31 7.31 7.31 7.30 7.30 7.29 7.29 7.29 7.28 7.28 7.27 7.27 7.27 7.26 7.26 7.22 7.22 7.22 7.21 7.21 7.21 7.20 7.19 7.19 7.19 7.18 7.18 7.18 7.17 7.17 7.17 7.15 7.15 7.02 7.02 7.01 7.00 7.00 6.99 6.99 6.99 6.97 6.97 6.97 6.73
1.56
1.00 1.00 3.07 3.14 1.00 0.95 6.26
Br
O
N
H
3ac
f1 (ppm)

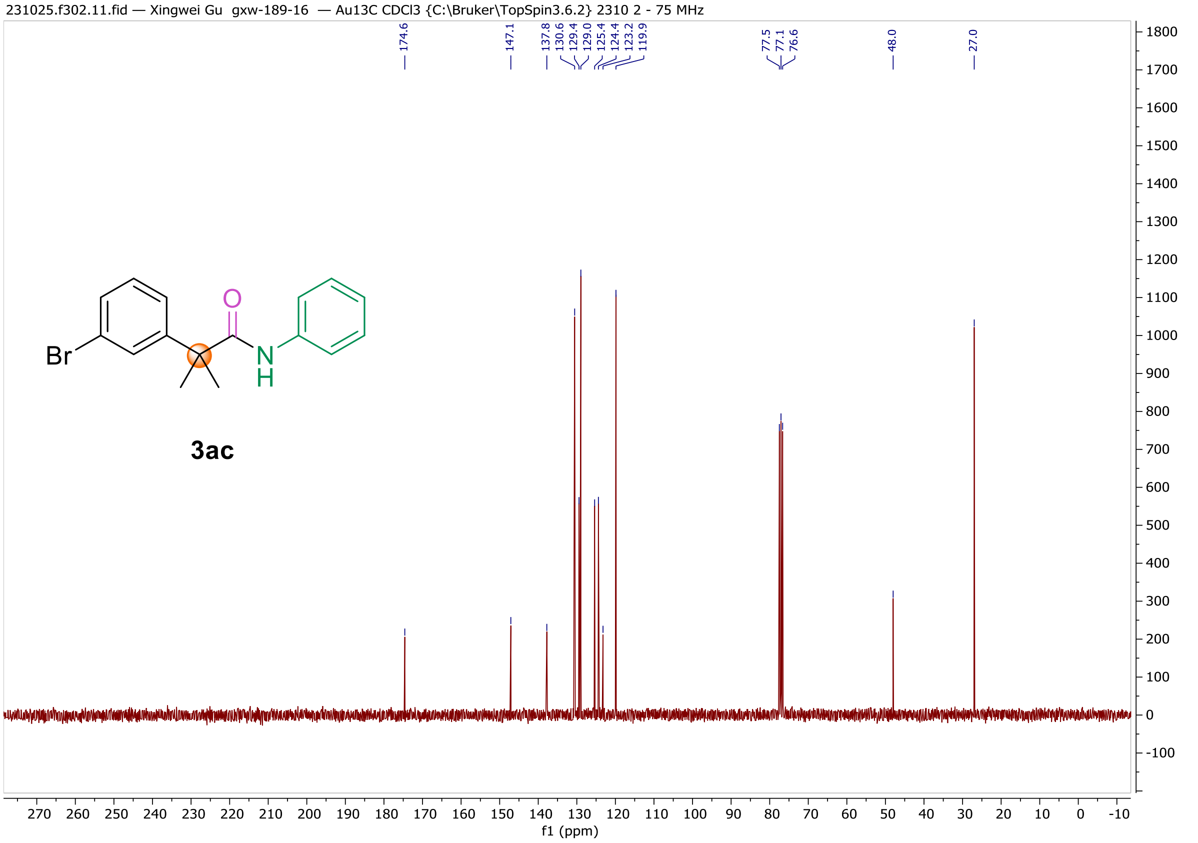
231025.f302.11.fid — Xingwei Gu gxw-189-16 — Au13C CDCl3 {C:\Bruker\TopSpin3.6.2} 2310 2 - 75 MHz
174.6
147.1
137.8 130.6 129.4 129.0 125.4 124.4 123.2 119.9
77.5 77.1 76.6
48.0
27.0
Br
O
N
H
3ac
f1 (ppm)

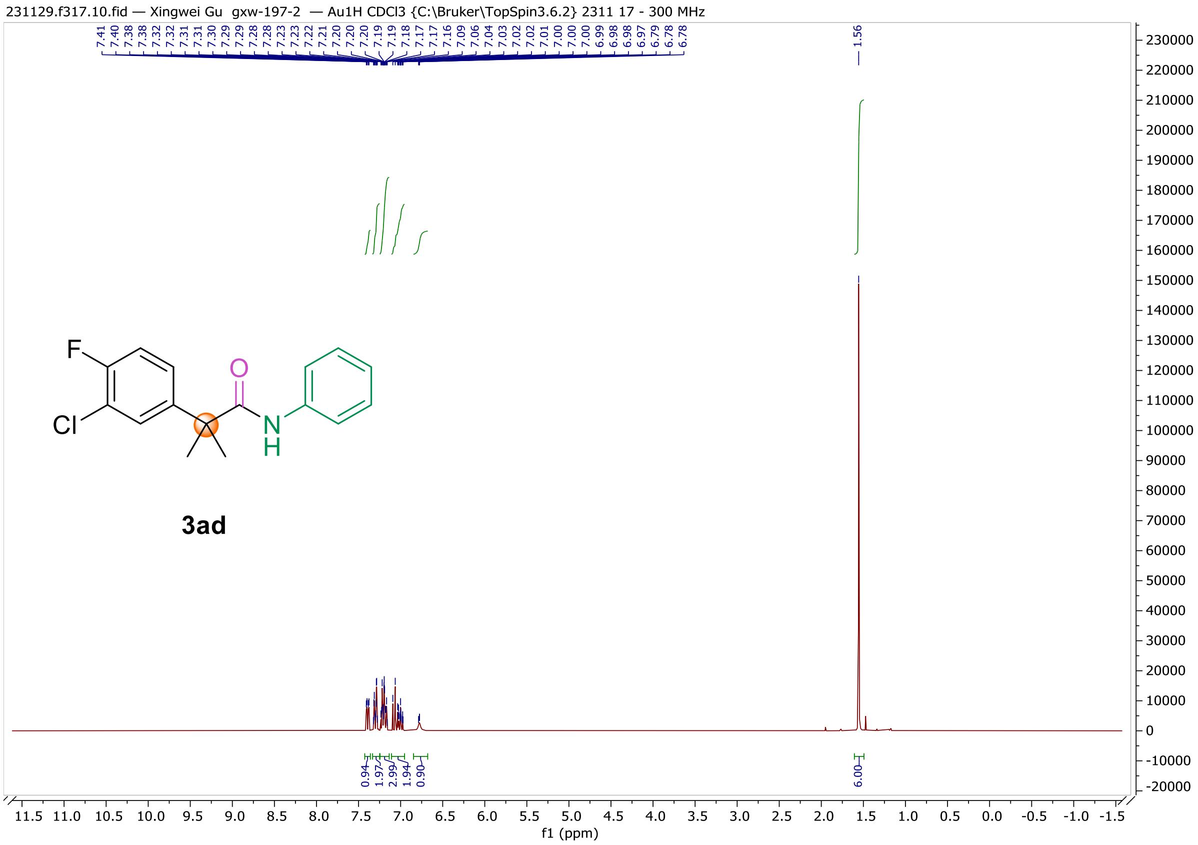
231129.f317.10.fid — Xingwei Gu gxw-197-2 — Au1H CDCl3 {C:\Bruker\TopSpin3.6.2} 2311 17 - 300 MHz
3ad
f1 (ppm)

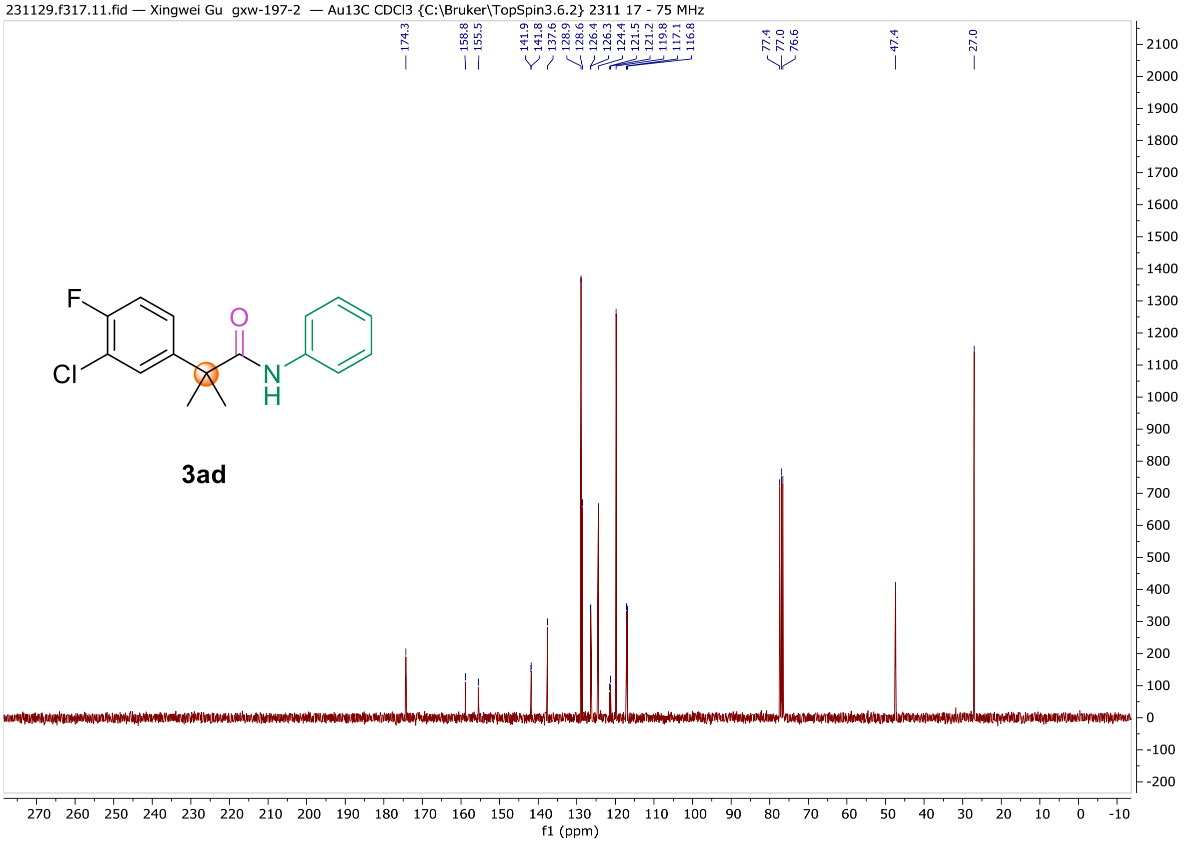
231129.f317.11.fid — Xingwei Gu gxw-197-2 — Au13C CDCl3 {C:\Bruker\TopSpin3.6.2} 2311 17 - 75 MHz
174.3
158.8
155.5
141.9
141.8
137.6
128.9
128.6
126.4
126.3
124.4
121.5
121.2
119.8
117.1
116.8
77.4
77.0
76.6
47.4
27.0
3ad
f1 (ppm)

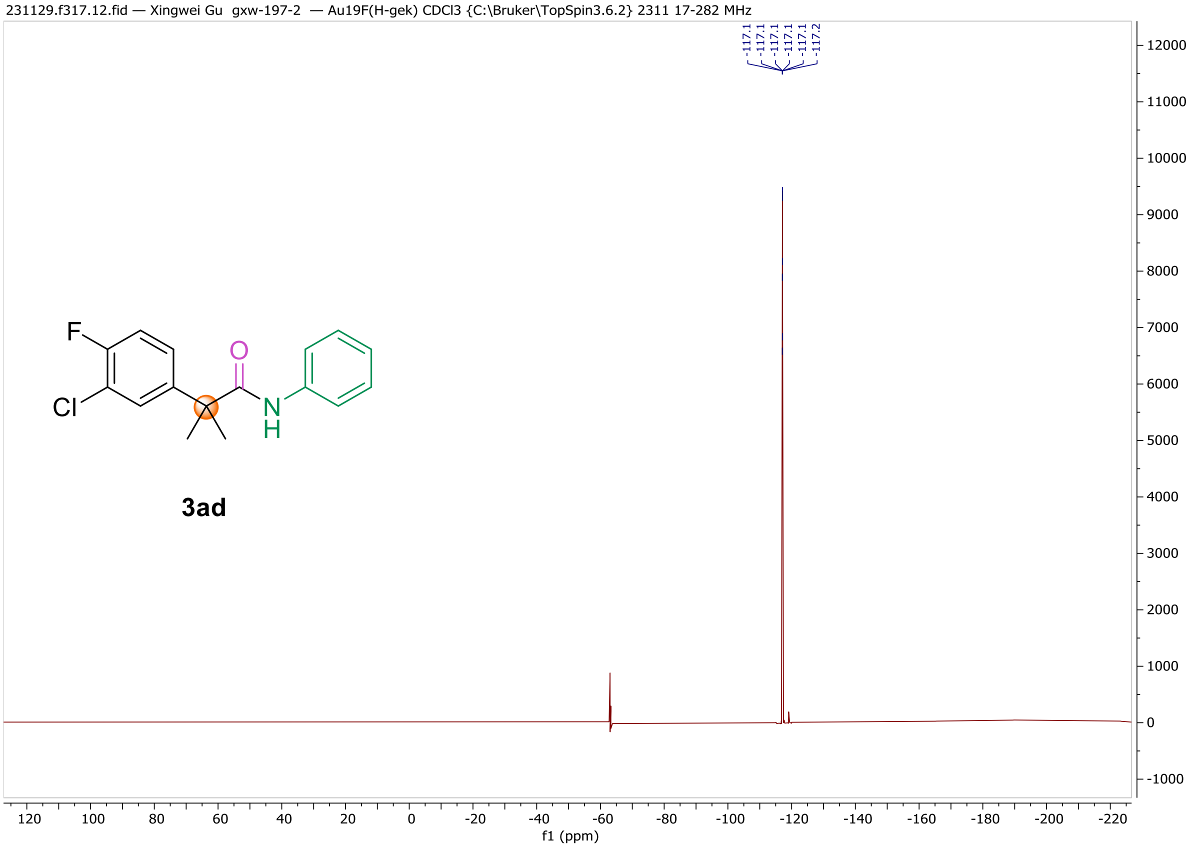
231129.f317.12.fid — Xingwei Gu gxw-197-2 — Au19F(H-gek) CDCl3 {C:\Bruker\TopSpin3.6.2} 2311 17-282 MHz
-117.1
-117.1
-117.1
-117.1
-117.1
-117.2
F
Cl
O
N
H
3ad
f1 (ppm)

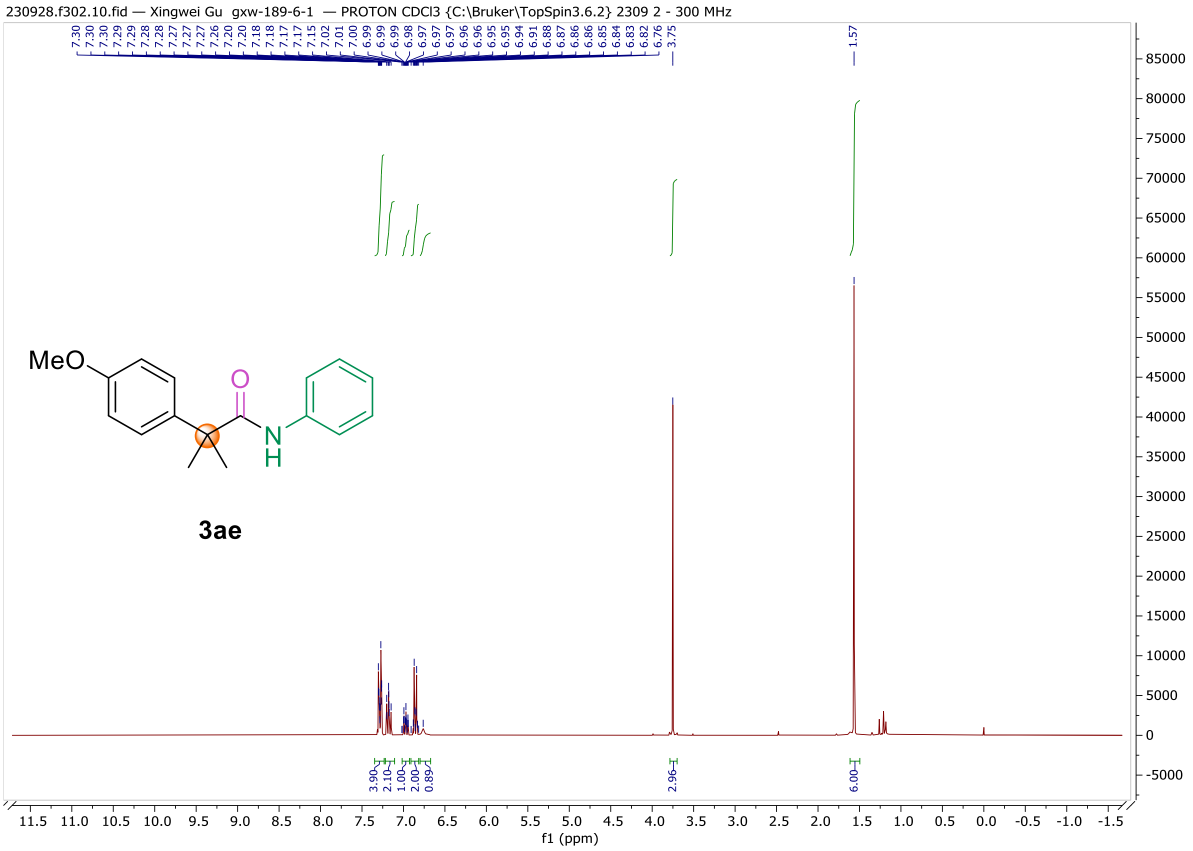
230928.f302.10.fid — Xingwei Gu gxw-189-6-1 — PROTON CDCl3 {C:\Bruker\TopSpin3.6.2} 2309 2 - 300 MHz
7.30 7.30 7.30 7.29 7.29 7.28 7.28 7.27 7.27 7.27 7.26 7.20 7.20 7.18 7.18 7.17 7.17 7.15 7.02 7.01 7.00 6.99 6.99 6.99 6.98 6.97 6.97 6.97 6.96 6.96 6.95 6.95 6.94 6.91 6.88 6.87 6.86 6.86 6.85 6.84 6.83 6.82 6.76 3.75
1.57
MeO
O
N
H
3ae
3.90 2.10 1.00 2.00 0.89
2.96
6.00
f1 (ppm)

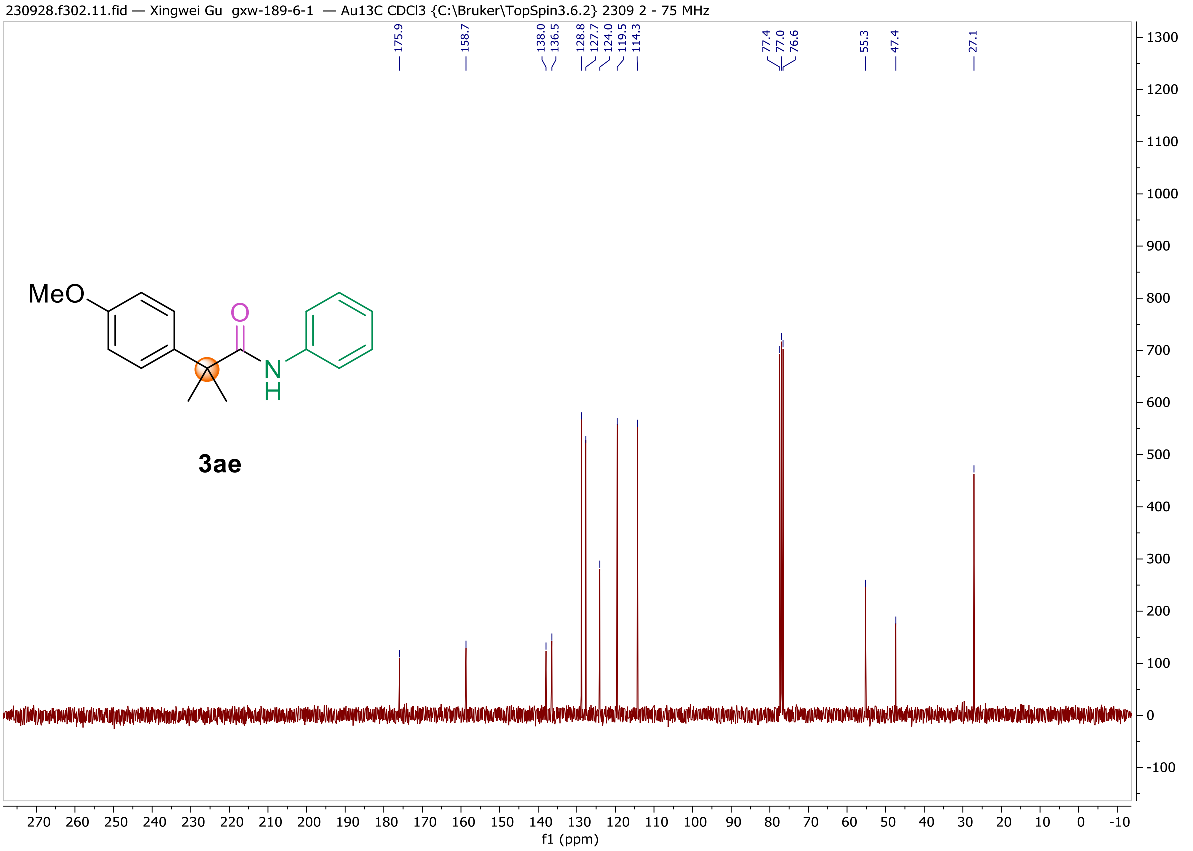
230928.f302.11.fid — Xingwei Gu gxw-189-6-1 — Au13C CDCl3 {C:\Bruker\TopSpin3.6.2} 2309 2 - 75 MHz
175.9
158.7
138.0 136.5
128.8 127.7 124.0 119.5 114.3
77.4 77.0 76.6
55.3
47.4
27.1
MeO
O
N
H
3ae
f1 (ppm)

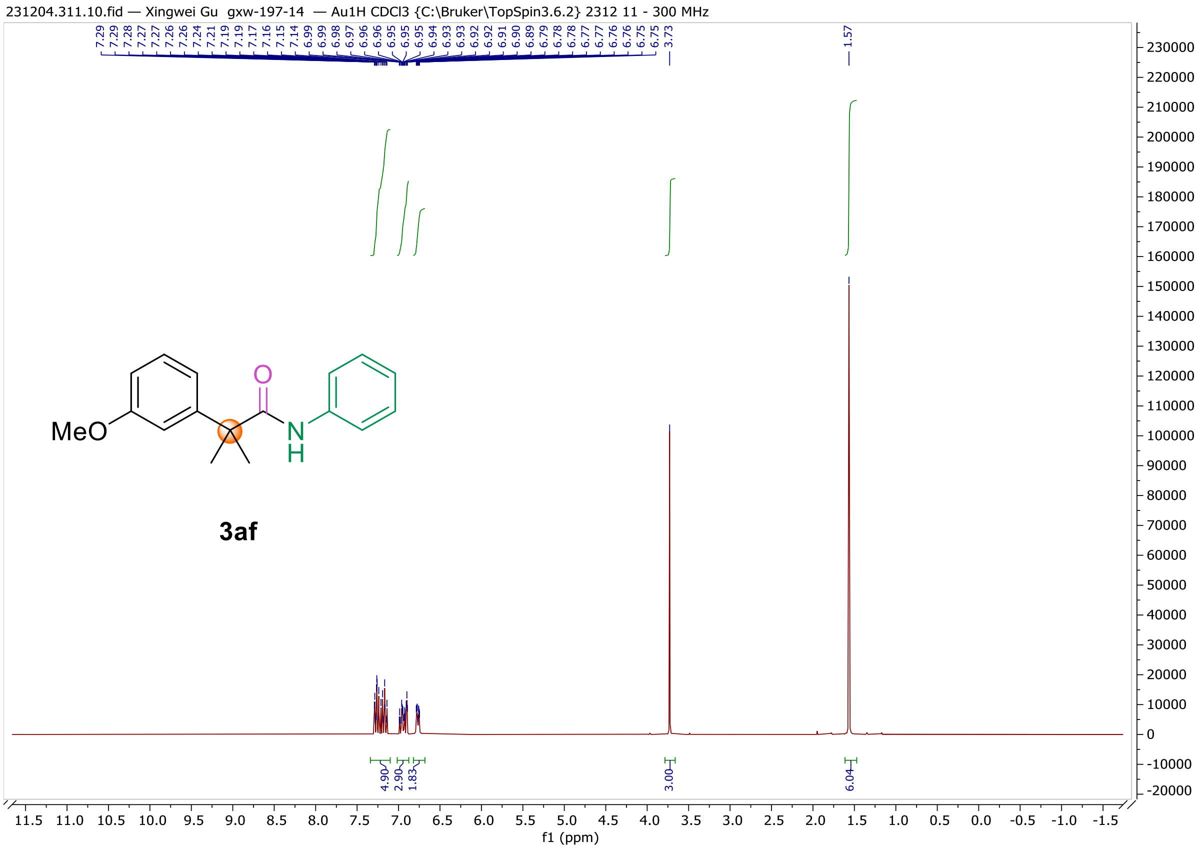
231204.311.10.fid — Xingwei Gu gxw-197-14 — Au1H CDCl3 {C:\Bruker\TopSpin3.6.2} 2312 11 - 300 MHz
MeO
O
N
H
3af
3.73
1.57
4.90
2.90
1.83
3.00
6.04
f1 (ppm)

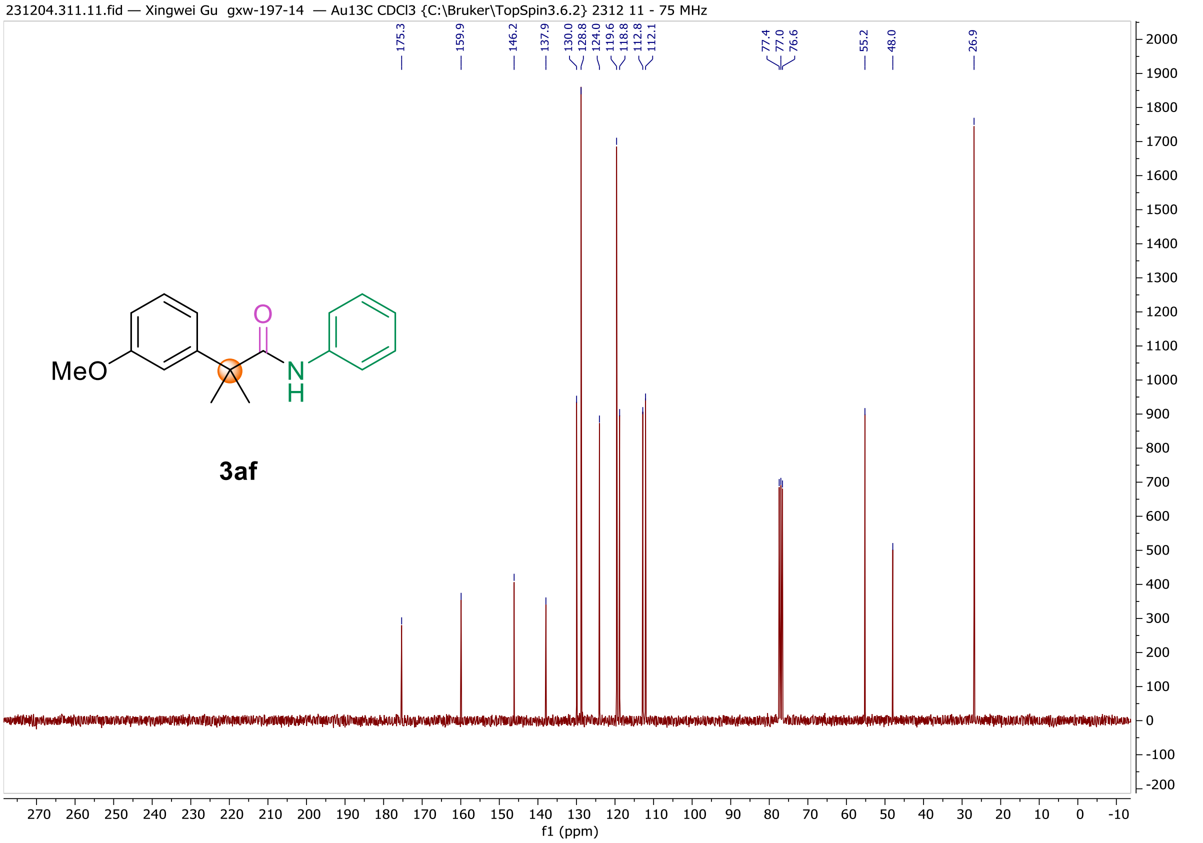
231204.311.11.fid — Xingwei Gu gxw-197-14 — Au13C CDCl3 {C:\Bruker\TopSpin3.6.2} 2312 11 - 75 MHz
175.3
159.9
146.2
137.9
130.0
128.8
124.0
119.6
118.8
112.8
112.1
77.4
77.0
76.6
55.2
48.0
26.9
MeO
O
N
H
3af
f1 (ppm)

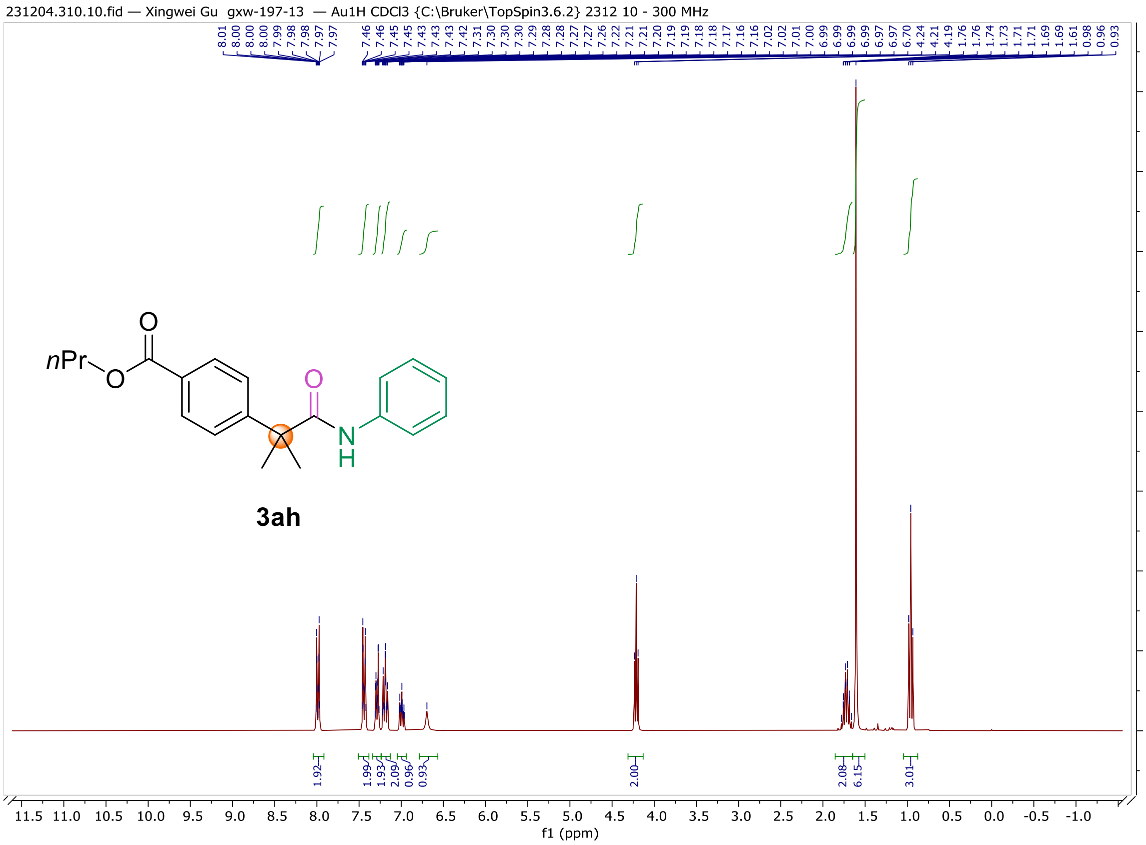
231204.310.10.fid — Xingwei Gu gxw-197-13 — Au1H CDCl3 {C:\Bruker\TopSpin3.6.2} 2312 10 - 300 MHz
8.01 8.00 8.00 7.99 7.98 7.97 7.97 7.46 7.46 7.45 7.45 7.43 7.43 7.42 7.31 7.30 7.30 7.29 7.28 7.28 7.27 7.27 7.26 7.22 7.21 7.21 7.20 7.19 7.19 7.18 7.18 7.17 7.16 7.02 7.02 7.01 7.00 6.99 6.99 6.99 6.99 6.97 6.70 4.24 4.21 4.19 1.76 1.76 1.74 1.73 1.71 1.71 1.69 1.61 0.98 0.96 0.93
nPr
O
O
N
H
3ah
1.92 1.99 1.93 2.09 0.96 0.93 2.00 2.08 6.15 3.01
f1 (ppm)
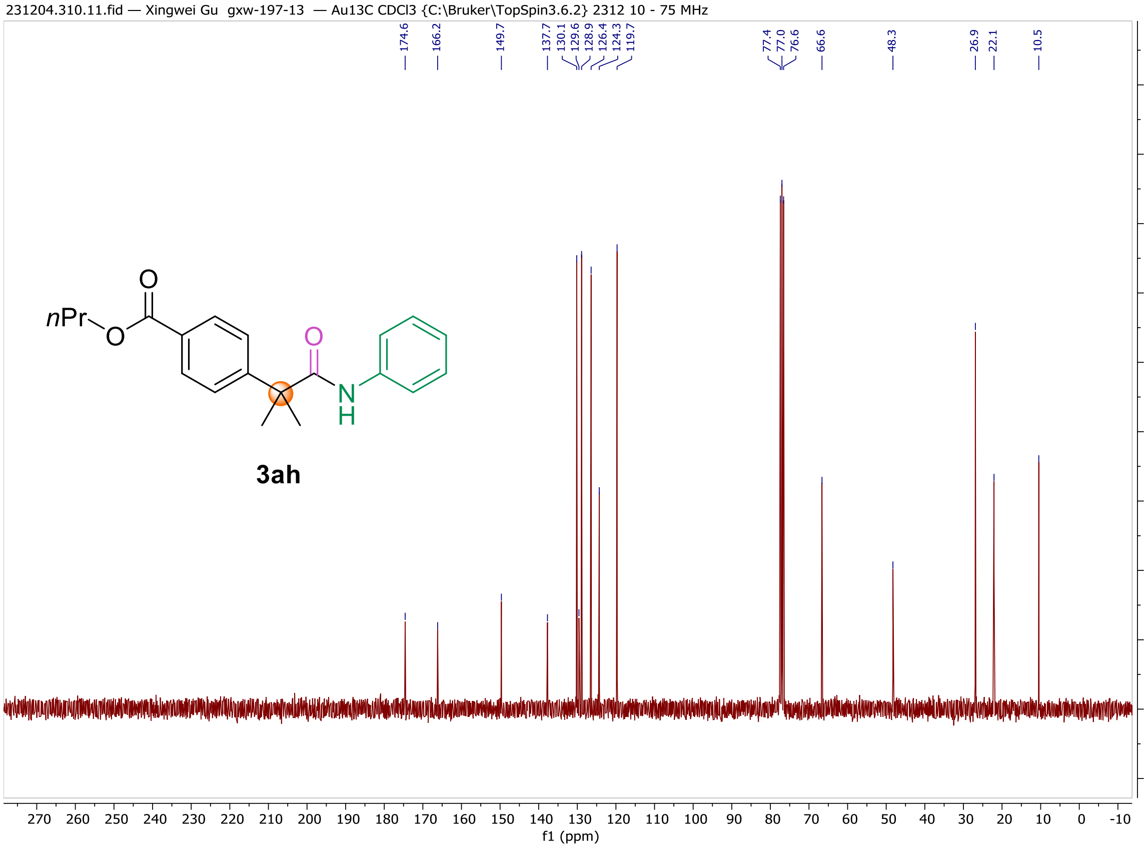
231204.310.11.fid — Xingwei Gu gxw-197-13 — Au13C CDCl3 {C:\Bruker\TopSpin3.6.2} 2312 10 - 75 MHz
174.6 166.2 149.7 137.7 130.1 129.6 128.9 126.4 124.3 119.7 77.4 77.0 76.6 66.6 48.3 26.9 22.1 10.5
nPr
O
O
N
H
3ah
f1 (ppm)

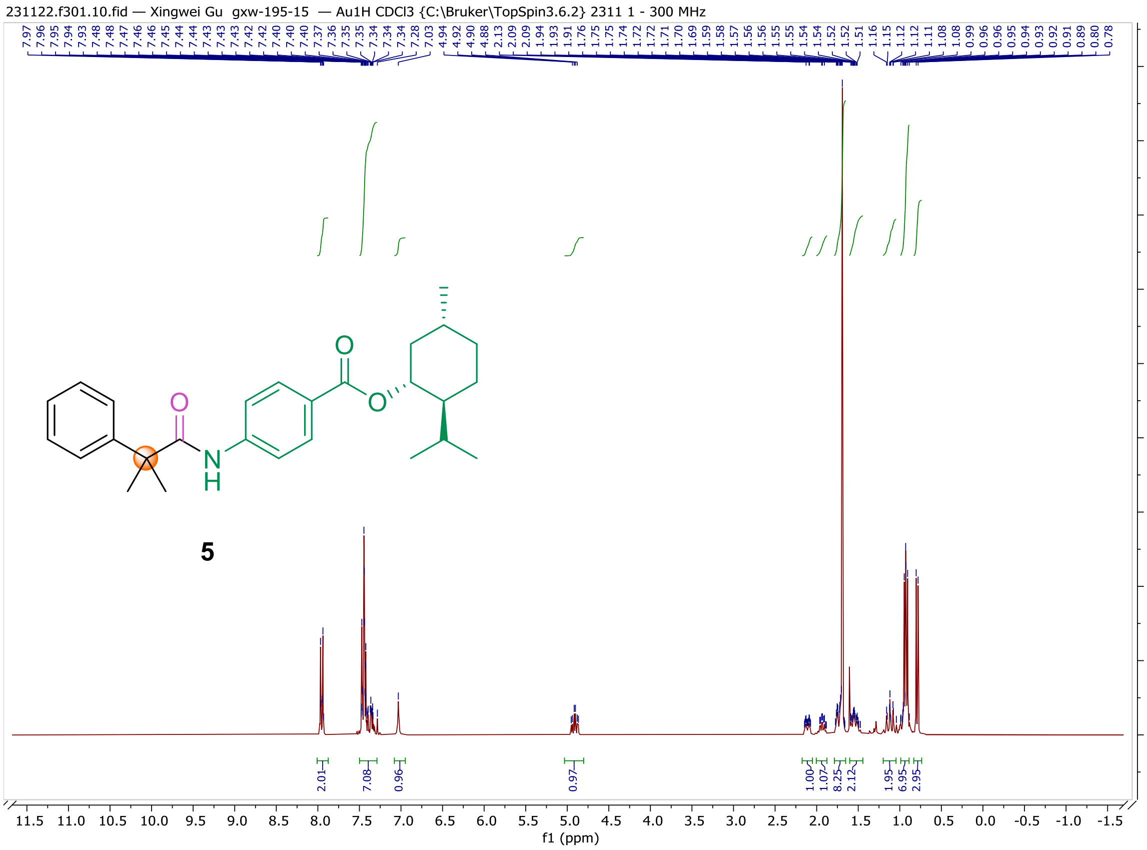
231122.f301.10.fid — Xingwei Gu gxw-195-15 — Au1H CDCl3 {C:\Bruker\TopSpin3.6.2} 2311 1 - 300 MHz
7.97 7.96 7.95 7.94 7.93 7.48 7.48 7.47 7.46 7.46 7.45 7.44 7.44 7.43 7.43 7.42 7.42 7.40 7.40 7.37 7.36 7.35 7.35 7.34 7.34 7.34 7.28 7.03 4.94 4.92 4.90 4.88 2.13 2.09 2.09 1.94 1.93 1.91 1.76 1.75 1.75 1.74 1.72 1.72 1.71 1.70 1.69 1.59 1.58 1.57 1.56 1.55 1.55 1.54 1.54 1.52 1.52 1.51 1.16 1.15 1.12 1.12 1.11 1.08 1.08 0.99 0.96 0.96 0.95 0.94 0.93 0.92 0.91 0.89 0.80 0.78
O
N
H
5
2.01
7.08
0.96
0.97
1.00
1.07
8.25
2.12
1.95
6.95
2.95
f1 (ppm)

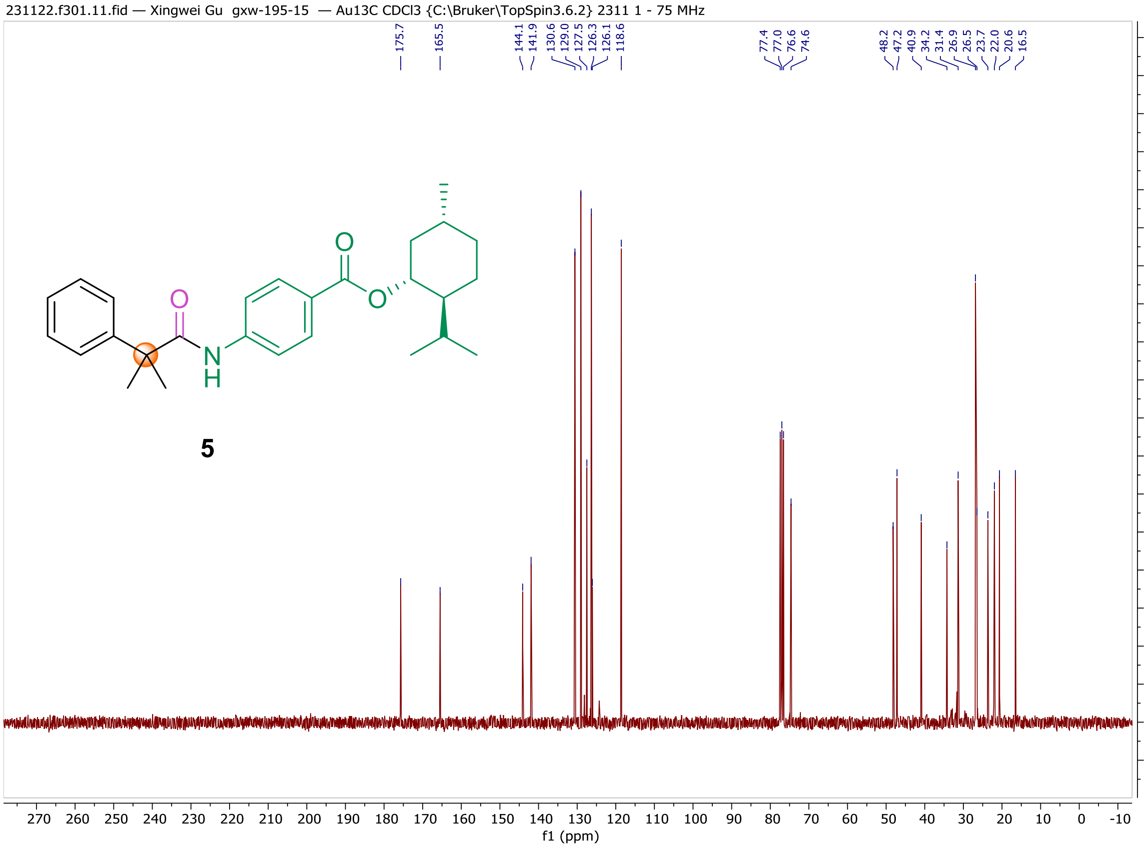
231122.f301.11.fid — Xingwei Gu gxw-195-15 — Au13C CDCl3 {C:\Bruker\TopSpin3.6.2} 2311 1 - 75 MHz
175.7
165.5
144.1 141.9
130.6 129.0 127.5 126.3 126.1 118.6
77.4 77.0 76.6 74.6
48.2 47.2 40.9 34.2 31.4 26.9 26.5 23.7 22.0 20.6 16.5
O
N
H
5
f1 (ppm)

240724.f347.10.fid — Xingwei Gu gxw-198-1 — Au1H CDCl3 {C:\Bruker\TopSpin3.6.2} 2407 47 - 300 MHz

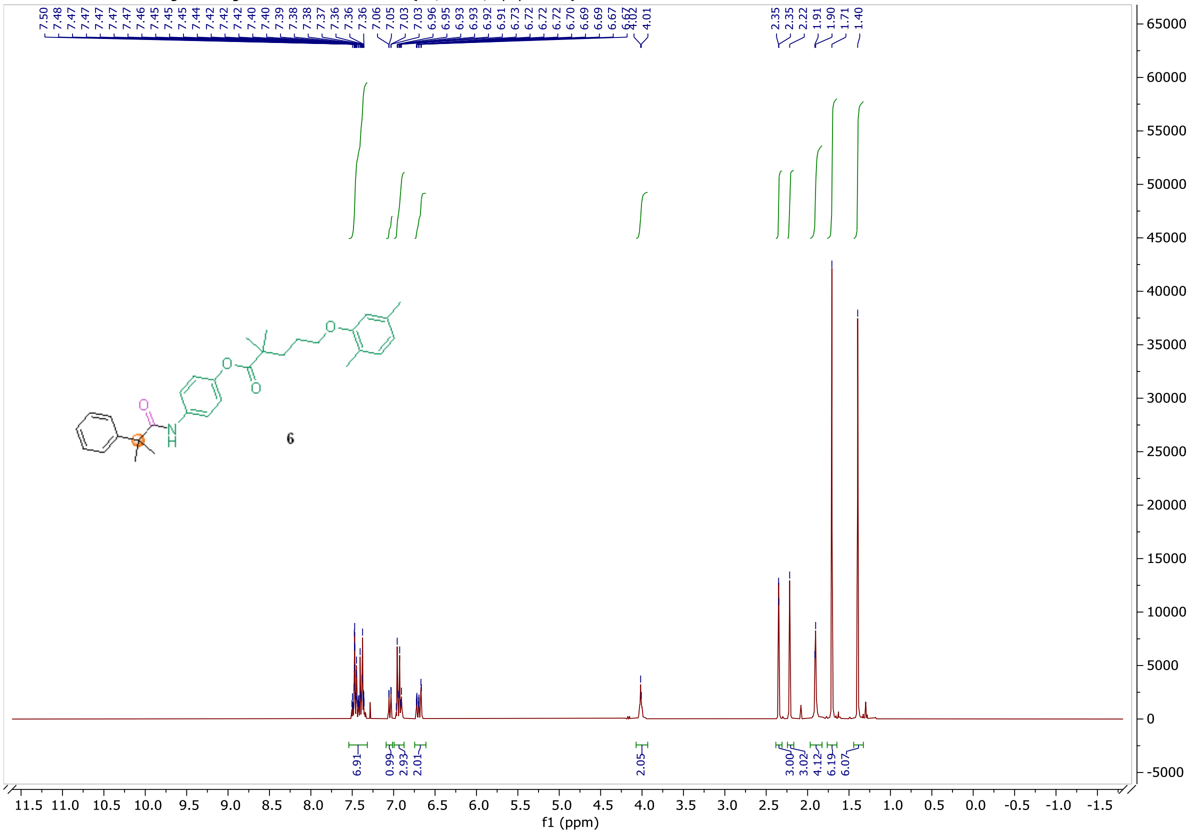


240724.f347.11.fid — Xingwei Gu gxw-198-1 — Au13C CDCl3 {C:\Bruker\TopSpin3.6.2} 2407 47 - 75 MHz

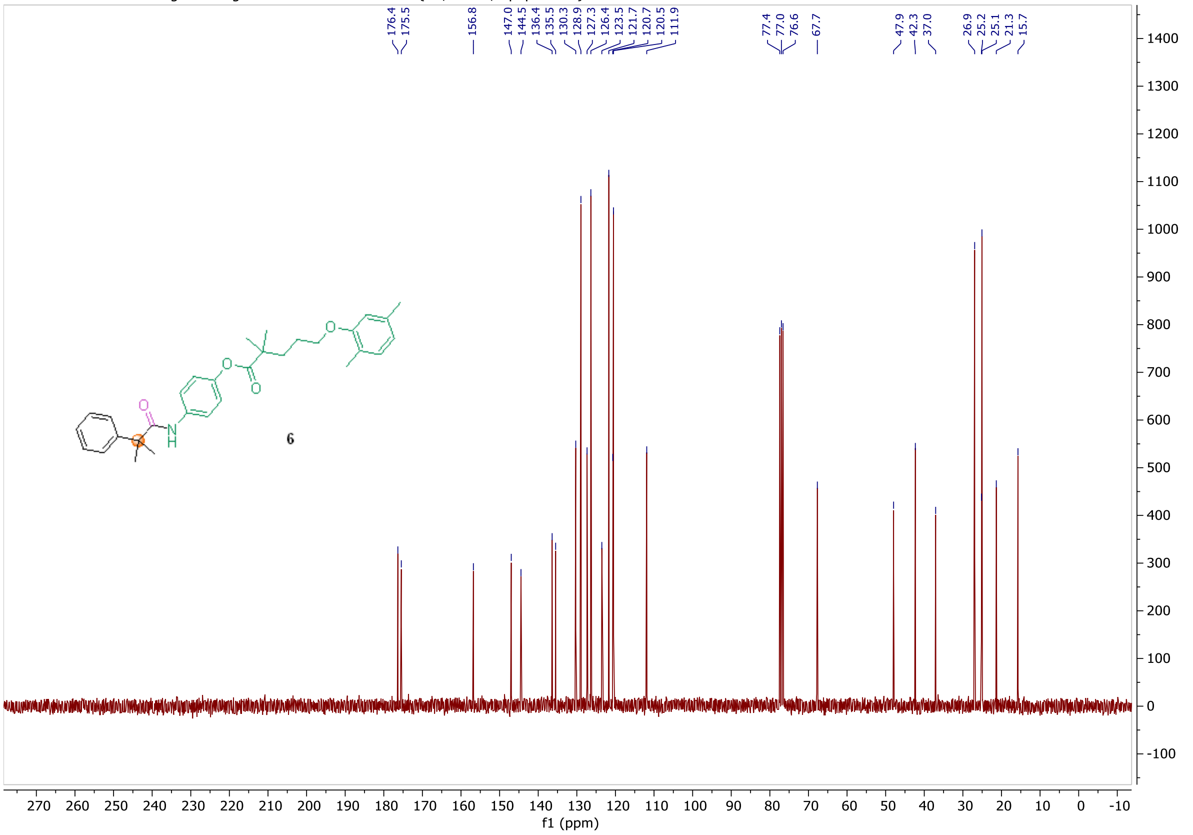

231122.f302.10.fid — Xingwei Gu gxw-195-16 — Au1H CDCl3 {C:\Bruker\TopSpin3.6.2} 2311 2 - 300 MHz

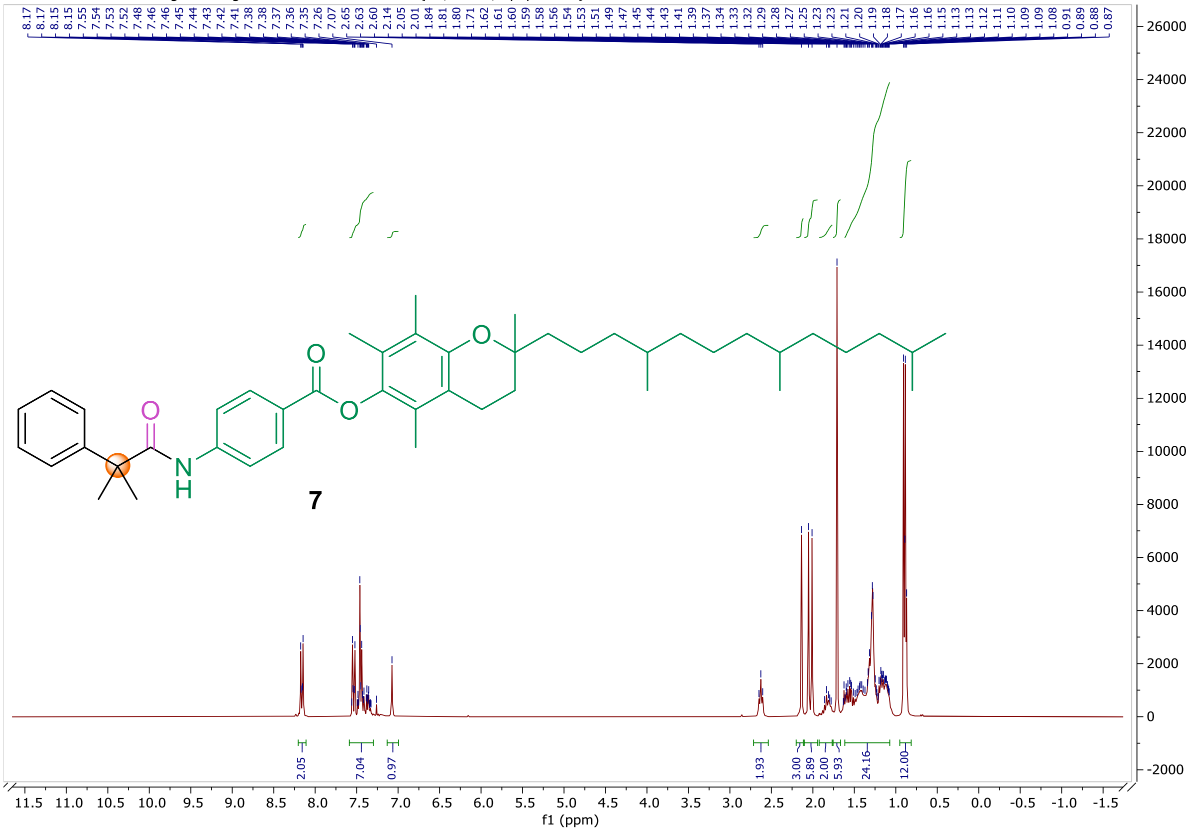

231122.f302.11.fid — Xingwei Gu gxw-195-16 — Au13C CDCl3 {C:\Bruker\TopSpin3.6.2} 2311 2 - 75 MHz

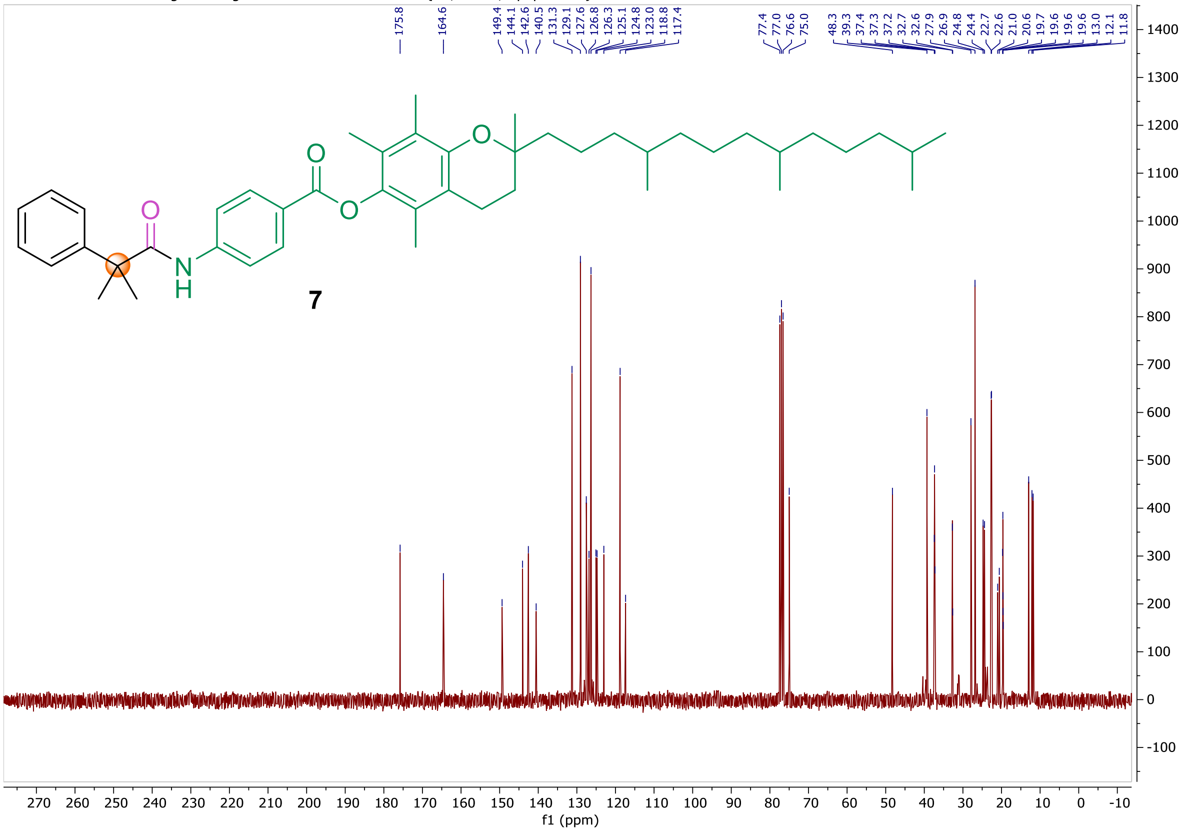

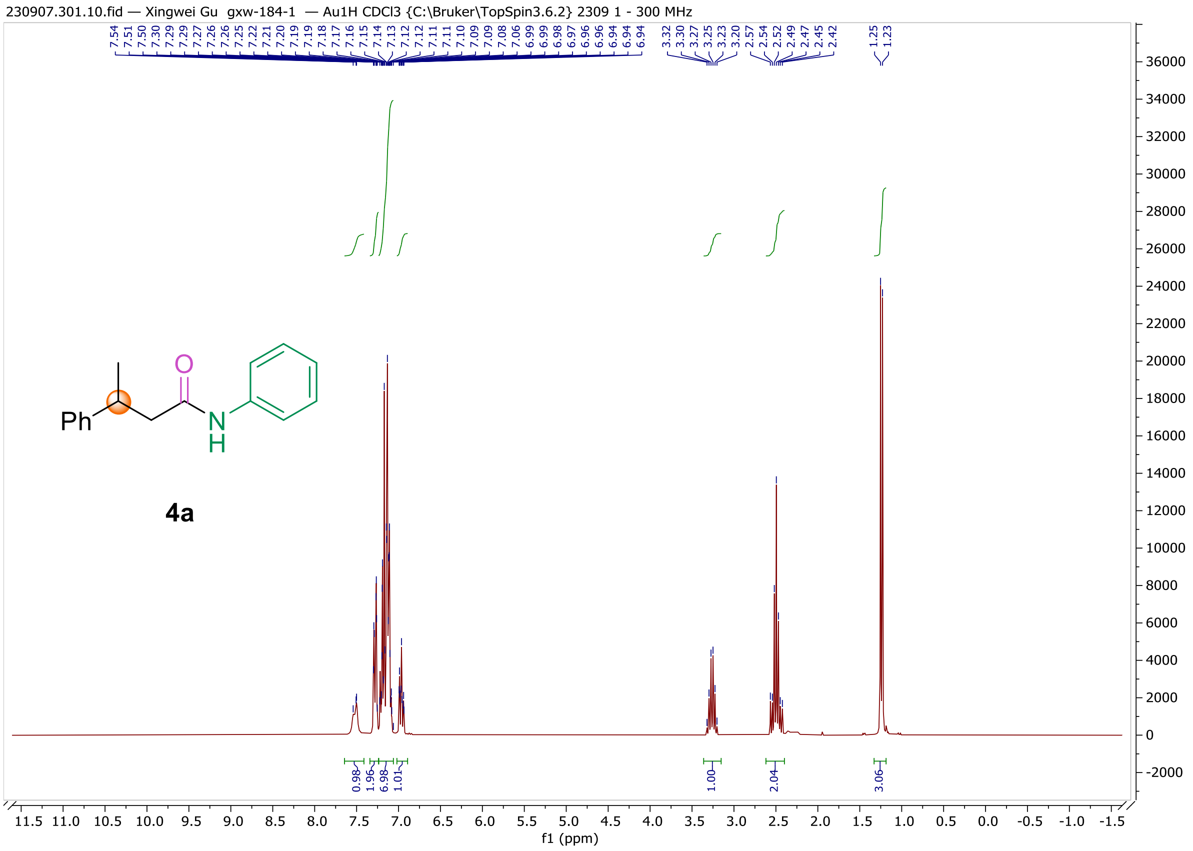
230907.301.10.fid — Xingwei Gu gxw-184-1 — Au1H CDCl3 {C:\Bruker\TopSpin3.6.2} 2309 1 - 300 MHz
4a
Ph
O
N
H
f1 (ppm)

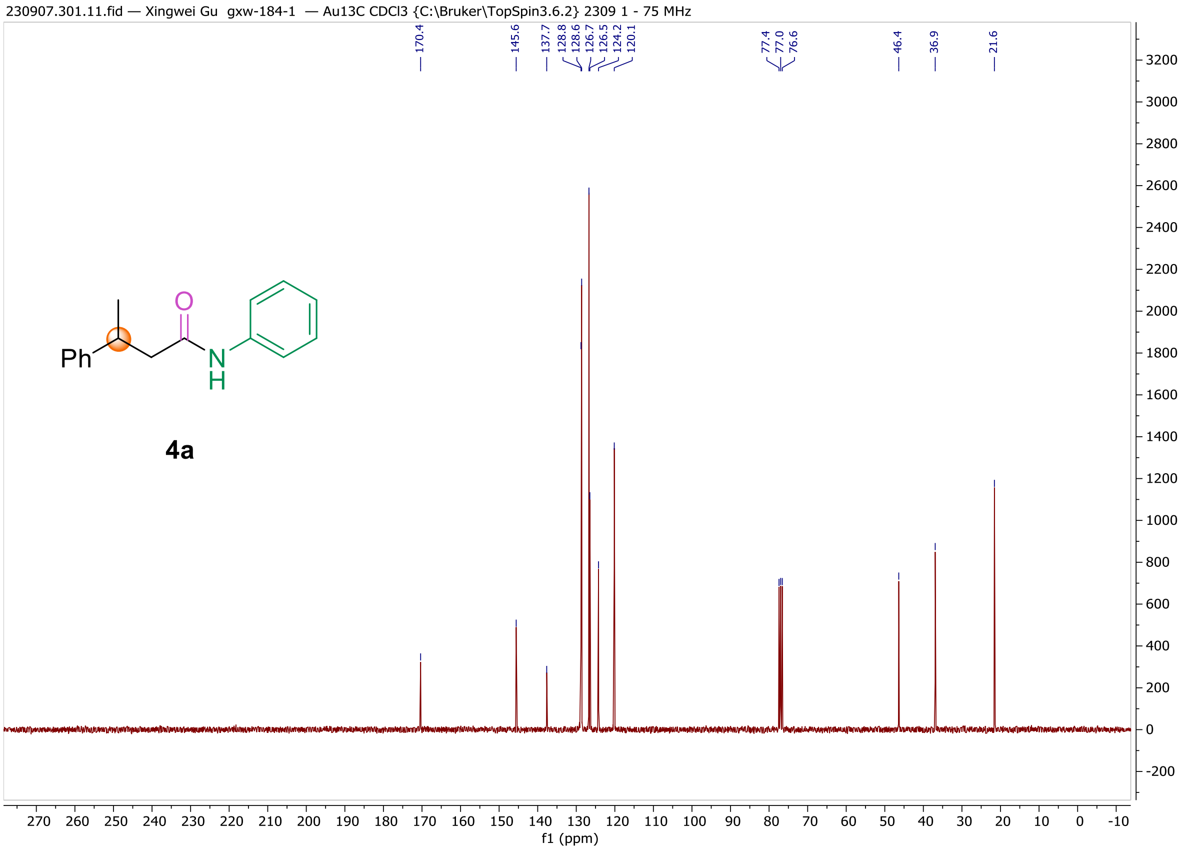
230907.301.11.fid — Xingwei Gu gxw-184-1 — Au13C CDCl3 {C:\Bruker\TopSpin3.6.2} 2309 1 - 75 MHz
170.4
145.6
137.7
128.8
128.6
126.7
126.5
124.2
120.1
77.4
77.0
76.6
46.4
36.9
21.6
4a
Ph
O
N
H
f1 (ppm)

231005.314.10.fid — Xingwei Gu gxw-194-2 — Au1H CDCl3 {C:\Bruker\TopSpin3.6.2} 2310 14 - 300 MHz

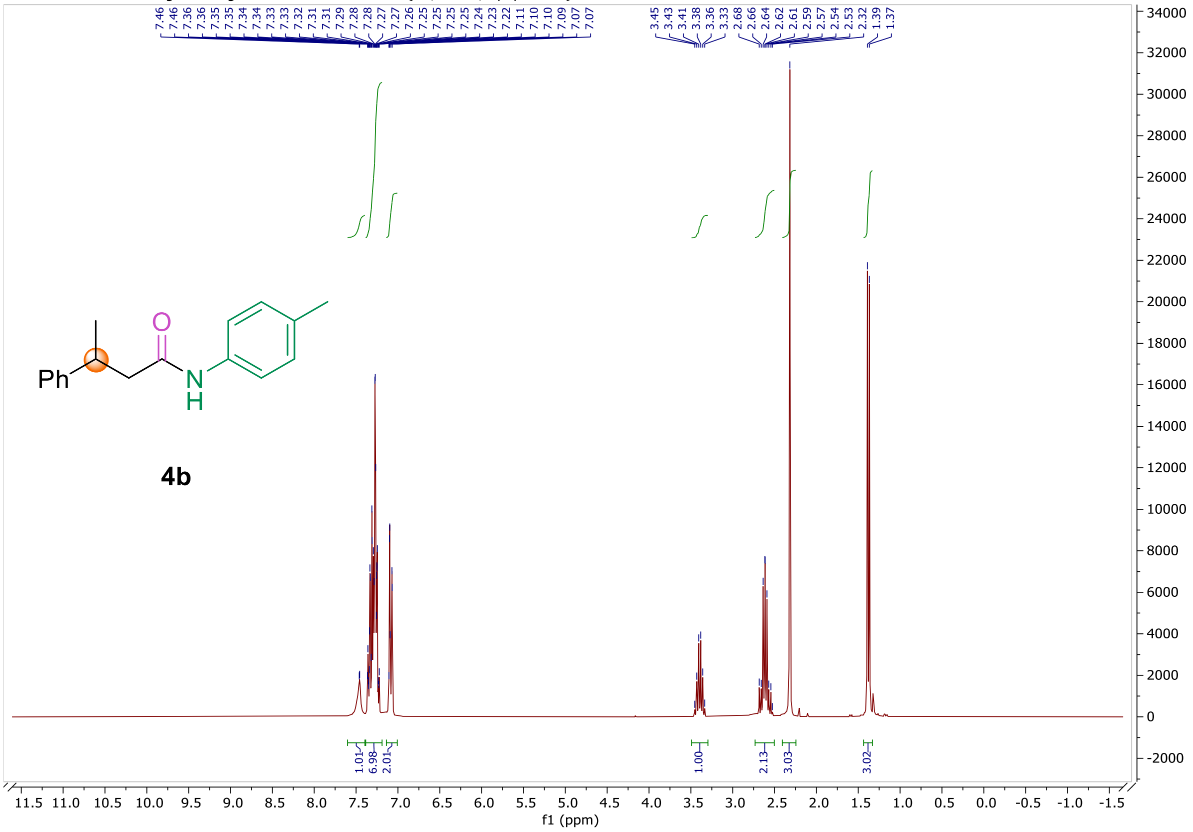


231001.302.11.fid — Xingwei Gu, gxw-194-2 — Au13C CDCl3 {C:\Bruker\TopSpin3.6.2} 2310 2 - 75 MHz

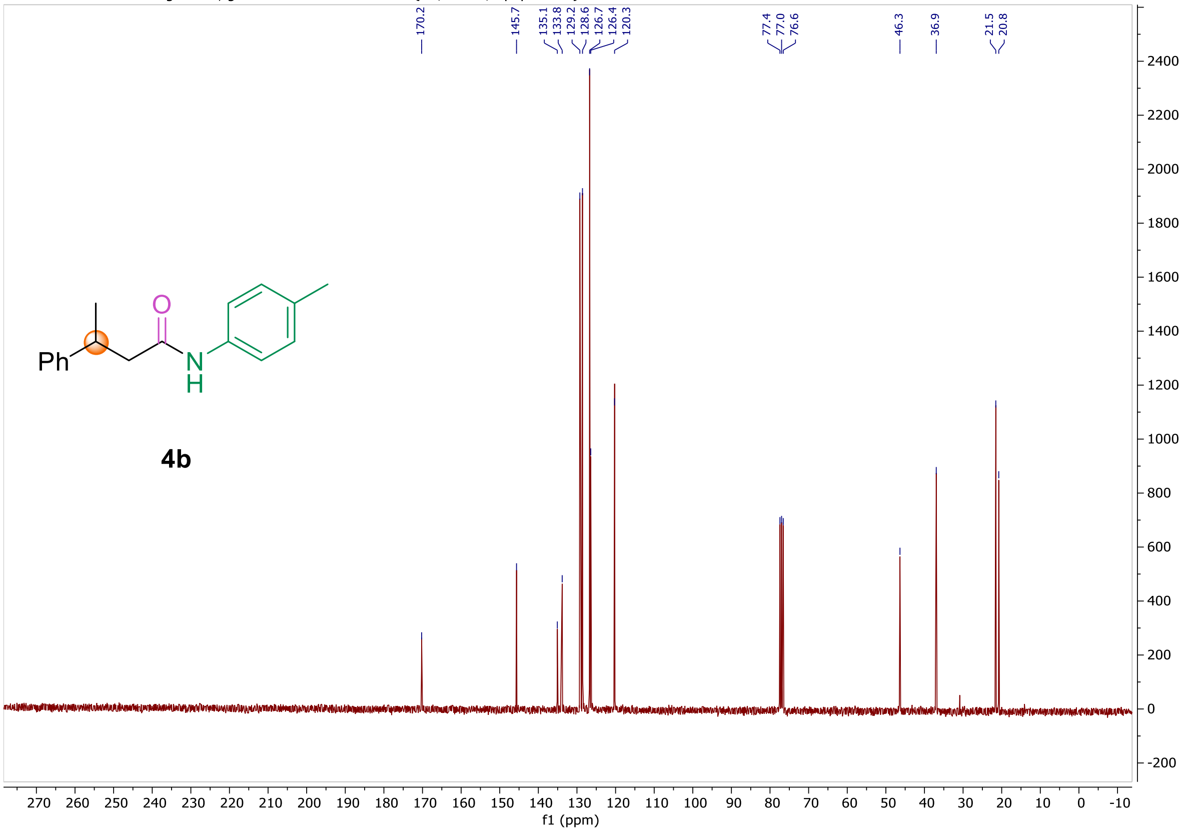

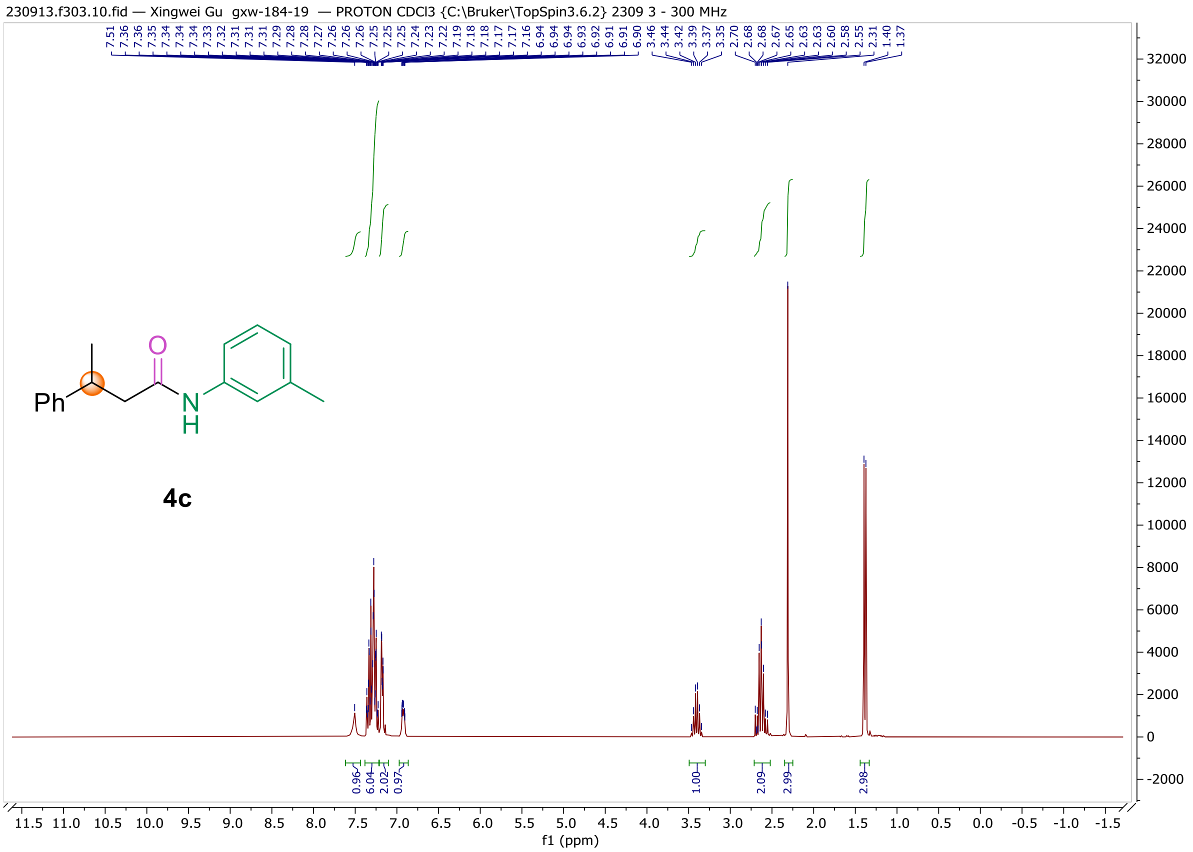
230913.f303.10.fid — Xingwei Gu gxw-184-19 — PROTON CDCl3 {C:\Bruker\TopSpin3.6.2} 2309 3 - 300 MHz
Ph
O
N
H
4c
f1 (ppm)

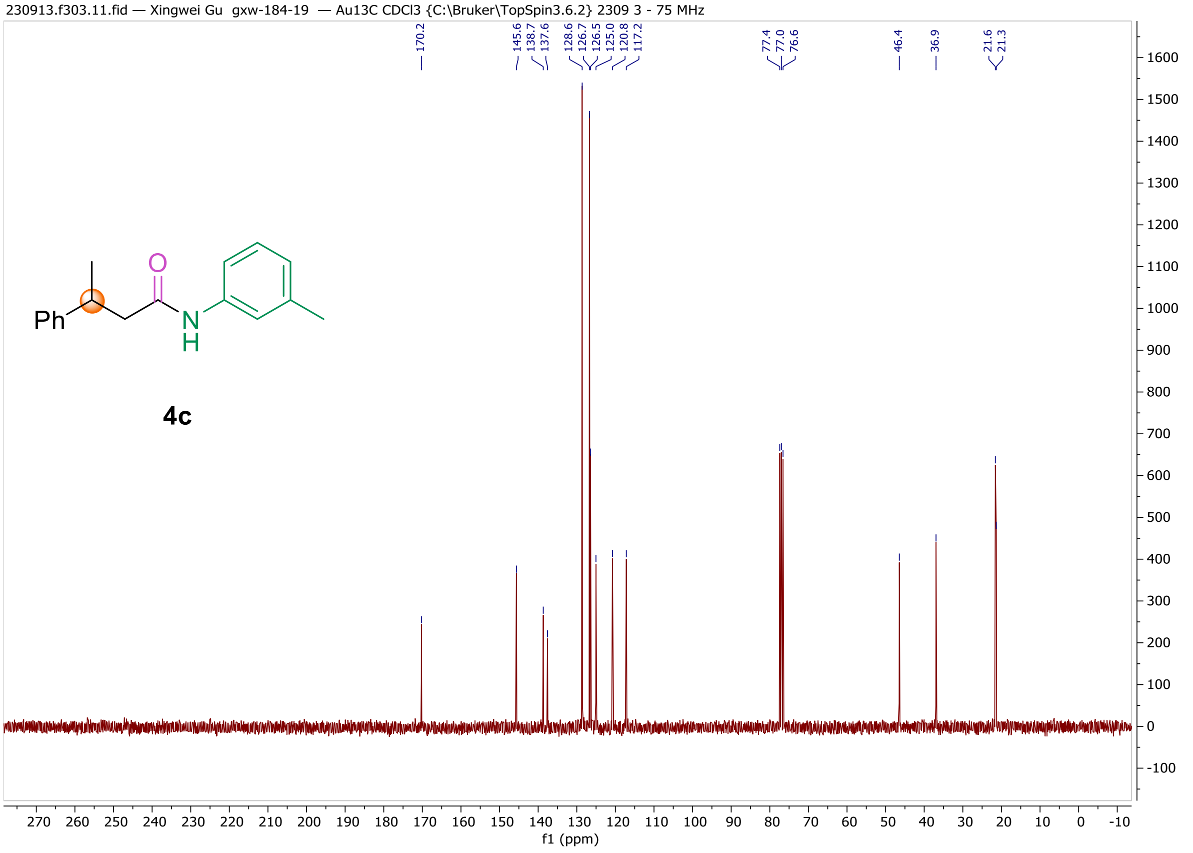
230913.f303.11.fid — Xingwei Gu gxw-184-19 — Au13C CDCl3 {C:\Bruker\TopSpin3.6.2} 2309 3 - 75 MHz
170.2
145.6
138.7
137.6
128.6
126.7
126.5
125.0
120.8
117.2
77.4
77.0
76.6
46.4
36.9
21.6
21.3
Ph
O
N
H
4c
f1 (ppm)

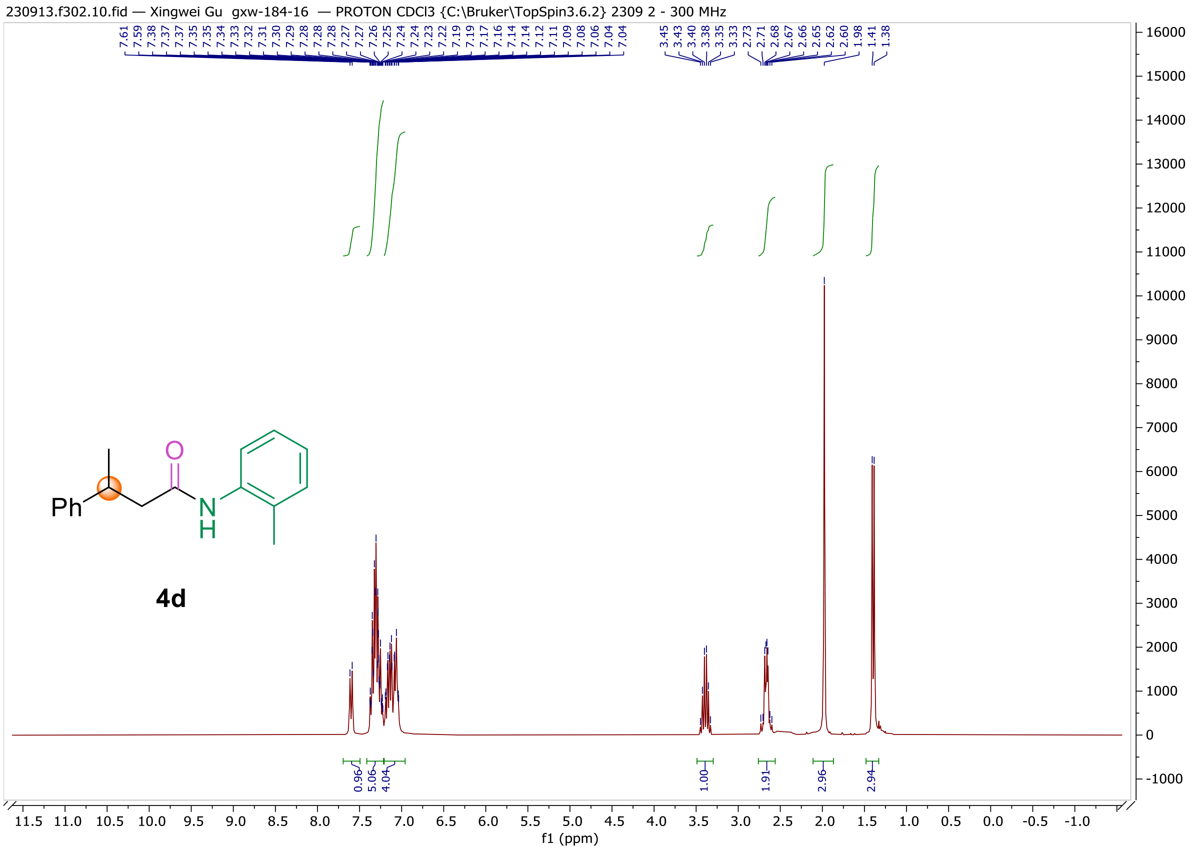
230913.f302.10.fid — Xingwei Gu gxw-184-16 — PROTON CDCl3 {C:\Bruker\TopSpin3.6.2} 2309 2 - 300 MHz
7.61 7.59 7.38 7.37 7.37 7.35 7.35 7.34 7.33 7.32 7.31 7.30 7.29 7.28 7.28 7.28 7.27 7.27 7.26 7.25 7.24 7.24 7.23 7.22 7.19 7.19 7.17 7.16 7.14 7.14 7.12 7.11 7.09 7.08 7.06 7.04 7.04
3.45 3.43 3.40 3.38 3.35 3.33 2.73 2.71 2.68 2.67 2.66 2.65 2.62 2.60 1.98 1.41 1.38
0.96 5.06 4.04 1.00 1.91 2.96 2.94
Ph
O
N
H
4d
f1 (ppm)
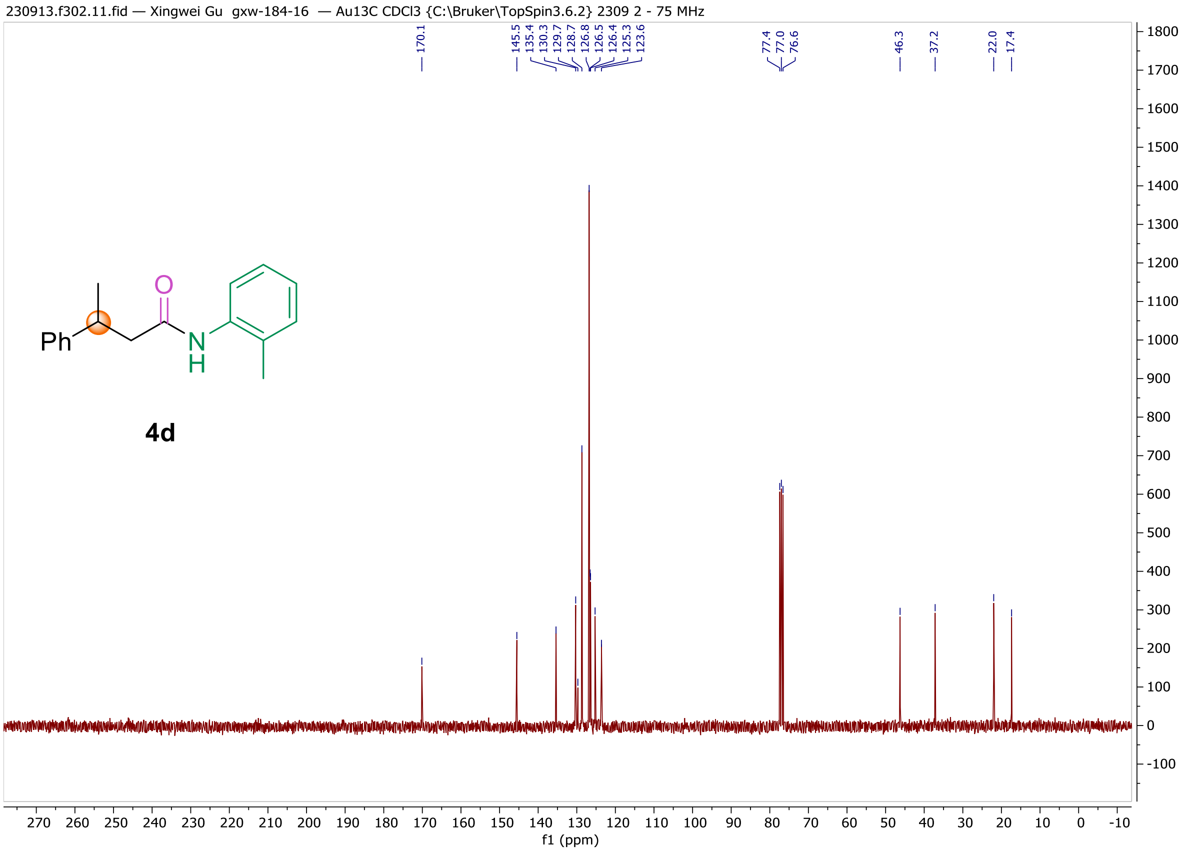
230913.f302.11.fid — Xingwei Gu gxw-184-16 — Au13C CDCl3 {C:\Bruker\TopSpin3.6.2} 2309 2 - 75 MHz
170.1
145.5 135.4 130.3 129.7 128.7 126.8 126.5 126.4 125.3 123.6
77.4 77.0 76.6
46.3
37.2
22.0 17.4
Ph
O
N
H
4d
f1 (ppm)

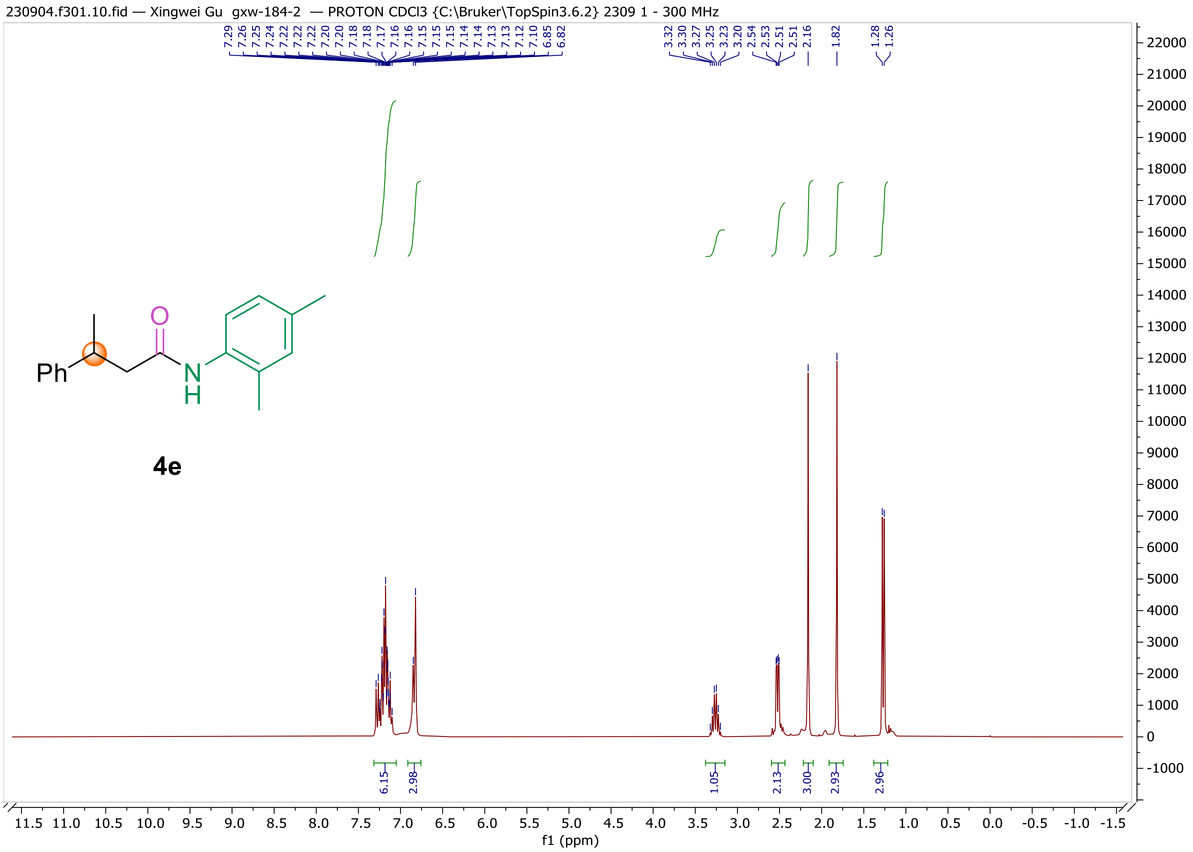
230904.f301.10.fid — Xingwei Gu gxw-184-2 — PROTON CDCl3 {C:\Bruker\TopSpin3.6.2} 2309 1 - 300 MHz
7.29 7.26 7.25 7.24 7.22 7.22 7.22 7.20 7.20 7.18 7.17 7.16 7.16 7.15 7.15 7.15 7.14 7.14 7.13 7.13 7.12 7.10 6.85 6.82
3.32 3.30 3.27 3.25 3.23 3.20 2.54 2.53 2.51 2.51 2.16 1.82 1.28 1.26
Ph
O
N
H
4e
6.15 2.98 1.05 2.13 3.00 2.93 2.96
f1 (ppm)

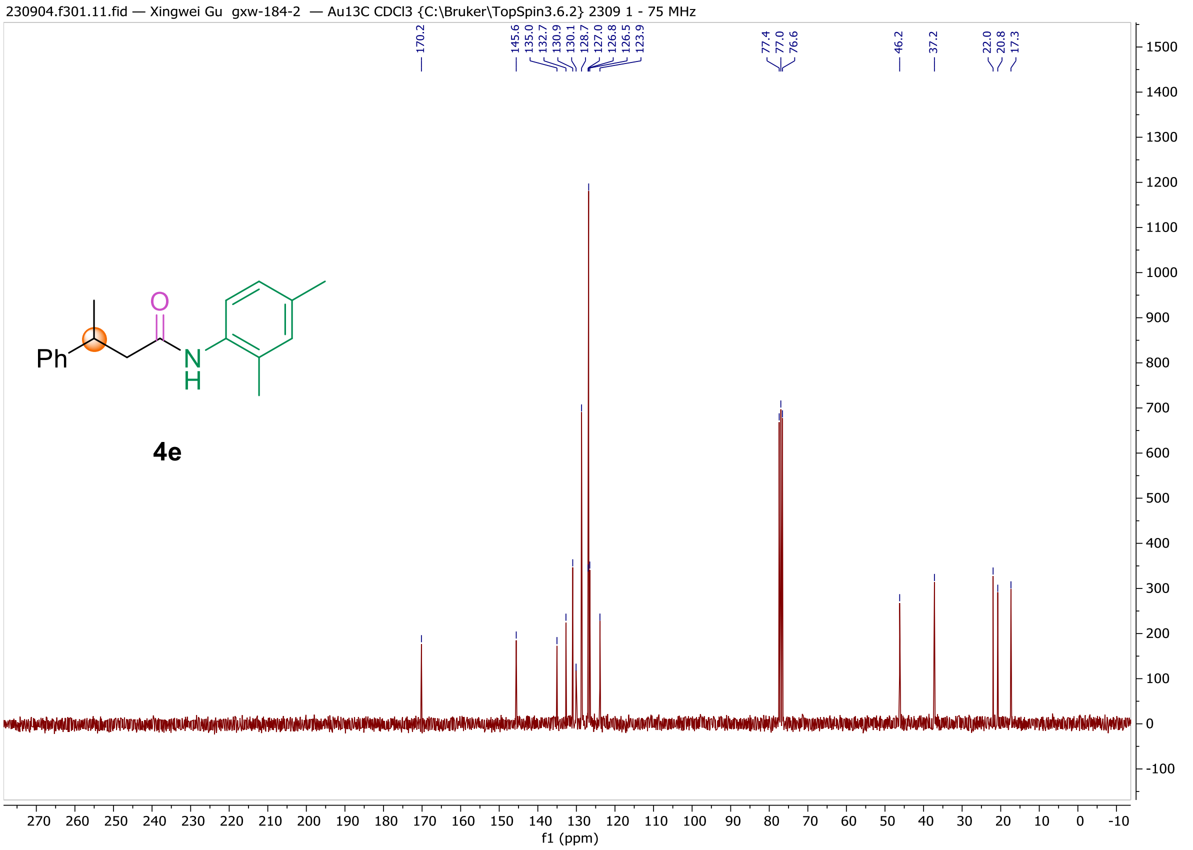
230904.f301.11.fid — Xingwei Gu gxw-184-2 — Au13C CDCl3 {C:\Bruker\TopSpin3.6.2} 2309 1 - 75 MHz
170.2 145.6 135.0 132.7 130.9 130.1 128.7 127.0 126.8 126.5 123.9 77.4 77.0 76.6 46.2 37.2 22.0 20.8 17.3
Ph
O
N
H
4e
f1 (ppm)

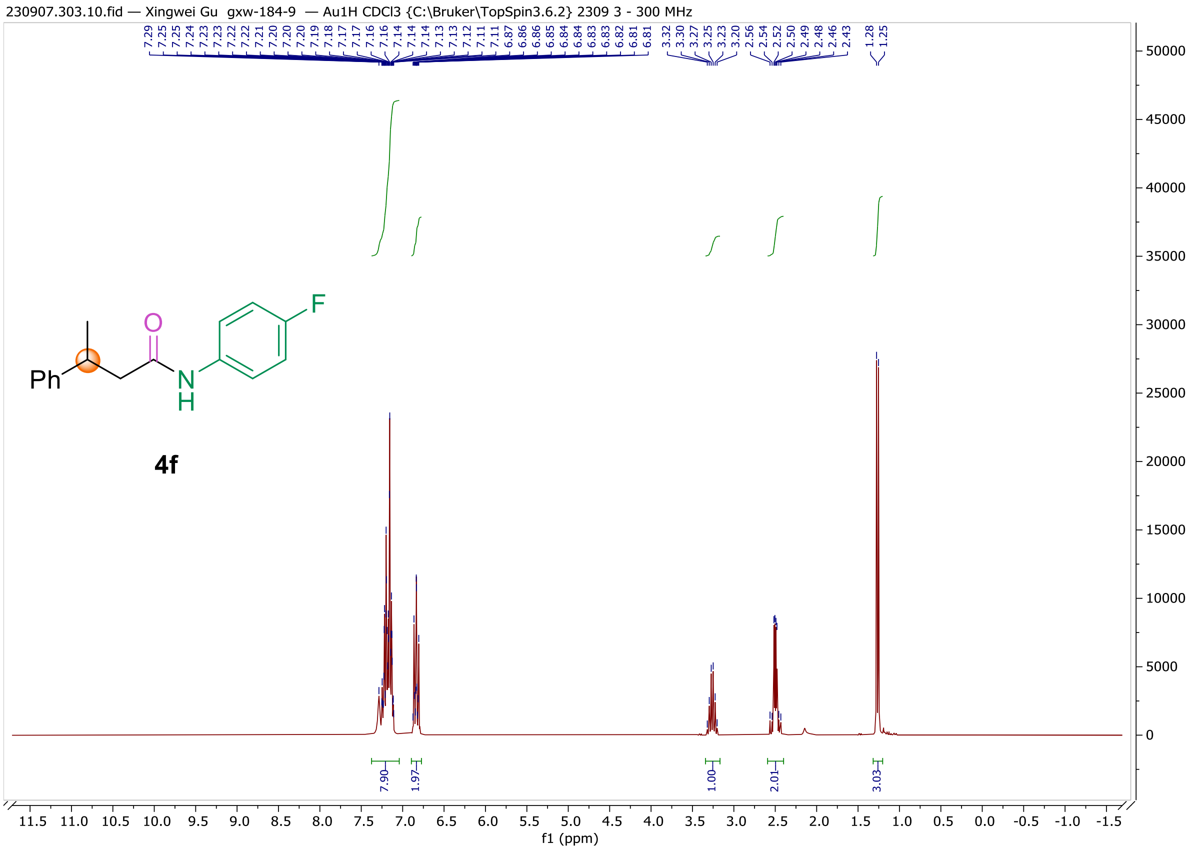
230907.303.10.fid — Xingwei Gu gxw-184-9 — Au1H CDCl3 {C:\Bruker\TopSpin3.6.2} 2309 3 - 300 MHz
4f
f1 (ppm)

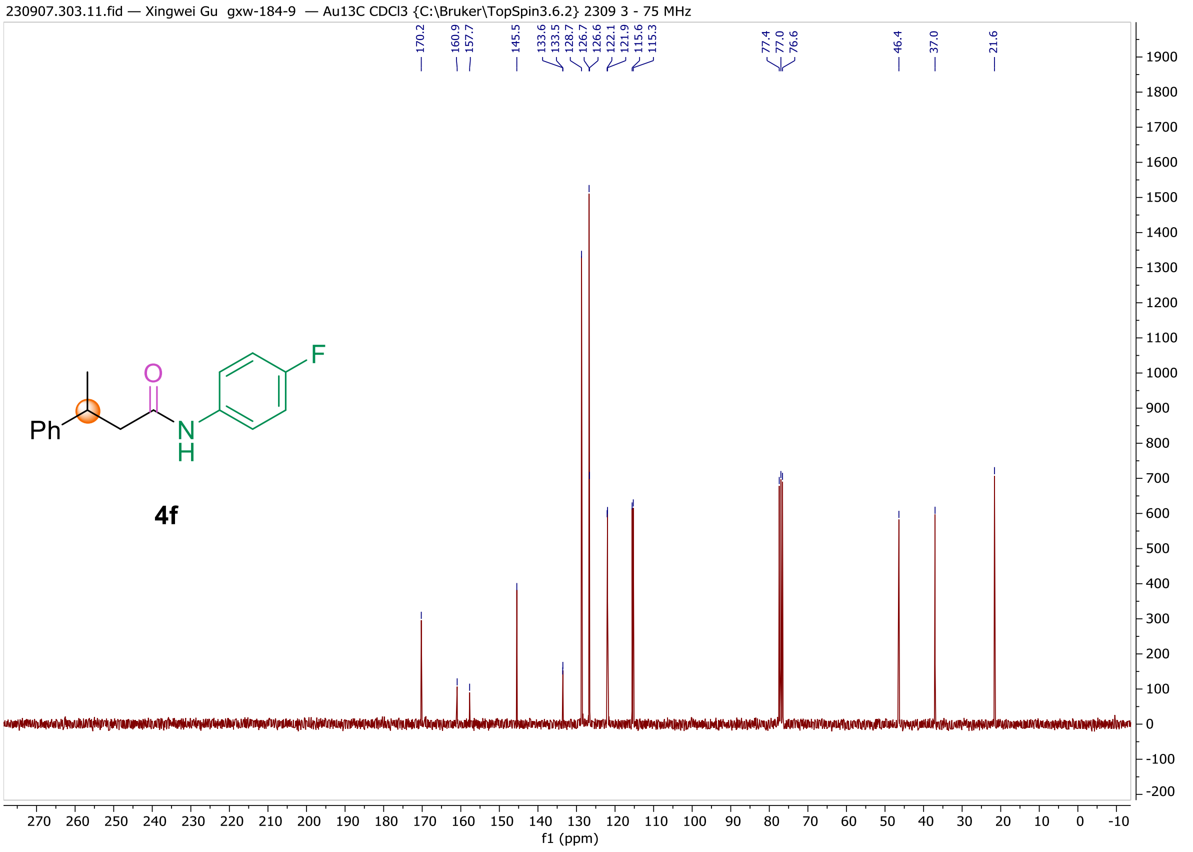
230907.303.11.fid — Xingwei Gu gxw-184-9 — Au13C CDCl3 {C:\Bruker\TopSpin3.6.2} 2309 3 - 75 MHz
4f
f1 (ppm)

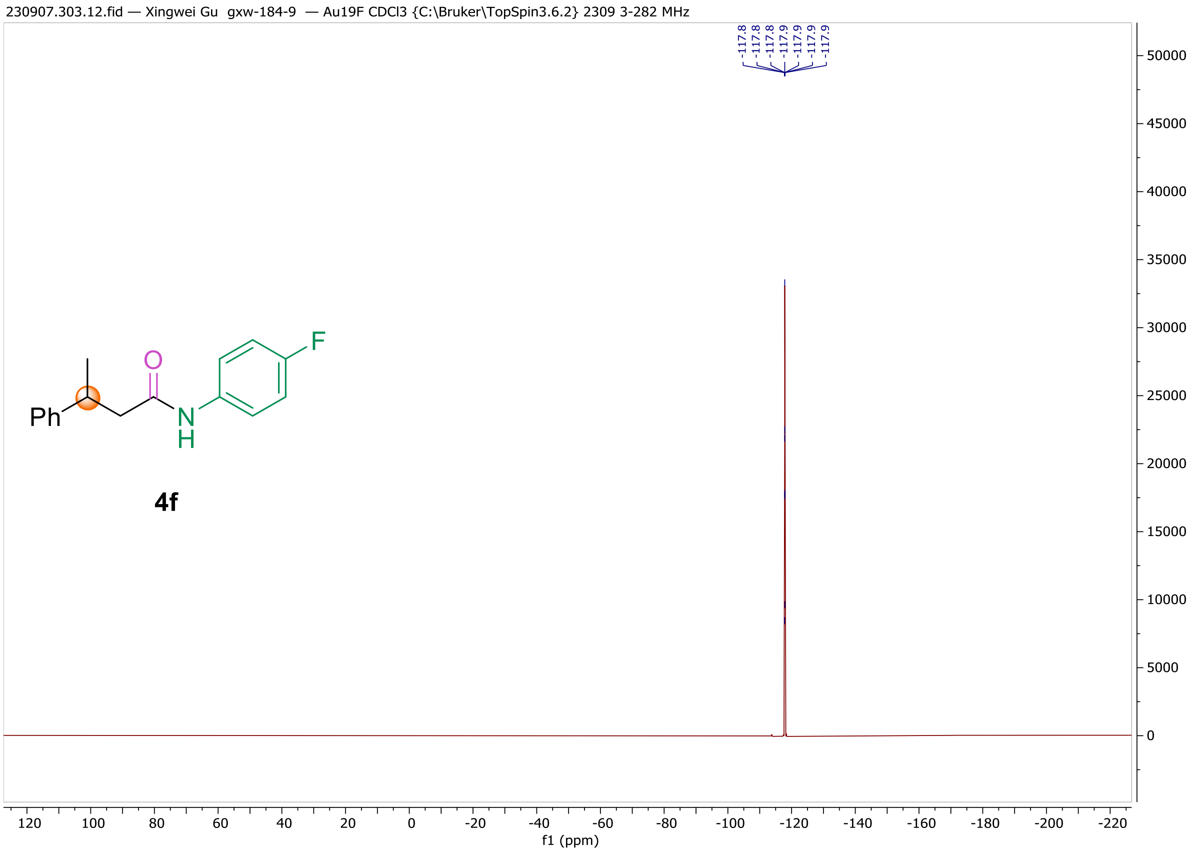

230907.303.12.fid — Xingwei Gu gxw-184-9 — Au19F CDCl3 {C:\Bruker\TopSpin3.6.2} 2309 3-282 MHz
-117.8
-117.8
-117.8
-117.9
-117.9
-117.9
-117.9
Ph
O
N
H
F
f1 (ppm)


**4f**

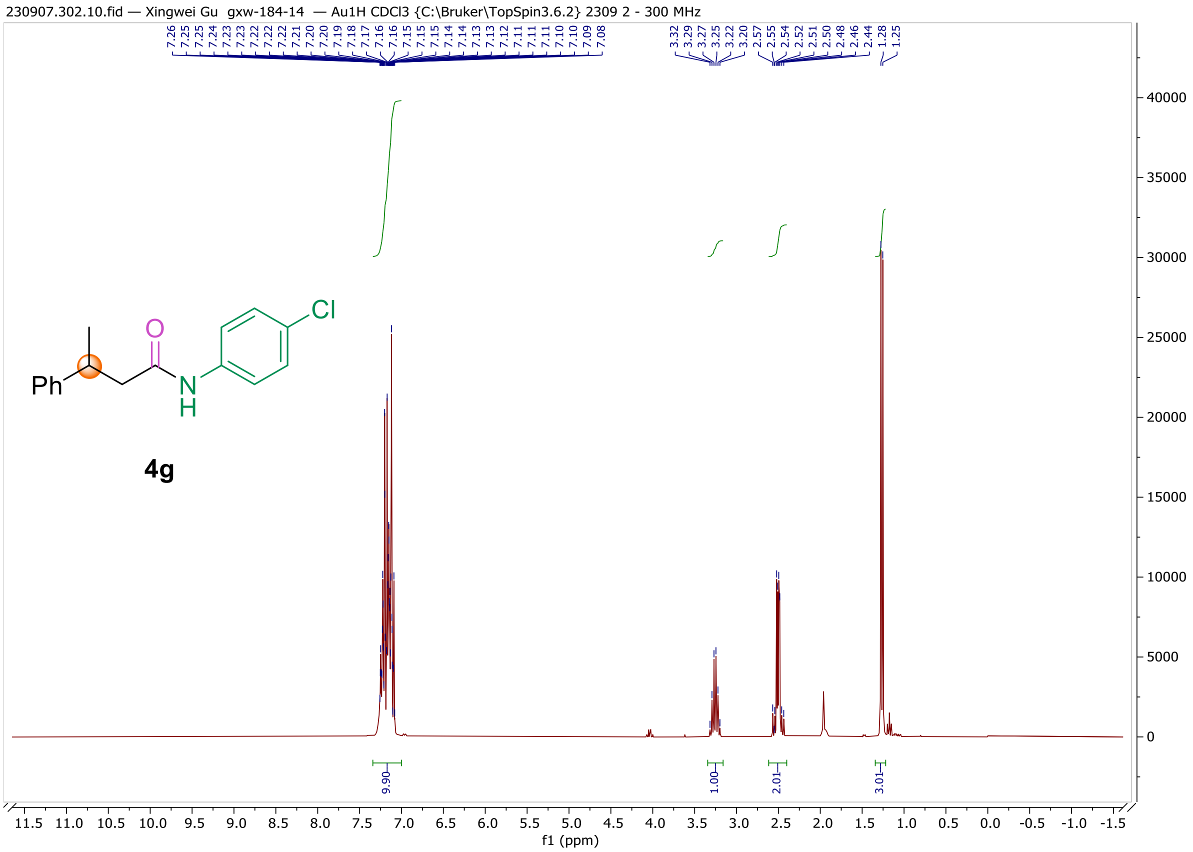
230907.302.10.fid — Xingwei Gu gxw-184-14 — Au1H CDCl3 {C:\Bruker\TopSpin3.6.2} 2309 2 - 300 MHz
Ph
O
N
H
Cl
4g
9.90
1.00
2.01
3.01
f1 (ppm)

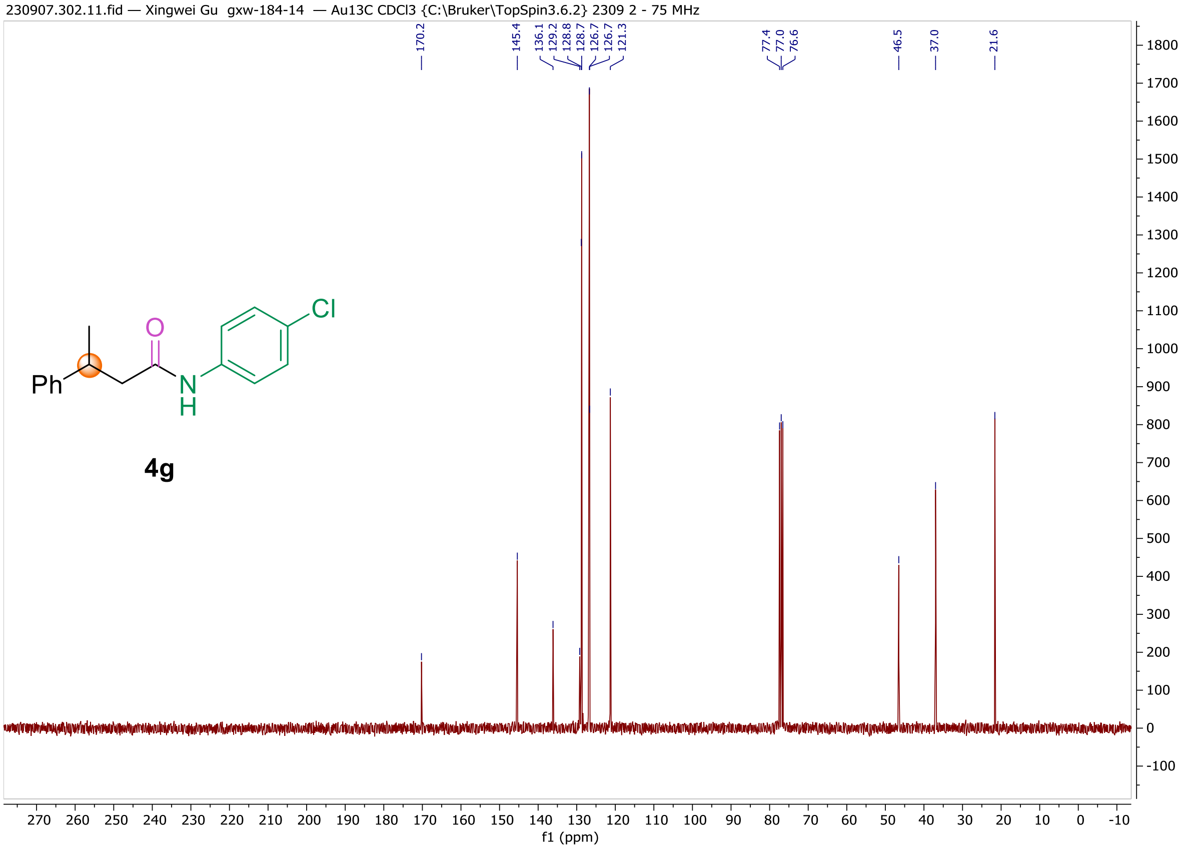
230907.302.11.fid — Xingwei Gu gxw-184-14 — Au13C CDCl3 {C:\Bruker\TopSpin3.6.2} 2309 2 - 75 MHz
170.2
145.4
136.1
129.2
128.8
128.7
126.7
126.7
121.3
77.4
77.0
76.6
46.5
37.0
21.6
Ph
O
N
H
Cl
4g
f1 (ppm)

230908.411.10.fid — Xingwei Gu gxw-184-12 — Au1H CDCl3 {C:\Bruker\TopSpin3.6.2} 2309 11 - 300 MHz

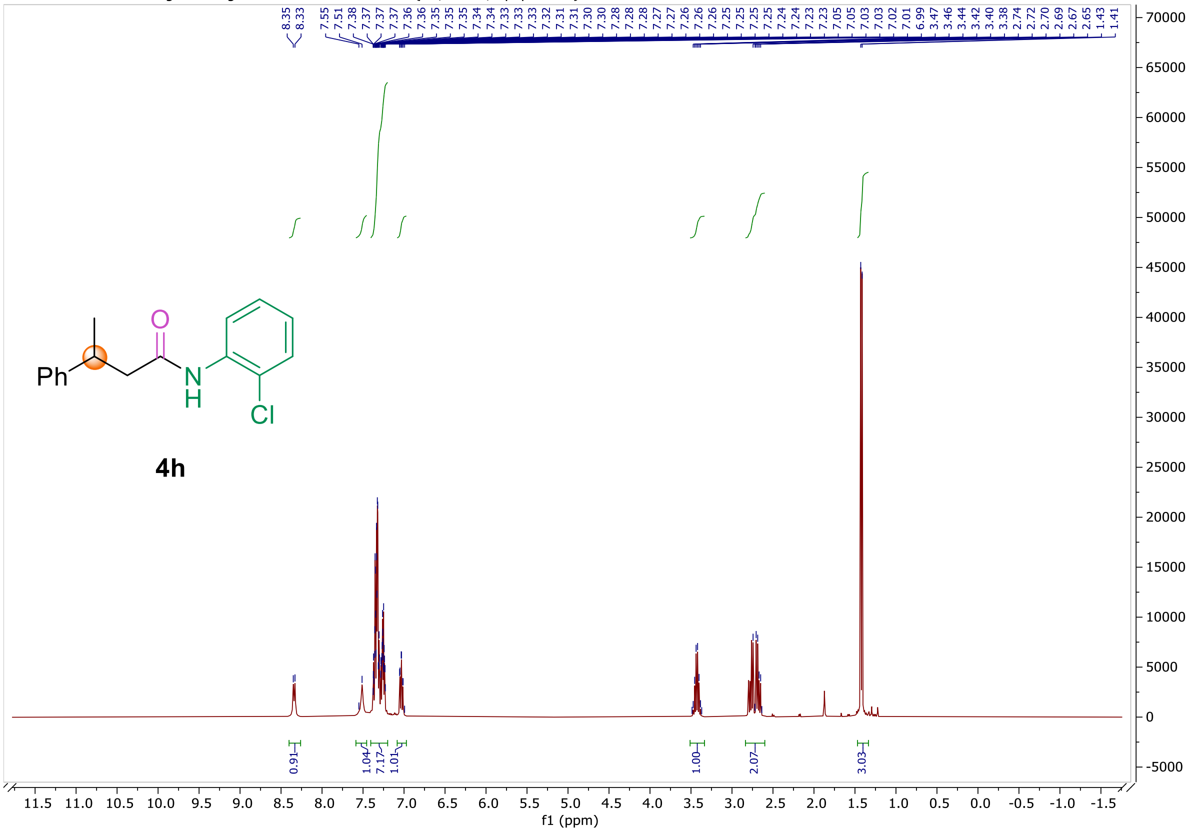


230908.411.11.fid — Xingwei Gu gxw-184-12 — Au13C CDCl3 {C:\Bruker\TopSpin3.6.2} 2309 11 - 75 MHz

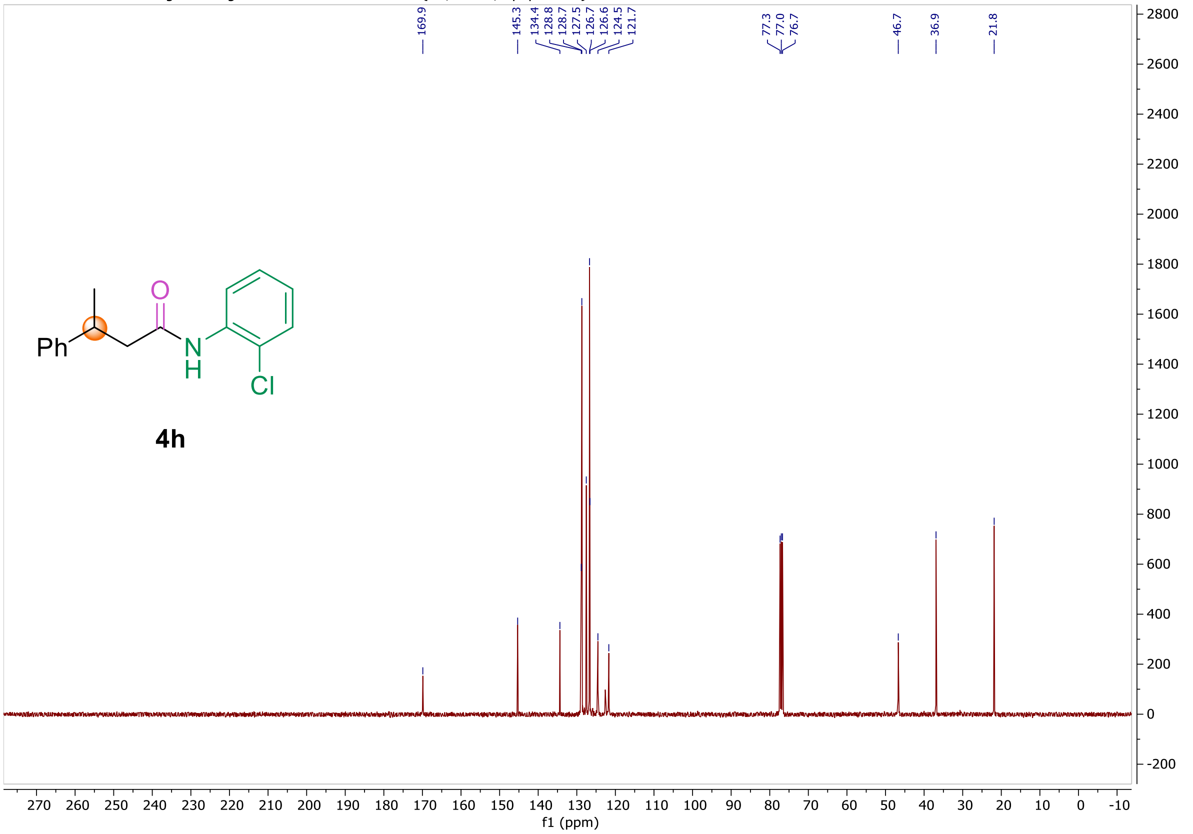

231005.313.10.fid — Xingwei Gu gxw-194-1 — Au1H CDCl3 {C:\Bruker\TopSpin3.6.2} 2310 13 - 300 MHz

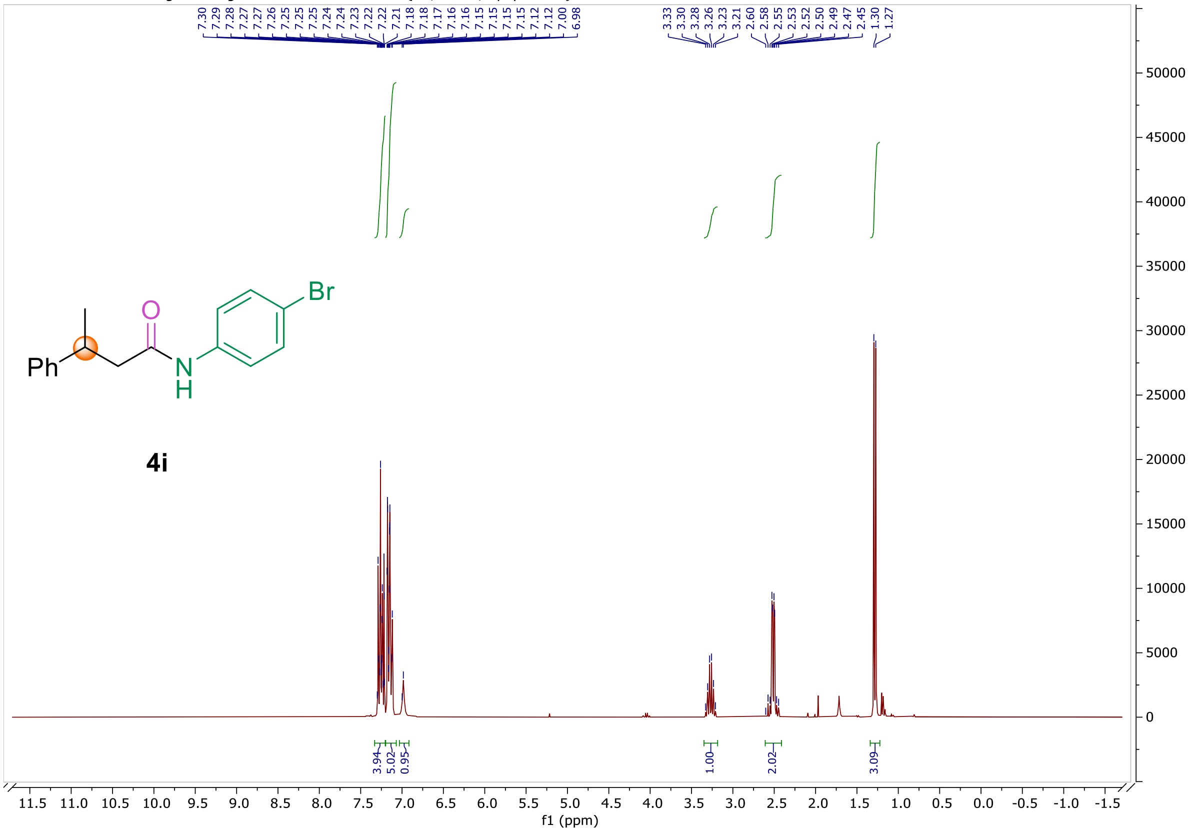


231001.303.11.fid — Xingwei Gu, gxw-194-1 — Au13C CDCl3 {C:\Bruker\TopSpin3.6.2} 2310 3 - 75 MHz

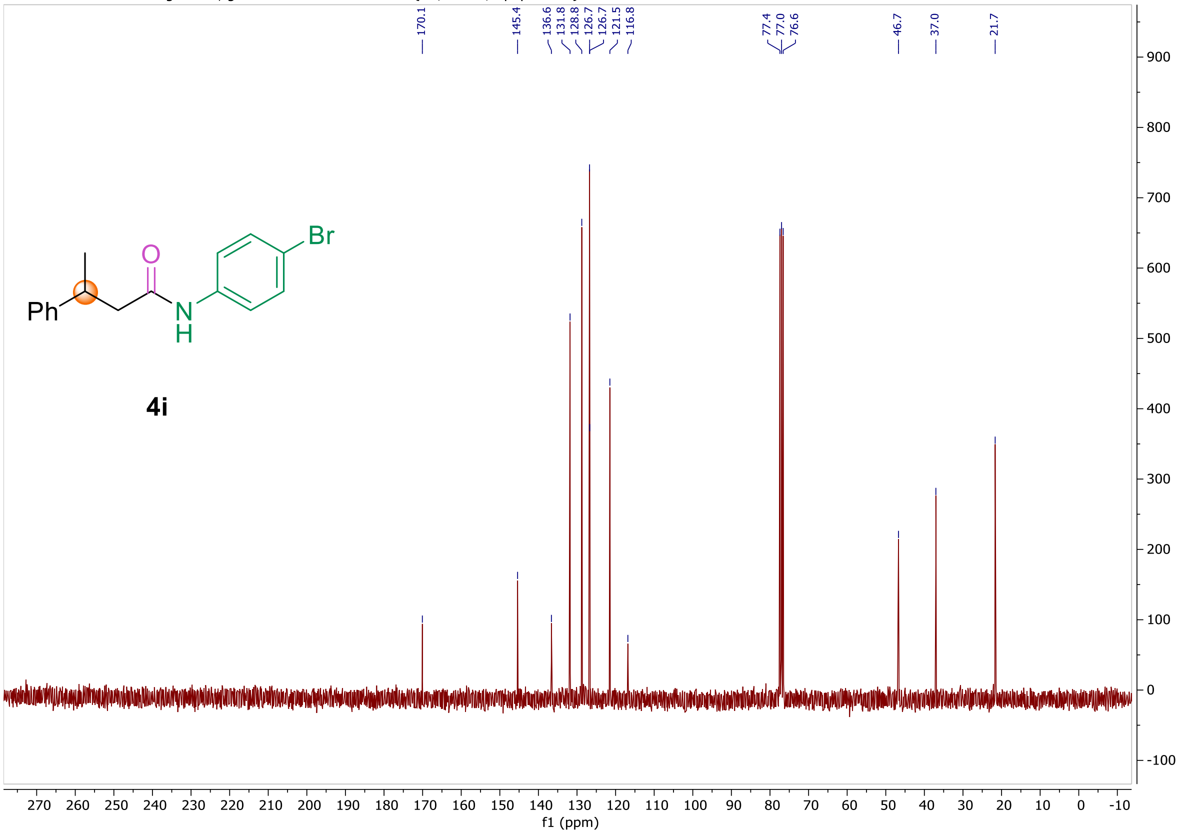

230904.f302.10.fid — Xingwei Gu gxw-184-4 — PROTON CDCl3 {C:\Bruker\TopSpin3.6.2} 2309 2 - 300 MHz

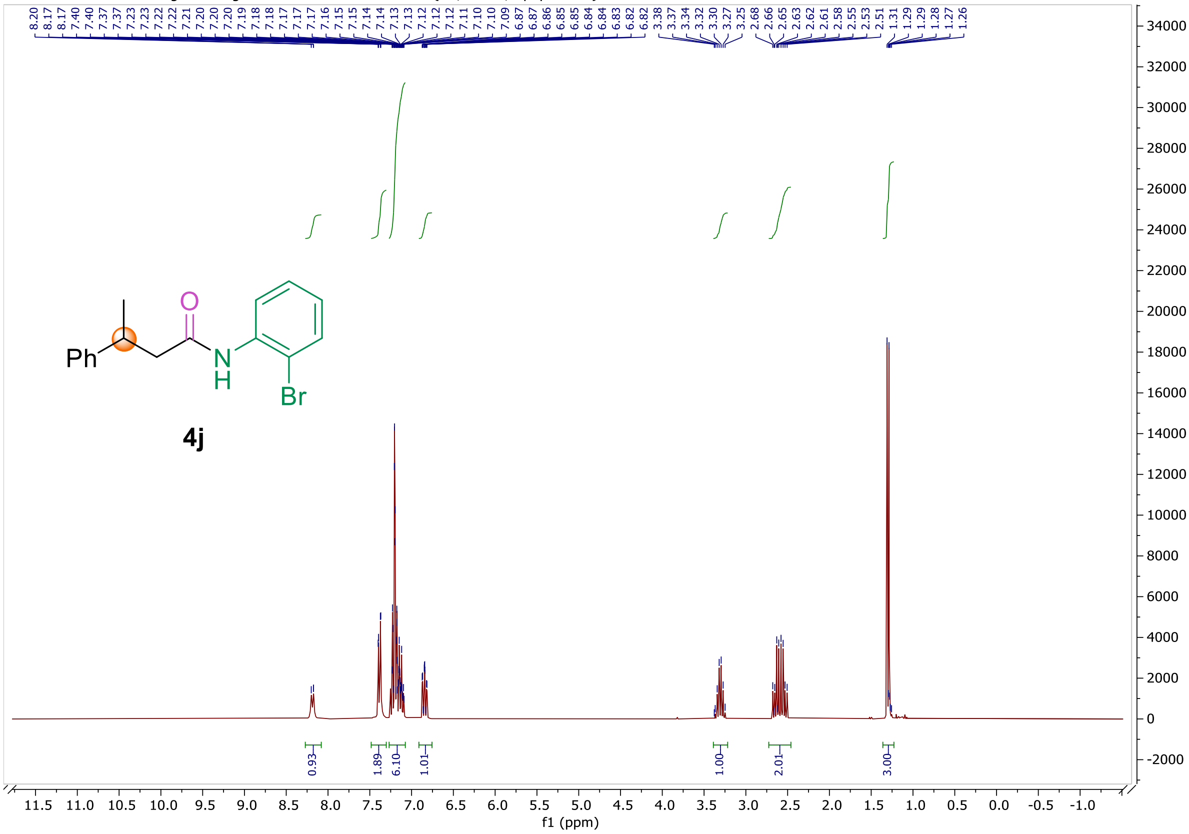


230904.f302.11.fid — Xingwei Gu gxw-184-4 — Au13C CDCl3 {C:\Bruker\TopSpin3.6.2} 2309 2 - 75 MHz

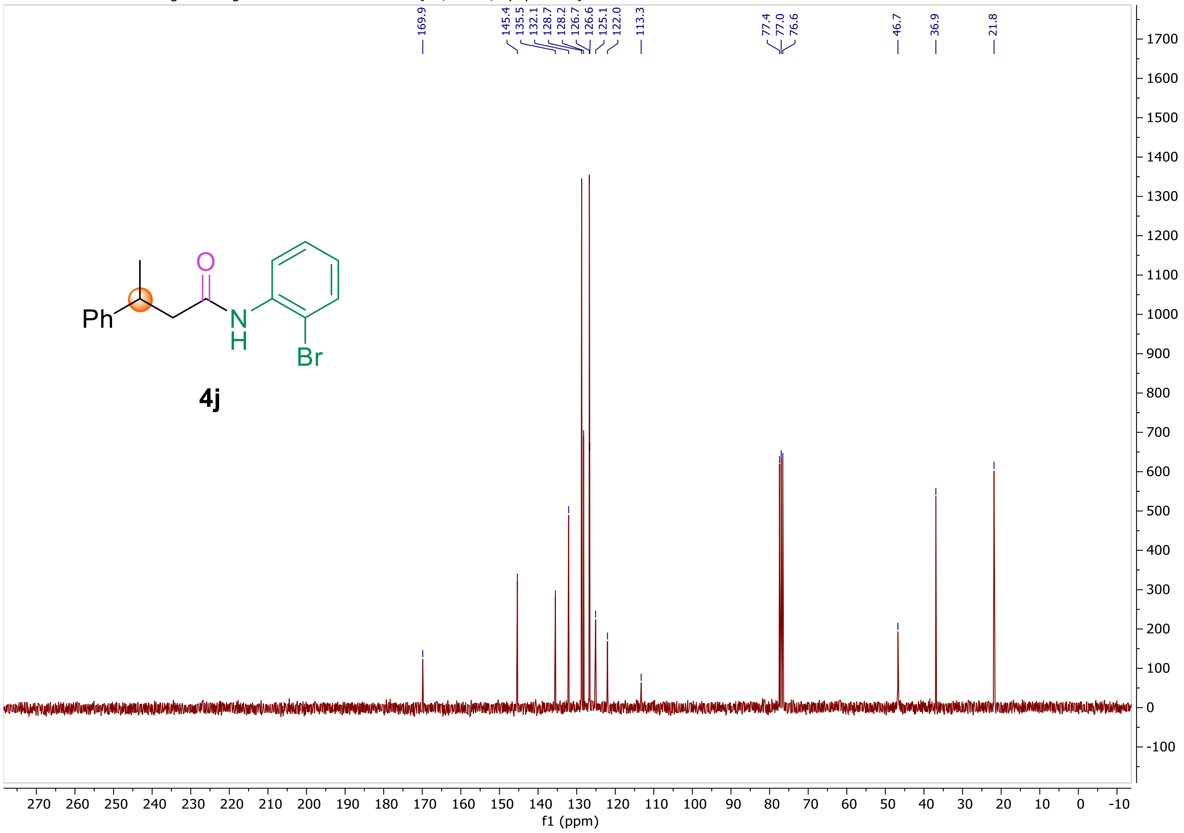

230904.f304.10.fid — Xingwei Gu gxw-184-8 — PROTON CDCl3 {C:\Bruker\TopSpin3.6.2} 2309 4 - 300 MHz

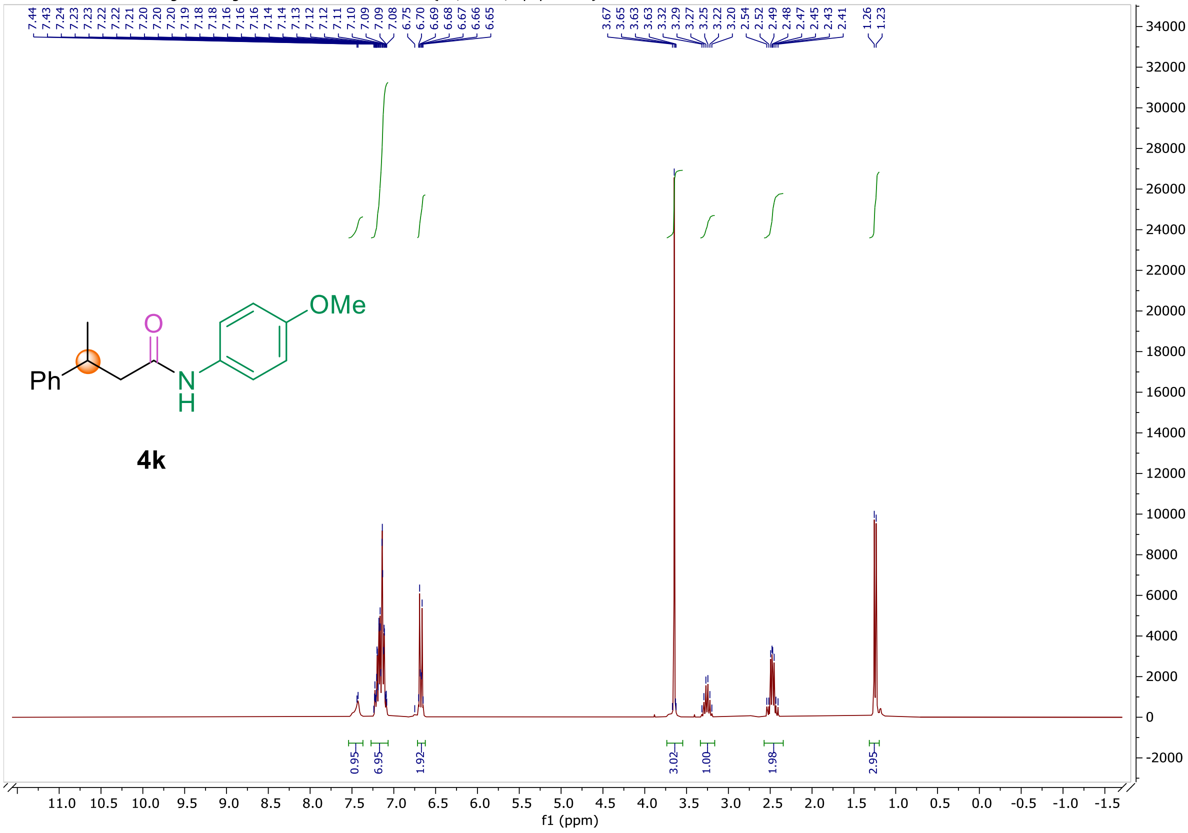


230904.f304.11.fid — Xingwei Gu gxw-184-8 — Au13C CDCl3 {C:\Bruker\TopSpin3.6.2} 2309 4 - 75 MHz

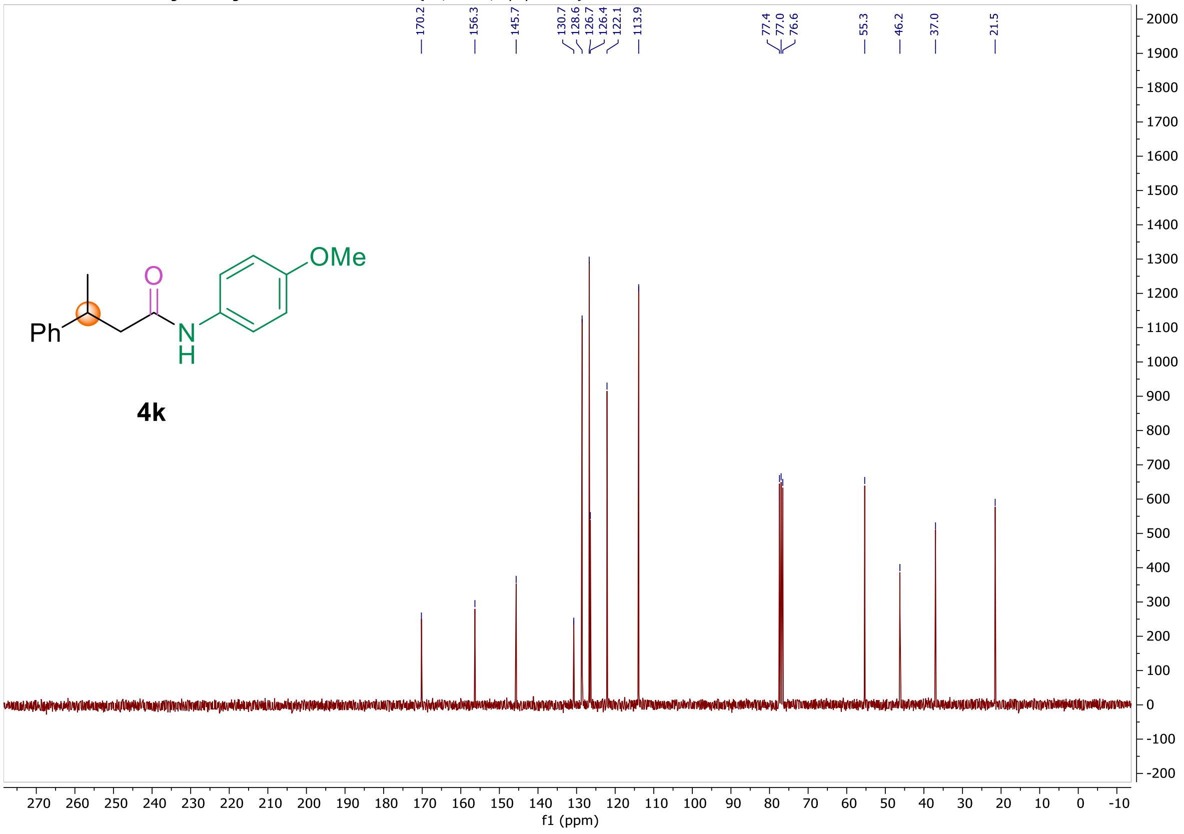

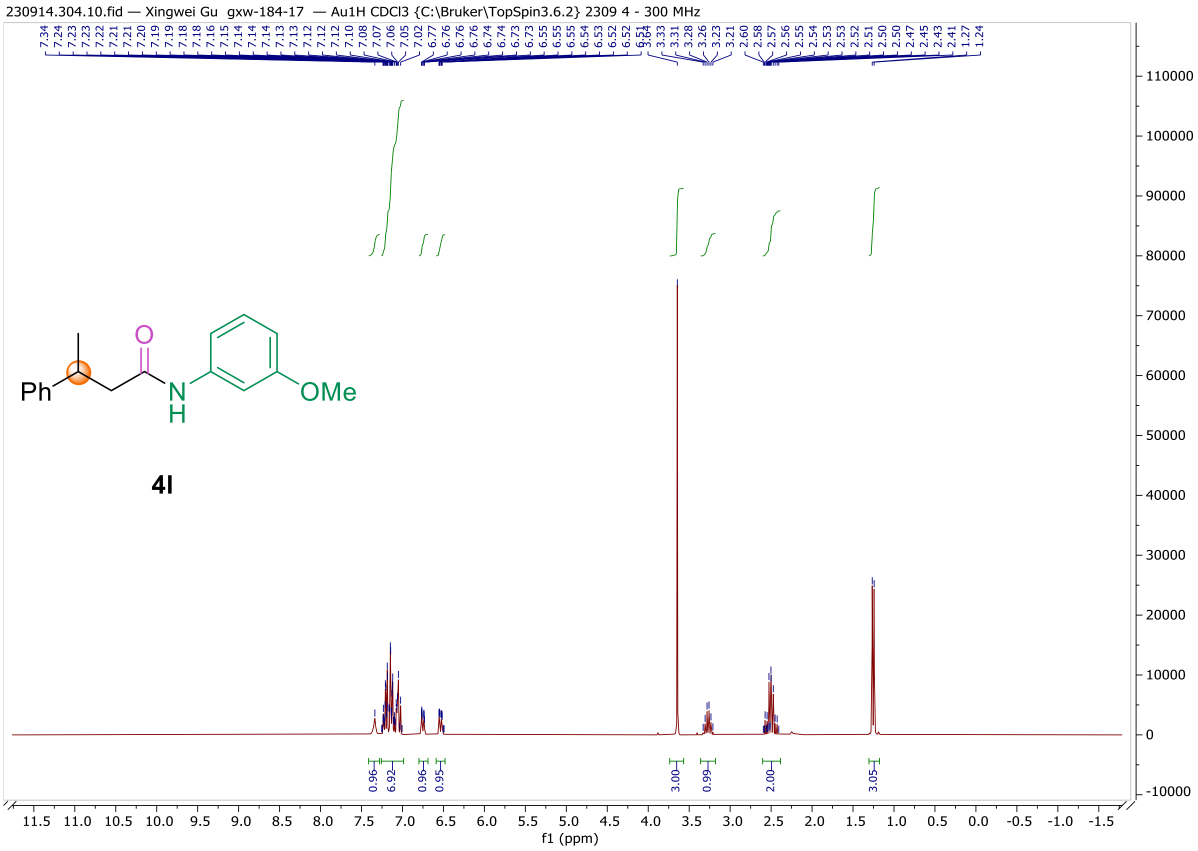
230914.304.10.fid — Xingwei Gu gxw-184-17 — Au1H CDCl3 {C:\Bruker\TopSpin3.6.2} 2309 4 - 300 MHz
7.34 7.24 7.23 7.23 7.22 7.21 7.21 7.20 7.19 7.19 7.18 7.18 7.16 7.15 7.15 7.14 7.14 7.13 7.13 7.12 7.12 7.12 7.10 7.08 7.07 7.06 7.05 7.02 6.77 6.76 6.76 6.74 6.74 6.73 6.73 6.55 6.55 6.55 6.54 6.53 6.52 6.52 6.51 3.64 3.33 3.31 3.28 3.26 3.23 3.21 2.60 2.58 2.57 2.56 2.55 2.54 2.53 2.53 2.52 2.51 2.50 2.50 2.47 2.45 2.43 2.41 1.27 1.24
Ph
O
N
H
OMe
4l
0.96 6.92 0.96 0.95 3.00 0.99 2.00 3.05
f1 (ppm)

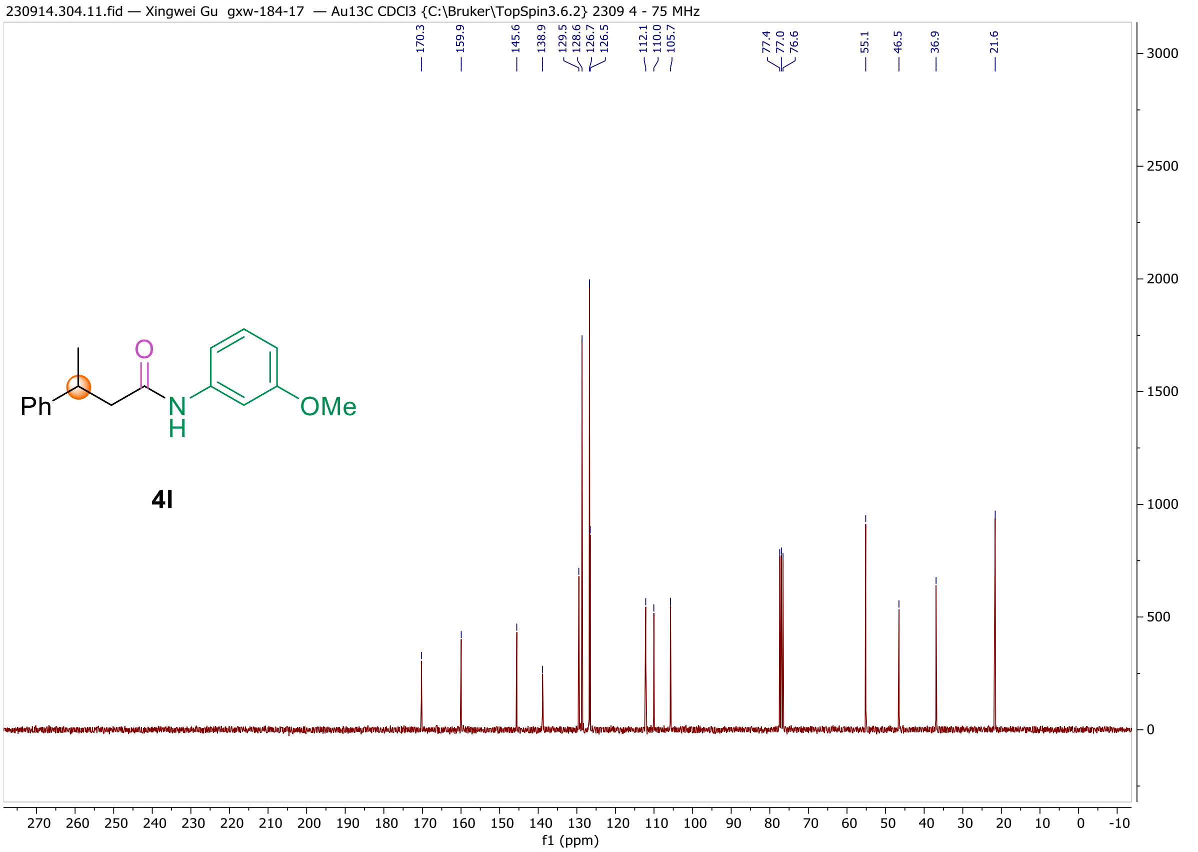
230914.304.11.fid — Xingwei Gu gxw-184-17 — Au13C CDCl3 {C:\Bruker\TopSpin3.6.2} 2309 4 - 75 MHz
170.3 159.9 145.6 138.9 129.5 128.6 126.7 126.5 112.1 110.0 105.7 77.4 77.0 76.6 55.1 46.5 36.9 21.6
Ph
O
N
H
OMe
4l
f1 (ppm)

230904.f303.10.fid — Xingwei Gu gxw-184-6 — PROTON CDCl3 {C:\Bruker\TopSpin3.6.2} 2309 3 - 300 MHz

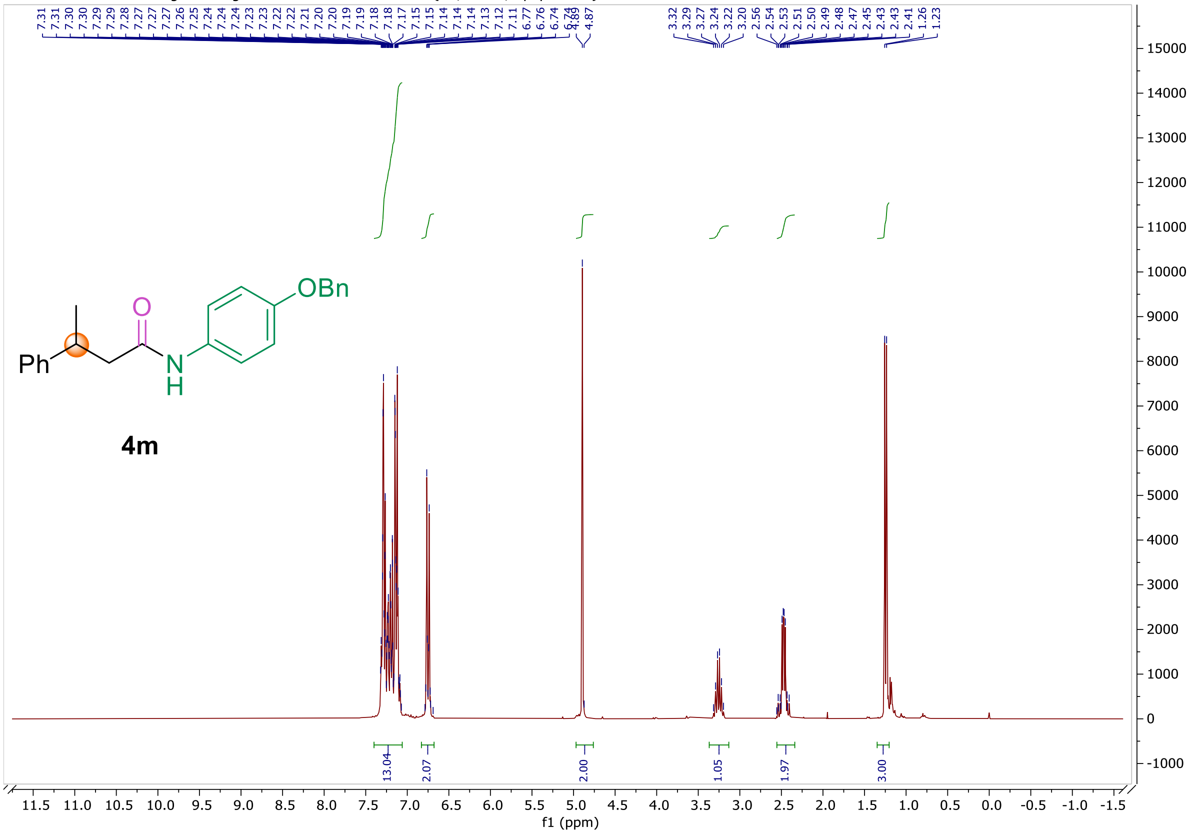

230904.f303.11.fid — Xingwei Gu gxw-184-6 — Au13C CDCl3 {C:\Bruker\TopSpin3.6.2} 2309 3 - 75 MHz

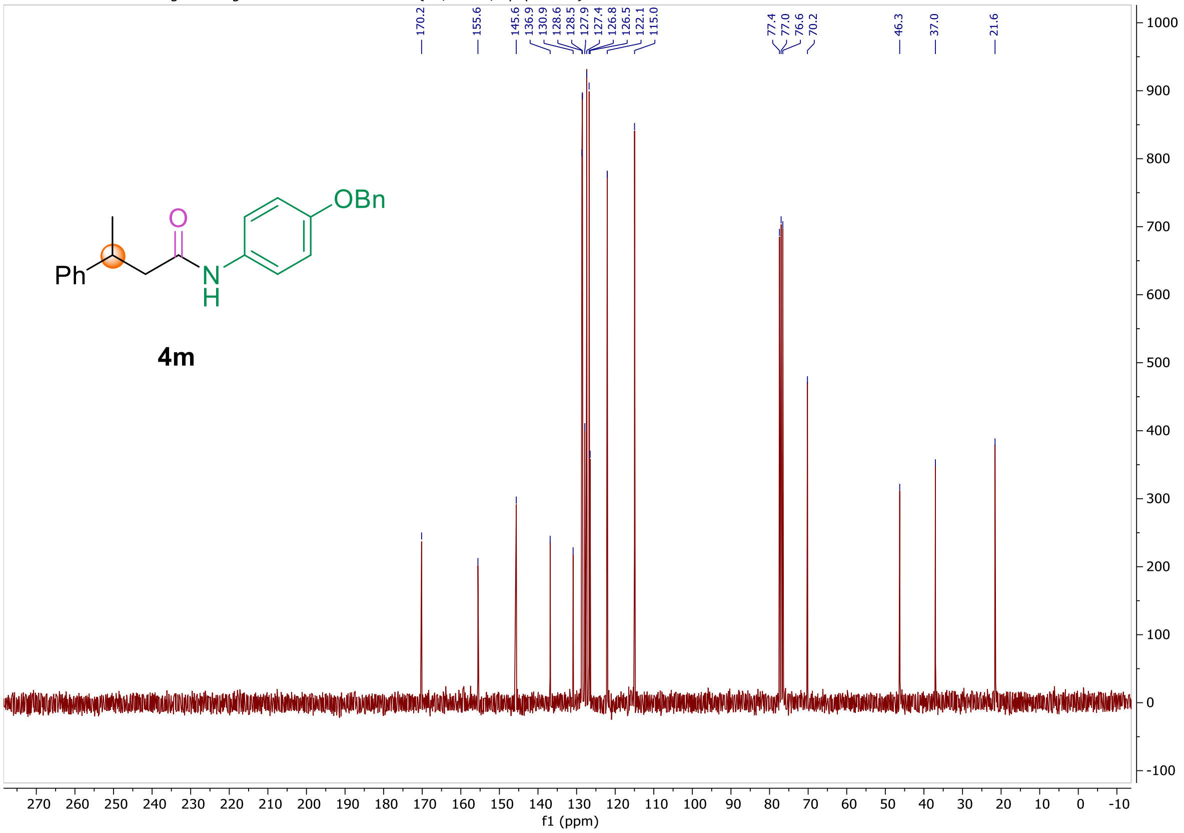

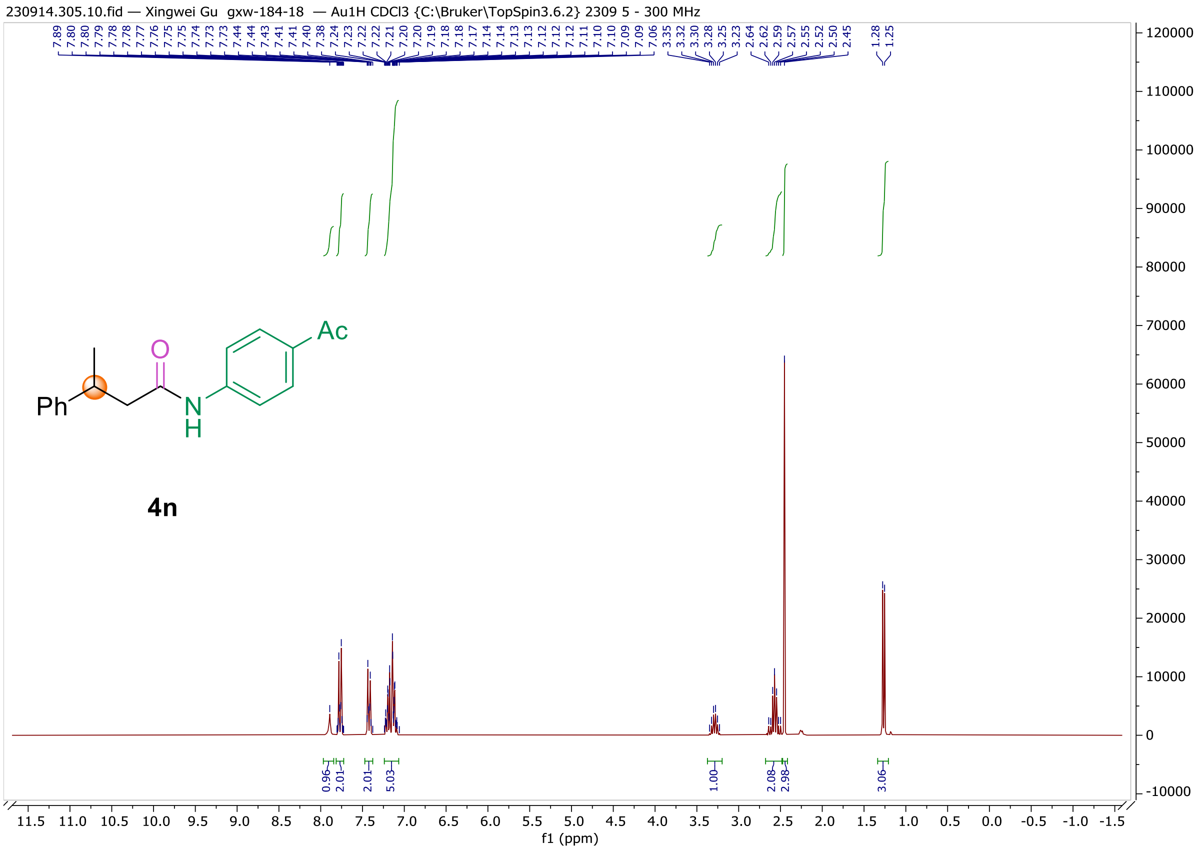
230914.305.10.fid — Xingwei Gu gxw-184-18 — Au1H CDCl3 {C:\Bruker\TopSpin3.6.2} 2309 5 - 300 MHz
4n
Ac
O
Ph
N
H
f1 (ppm)

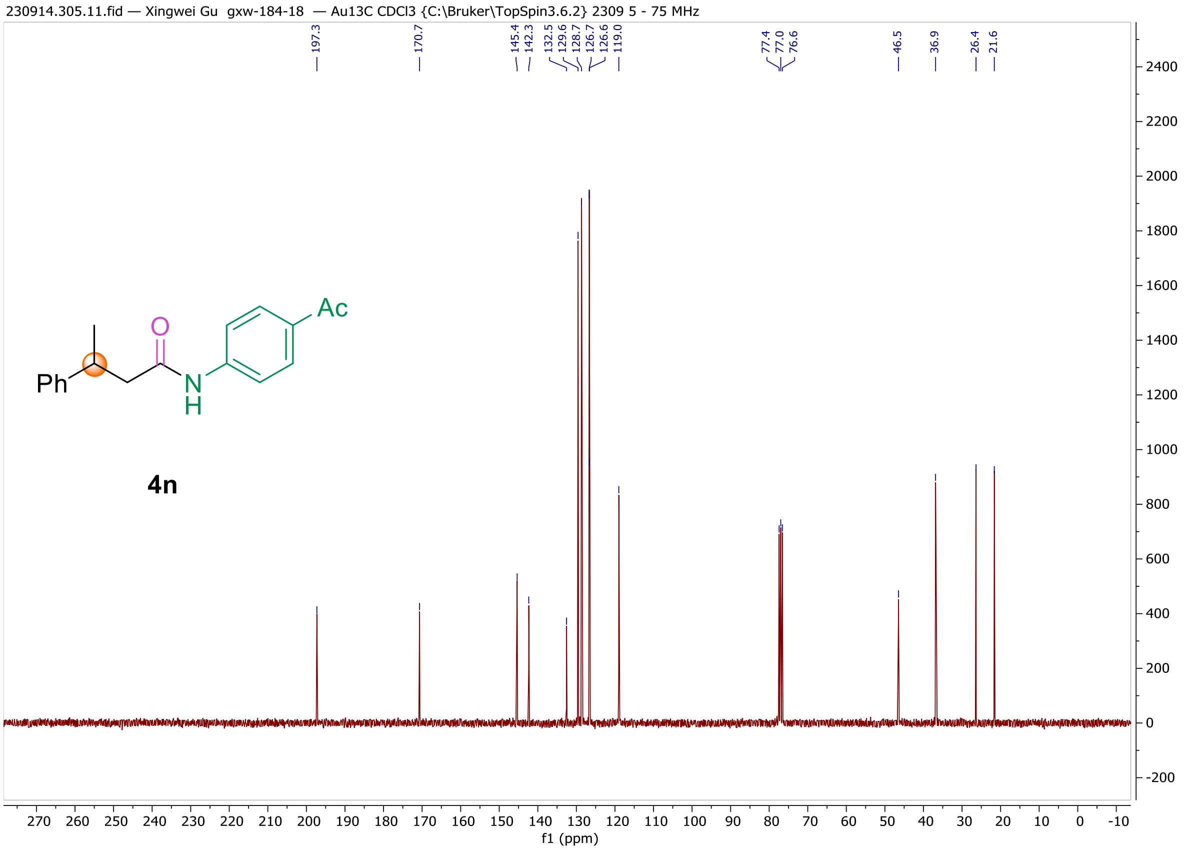
230914.305.11.fid — Xingwei Gu gxw-184-18 — Au13C CDCl3 {C:\Bruker\TopSpin3.6.2} 2309 5 - 75 MHz
197.3
170.7
145.4
142.3
132.5
129.6
128.7
126.7
126.6
119.0
77.4
77.0
76.6
46.5
36.9
26.4
21.6
4n
Ac
O
Ph
N
H
f1 (ppm)

230914.327.10.fid — Xingwei Gu, gxw-184-5 — Au1H CDCl3 {C:\Bruker\TopSpin3.6.2} 2309 27 - 300 MHz

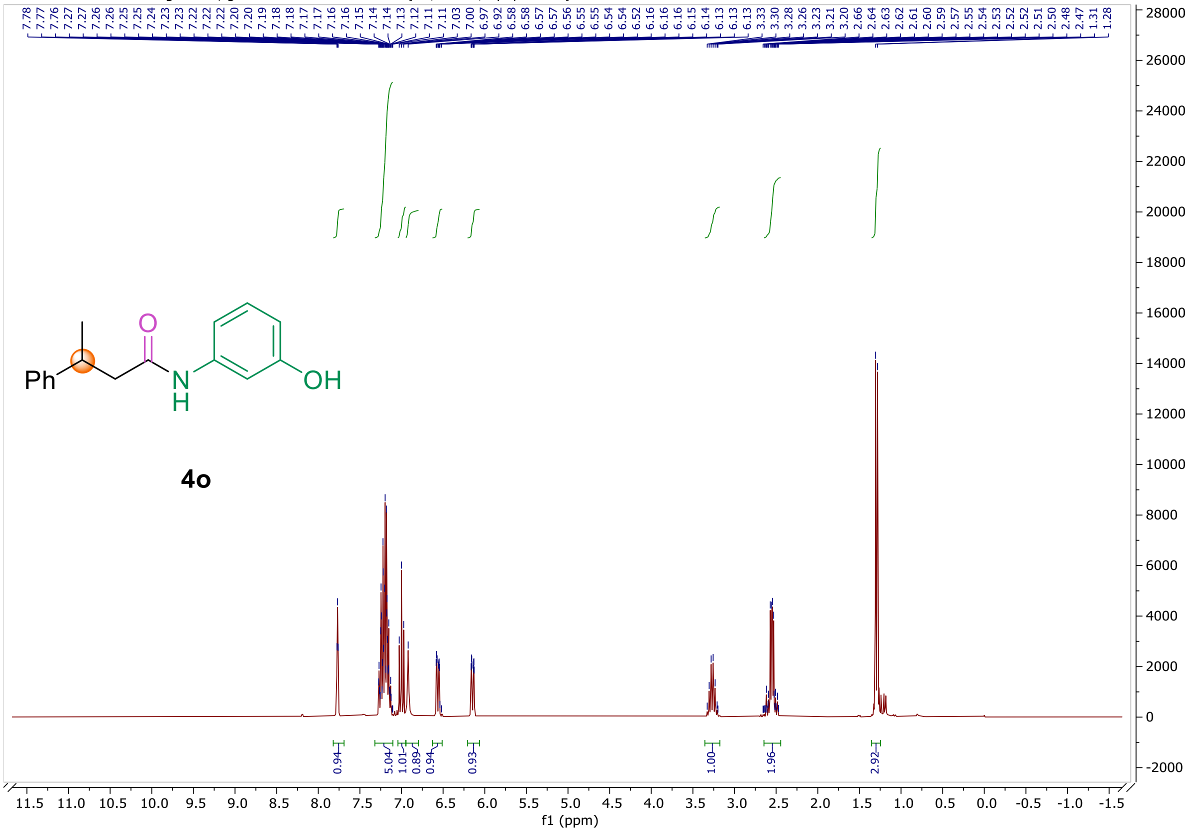


230914.327.11.fid — Xingwei Gu, gxw-184-5 — Au13C CDCl3 {C:\Bruker\TopSpin3.6.2} 2309 27 - 75 MHz

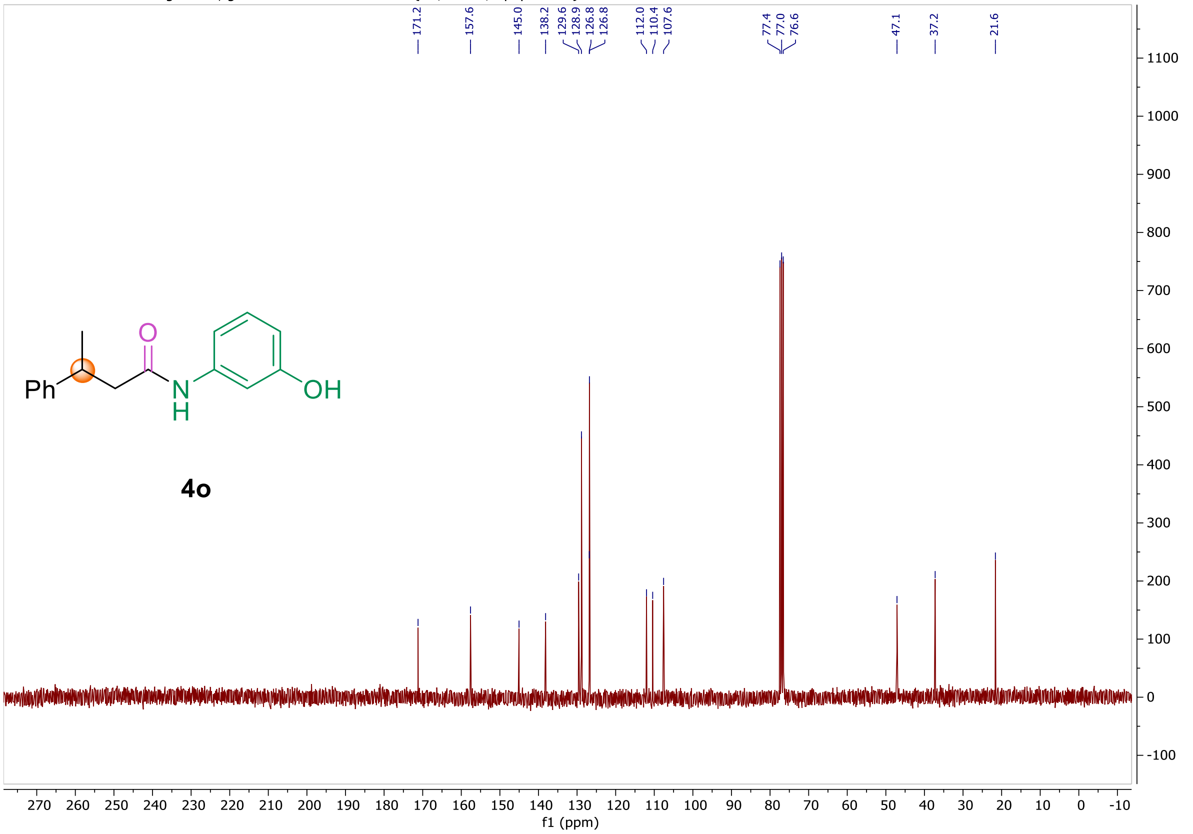

231005.310.10.fid — Xingwei Gu gxw-194-3 — Au1H CDCl3 {C:\Bruker\TopSpin3.6.2} 2310 10 - 300 MHz

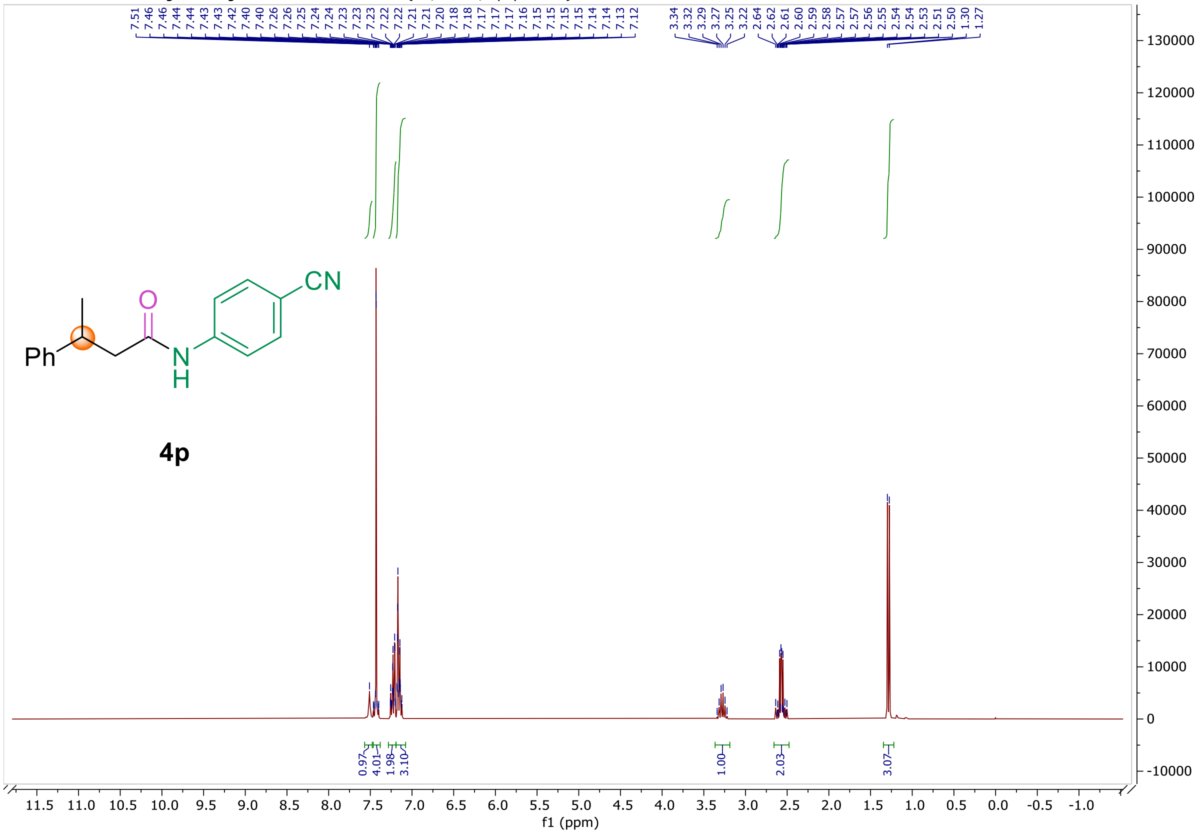

4p

231005.310.11.fid — Xingwei Gu gxw-194-3 — Au13C CDCl3 {C:\Bruker\TopSpin3.6.2} 2310 10 - 75 MHz

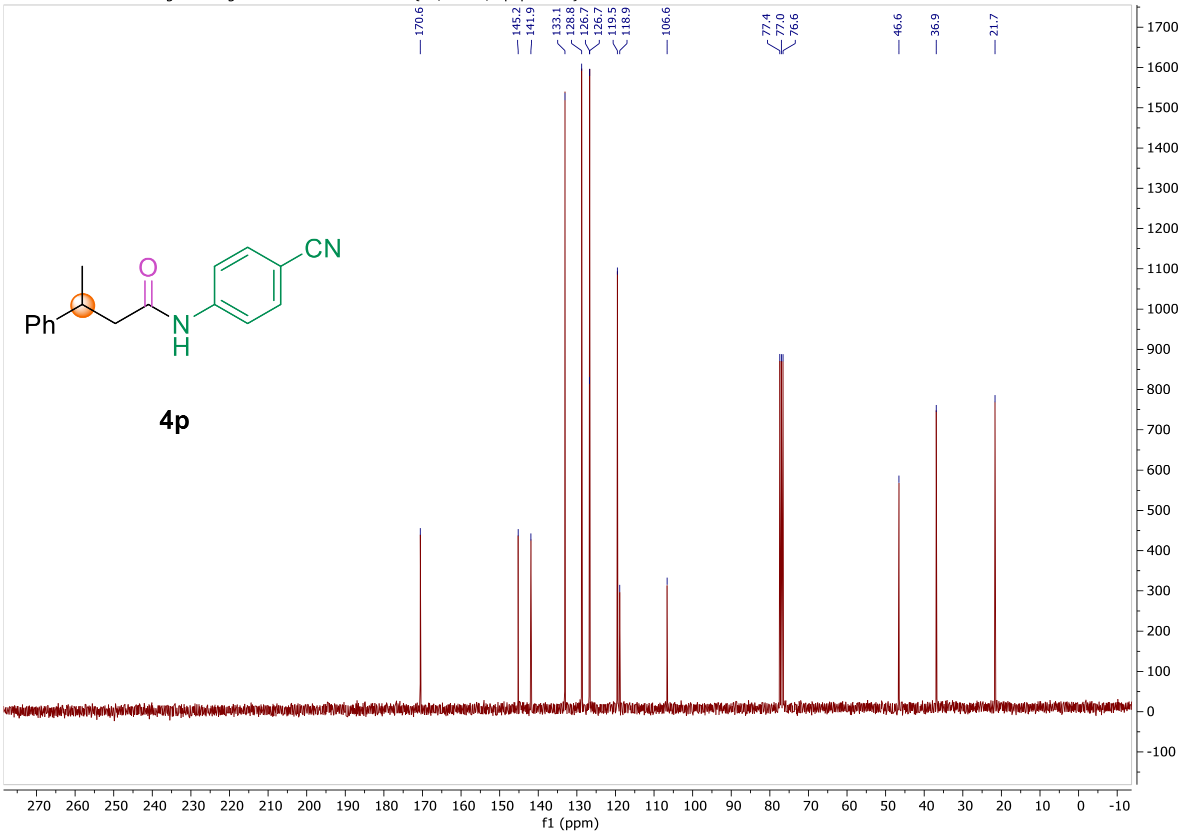

4p

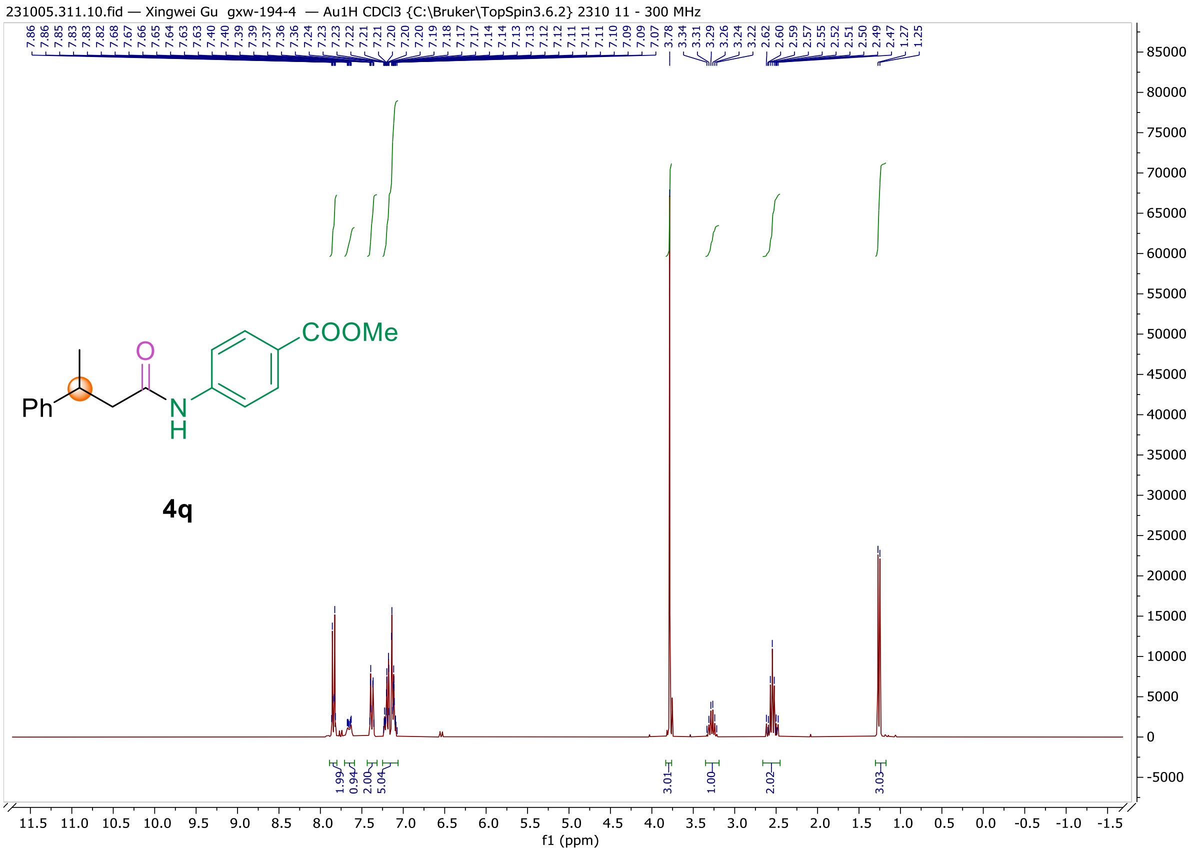
231005.311.10.fid — Xingwei Gu gxw-194-4 — Au1H CDCl3 {C:\Bruker\TopSpin3.6.2} 2310 11 - 300 MHz
7.86 7.86 7.85 7.83 7.83 7.82 7.68 7.67 7.66 7.65 7.64 7.63 7.63 7.40 7.40 7.39 7.39 7.37 7.36 7.36 7.24 7.23 7.23 7.22 7.21 7.21 7.20 7.20 7.20 7.19 7.18 7.17 7.17 7.14 7.14 7.13 7.13 7.12 7.12 7.11 7.11 7.11 7.10 7.09 7.09 7.07 3.78 3.34 3.31 3.29 3.26 3.24 3.22 2.62 2.60 2.59 2.57 2.55 2.52 2.51 2.50 2.49 2.47 1.27 1.25
COOMe
O
Ph
N
H
4q
1.99
0.94
2.00
5.04
3.01
1.00
2.02
3.03
f1 (ppm)

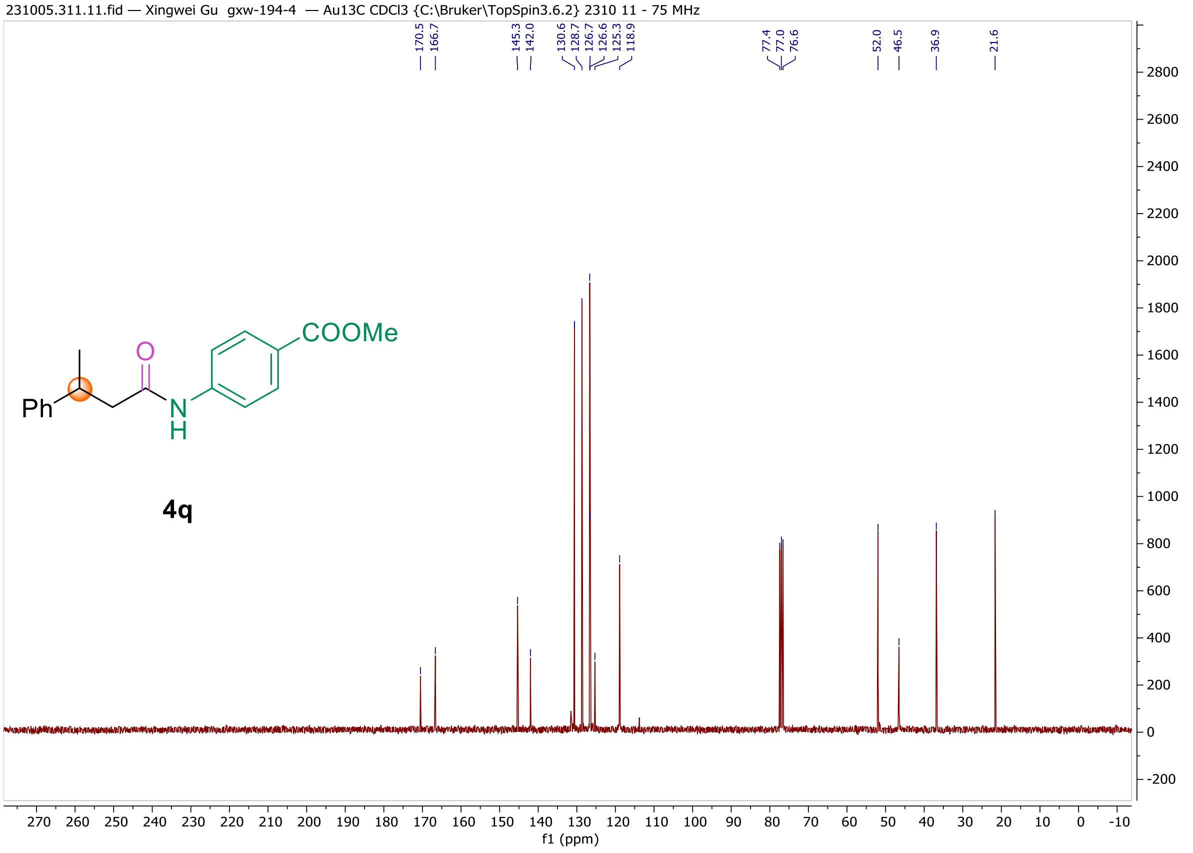
231005.311.11.fid — Xingwei Gu gxw-194-4 — Au13C CDCl3 {C:\Bruker\TopSpin3.6.2} 2310 11 - 75 MHz
170.5 166.7 145.3 142.0 130.6 128.7 126.7 126.6 125.3 118.9 77.4 77.0 76.6 52.0 46.5 36.9 21.6
COOMe
O
Ph
N
H
4q
f1 (ppm)

231009.f312.10.fid — Xingwei Gu gxw-194-9 — Au1H CDCl3 {C:\Bruker\TopSpin3.6.2} 2310 12 - 300 MHz

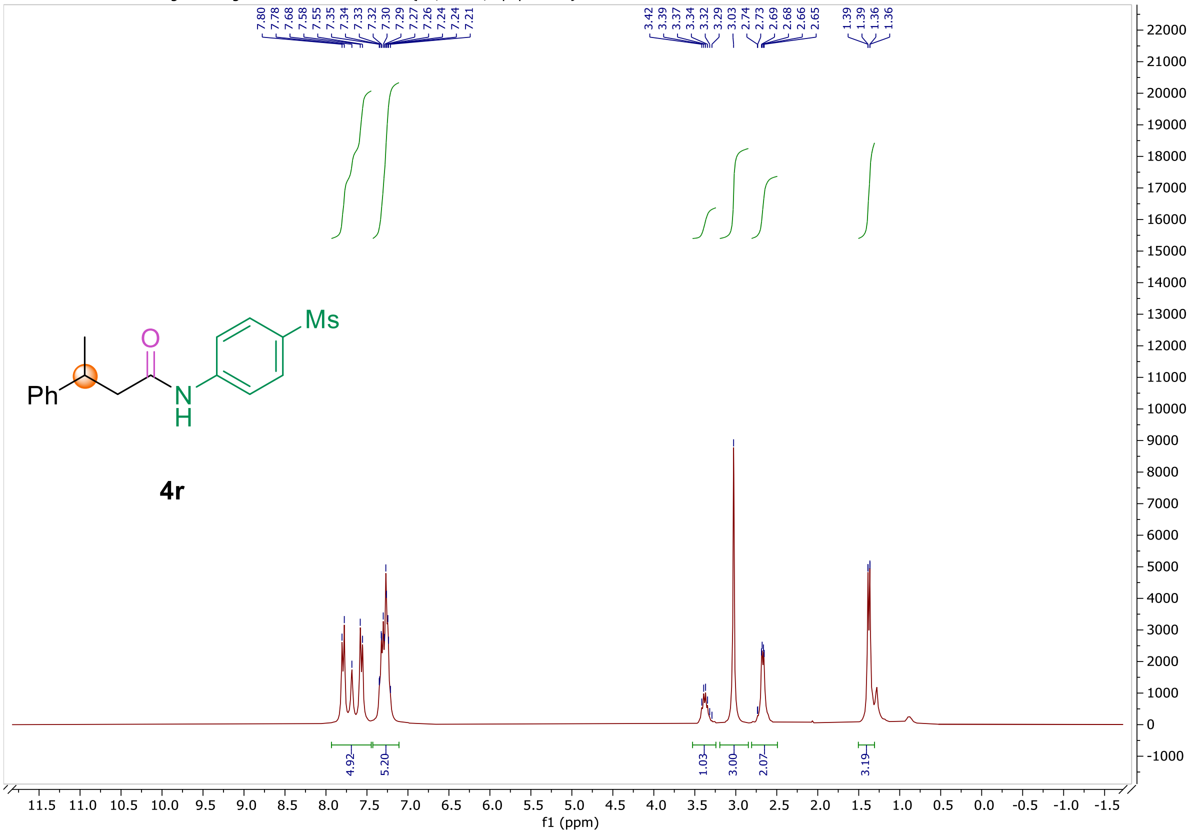


231009.f312.11.fid — Xingwei Gu gxw-194-9 — Au13C CDCl3 {C:\Bruker\TopSpin3.6.2} 2310 12 - 75 MHz

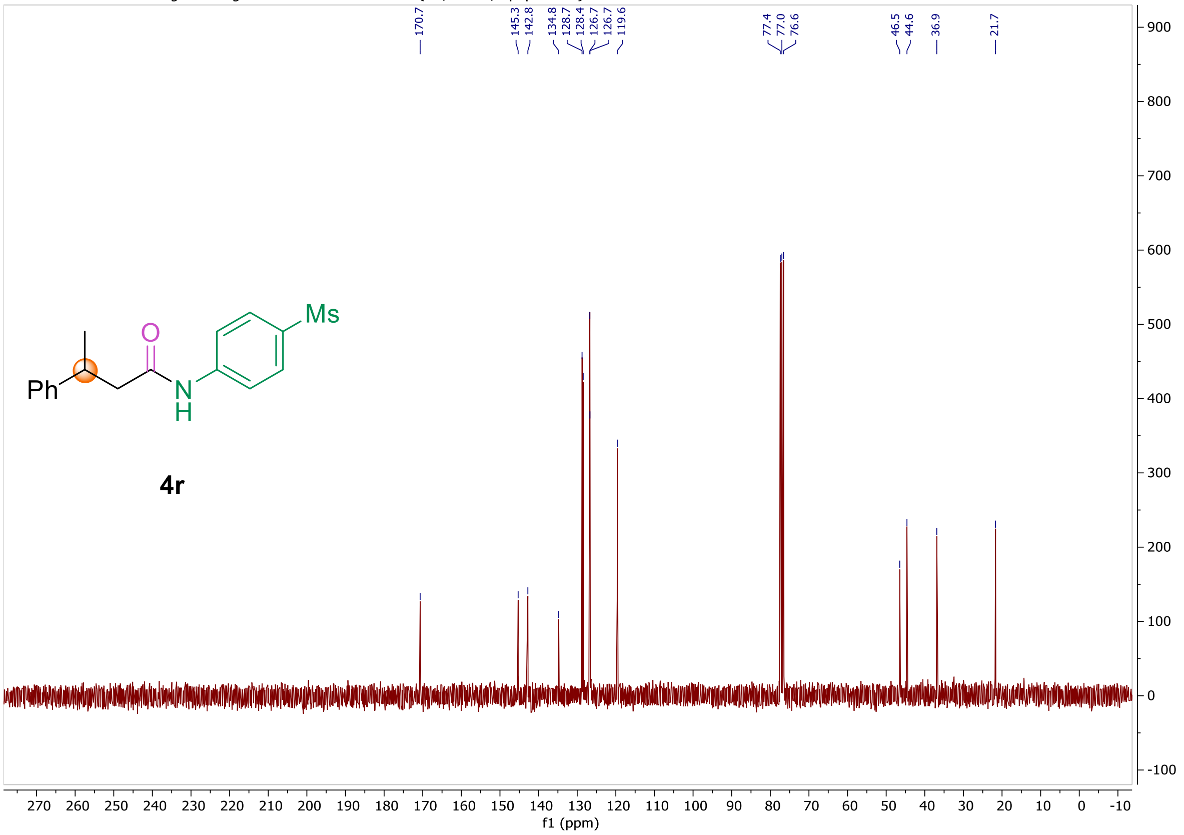

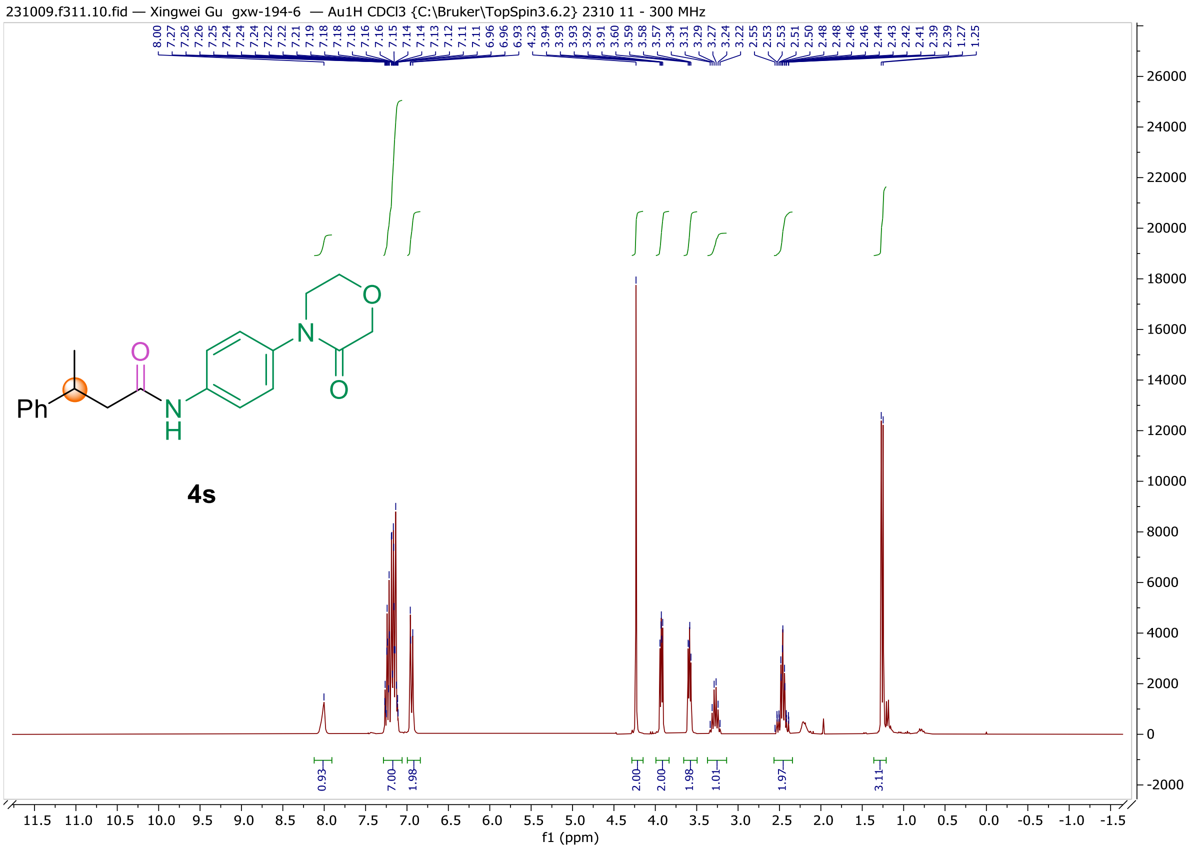
231009.f311.10.fid — Xingwei Gu gxw-194-6 — Au1H CDCl3 {C:\Bruker\TopSpin3.6.2} 2310 11 - 300 MHz
8.00 7.27 7.26 7.26 7.25 7.24 7.24 7.24 7.22 7.22 7.21 7.19 7.18 7.18 7.18 7.16 7.16 7.16 7.15 7.14 7.14 7.13 7.12 7.11 7.11 6.96 6.93 4.23 3.94 3.93 3.93 3.92 3.91 3.60 3.59 3.58 3.57 3.34 3.31 3.29 3.27 3.24 3.22 2.55 2.53 2.53 2.51 2.50 2.48 2.48 2.46 2.46 2.44 2.43 2.42 2.41 2.39 2.39 1.27 1.25
0.93 7.00 1.98 2.00 2.00 1.98 1.01 1.97 3.11
Ph
4s
f1 (ppm)

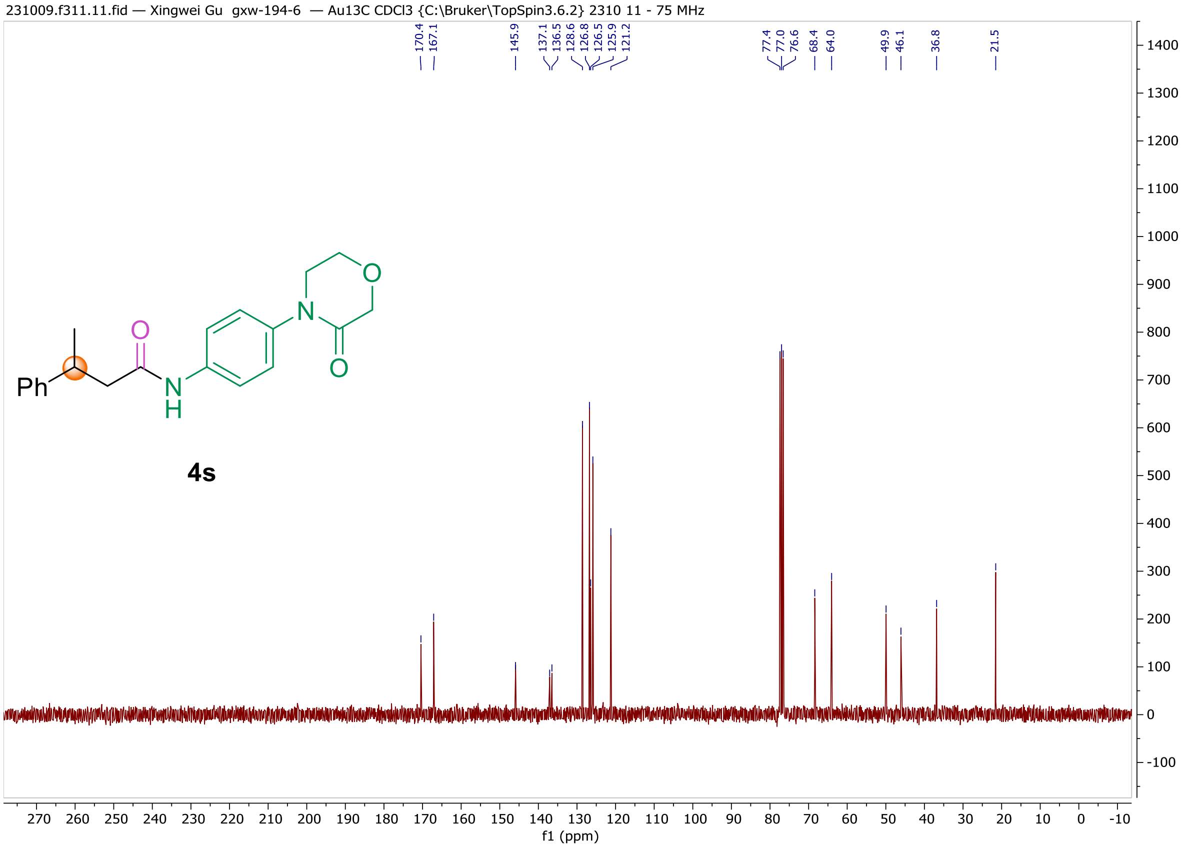
231009.f311.11.fid — Xingwei Gu gxw-194-6 — Au13C CDCl3 {C:\Bruker\TopSpin3.6.2} 2310 11 - 75 MHz
170.4 167.1 145.9 137.1 136.5 128.6 126.8 126.5 125.9 121.2 77.4 77.0 76.6 68.4 64.0 49.9 46.1 36.8 21.5
Ph
4s
f1 (ppm)

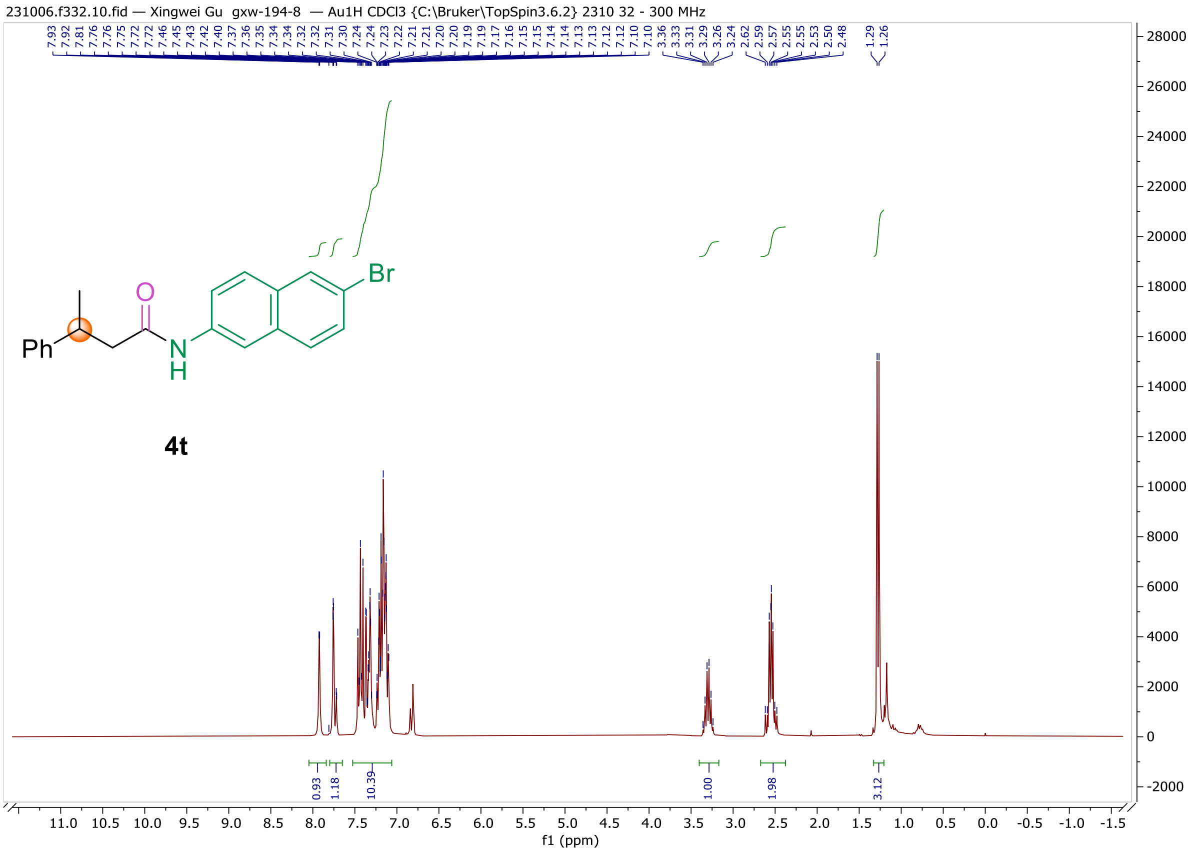
231006.f332.10.fid — Xingwei Gu gxw-194-8 — Au1H CDCl3 {C:\Bruker\TopSpin3.6.2} 2310 32 - 300 MHz
4t
f1 (ppm)

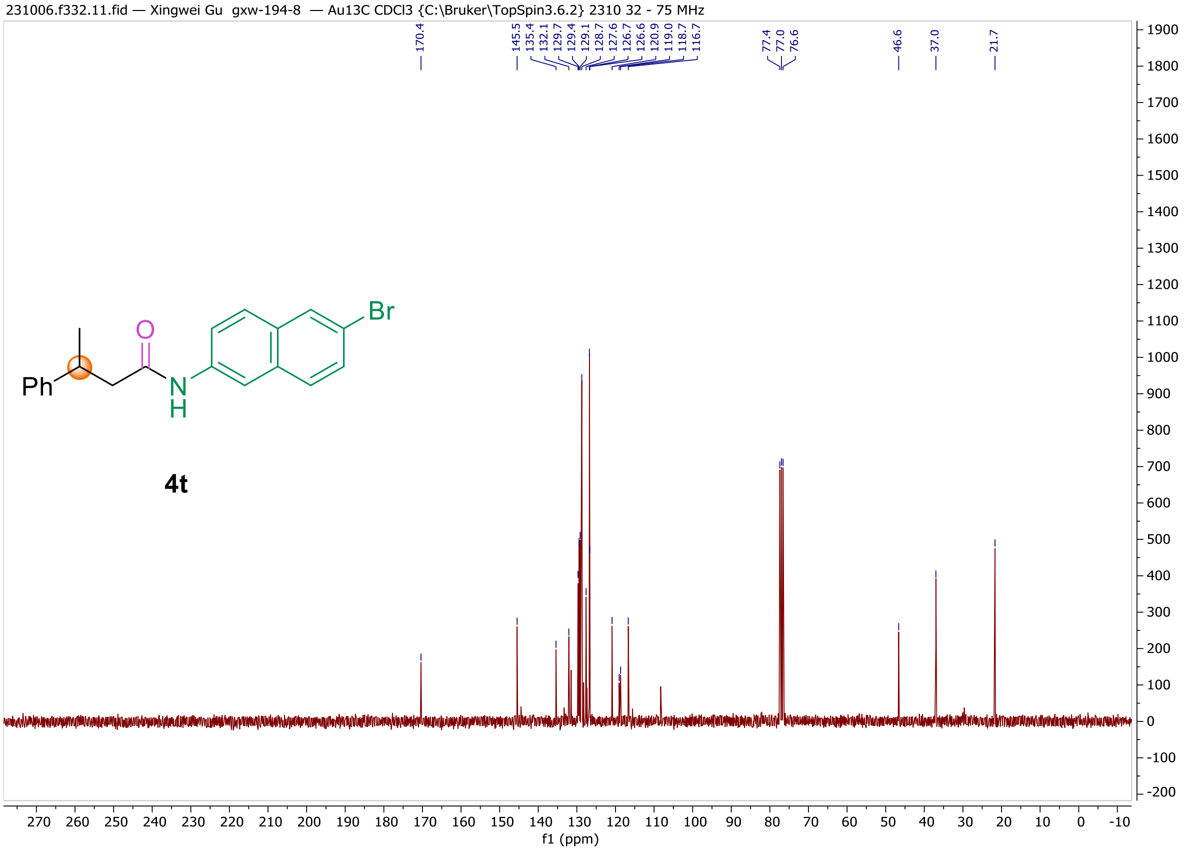
231006.f332.11.fid — Xingwei Gu gxw-194-8 — Au13C CDCl3 {C:\Bruker\TopSpin3.6.2} 2310 32 - 75 MHz
4t
f1 (ppm)

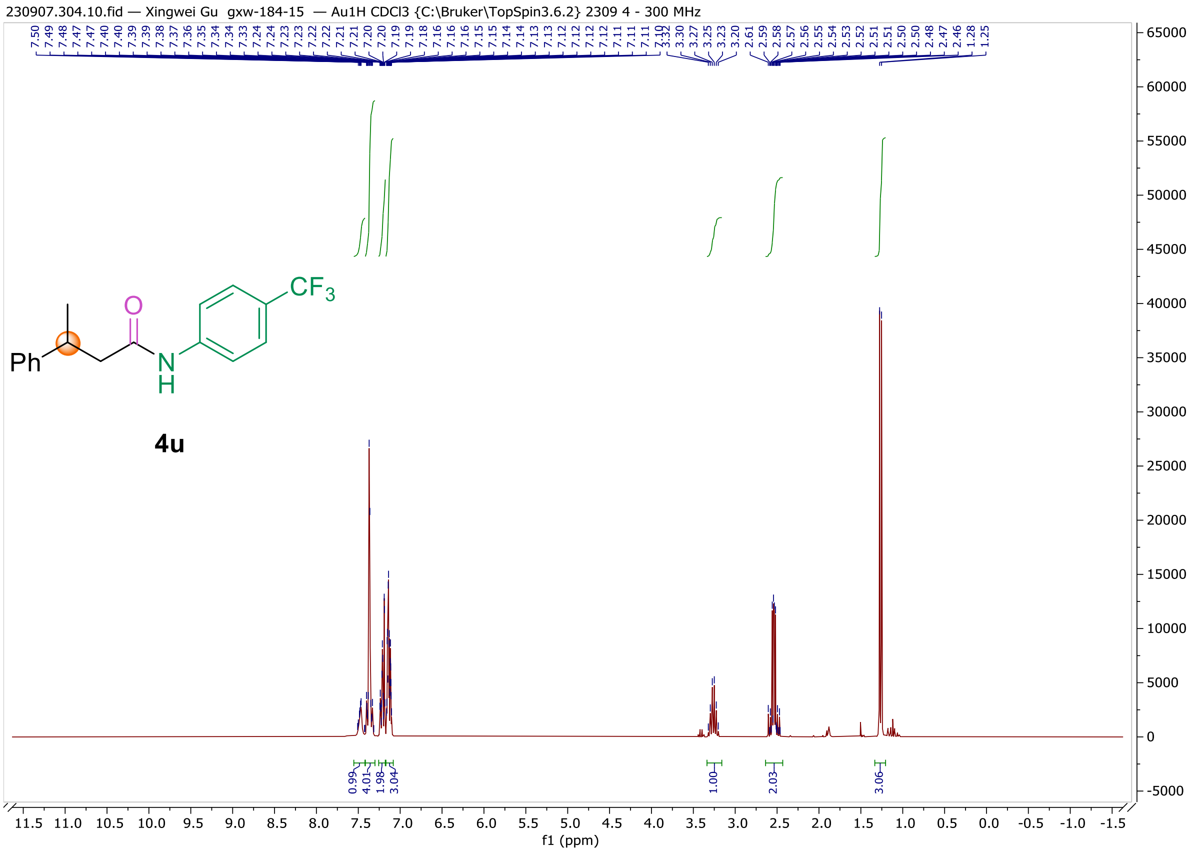

230907.304.10.fid — Xingwei Gu gxw-184-15 — Au1H CDCl3 {C:\Bruker\TopSpin3.6.2} 2309 4 - 300 MHz
4u
f1 (ppm)


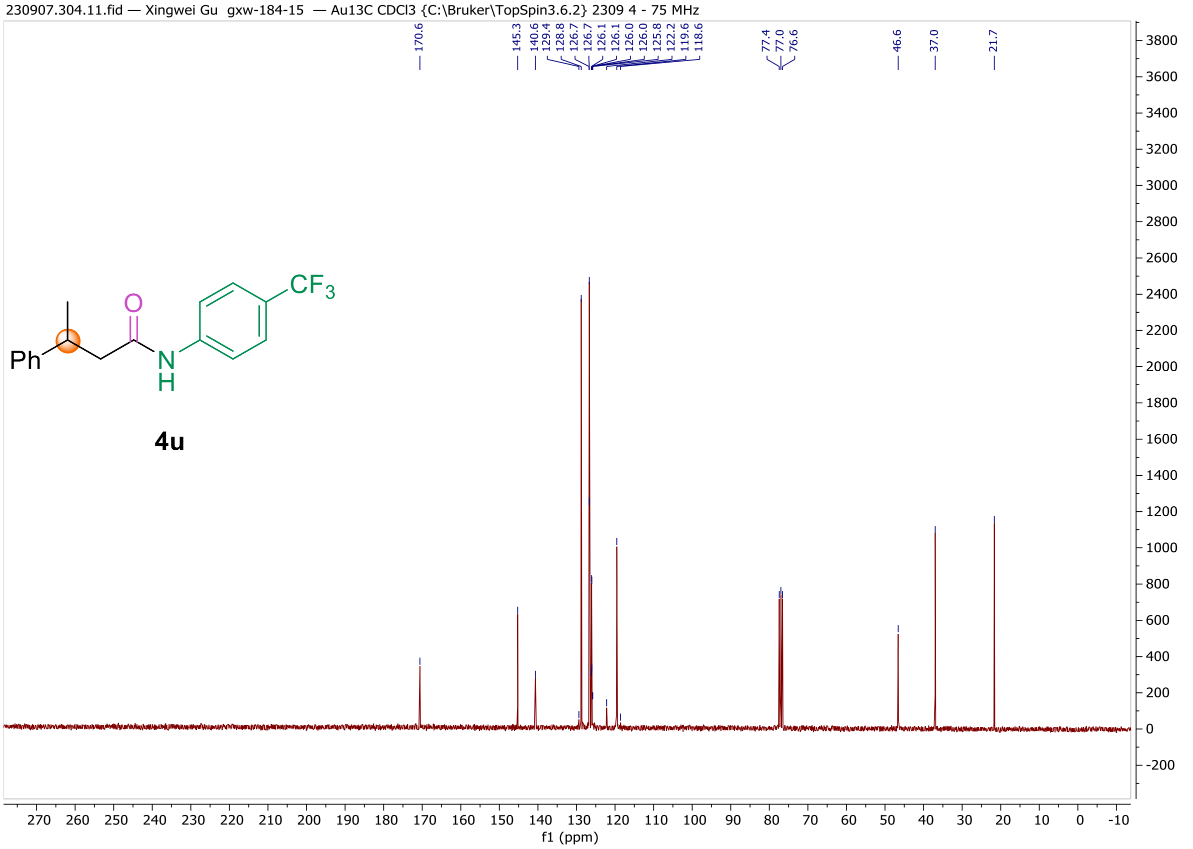

230907.304.11.fid — Xingwei Gu gxw-184-15 — Au13C CDCl3 {C:\Bruker\TopSpin3.6.2} 2309 4 - 75 MHz
170.6
145.3
140.6
129.4
128.8
126.7
126.7
126.1
126.0
125.8
122.2
119.6
118.6
77.4
77.0
76.6
46.6
37.0
21.7
4u
f1 (ppm)

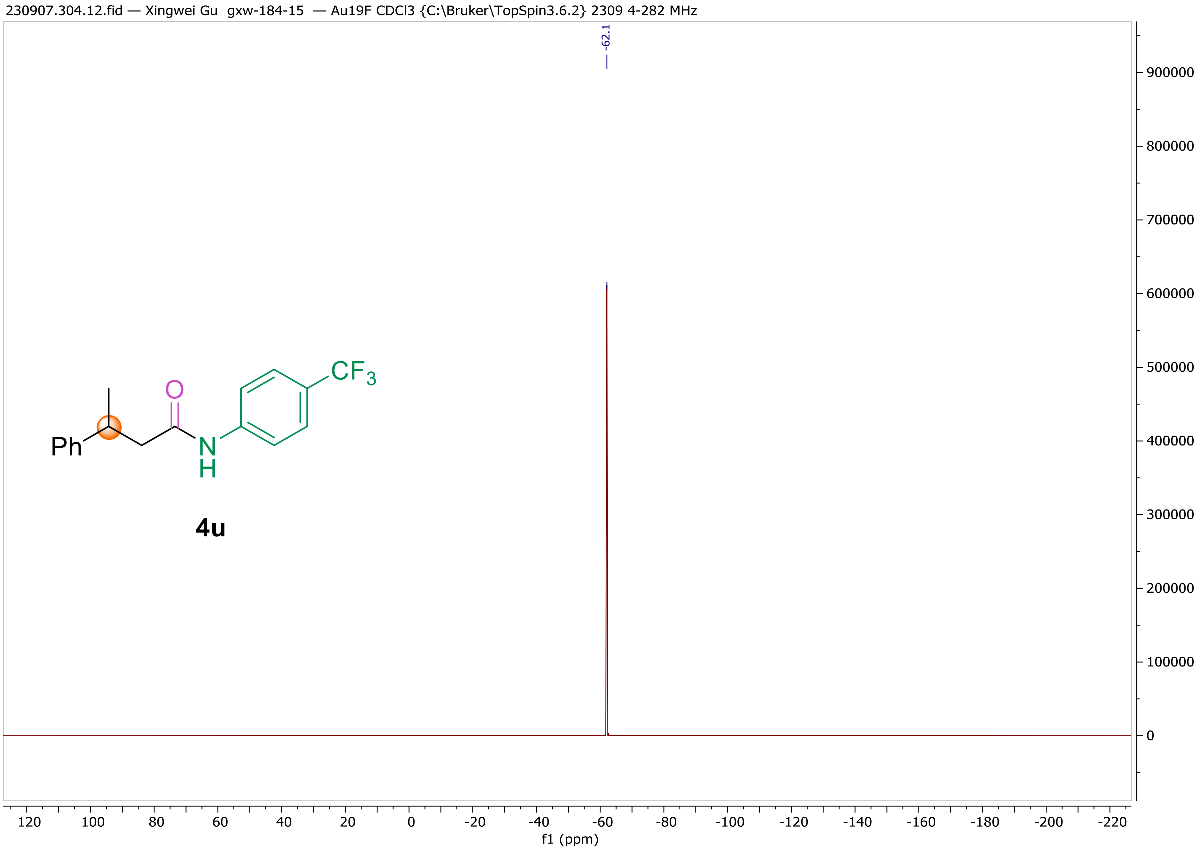
230907.304.12.fid — Xingwei Gu gxw-184-15 — Au19F CDCl3 {C:\Bruker\TopSpin3.6.2} 2309 4-282 MHz
-62.1
Ph
O
N
H
CF3
4u
900000
800000
700000
600000
500000
400000
300000
200000
100000
0
120
100
80
60
40
20
0
-20
-40
-60
-80
-100
-120
-140
-160
-180
-200
-220
f1 (ppm)

231004.302.10.fid — Xingwei Gu gxw-194-5 — Au1H CDCl3 {C:\Bruker\TopSpin3.6.2} 2310 2 - 300 MHz

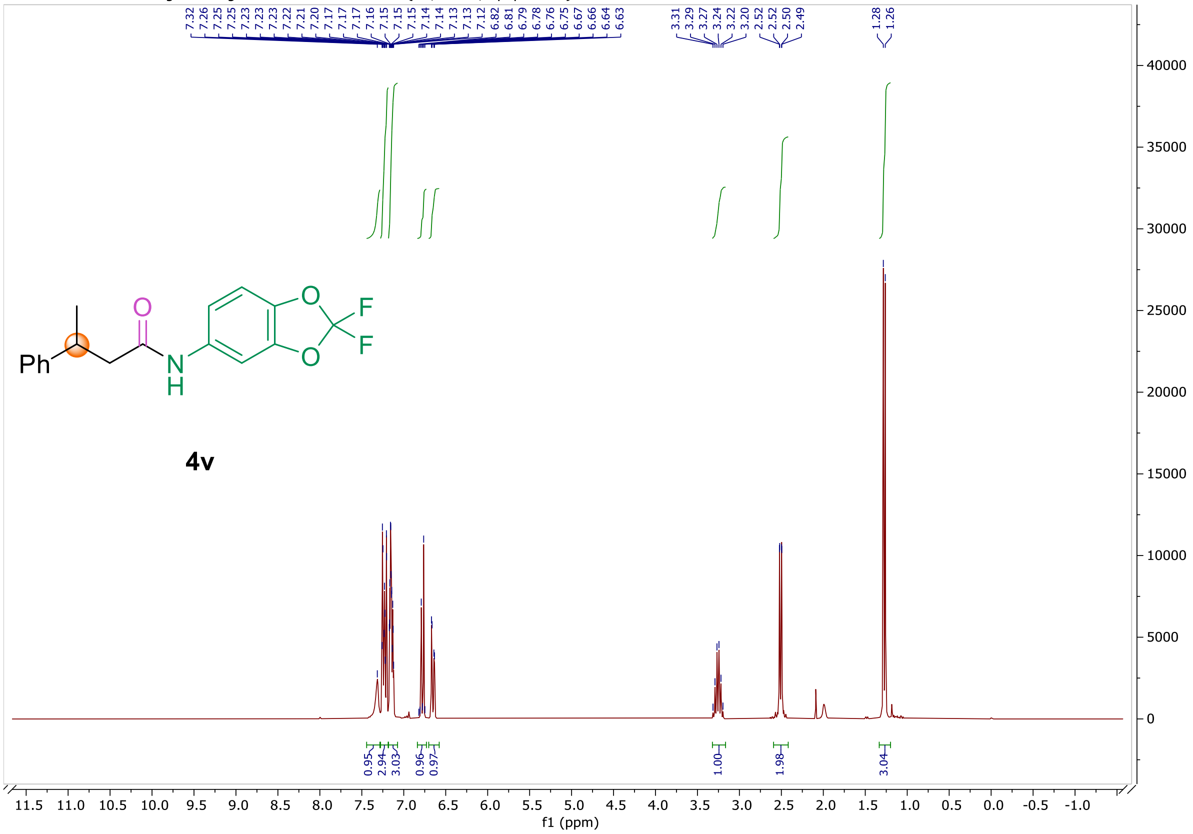


231001.301.11.fid — Xingwei Gu, gxw-194-5 — Au13C CDCl3 {C:\Bruker\TopSpin3.6.2} 2310 1 - 75 MHz

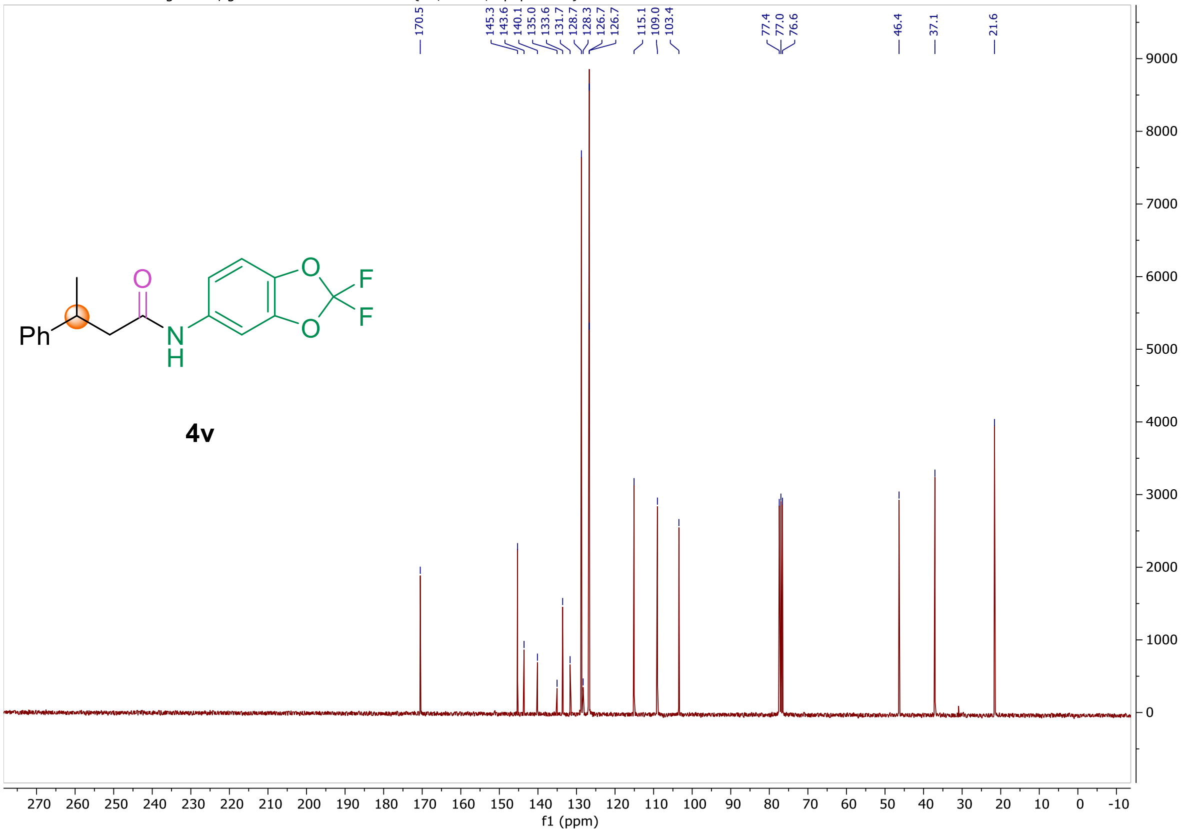

231001.301.12.fid — Xingwei Gu, gxw-194-5 — Au19F CDCl3 {C:\Bruker\TopSpin3.6.2} 2310 1-282 MHz

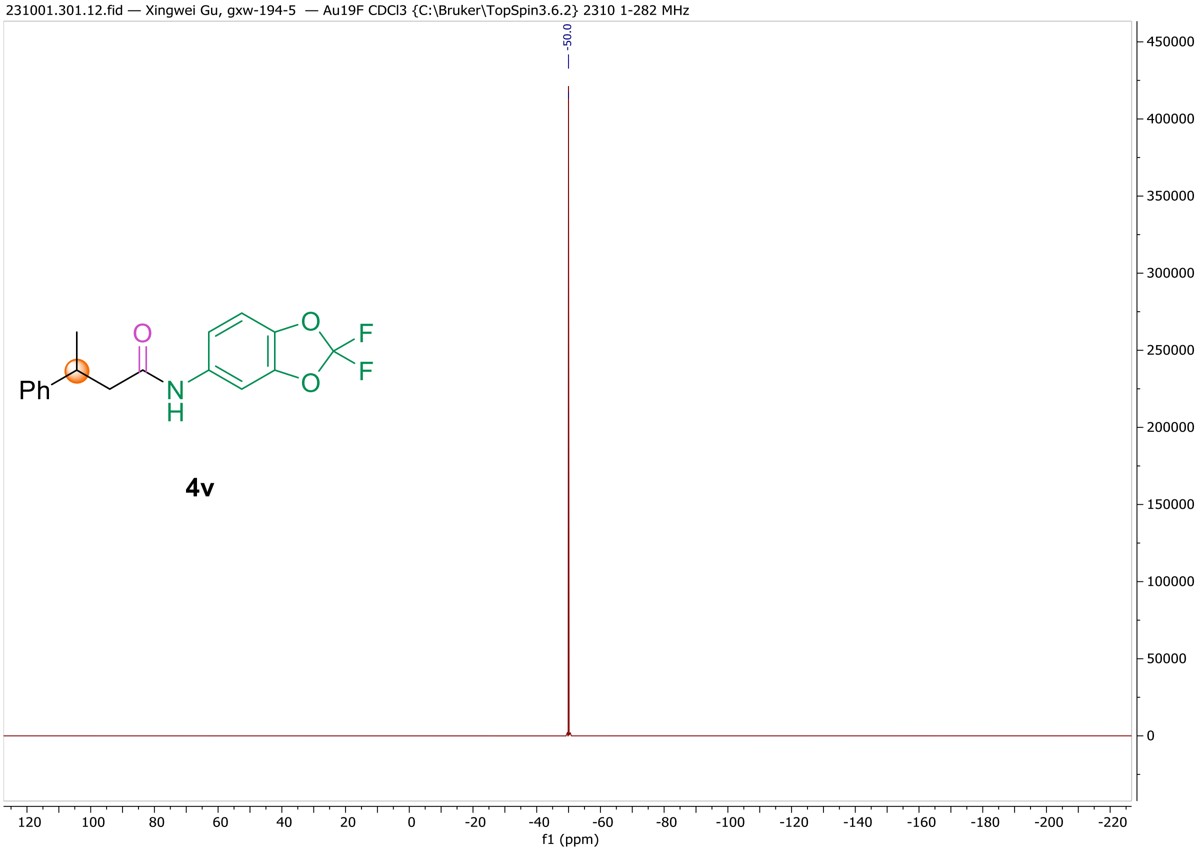


**4v**

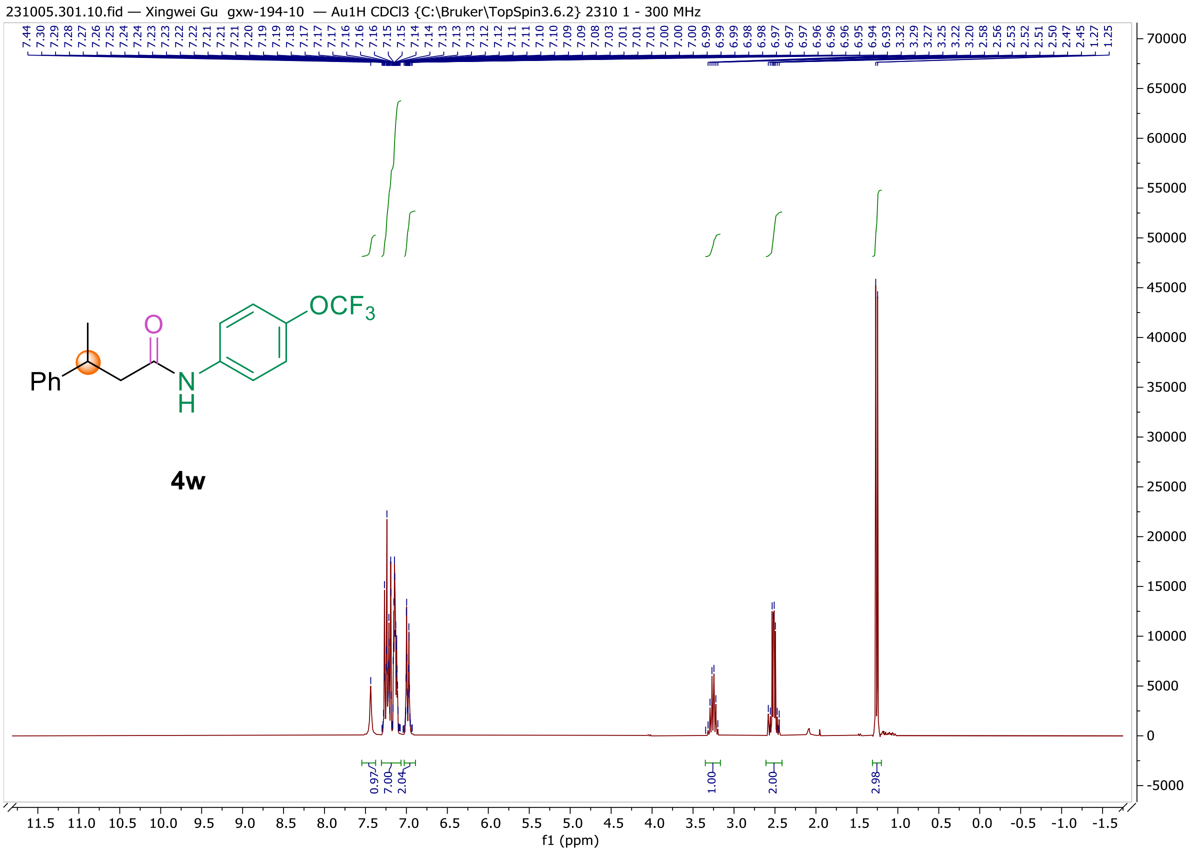
231005.301.10.fid — Xingwei Gu gxw-194-10 — Au1H CDCl3 {C:\Bruker\TopSpin3.6.2} 2310 1 - 300 MHz
OCF3
O
Ph
N
H
4w
f1 (ppm)

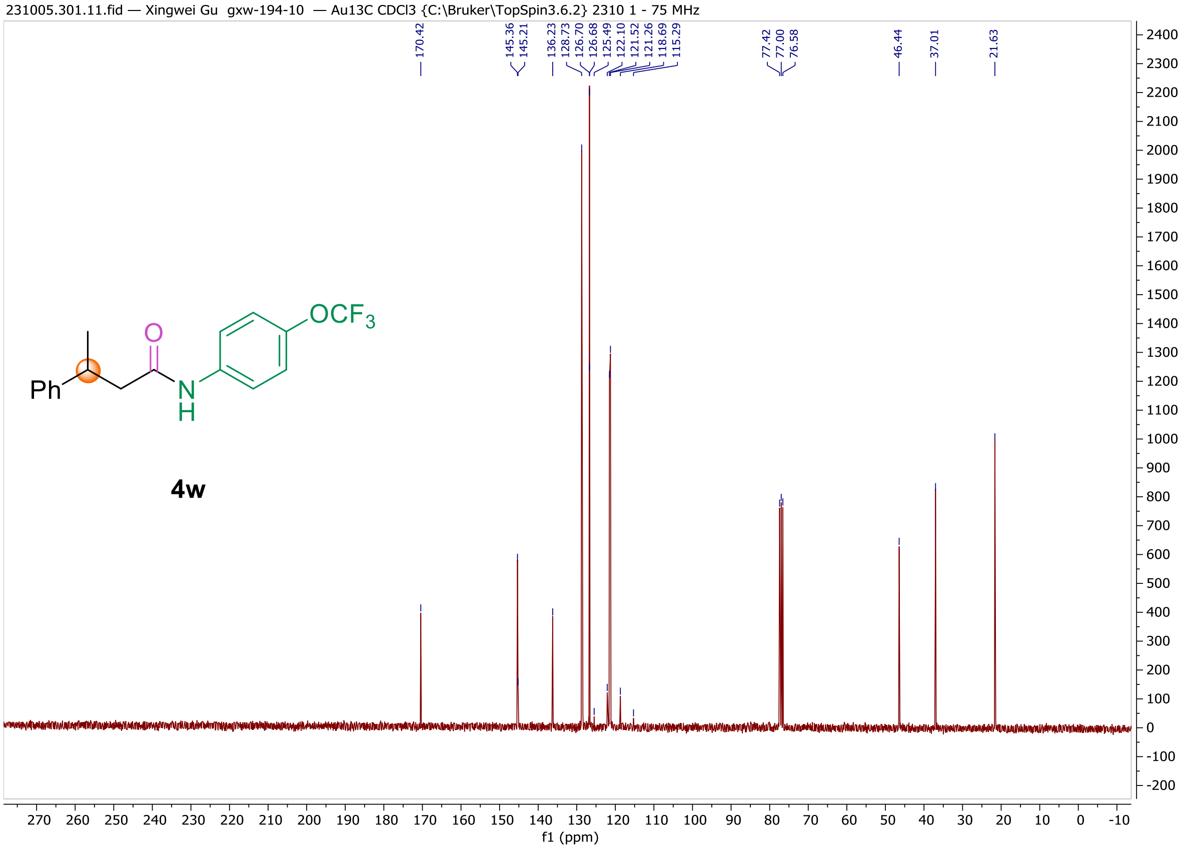
231005.301.11.fid — Xingwei Gu gxw-194-10 — Au13C CDCl3 {C:\Bruker\TopSpin3.6.2} 2310 1 - 75 MHz
170.42
145.36
145.21
136.23
128.73
126.70
126.68
125.49
122.10
121.52
121.26
118.69
115.29
77.42
77.00
76.58
46.44
37.01
21.63
OCF3
O
Ph
N
H
4w
f1 (ppm)

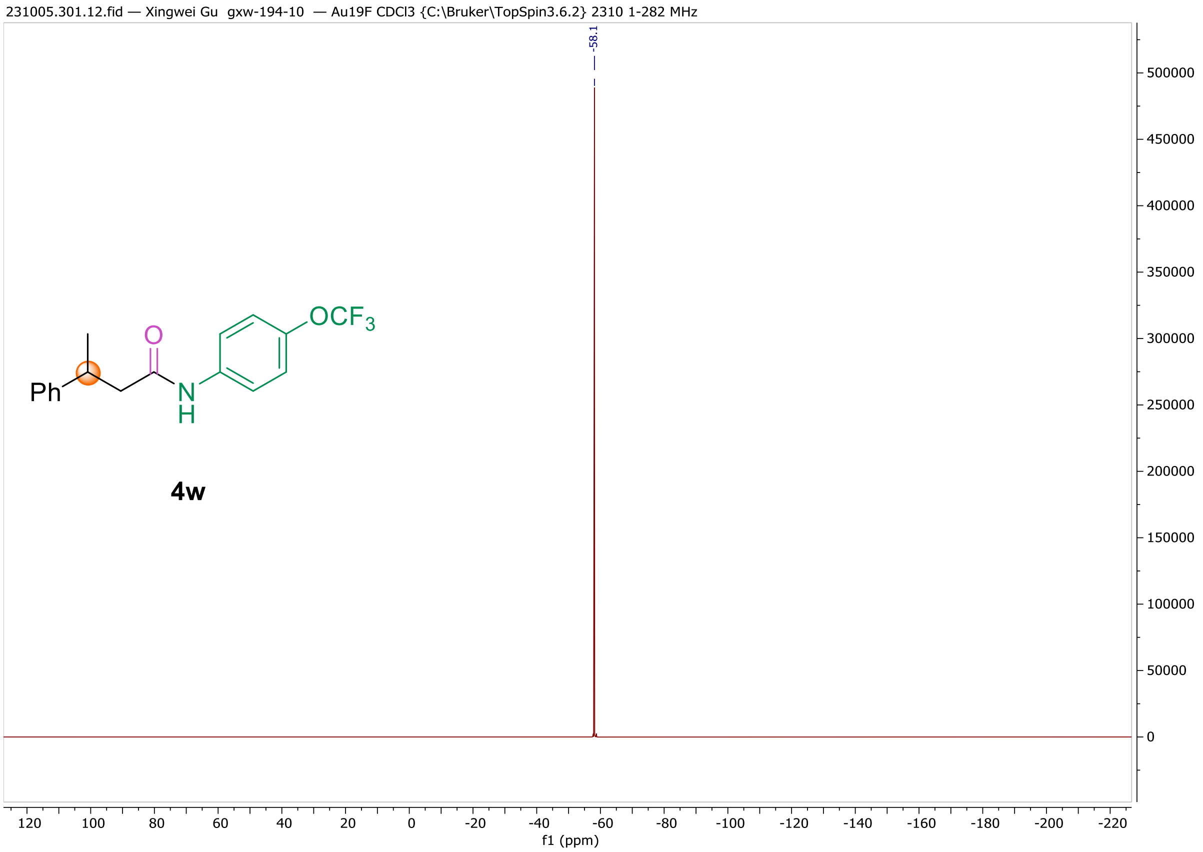
231005.301.12.fid — Xingwei Gu gxw-194-10 — Au19F CDCl3 {C:\Bruker\TopSpin3.6.2} 2310 1-282 MHz
-58.1
OCF3
O
Ph
N
H
4w
f1 (ppm)

231005.303.10.fid — Xingwei Gu gxw-194-12 — Au1H CDCl3 {C:\Bruker\TopSpin3.6.2} 2310 3 - 300 MHz

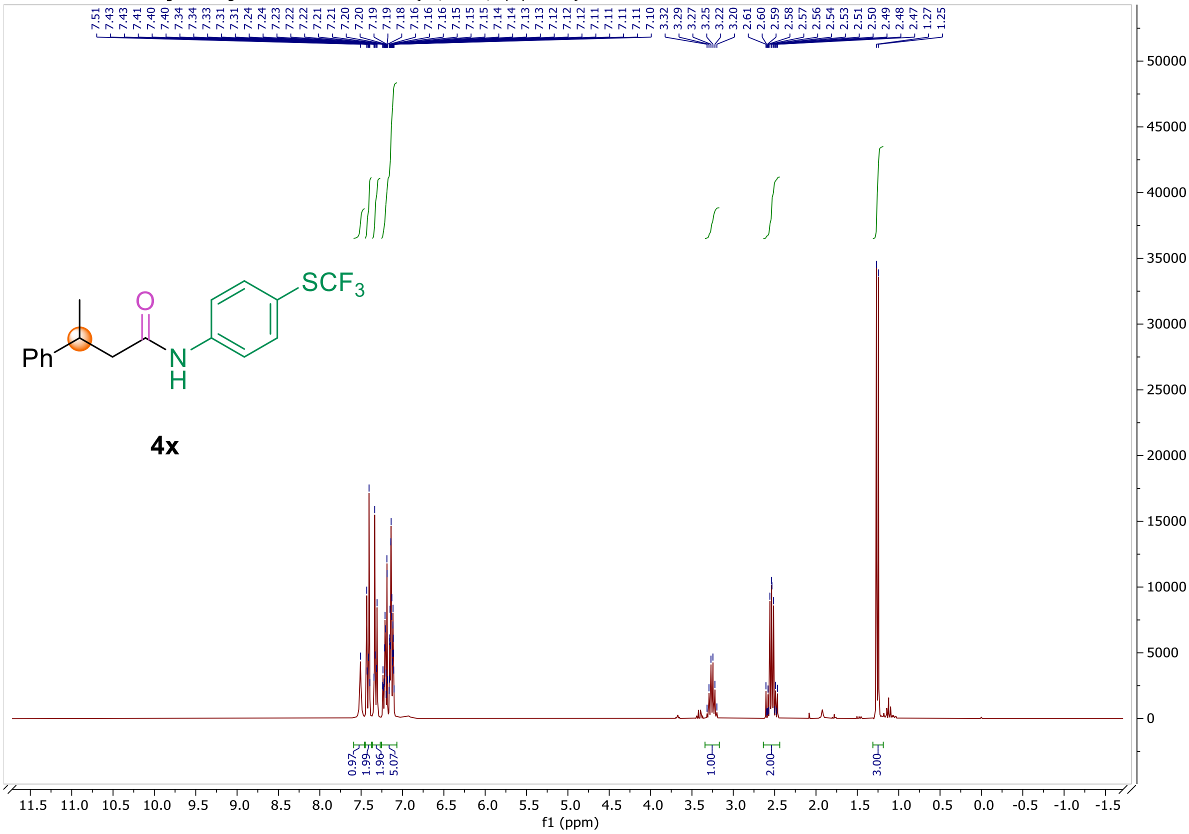


**4x**

231005.303.11.fid — Xingwei Gu gxw-194-12 — Au13C CDCl3 {C:\Bruker\TopSpin3.6.2} 2310 3 - 75 MHz

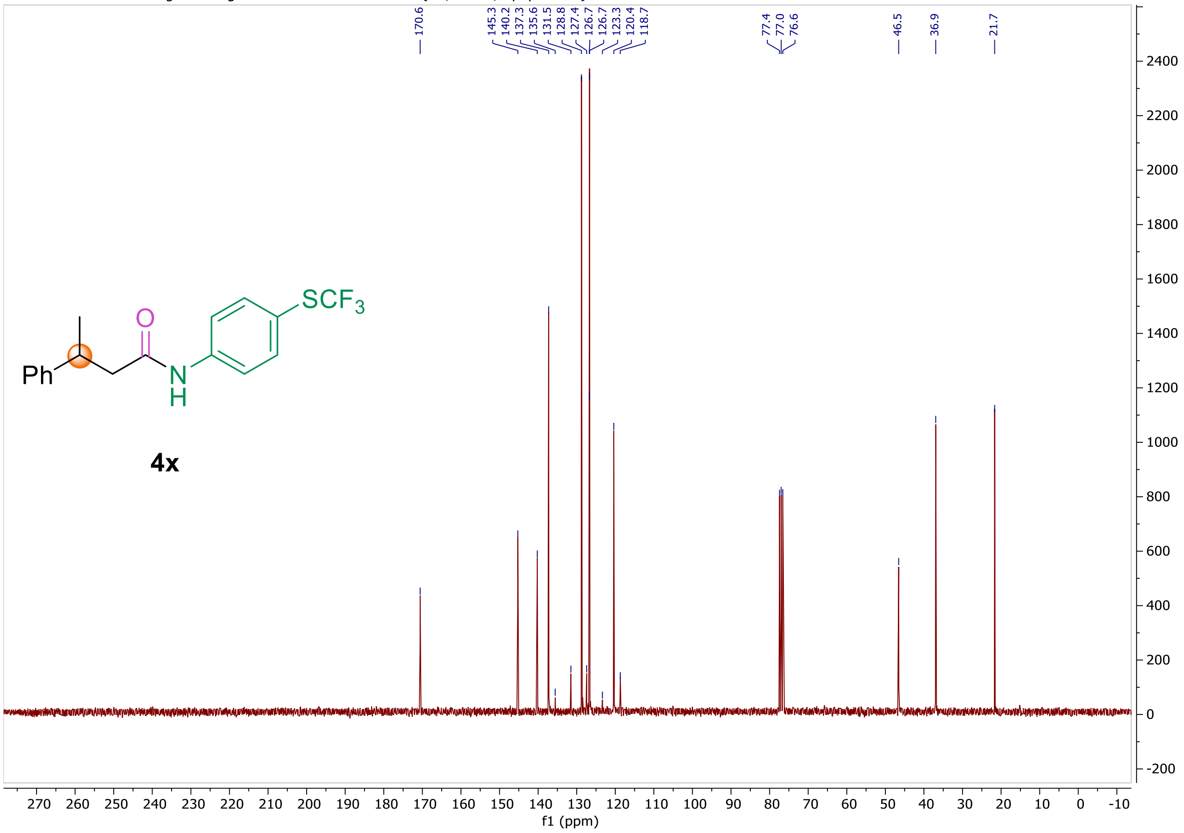


**4x**

231005.303.12.fid — Xingwei Gu gxw-194-12 — Au19F CDCl3 {C:\Bruker\TopSpin3.6.2} 2310 3-282 MHz

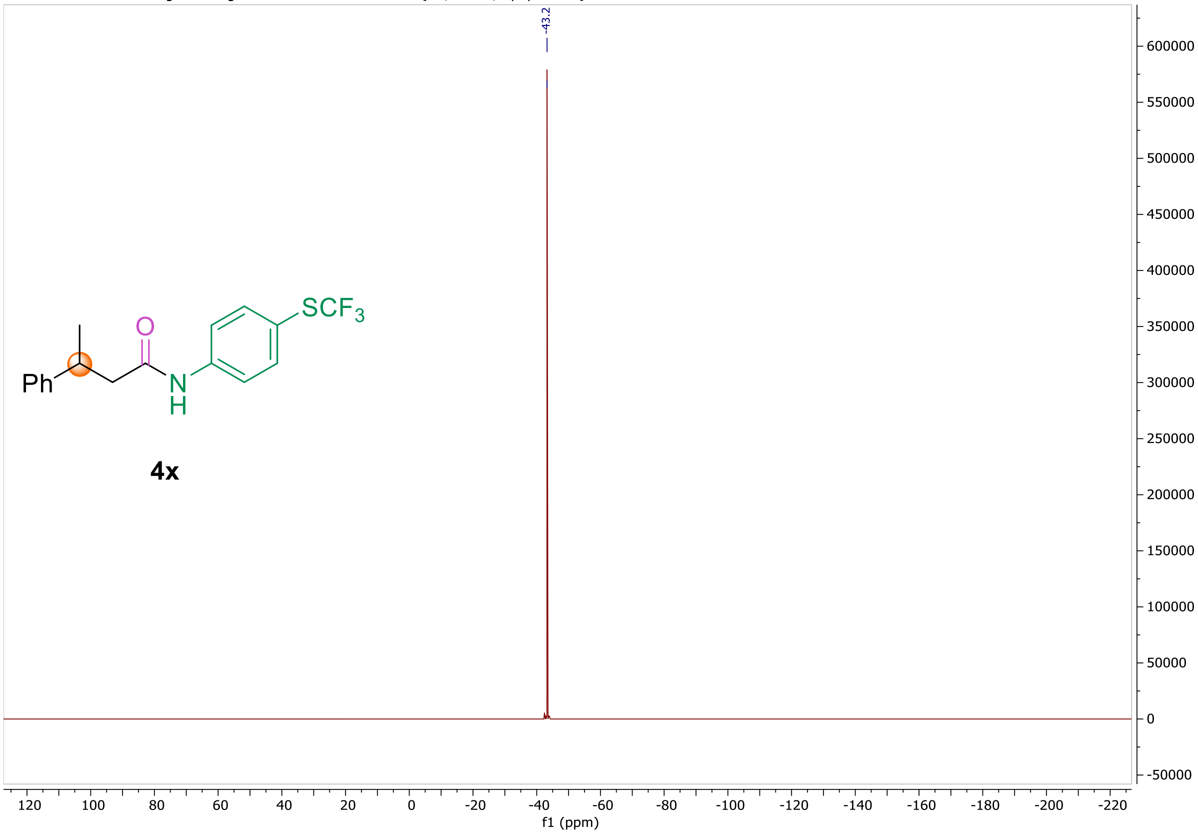

231005.302.10.fid — Xingwei Gu gxw-194-11 — Au1H CDCl3 {C:\Bruker\TopSpin3.6.2} 2310 2 - 300 MHz

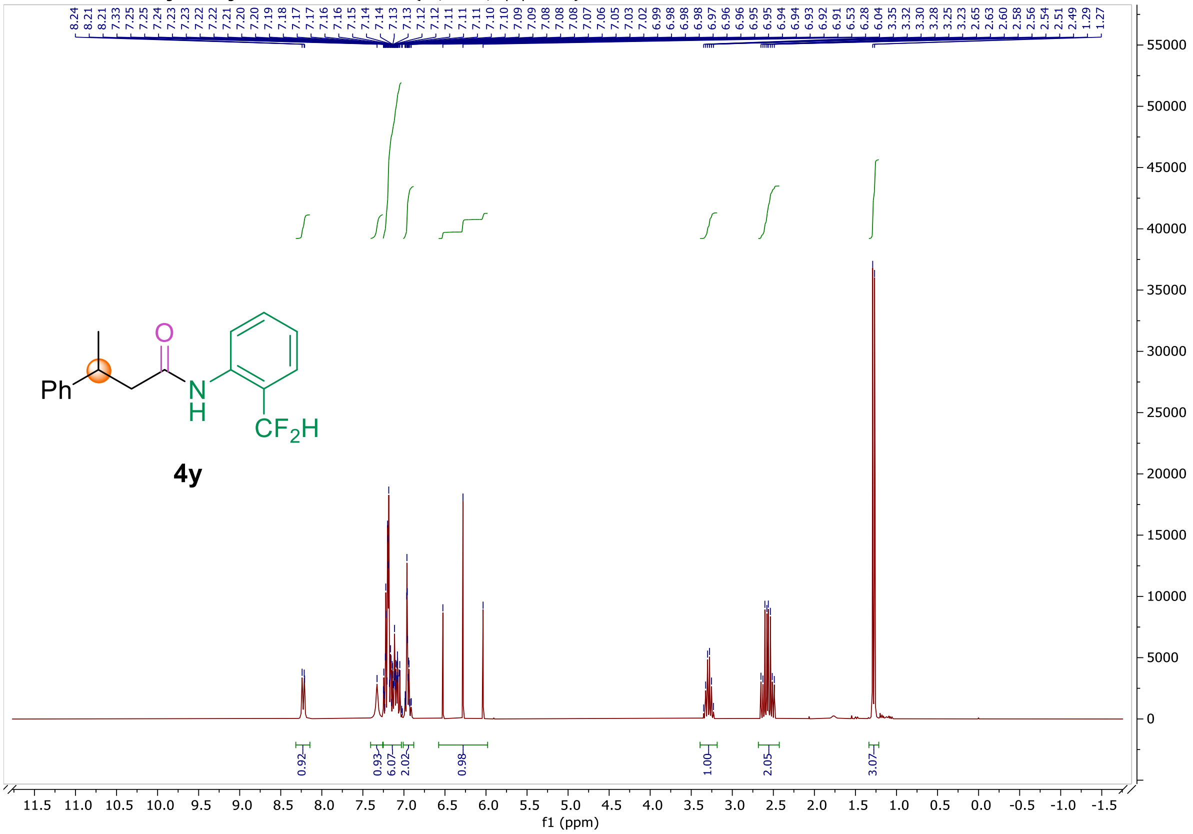


231005.302.11.fid — Xingwei Gu gxw-194-11 — Au13C CDCl3 {C:\Bruker\TopSpin3.6.2} 2310 2 - 75 MHz

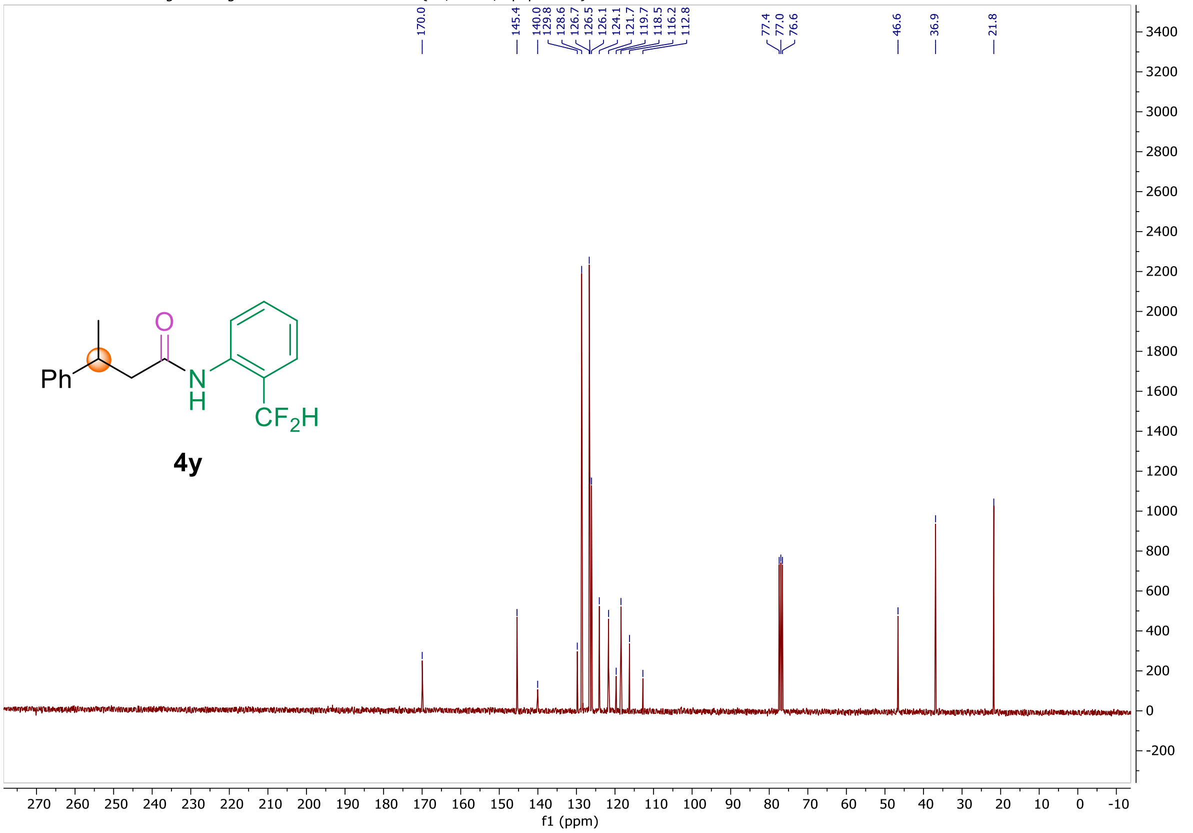

231005.302.12.fid — Xingwei Gu gxw-194-11 — Au19F CDCl3 {C:\Bruker\TopSpin3.6.2} 2310 2-282 MHz

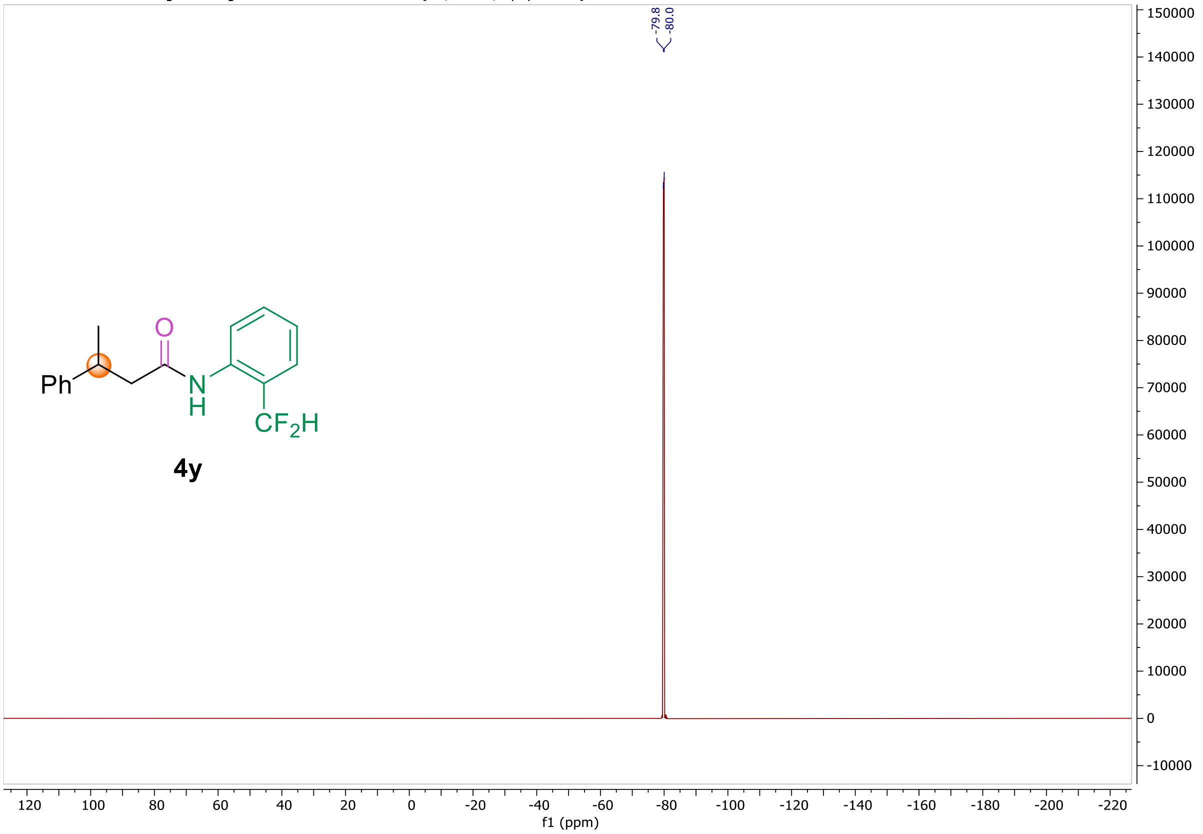


**4y**

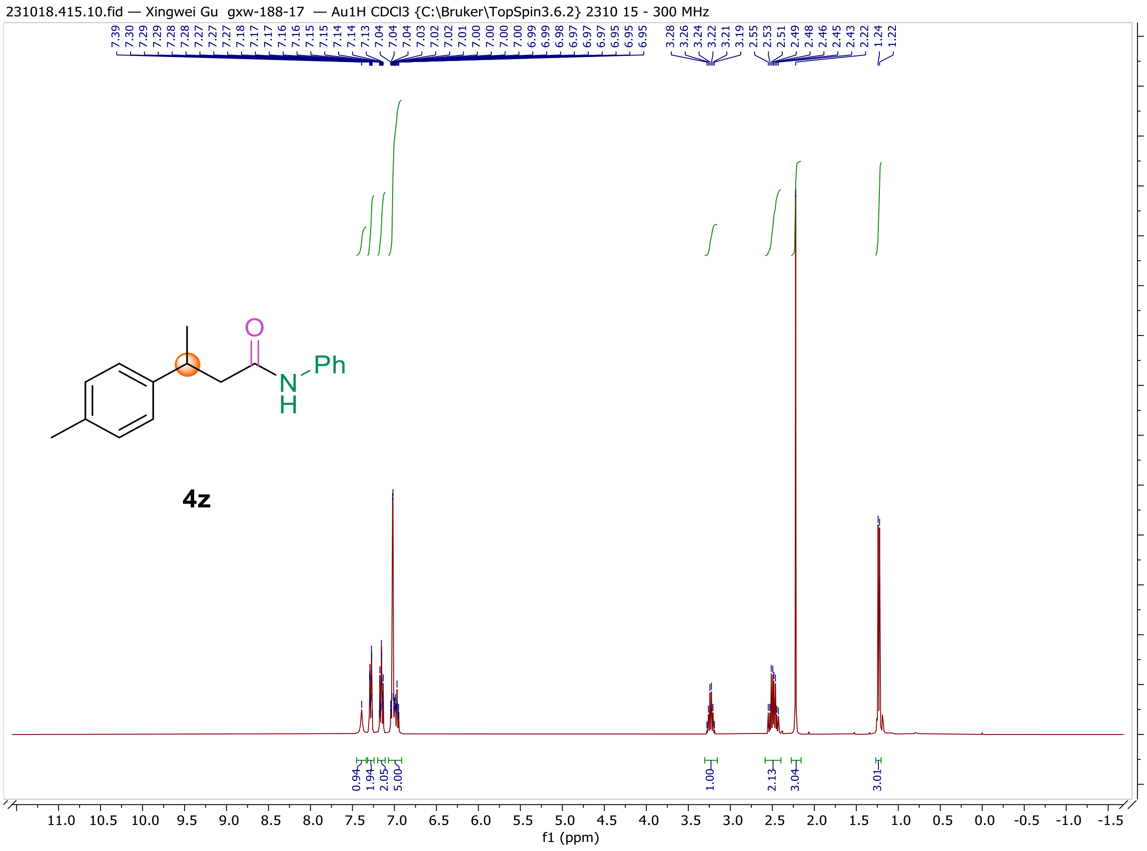
231018.415.10.fid — Xingwei Gu gxw-188-17 — Au1H CDCl3 {C:\Bruker\TopSpin3.6.2} 2310 15 - 300 MHz
7.39 7.30 7.29 7.29 7.28 7.28 7.27 7.27 7.27 7.18 7.17 7.17 7.16 7.16 7.15 7.15 7.14 7.14 7.13 7.04 7.04 7.04 7.03 7.02 7.02 7.01 7.00 7.00 7.00 7.00 6.99 6.99 6.98 6.97 6.97 6.97 6.95 6.95 6.95
3.28 3.26 3.24 3.22 3.21 3.19 2.55 2.53 2.51 2.49 2.48 2.46 2.45 2.43 2.22 1.24 1.22
0.94 1.94 2.05 5.00 1.00 2.13 3.04 3.01
O
N
H
Ph
4z
f1 (ppm)

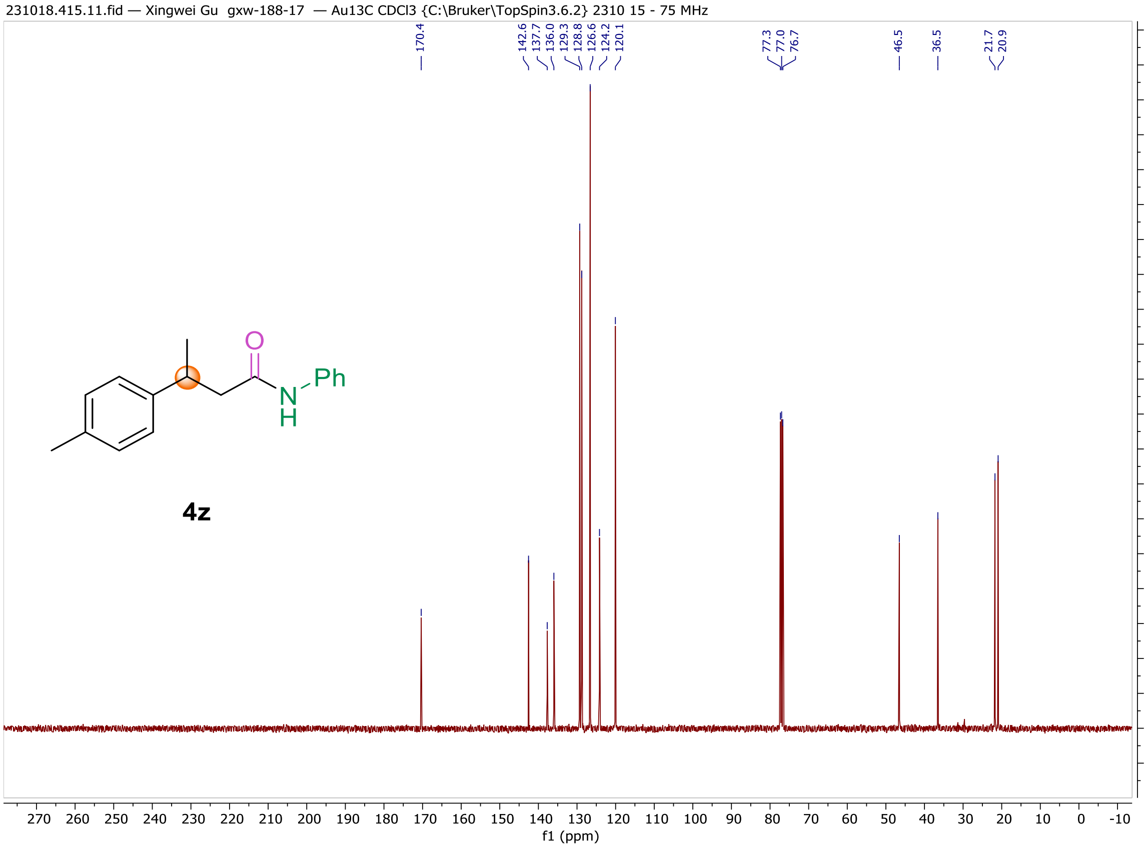
231018.415.11.fid — Xingwei Gu gxw-188-17 — Au13C CDCl3 {C:\Bruker\TopSpin3.6.2} 2310 15 - 75 MHz
170.4
142.6 137.7 136.0 129.3 128.8 126.6 124.2 120.1
77.3 77.0 76.7
46.5
36.5
21.7 20.9
O
N
H
Ph
4z
f1 (ppm)

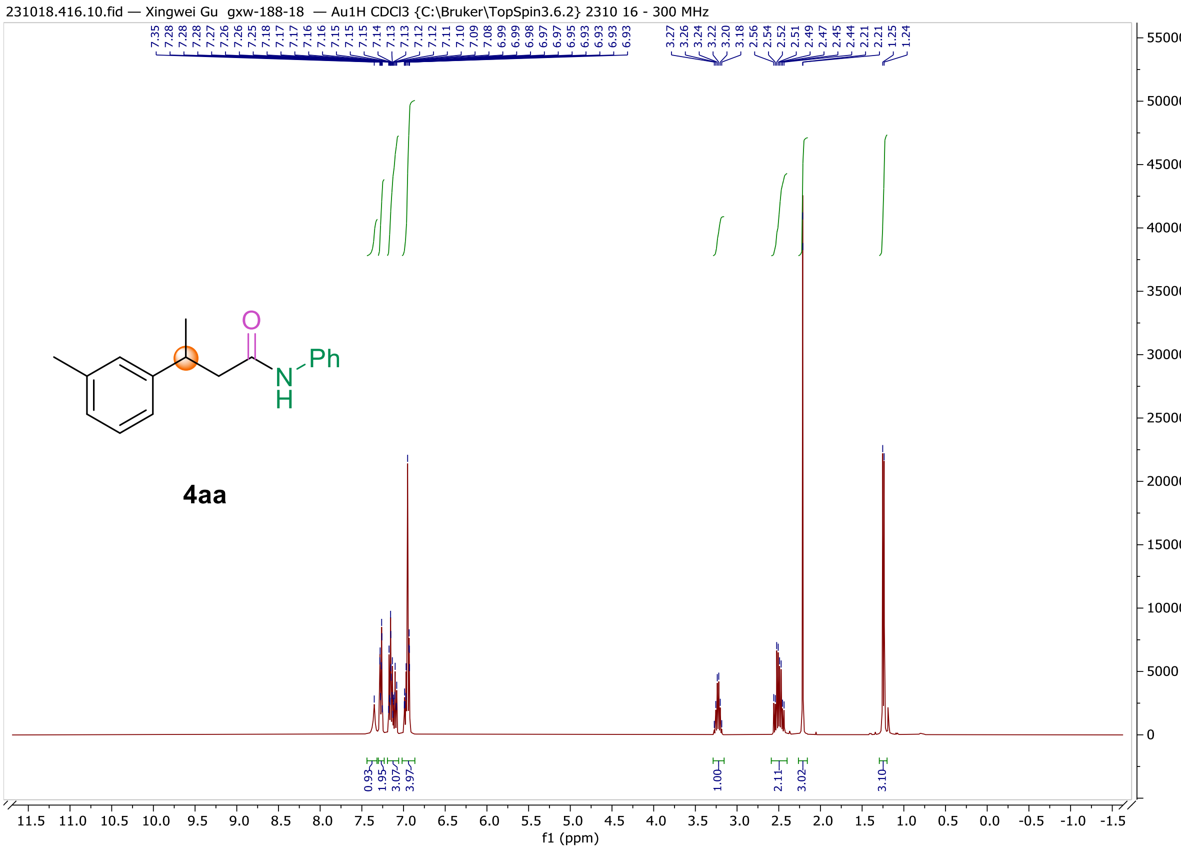
231018.416.10.fid — Xingwei Gu gxw-188-18 — Au1H CDCl3 {C:\Bruker\TopSpin3.6.2} 2310 16 - 300 MHz
7.35 7.28 7.28 7.28 7.27 7.26 7.26 7.25 7.18 7.17 7.17 7.16 7.16 7.15 7.15 7.14 7.13 7.13 7.12 7.12 7.11 7.10 7.09 7.08 6.99 6.99 6.98 6.97 6.97 6.95 6.93 6.93 6.93 6.93
3.27 3.26 3.24 3.22 3.20 3.18 2.56 2.54 2.52 2.51 2.49 2.47 2.45 2.44 2.21 2.21 1.25 1.24
0.93 1.95 3.07 3.97 1.00 2.11 3.02 3.10
O
N
H
Ph
4aa
f1 (ppm)

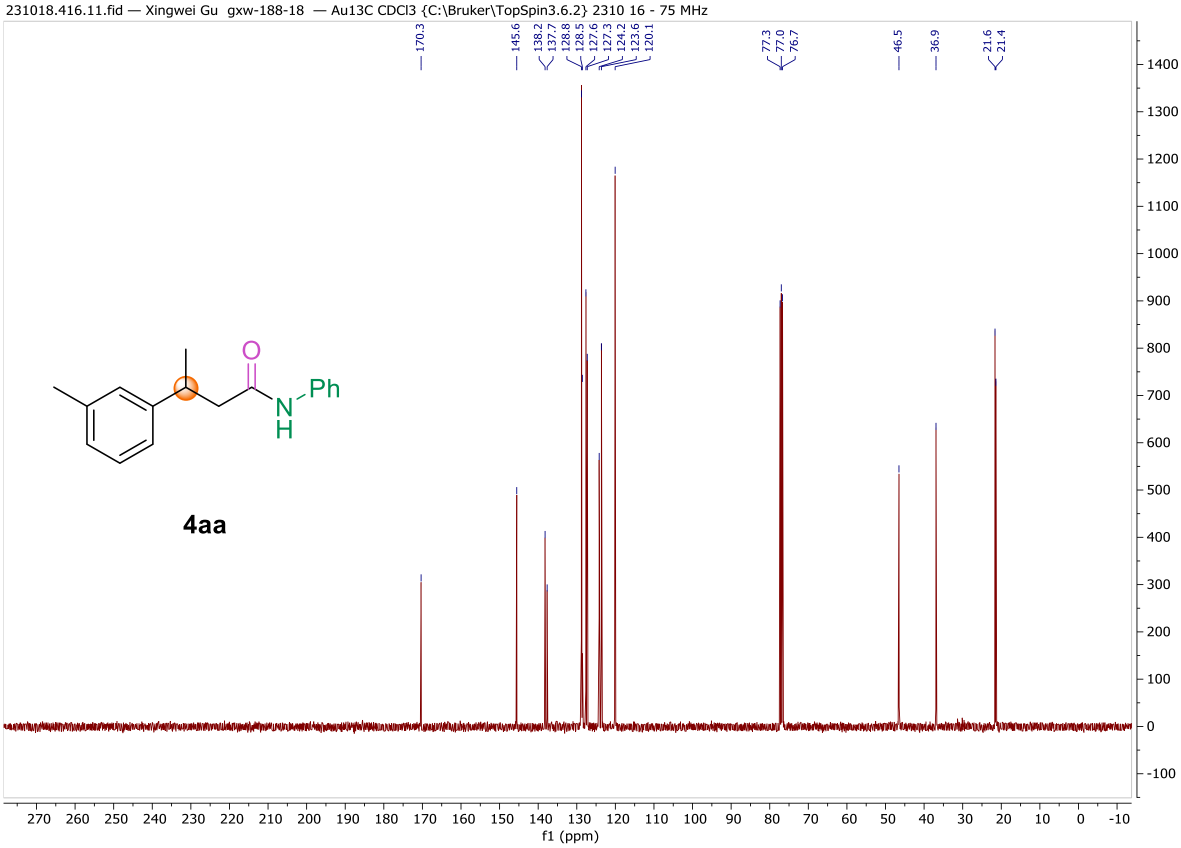
231018.416.11.fid — Xingwei Gu gxw-188-18 — Au13C CDCl3 {C:\Bruker\TopSpin3.6.2} 2310 16 - 75 MHz
170.3
145.6 138.2 137.7 128.8 128.5 127.6 127.3 124.2 123.6 120.1
77.3 77.0 76.7
46.5
36.9
21.6 21.4
O
N
H
Ph
4aa
f1 (ppm)

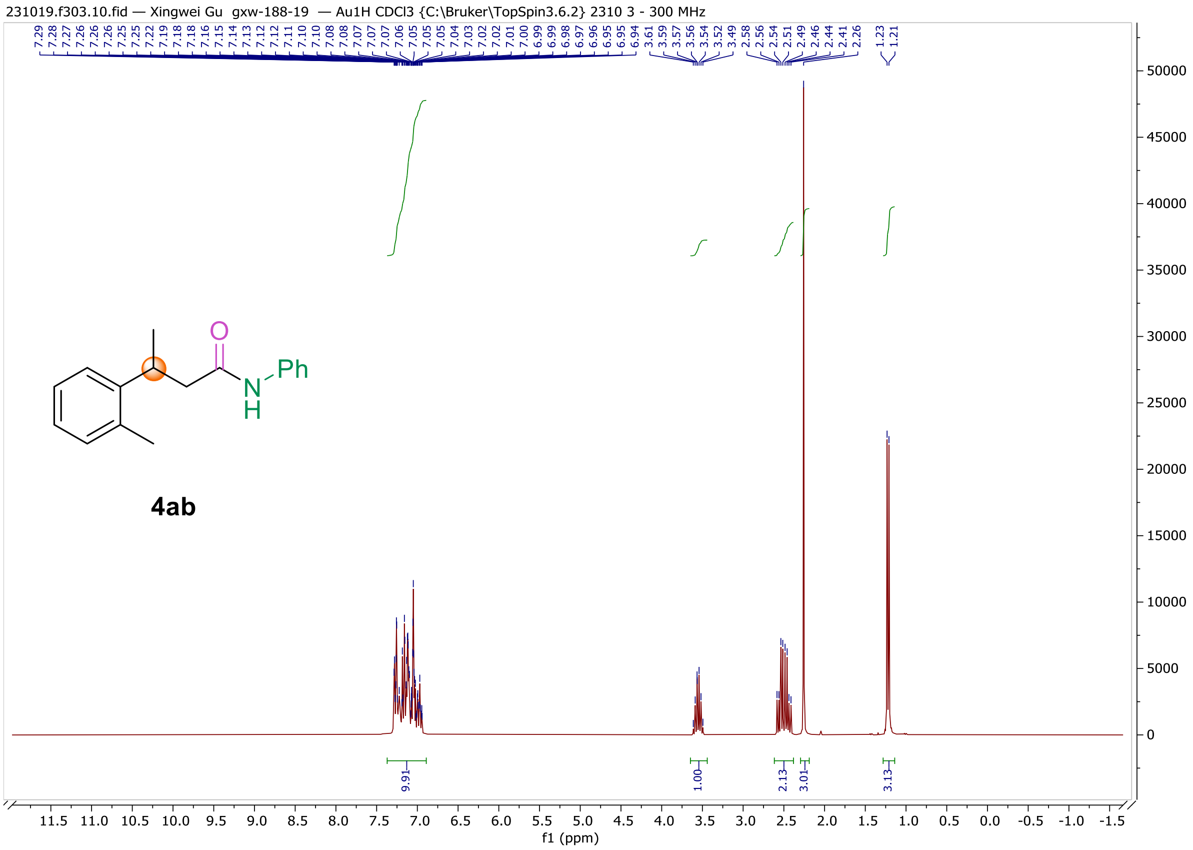
231019.f303.10.fid — Xingwei Gu gxw-188-19 — Au1H CDCl3 {C:\Bruker\TopSpin3.6.2} 2310 3 - 300 MHz
7.29 7.28 7.27 7.26 7.26 7.26 7.25 7.25 7.22 7.19 7.18 7.18 7.16 7.15 7.14 7.13 7.12 7.12 7.11 7.10 7.10 7.08 7.08 7.07 7.07 7.06 7.05 7.05 7.05 7.04 7.03 7.02 7.02 7.01 7.00 6.99 6.99 6.98 6.97 6.96 6.96 6.95 6.95 6.94
3.61 3.59 3.57 3.56 3.54 3.52 3.49
2.58 2.56 2.54 2.51 2.49 2.46 2.44 2.41 2.26
1.23 1.21
O
N
H
Ph
4ab
9.91
1.00
2.13
3.01
3.13
f1 (ppm)

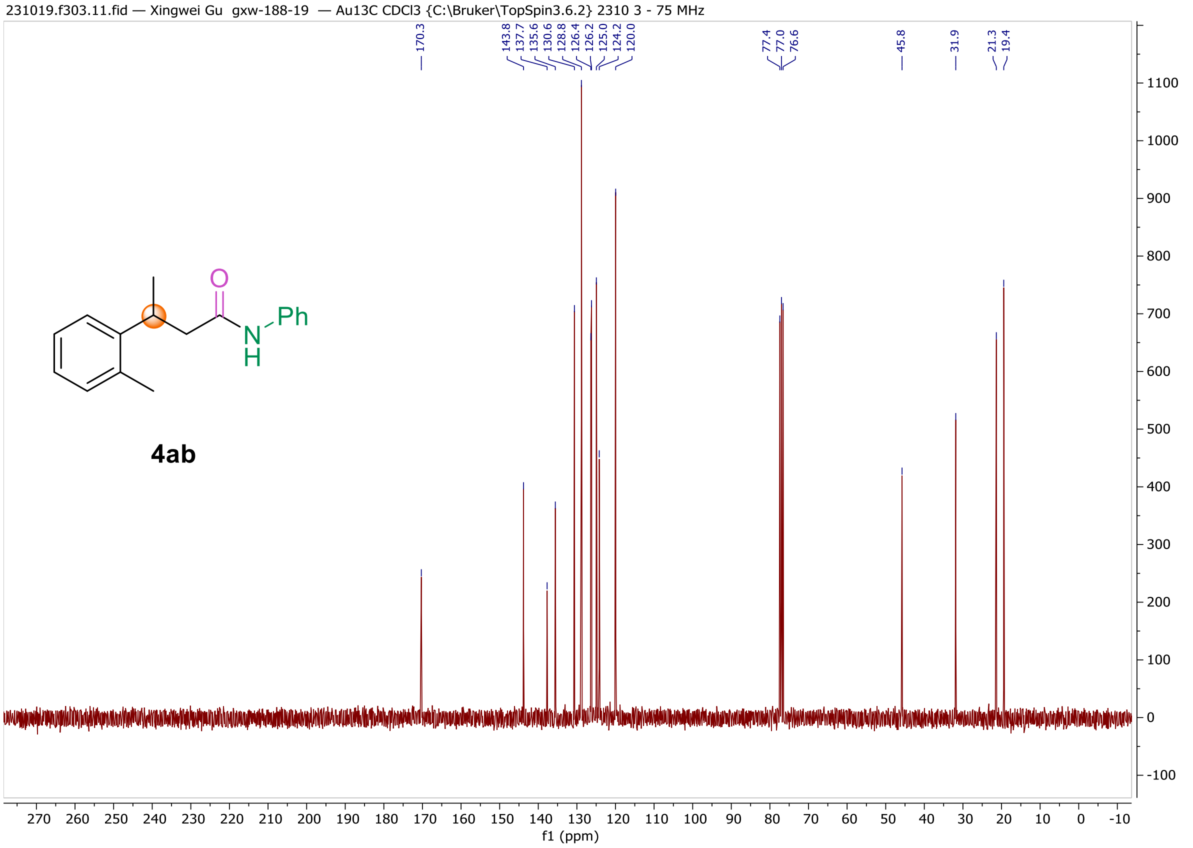
231019.f303.11.fid — Xingwei Gu gxw-188-19 — Au13C CDCl3 {C:\Bruker\TopSpin3.6.2} 2310 3 - 75 MHz
170.3
143.8 137.7 135.6 130.6 128.8 126.4 126.2 125.0 124.2 120.0
77.4 77.0 76.6
45.8
31.9
21.3 19.4
O
N
H
Ph
4ab
f1 (ppm)

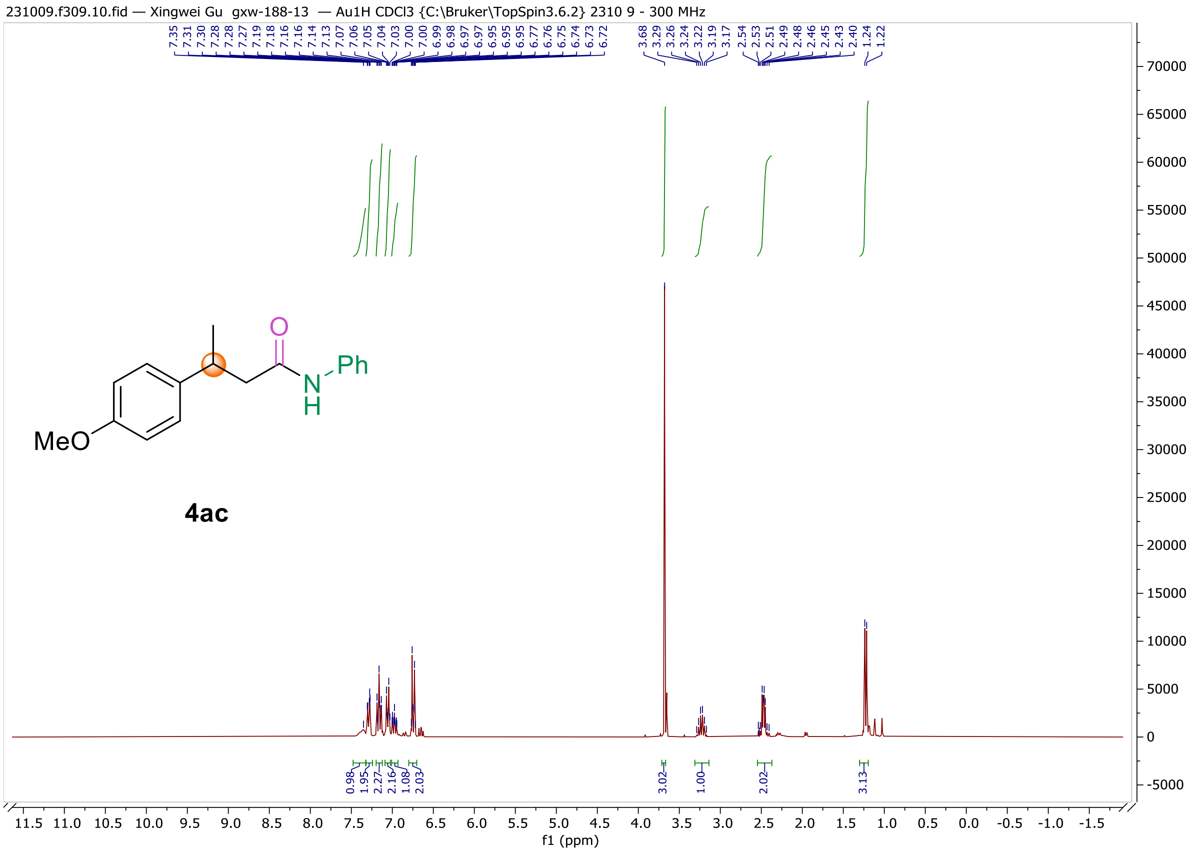
231009.f309.10.fid — Xingwei Gu gxw-188-13 — Au1H CDCl3 {C:\Bruker\TopSpin3.6.2} 2310 9 - 300 MHz
MeO
O
N
H
Ph
4ac
f1 (ppm)

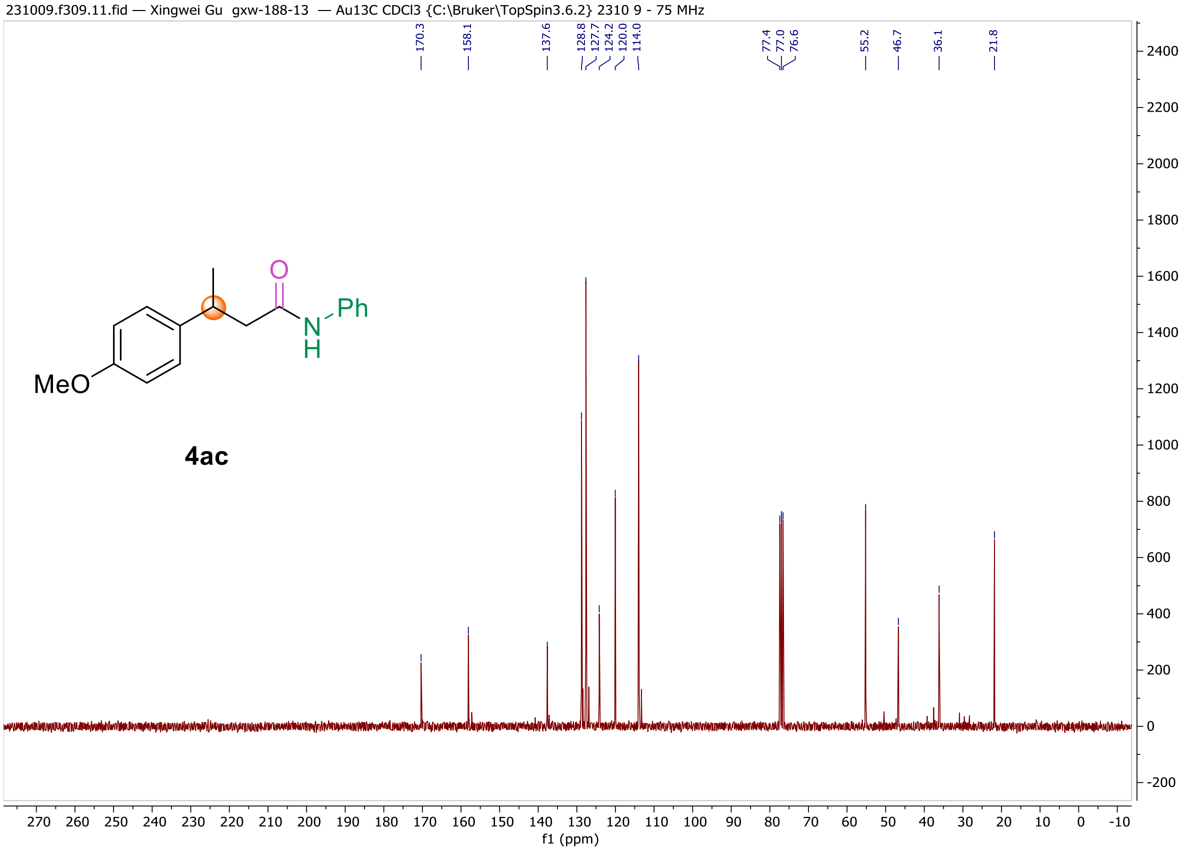
231009.f309.11.fid — Xingwei Gu gxw-188-13 — Au13C CDCl3 {C:\Bruker\TopSpin3.6.2} 2310 9 - 75 MHz
170.3
158.1
137.6
128.8
127.7
124.2
120.0
114.0
77.4
77.0
76.6
55.2
46.7
36.1
21.8
MeO
O
N
H
Ph
4ac
f1 (ppm)

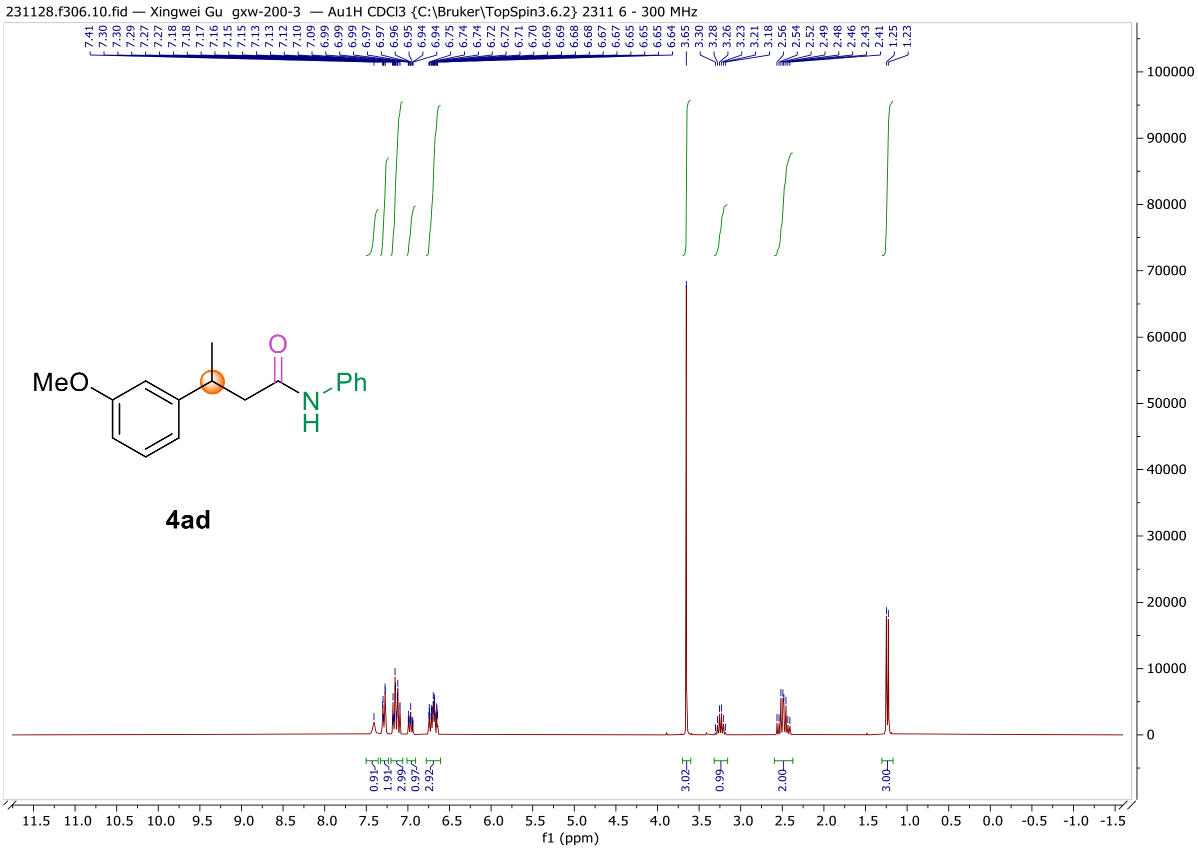
231128.f306.10.fid — Xingwei Gu gxw-200-3 — Au1H CDCl3 {C:\Bruker\TopSpin3.6.2} 2311 6 - 300 MHz
MeO
O
N
H
Ph
4ad
f1 (ppm)

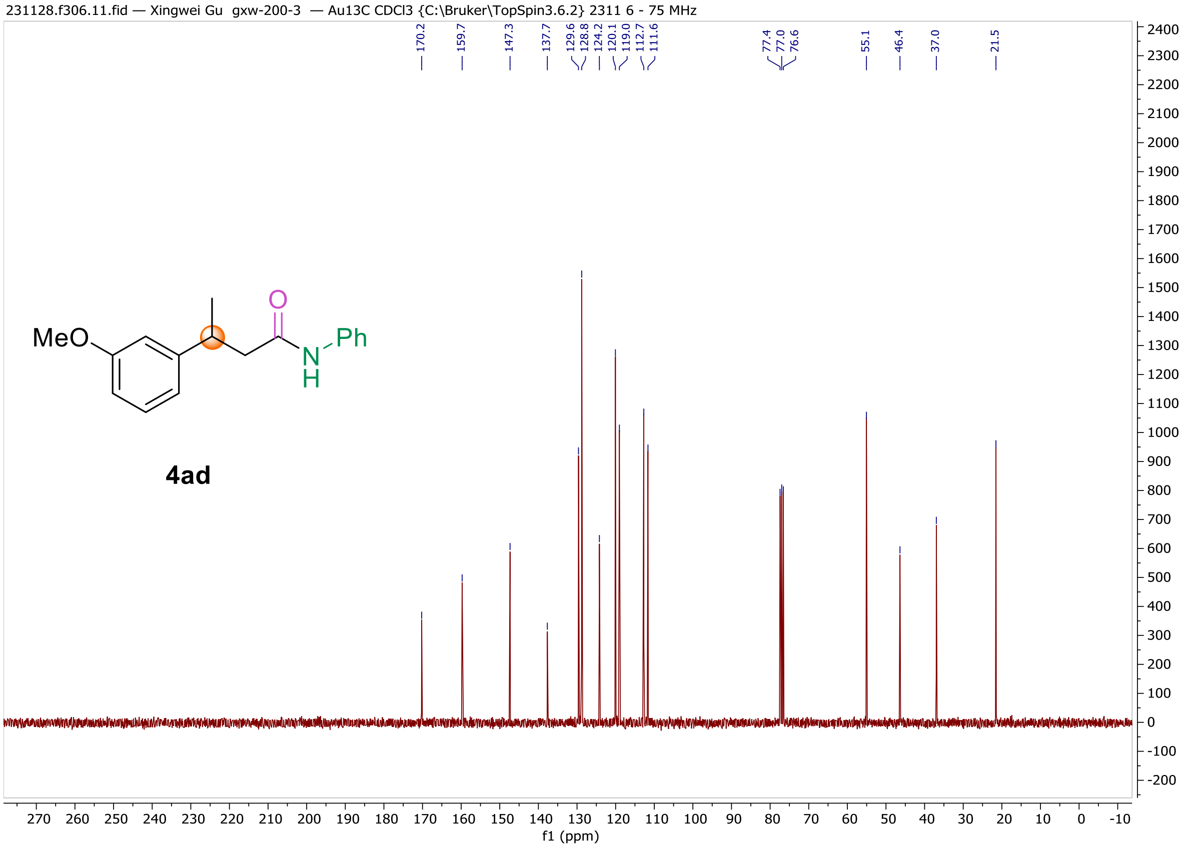
231128.f306.11.fid — Xingwei Gu gxw-200-3 — Au13C CDCl3 {C:\Bruker\TopSpin3.6.2} 2311 6 - 75 MHz
170.2
159.7
147.3
137.7
129.6
128.8
124.2
120.1
119.0
112.7
111.6
77.4
77.0
76.6
55.1
46.4
37.0
21.5
MeO
O
N
H
Ph
4ad
f1 (ppm)

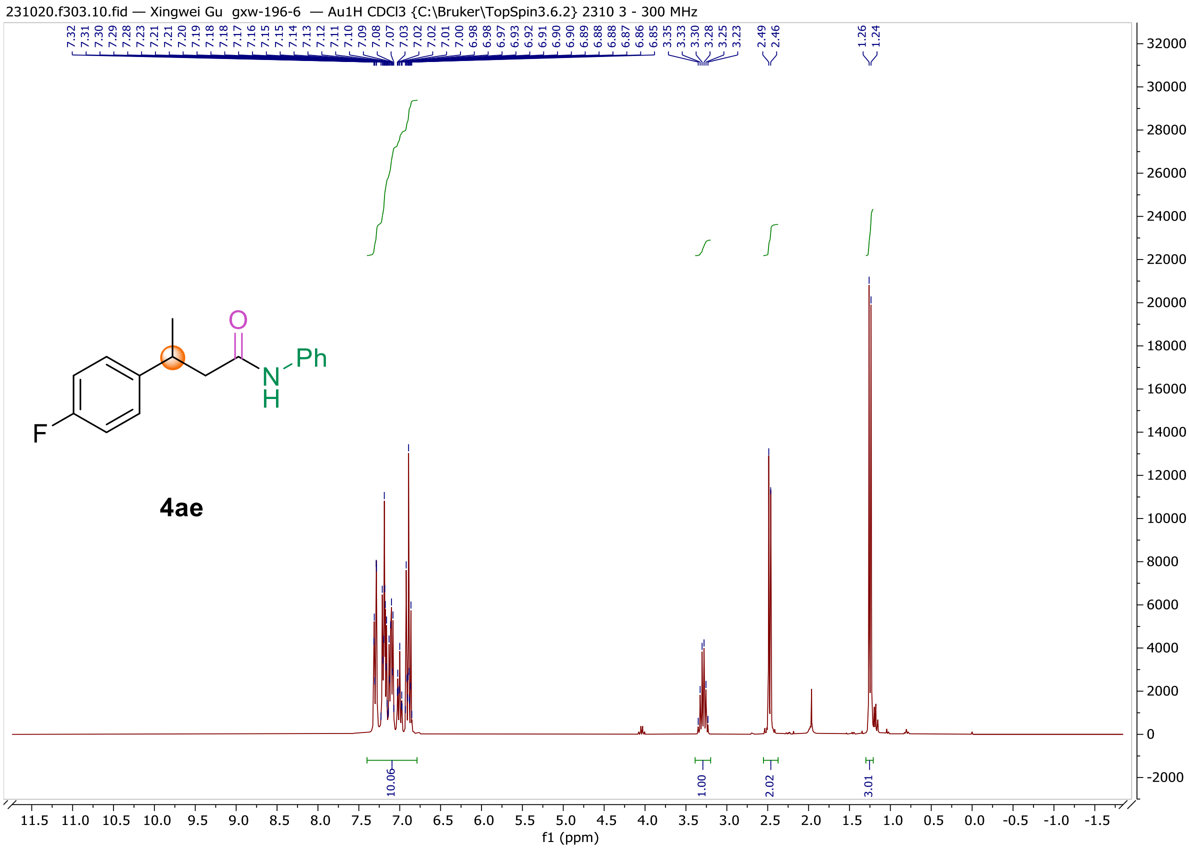
231020.f303.10.fid — Xingwei Gu gxw-196-6 — Au1H CDCl3 {C:\Bruker\TopSpin3.6.2} 2310 3 - 300 MHz
7.32 7.31 7.30 7.29 7.28 7.23 7.21 7.21 7.20 7.19 7.18 7.18 7.17 7.16 7.15 7.15 7.14 7.13 7.12 7.11 7.10 7.09 7.08 7.07 7.03 7.02 7.02 7.01 7.00 6.98 6.98 6.97 6.93 6.92 6.91 6.90 6.90 6.89 6.88 6.88 6.87 6.86 6.85 3.35 3.33 3.30 3.28 3.25 3.23 2.49 2.46 1.26 1.24
O
Ph
N
H
F
4ae
10.06
1.00
2.02
3.01
f1 (ppm)

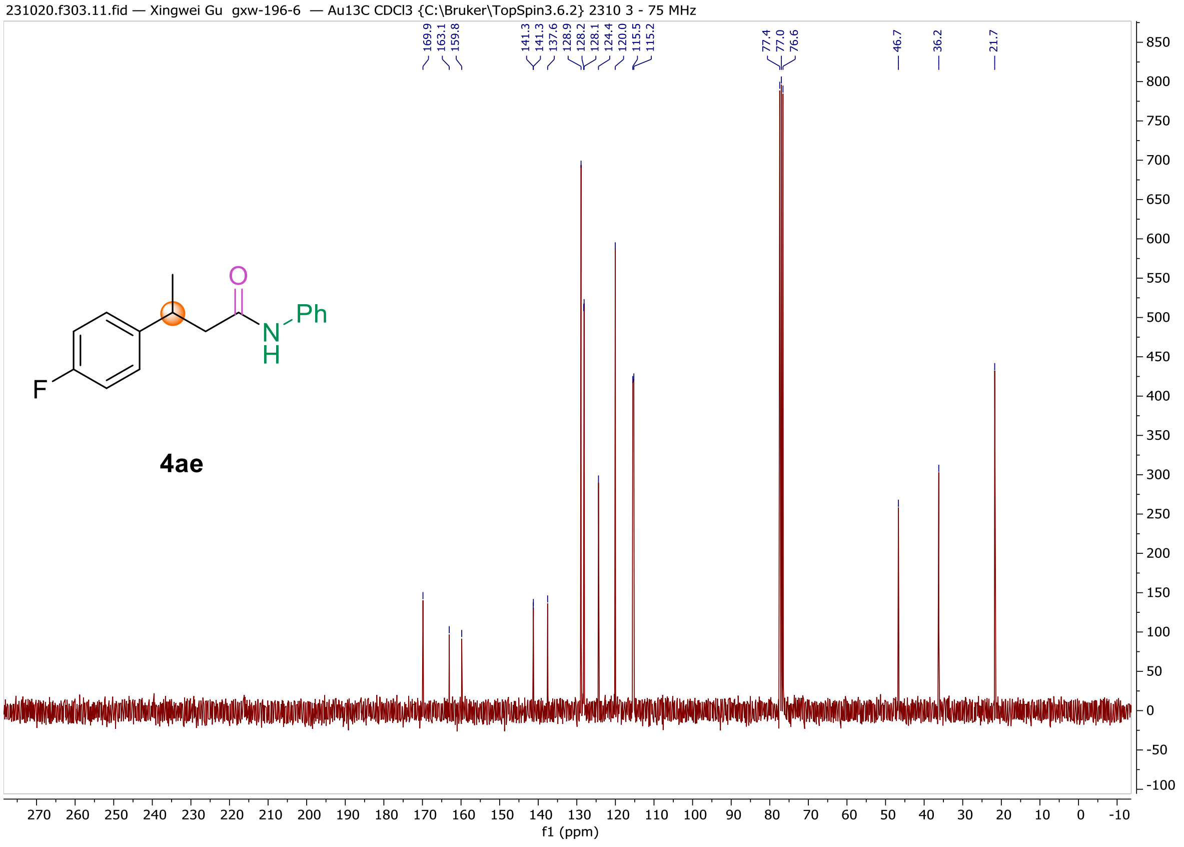
231020.f303.11.fid — Xingwei Gu gxw-196-6 — Au13C CDCl3 {C:\Bruker\TopSpin3.6.2} 2310 3 - 75 MHz
169.9 163.1 159.8 141.3 141.3 137.6 128.9 128.2 128.1 124.4 120.0 115.5 115.2 77.4 77.0 76.6 46.7 36.2 21.7
O
Ph
N
H
F
4ae
f1 (ppm)

231020.f303.12.fid — Xingwei Gu gxw-196-6 — Au19F(H-gek) CDCl3 {C:\Bruker\TopSpin3.6.2} 2310 3 - 75 MHz

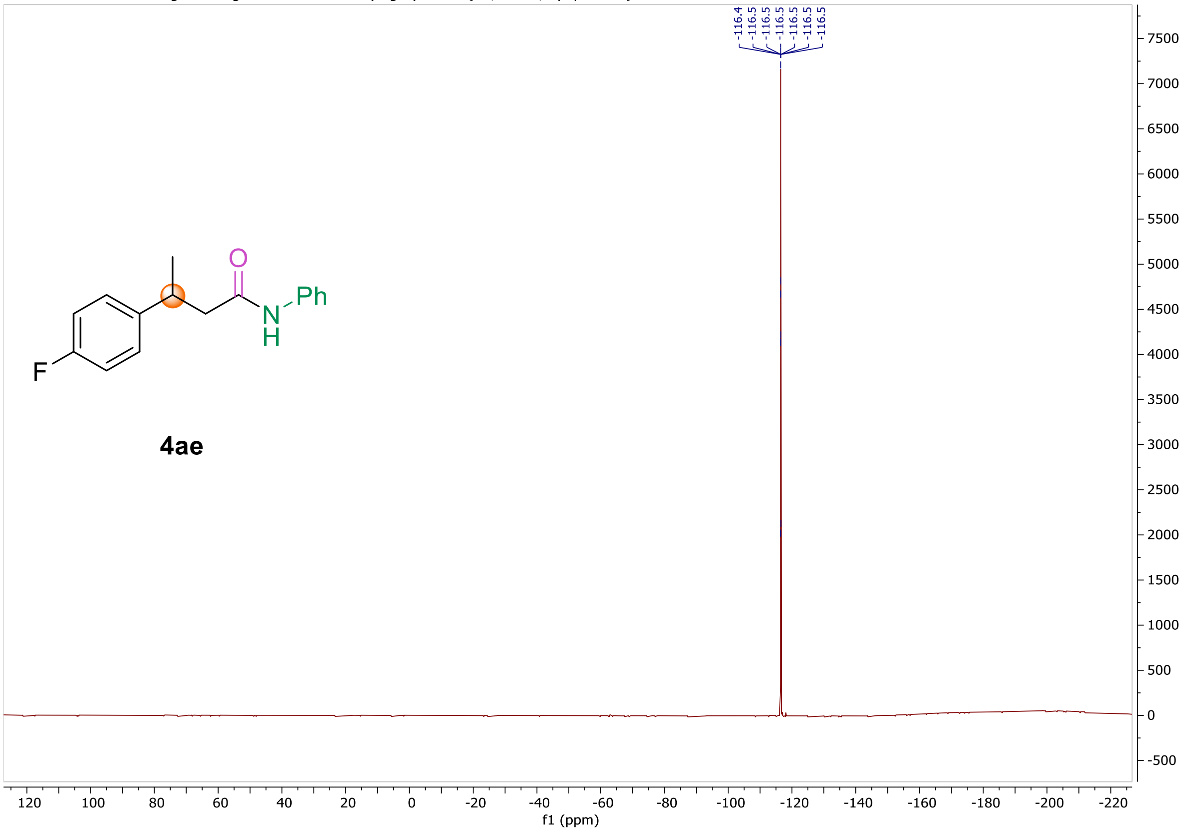



**4ae**

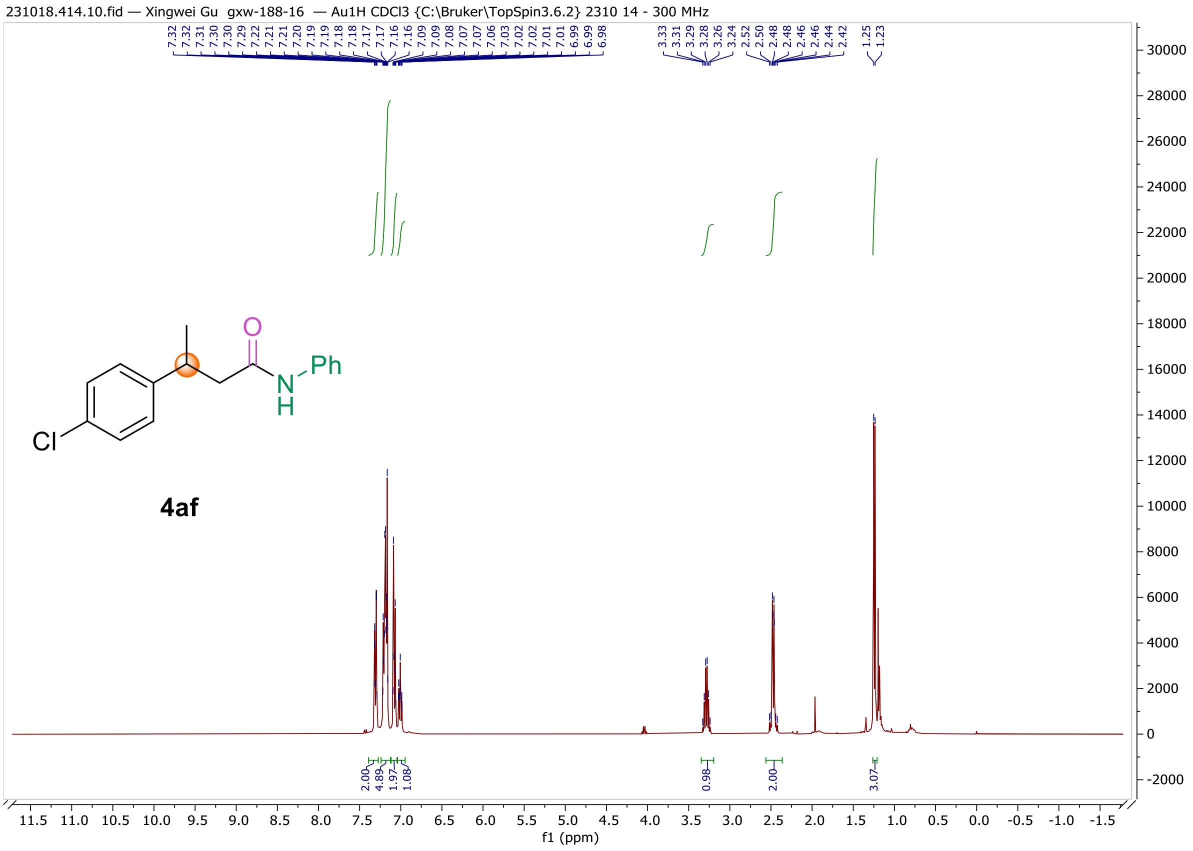
231018.414.10.fid — Xingwei Gu gxw-188-16 — Au1H CDCl3 {C:\Bruker\TopSpin3.6.2} 2310 14 - 300 MHz
4af
f1 (ppm)

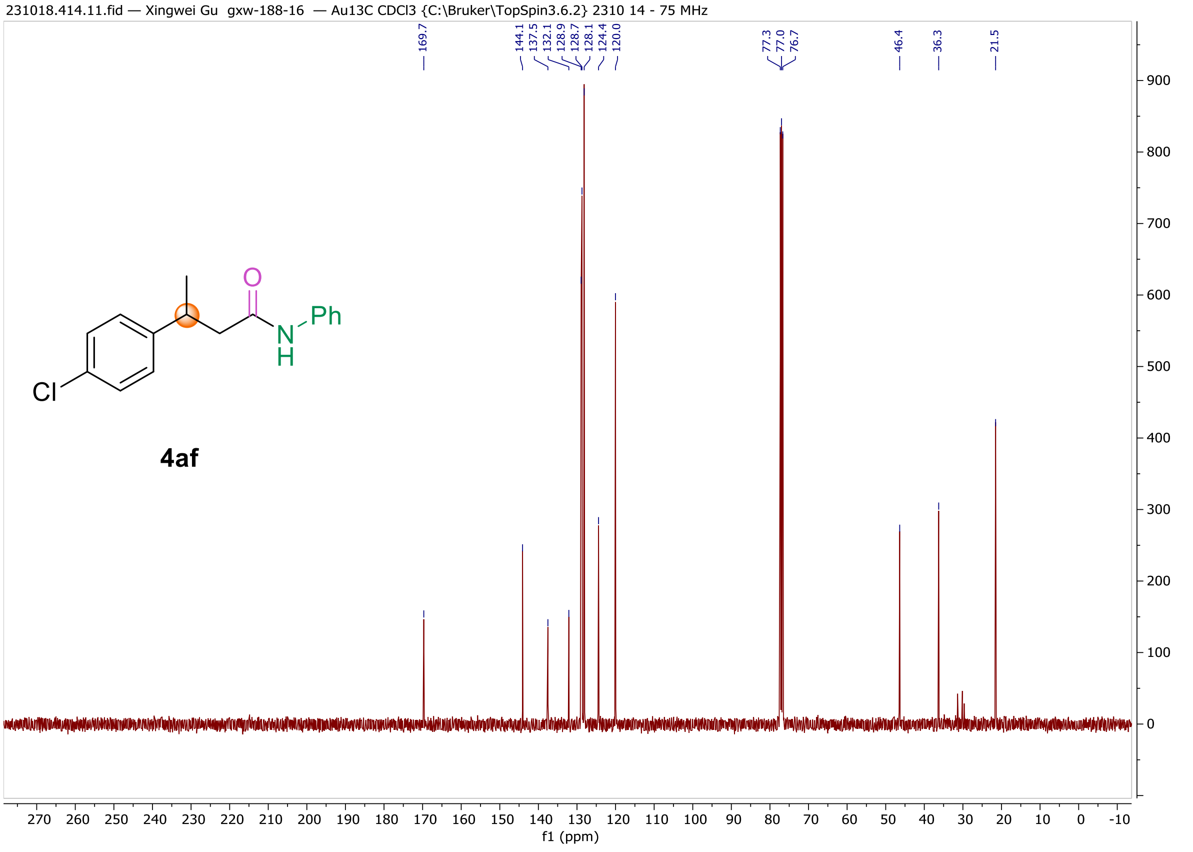
231018.414.11.fid — Xingwei Gu gxw-188-16 — Au13C CDCl3 {C:\Bruker\TopSpin3.6.2} 2310 14 - 75 MHz
169.7
144.1
137.5
132.1
128.9
128.7
128.1
124.4
120.0
77.3
77.0
76.7
46.4
36.3
21.5
4af
f1 (ppm)

231019.f305.10.fid — Xingwei Gu gxw-188-24 — Au1H CDCl3 {C:\Bruker\TopSpin3.6.2} 2310 5 - 300 MHz

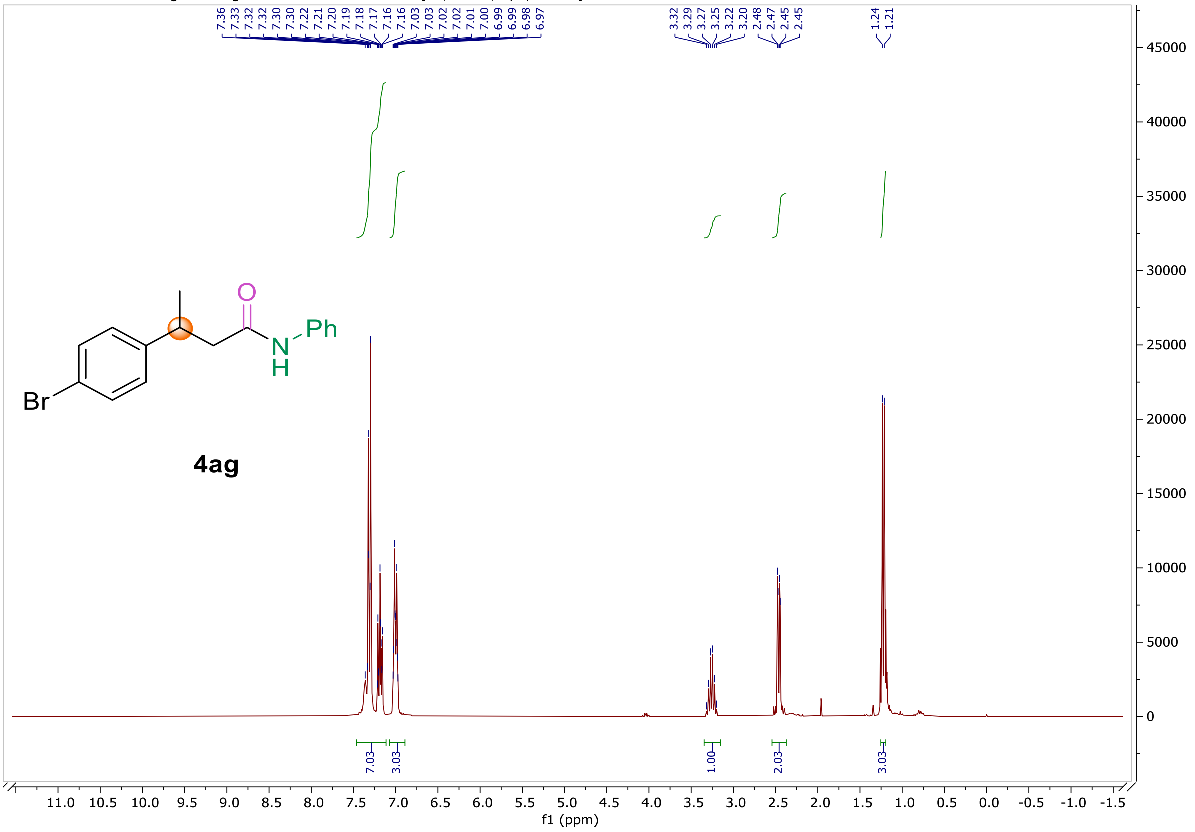


231019.f305.11.fid — Xingwei Gu gxw-188-24 — Au13C CDCl3 {C:\Bruker\TopSpin3.6.2} 2310 5 - 75 MHz

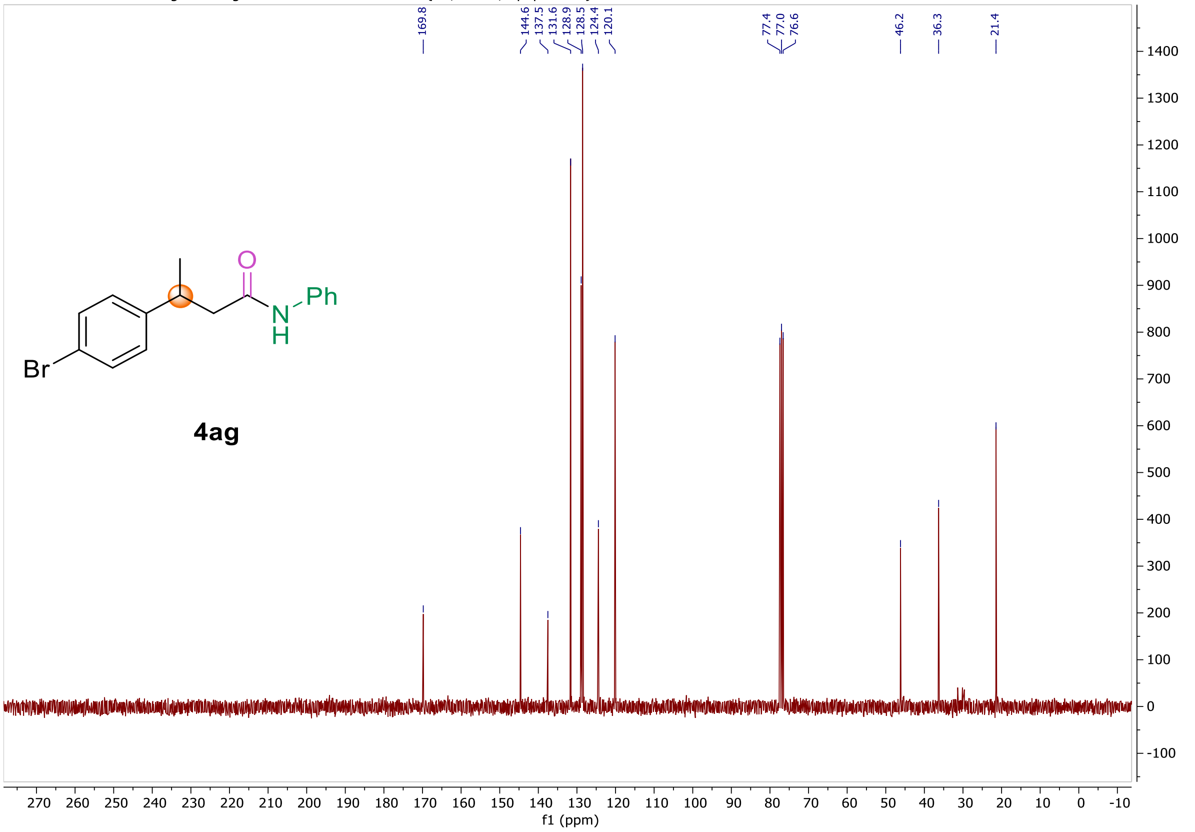

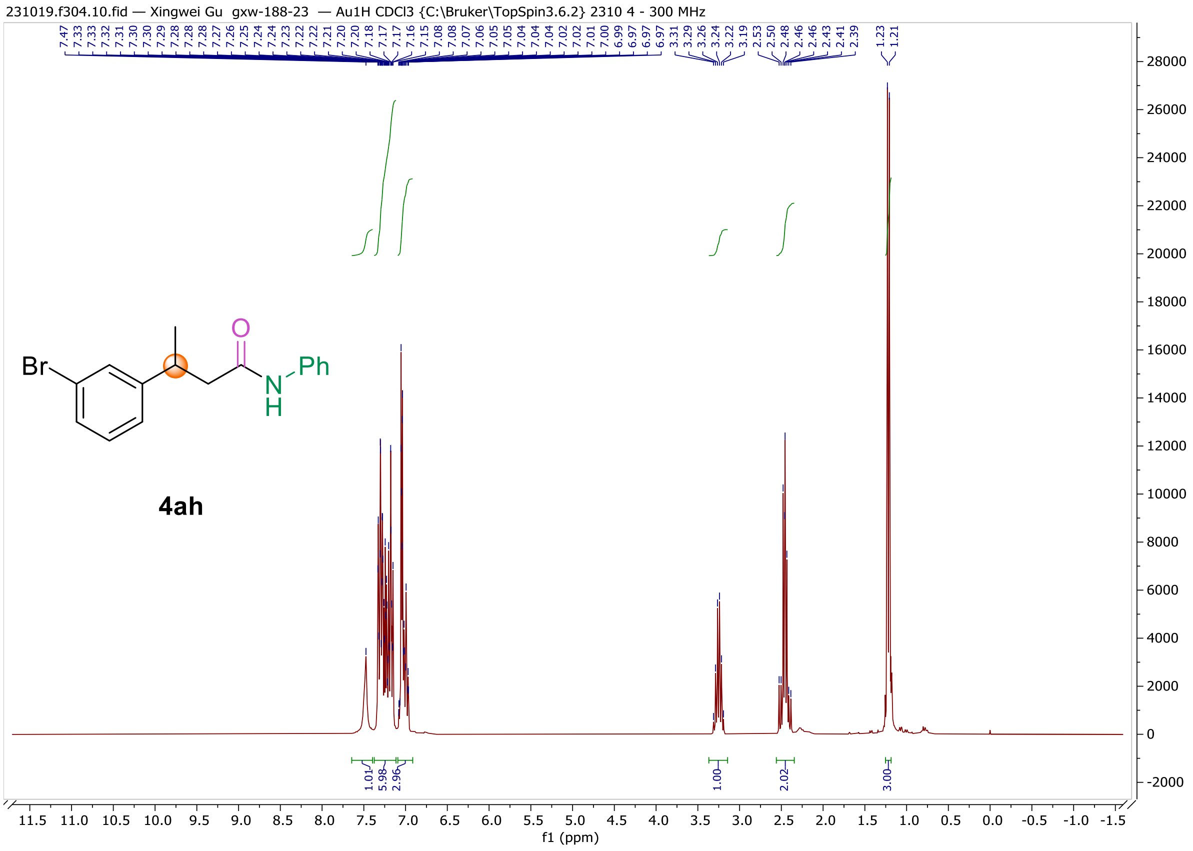

231019.f304.10.fid — Xingwei Gu gxw-188-23 — Au1H CDCl3 {C:\Bruker\TopSpin3.6.2} 2310 4 - 300 MHz
Br
O
N
Ph
H
4ah
f1 (ppm)


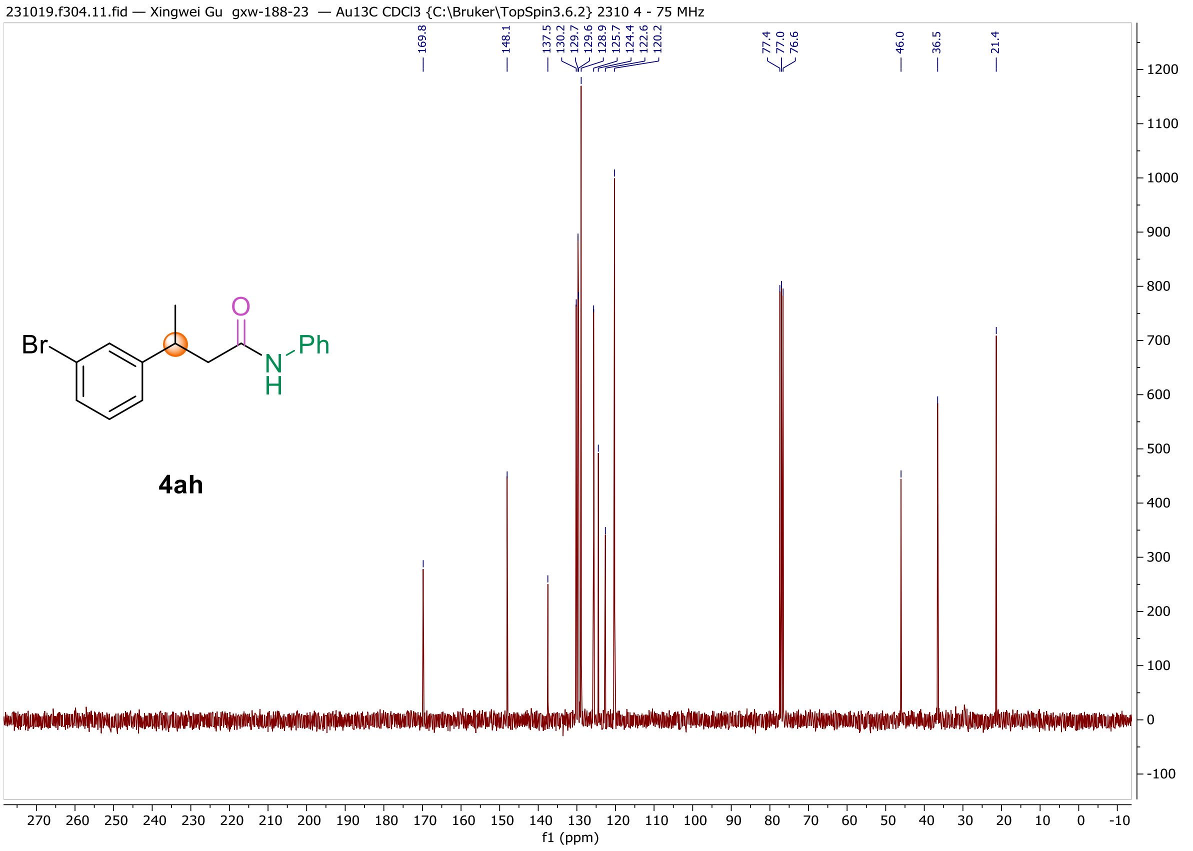

231019.f304.11.fid — Xingwei Gu gxw-188-23 — Au13C CDCl3 {C:\Bruker\TopSpin3.6.2} 2310 4 - 75 MHz
169.8
148.1
137.5
130.2
129.7
129.6
128.9
125.7
124.4
122.6
120.2
77.4
77.0
76.6
46.0
36.5
21.4
Br
O
N
Ph
H
4ah
f1 (ppm)

231020.f301.10.fid — Xingwei Gu gxw-196-4 — Au1H CDCl3 {C:\Bruker\TopSpin3.6.2} 2310 1 - 300 MHz

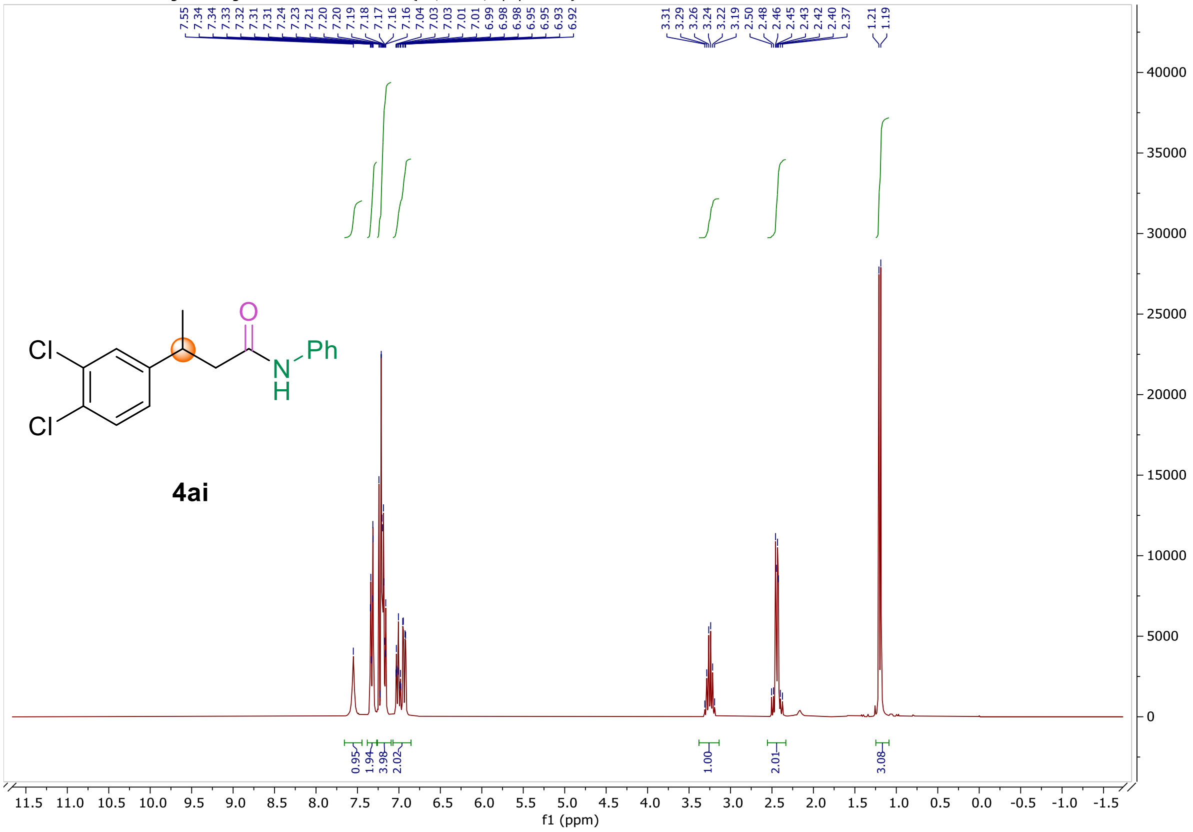


231020.f301.11.fid — Xingwei Gu gxw-196-4 — Au13C CDCl3 {C:\Bruker\TopSpin3.6.2} 2310 1 - 75 MHz

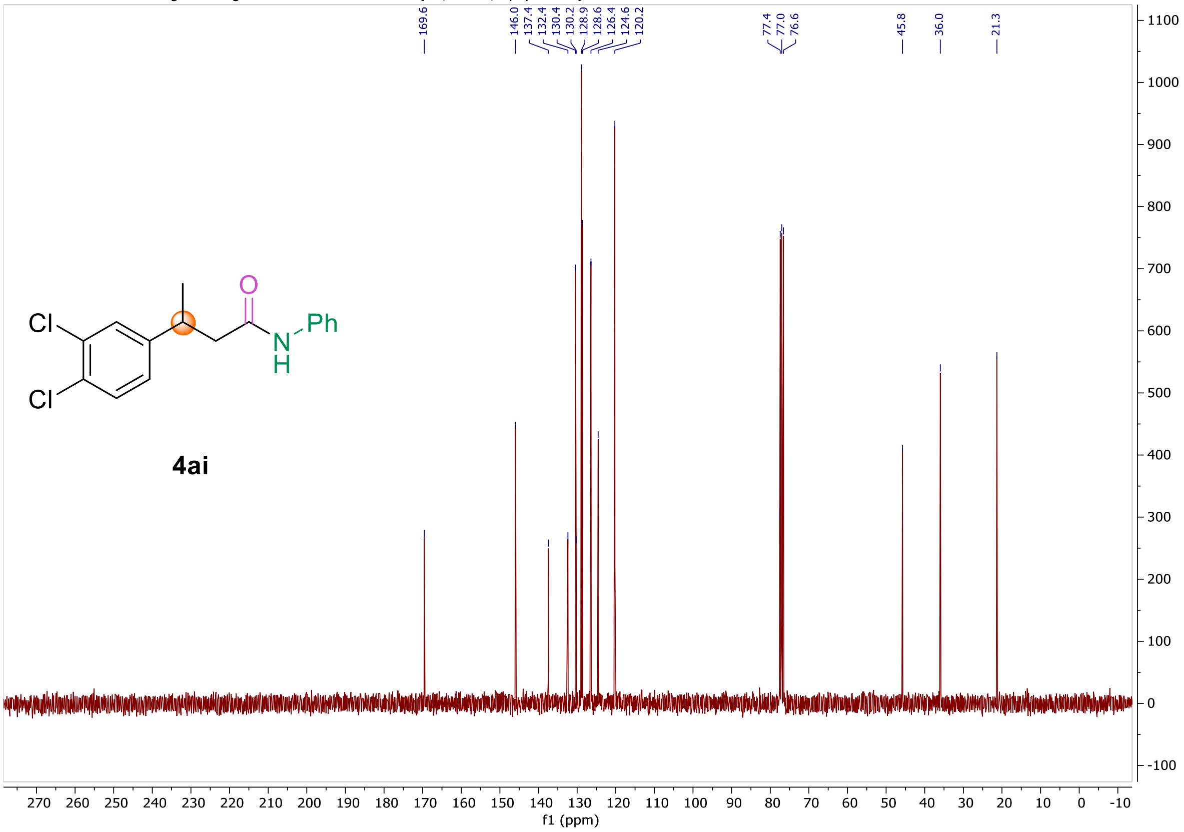

231020.f302.10.fid — Xingwei Gu gxw-196-5 — Au1H CDCl3 {C:\Bruker\TopSpin3.6.2} 2310 2 - 300 MHz

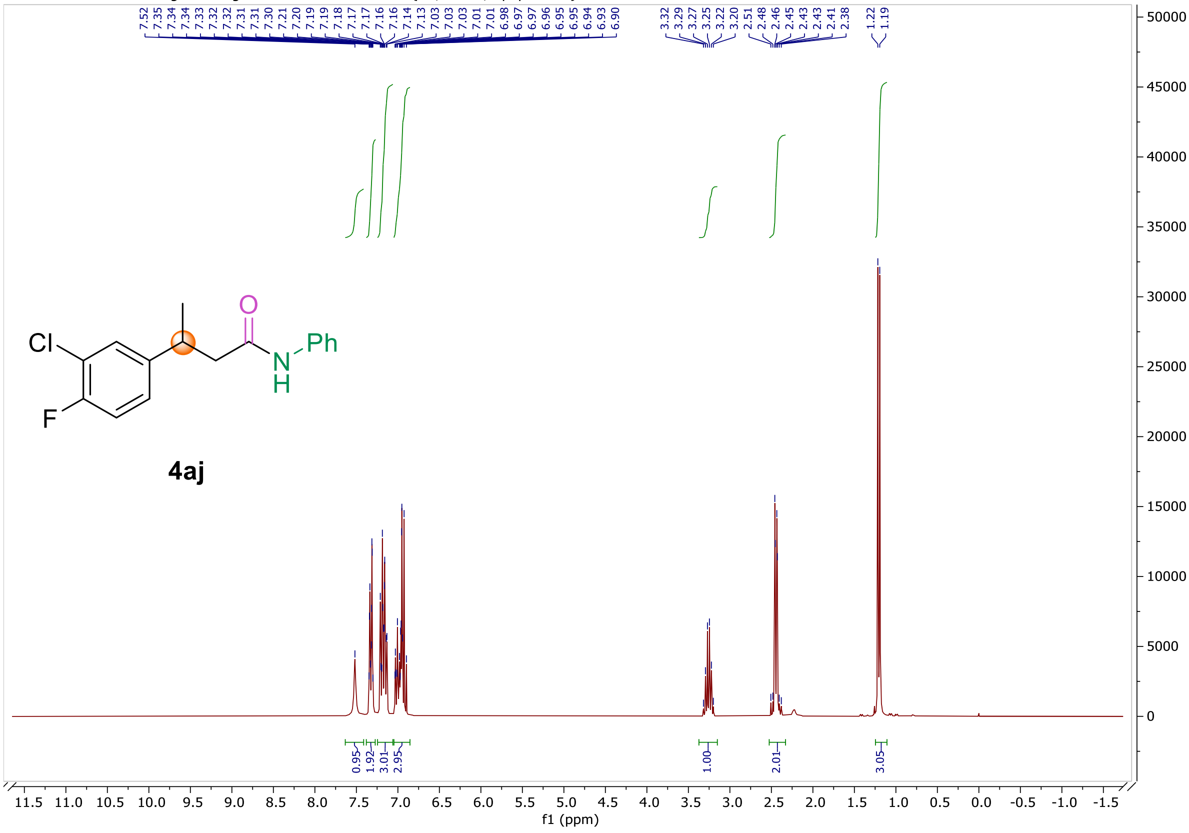


231020.f302.11.fid — Xingwei Gu gxw-196-5 — Au13C CDCl3 {C:\Bruker\TopSpin3.6.2} 2310 2 - 75 MHz

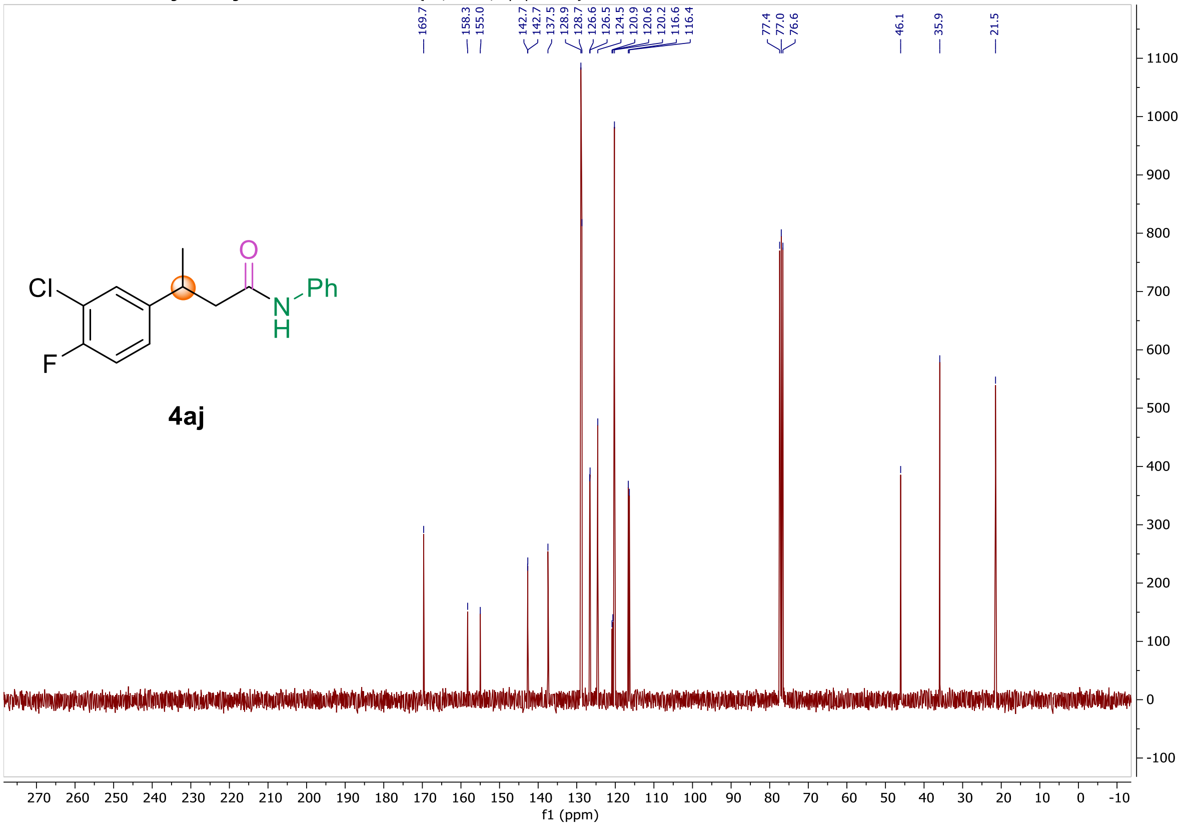

231020.f302.12.fid — Xingwei Gu gxw-196-5 — Au19F(H-gek) CDCl3 {C:\Bruker\TopSpin3.6.2} 2310 2 - 75 MHz

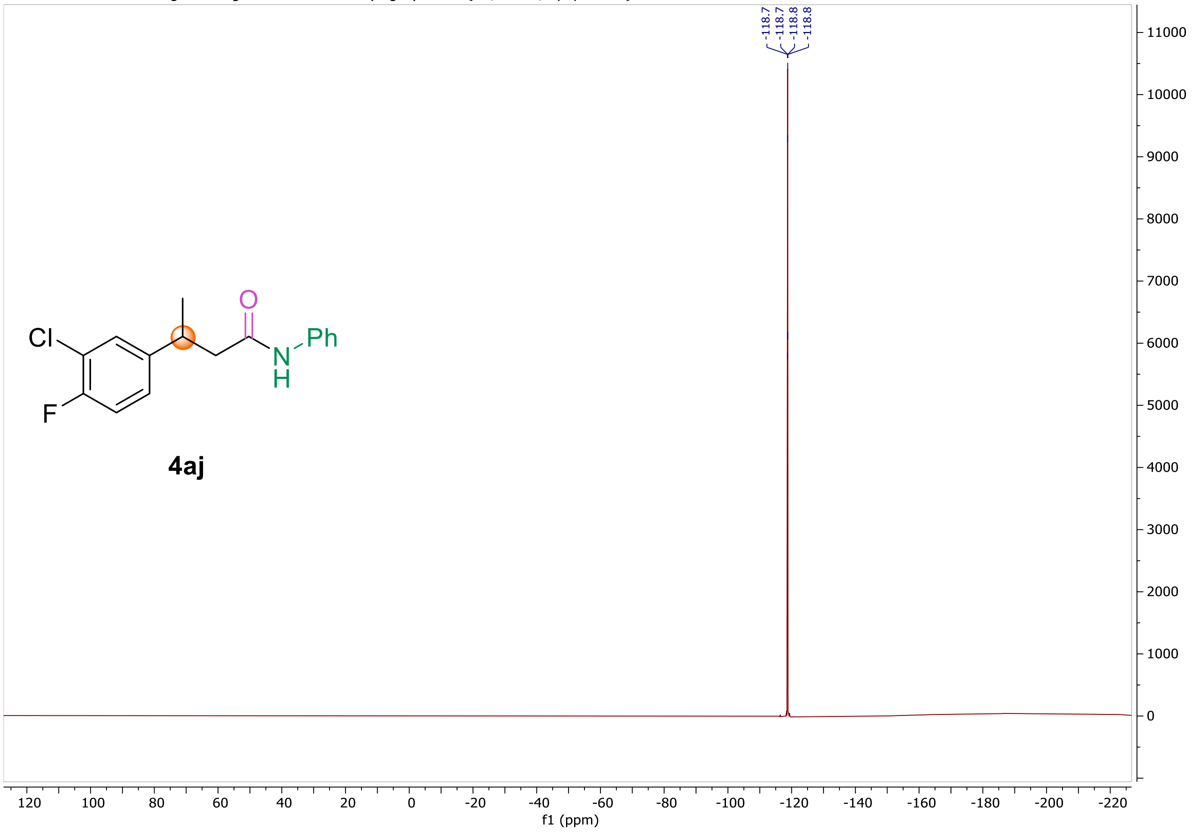


**4aj**

230914.307.10.fid — Xingwei Gu gxw-188-11 — Au1H CDCl3 {C:\Bruker\TopSpin3.6.2} 2309 7 - 300 MHz

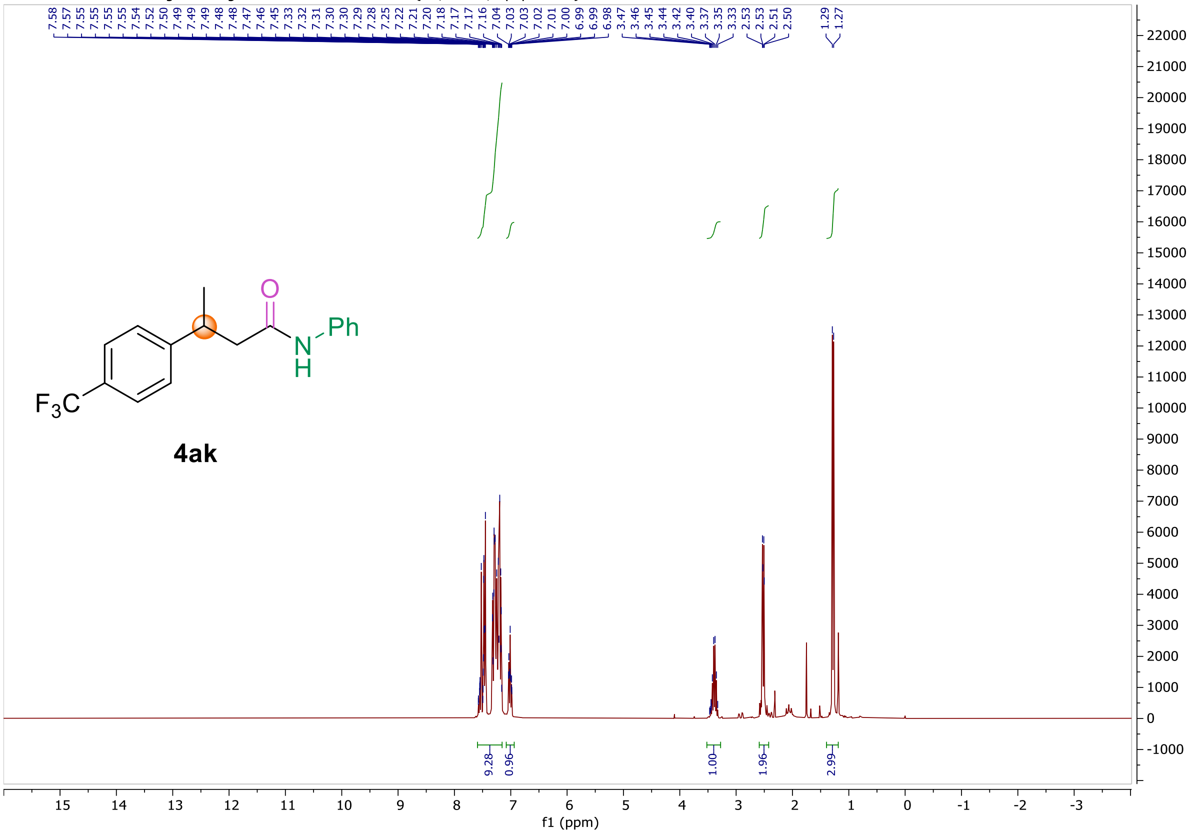


230914.307.11.fid — Xingwei Gu gxw-188-11 — Au13C CDCl3 {C:\Bruker\TopSpin3.6.2} 2309 7 - 75 MHz

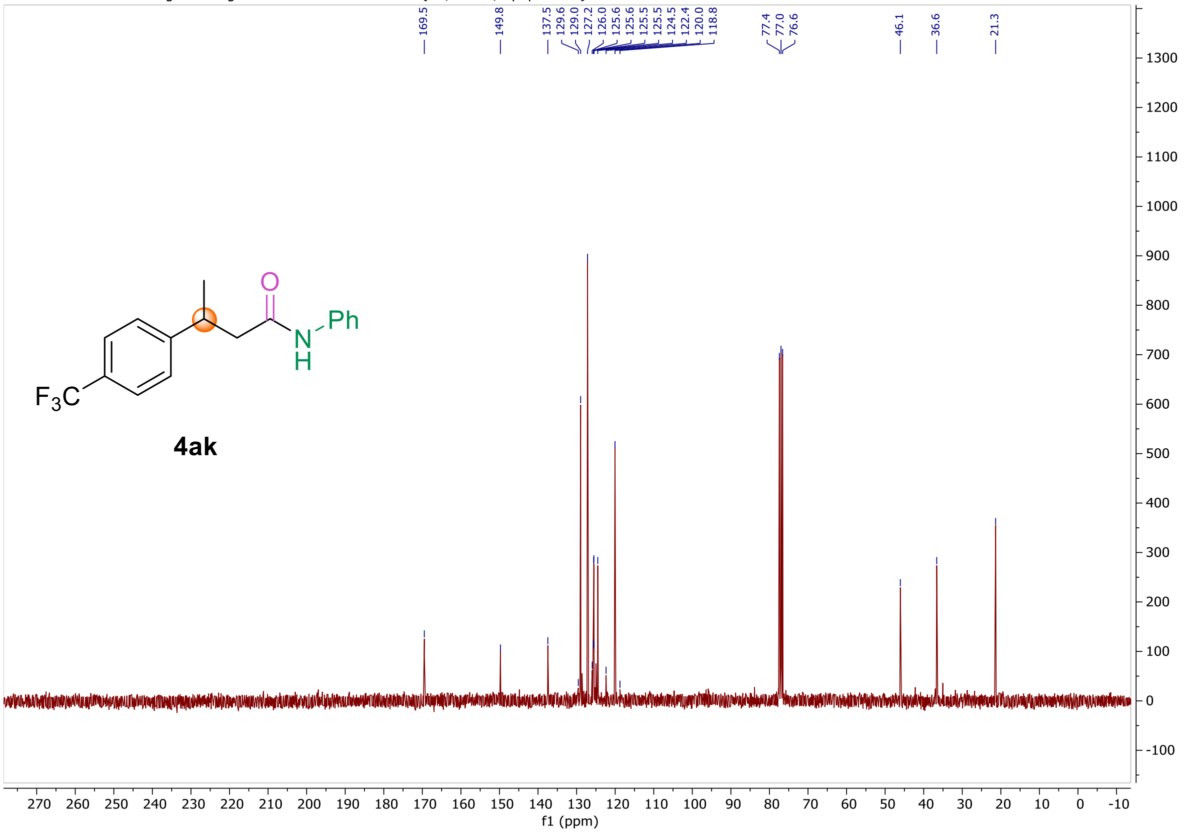

230914.307.12.fid — Xingwei Gu gxw-188-11 — Au19F CDCl3 {C:\Bruker\TopSpin3.6.2} 2309 7-282 MHz

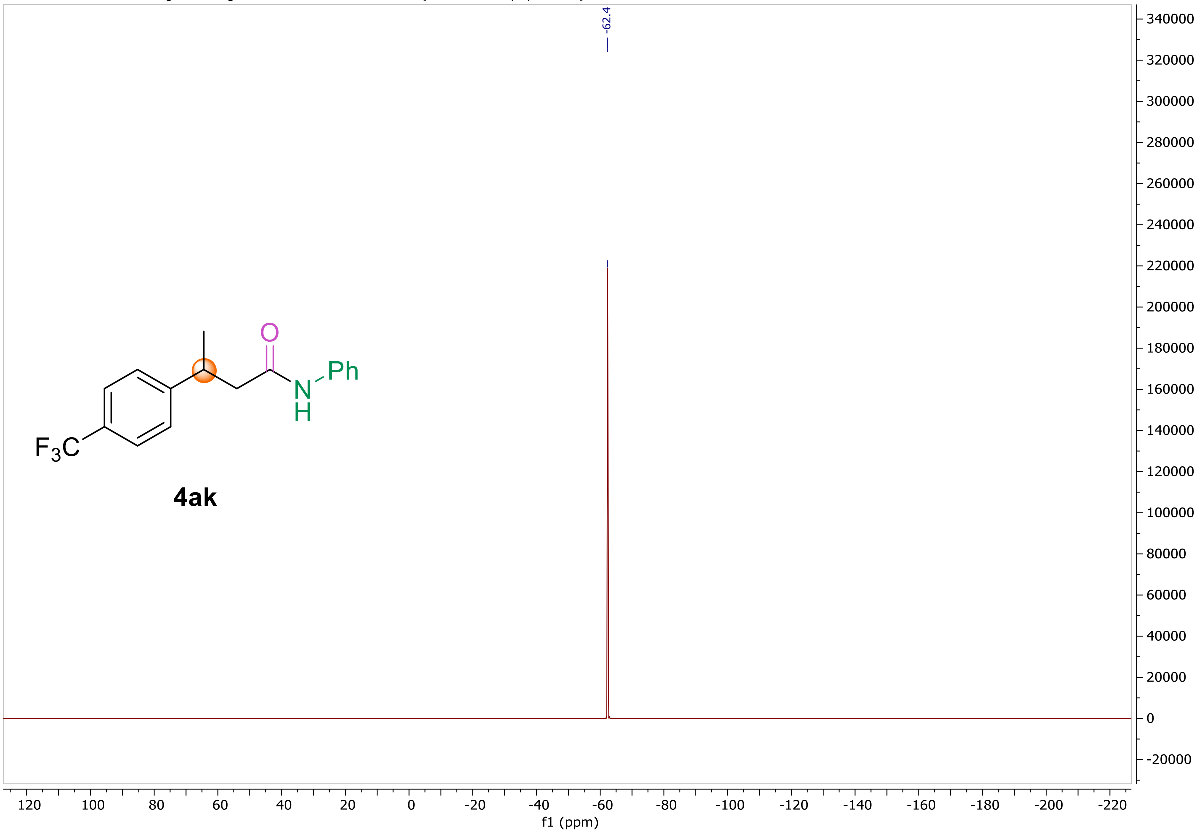


4ak

231030.f309.10.fid — Xingwei Gu gxw-196-10 — Au1H CDCl3 {C:\Bruker\TopSpin3.6.2} 2310 9 - 300 MHz

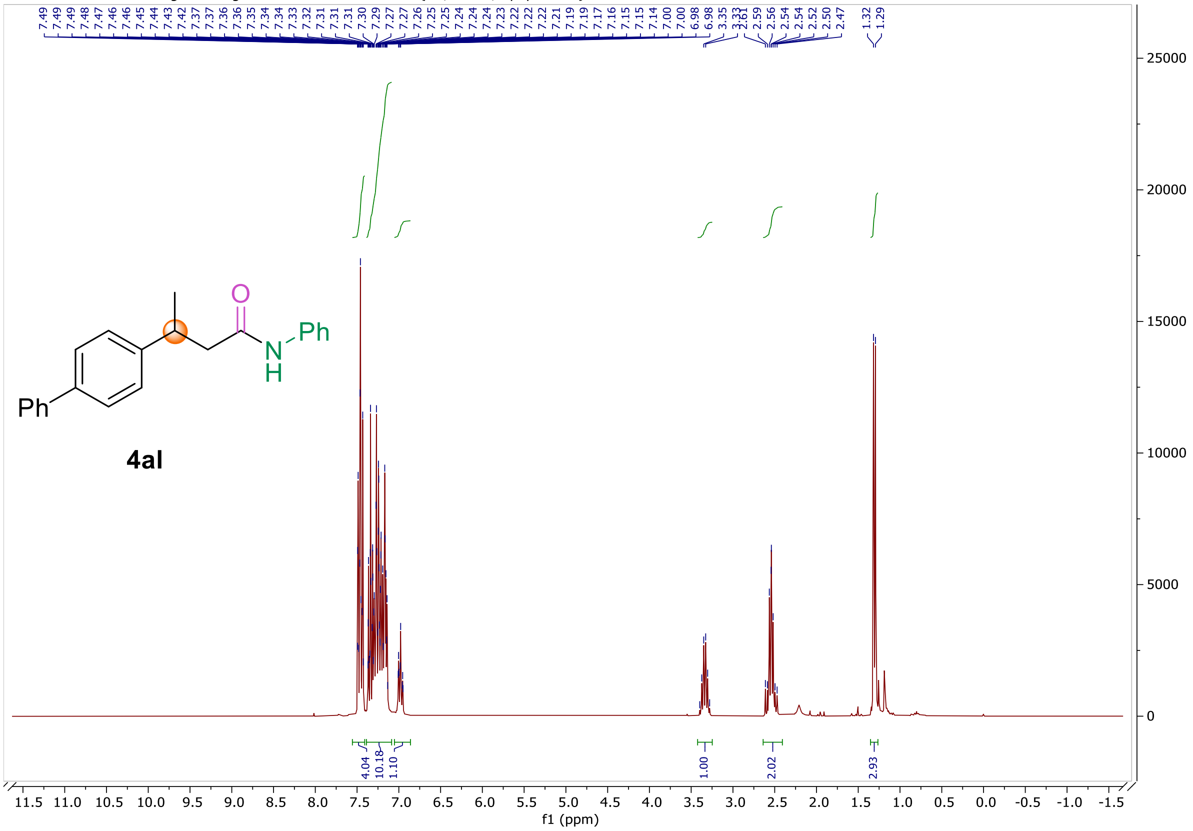

231030.f309.11.fid — Xingwei Gu gxw-196-10 — Au13C CDCl3 {C:\Bruker\TopSpin3.6.2} 2310 9 - 75 MHz

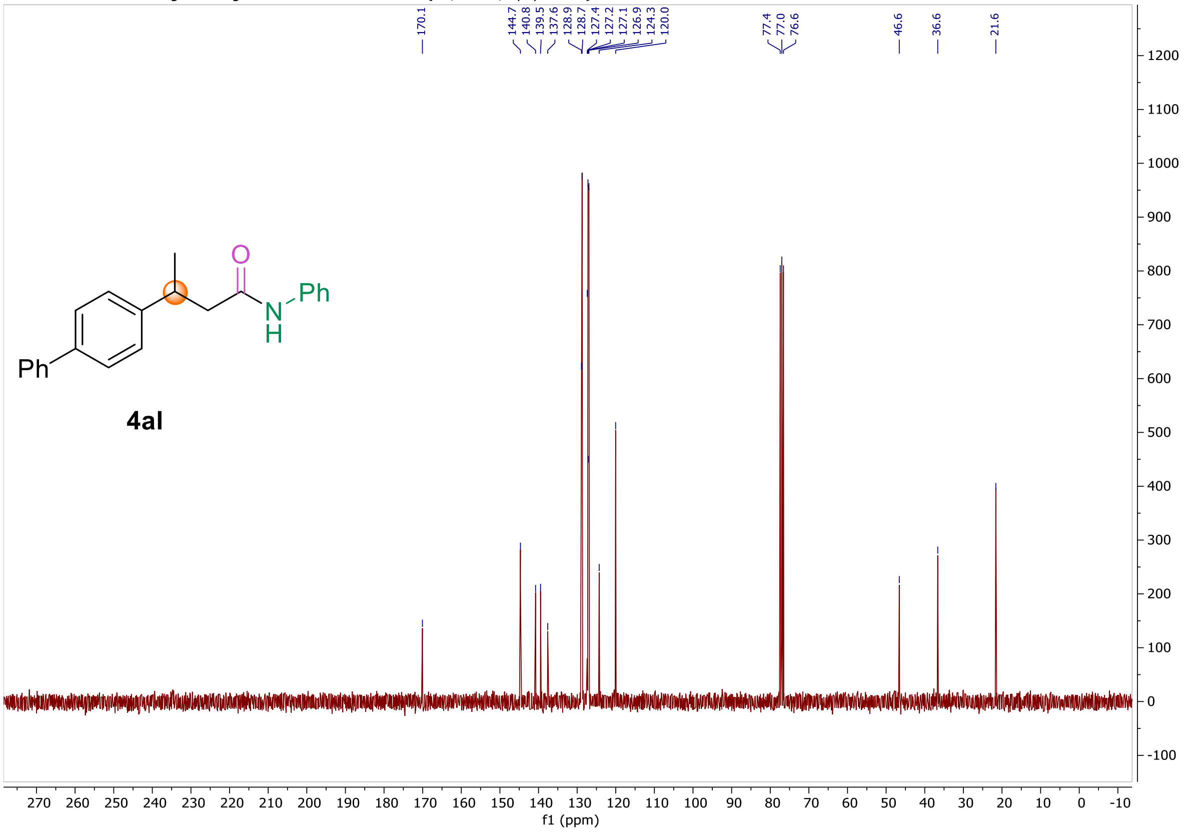

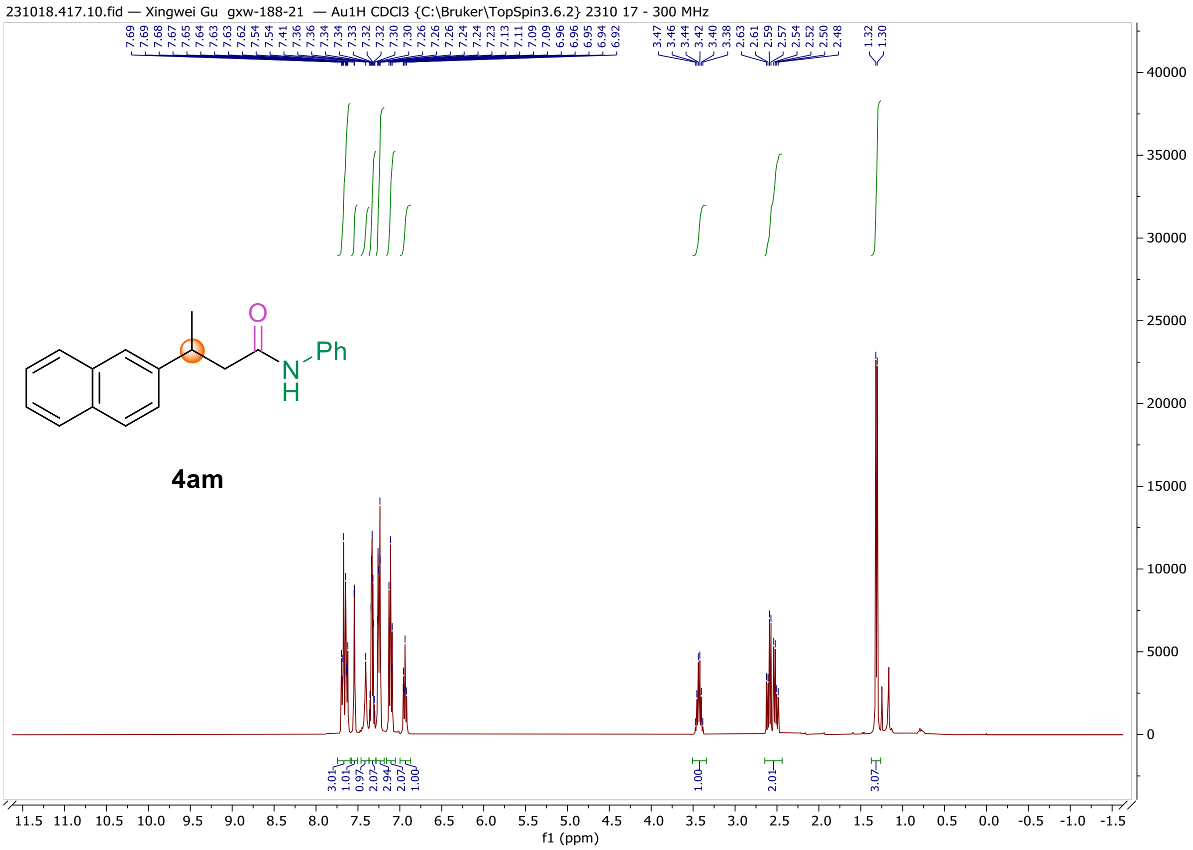
231018.417.10.fid — Xingwei Gu gxw-188-21 — Au1H CDCl3 {C:\Bruker\TopSpin3.6.2} 2310 17 - 300 MHz
4am
Ph
O
N
H
f1 (ppm)

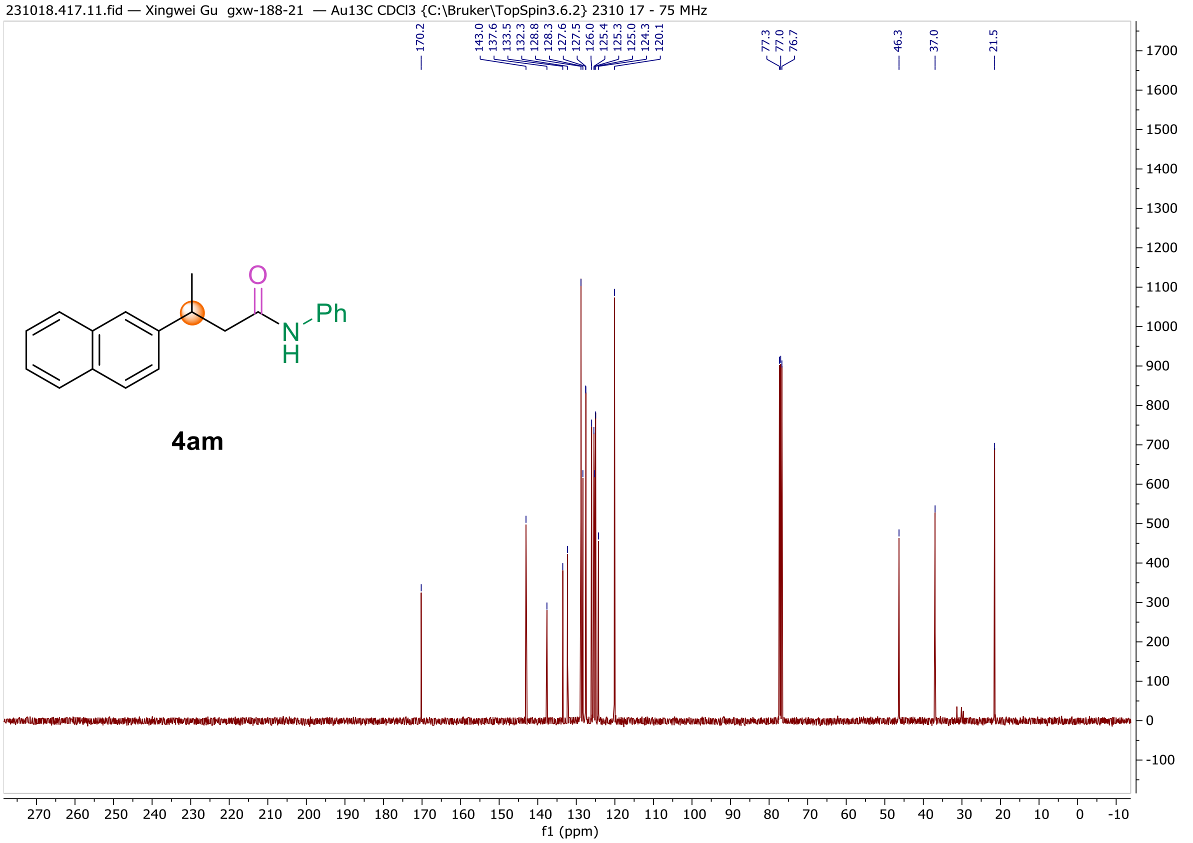
231018.417.11.fid — Xingwei Gu gxw-188-21 — Au13C CDCl3 {C:\Bruker\TopSpin3.6.2} 2310 17 - 75 MHz
170.2
143.0
137.6
133.5
132.3
128.8
128.3
127.6
127.5
126.0
125.4
125.3
125.0
124.3
120.1
77.3
77.0
76.7
46.3
37.0
21.5
4am
f1 (ppm)

231019.f306.10.fid — Xingwei Gu gxw-196-1 — Au1H CDCl3 {C:\Bruker\TopSpin3.6.2} 2310 6 - 300 MHz

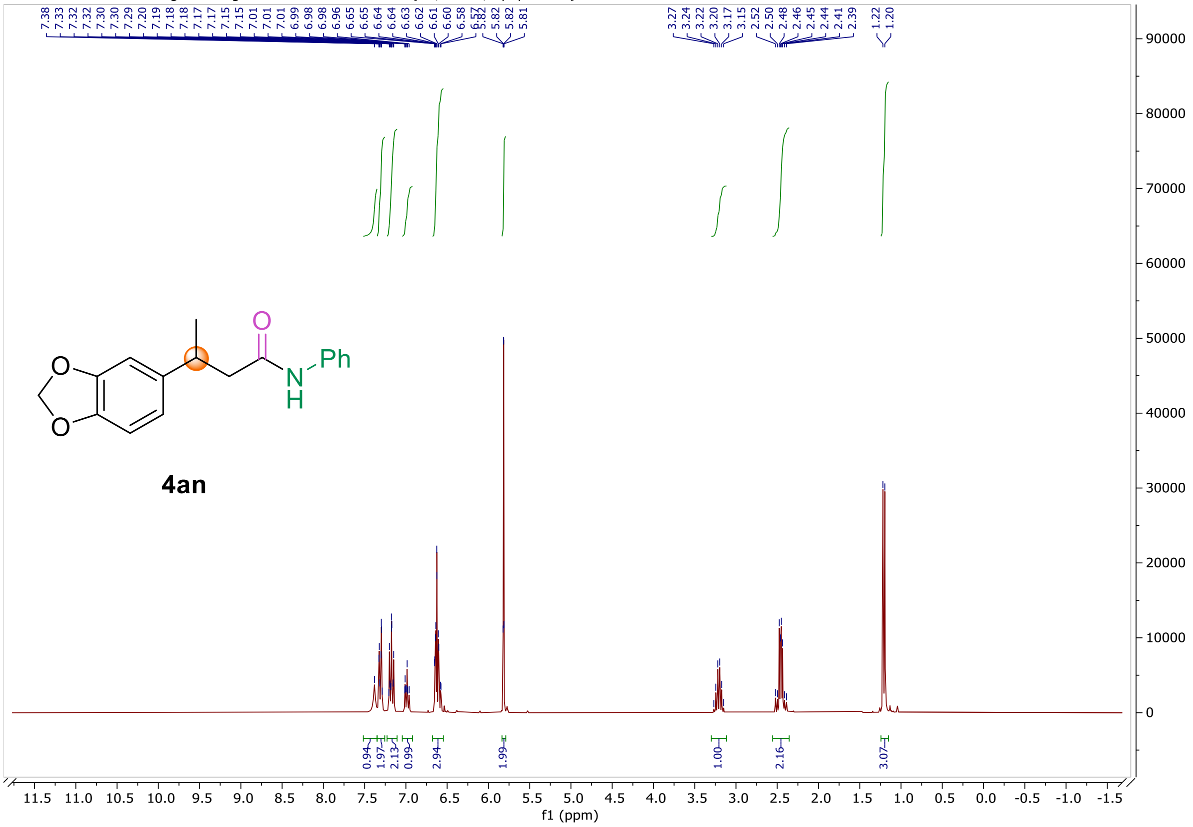


231019.f306.11.fid — Xingwei Gu gxw-196-1 — Au13C CDCl3 {C:\Bruker\TopSpin3.6.2} 2310 6 - 75 MHz

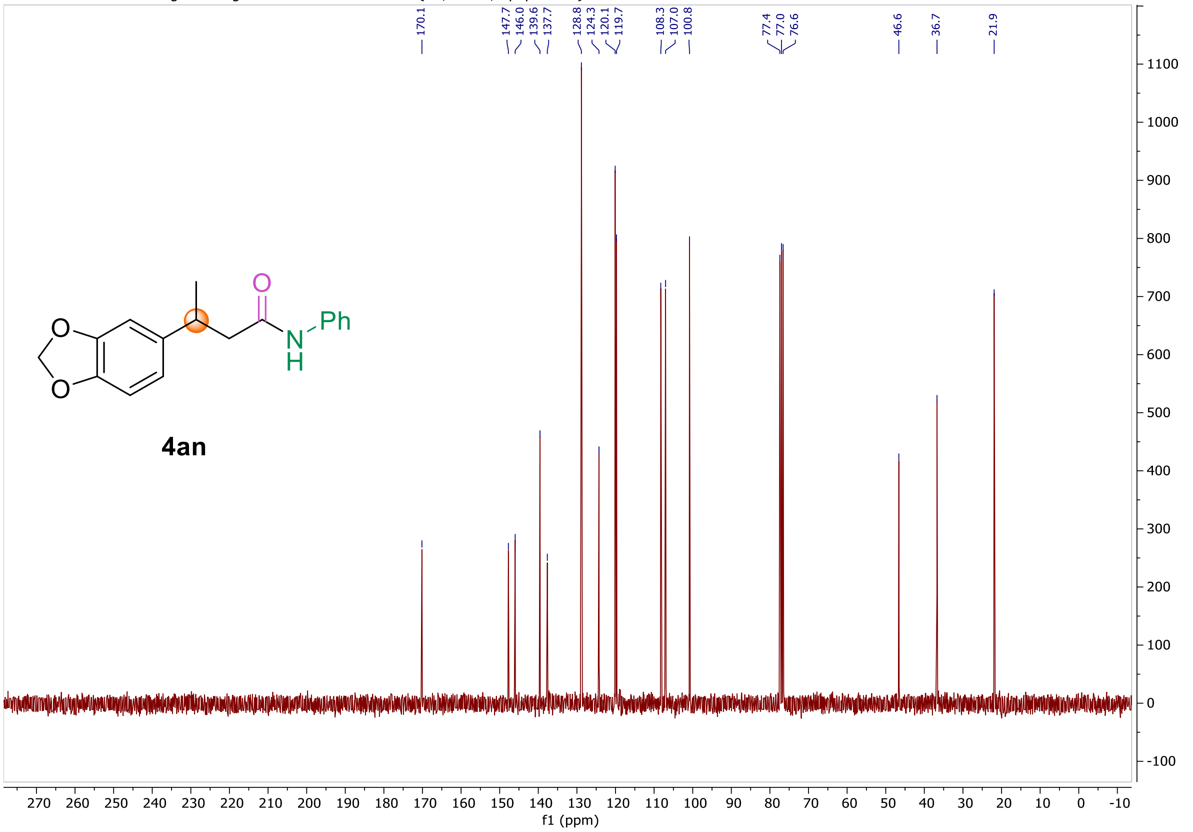

231201.309.10.fid — Xingwei Gu gxw-188-20 — Au1H CDCl3 {C:\Bruker\TopSpin3.6.2} 2312 9 - 300 MHz

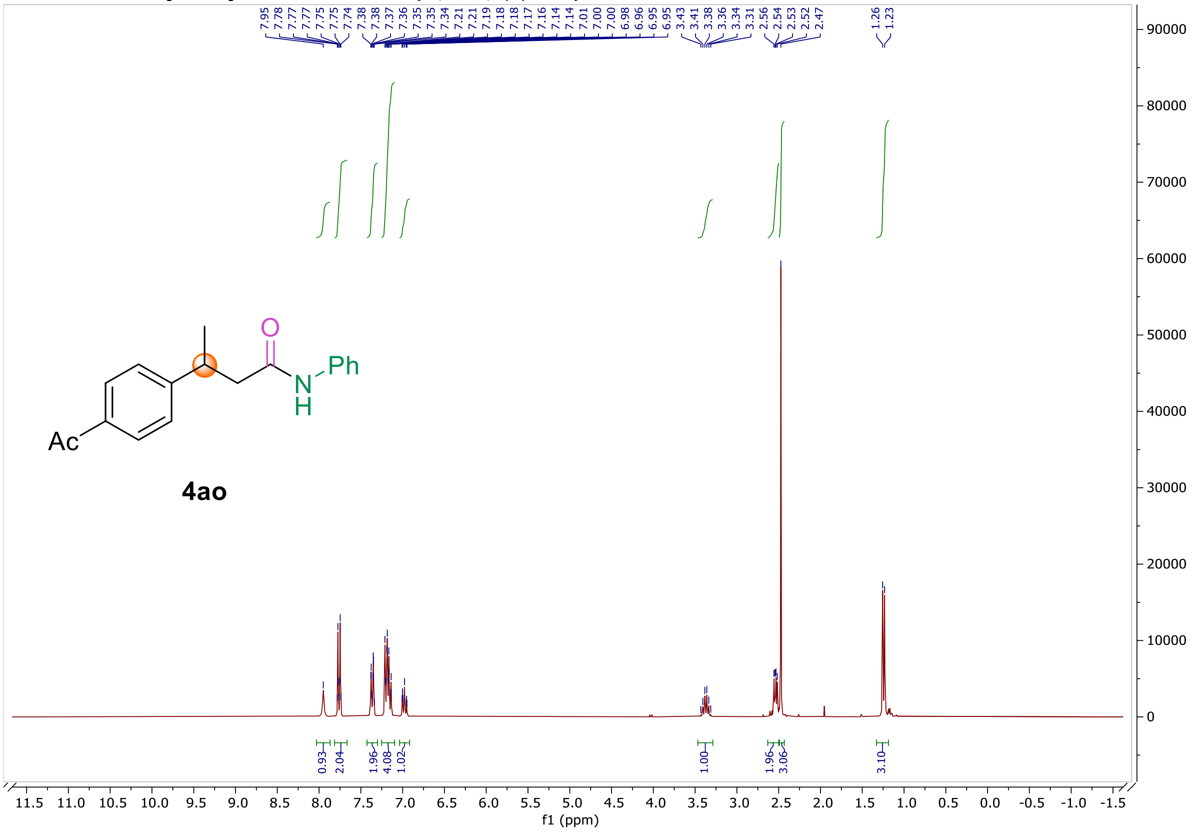


231201.309.11.fid — Xingwei Gu gxw-188-20 — Au13C CDCl3 {C:\Bruker\TopSpin3.6.2} 2312 9 - 75 MHz

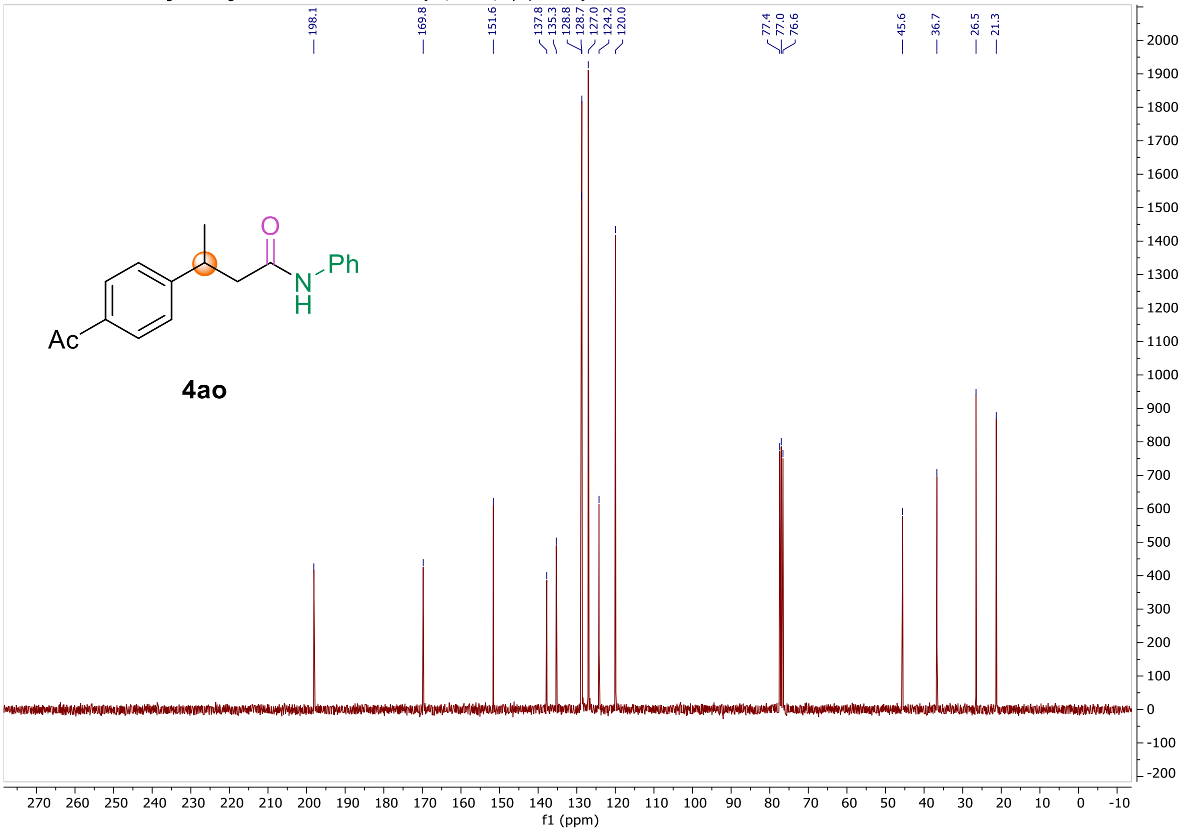

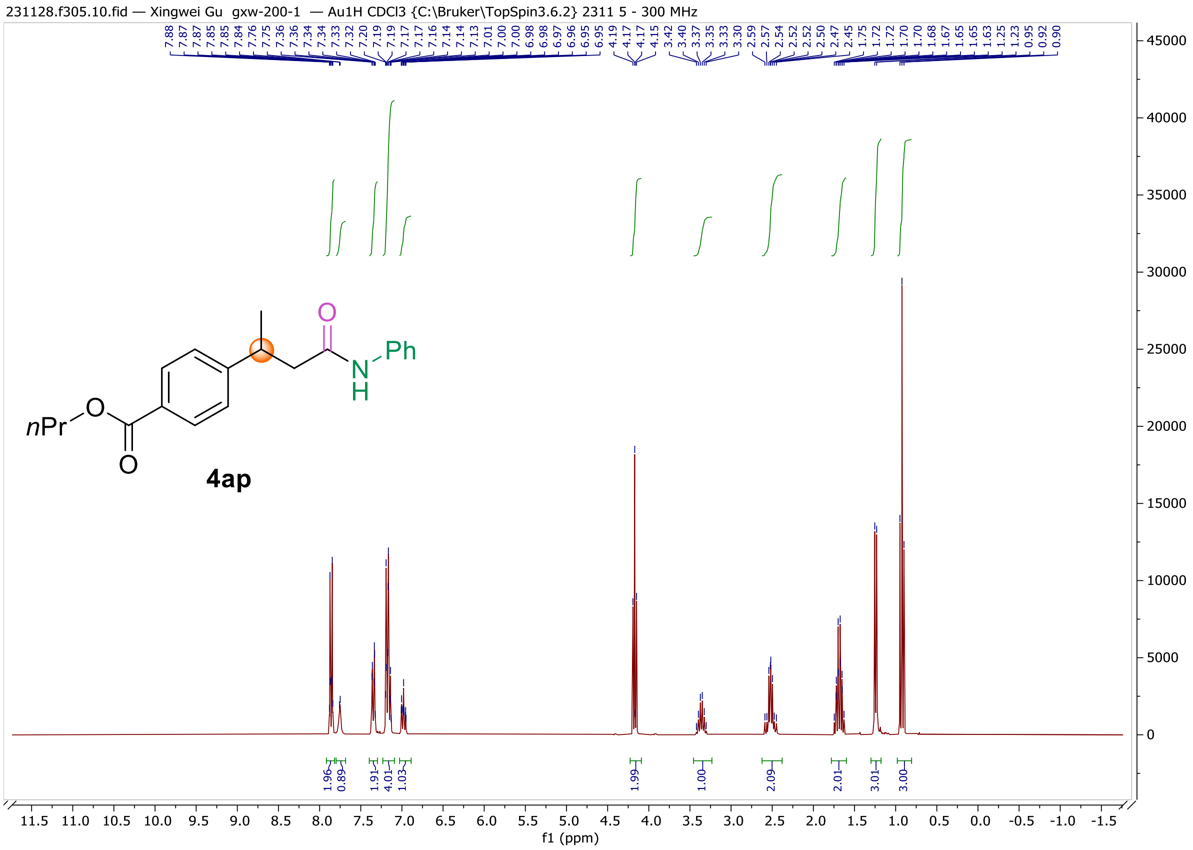
231128.f305.10.fid — Xingwei Gu gxw-200-1 — Au1H CDCl3 {C:\Bruker\TopSpin3.6.2} 2311 5 - 300 MHz
7.88 7.87 7.87 7.85 7.84 7.76 7.75 7.36 7.36 7.34 7.34 7.33 7.32 7.20 7.19 7.19 7.17 7.17 7.16 7.14 7.14 7.13 7.01 7.00 7.00 6.98 6.98 6.97 6.96 6.95 6.95 4.19 4.17 4.17 4.15 3.42 3.40 3.37 3.35 3.33 3.30 2.59 2.57 2.54 2.52 2.52 2.50 2.47 2.45 1.75 1.72 1.72 1.70 1.70 1.68 1.67 1.65 1.65 1.63 1.25 1.23 0.95 0.92 0.90
1.96 0.89 1.91 4.01 1.03 1.99 1.00 2.09 2.01 3.01 3.00
nPr
O
N
H
Ph
4ap
f1 (ppm)
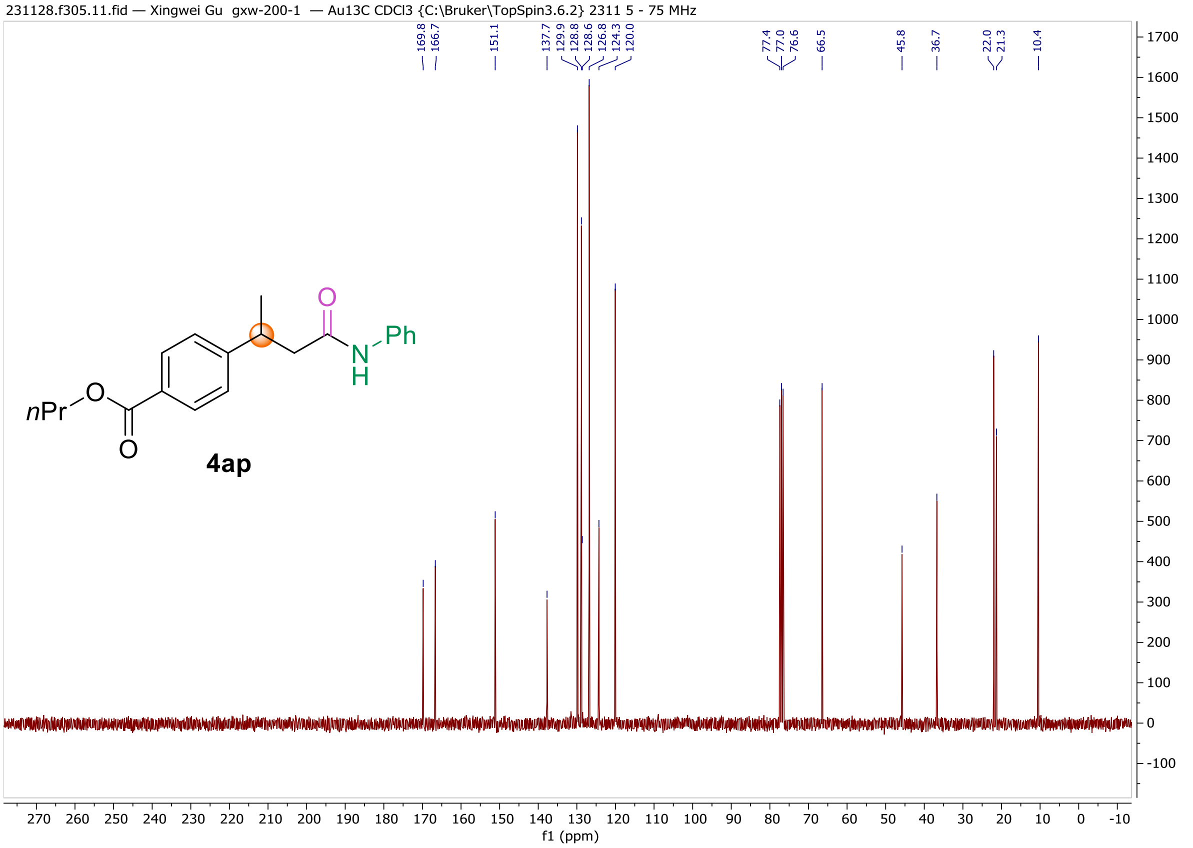
231128.f305.11.fid — Xingwei Gu gxw-200-1 — Au13C CDCl3 {C:\Bruker\TopSpin3.6.2} 2311 5 - 75 MHz
169.8 166.7 151.1 137.7 129.9 128.8 128.6 126.8 124.3 120.0 77.4 77.0 76.6 66.5 45.8 36.7 22.0 21.3 10.4
nPr
O
N
H
Ph
4ap
f1 (ppm)

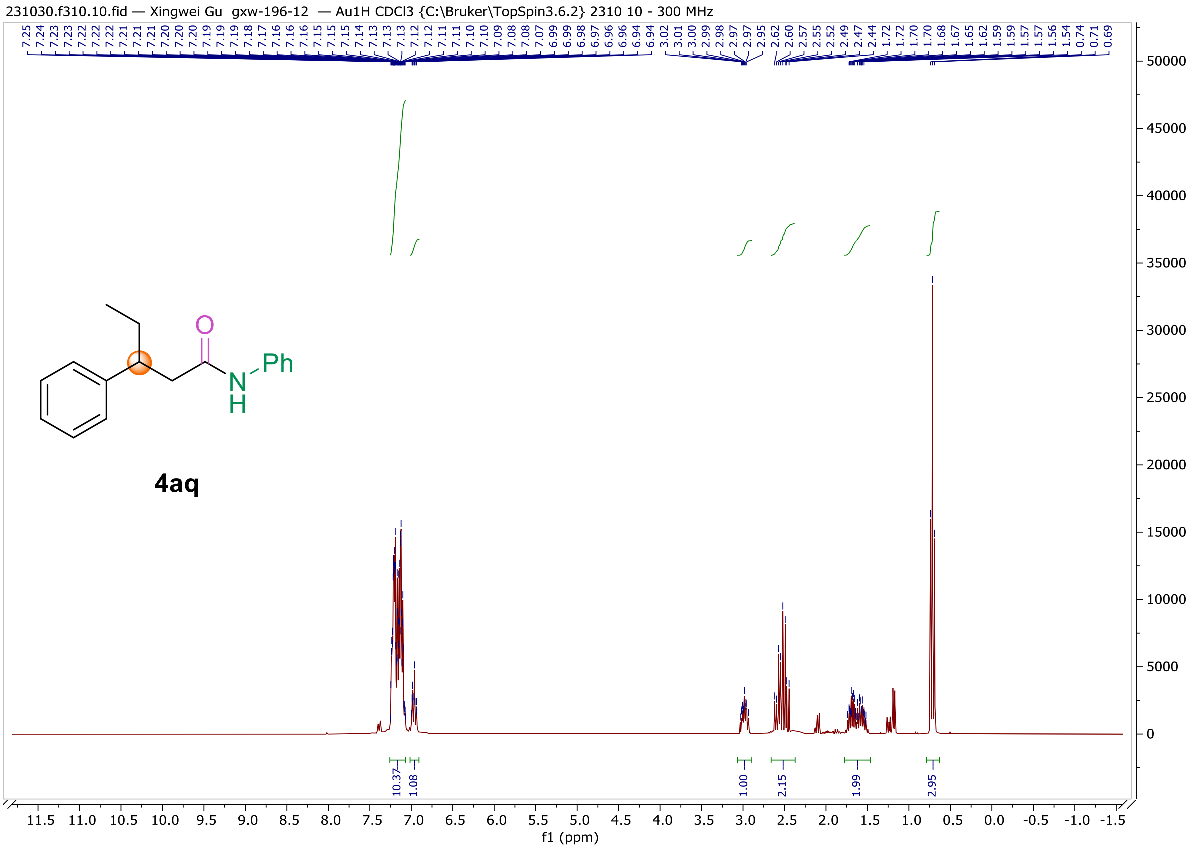
231030.f310.10.fid — Xingwei Gu gxw-196-12 — Au1H CDCl3 {C:\Bruker\TopSpin3.6.2} 2310 10 - 300 MHz
7.25 7.24 7.23 7.23 7.22 7.22 7.22 7.21 7.21 7.21 7.20 7.20 7.20 7.19 7.19 7.19 7.18 7.17 7.16 7.16 7.15 7.15 7.15 7.14 7.13 7.13 7.13 7.12 7.12 7.11 7.11 7.10 7.10 7.09 7.08 7.08 7.07 6.99 6.99 6.98 6.98 6.97 6.96 6.96 6.94 6.94 3.02 3.01 3.00 2.99 2.98 2.97 2.97 2.95 2.62 2.60 2.57 2.55 2.52 2.49 2.47 2.44 1.72 1.72 1.70 1.70 1.68 1.67 1.65 1.62 1.59 1.59 1.57 1.57 1.56 1.54 0.74 0.71 0.69
O
N
H
Ph
4aq
10.37 1.08 1.00 2.15 1.99 2.95
f1 (ppm)

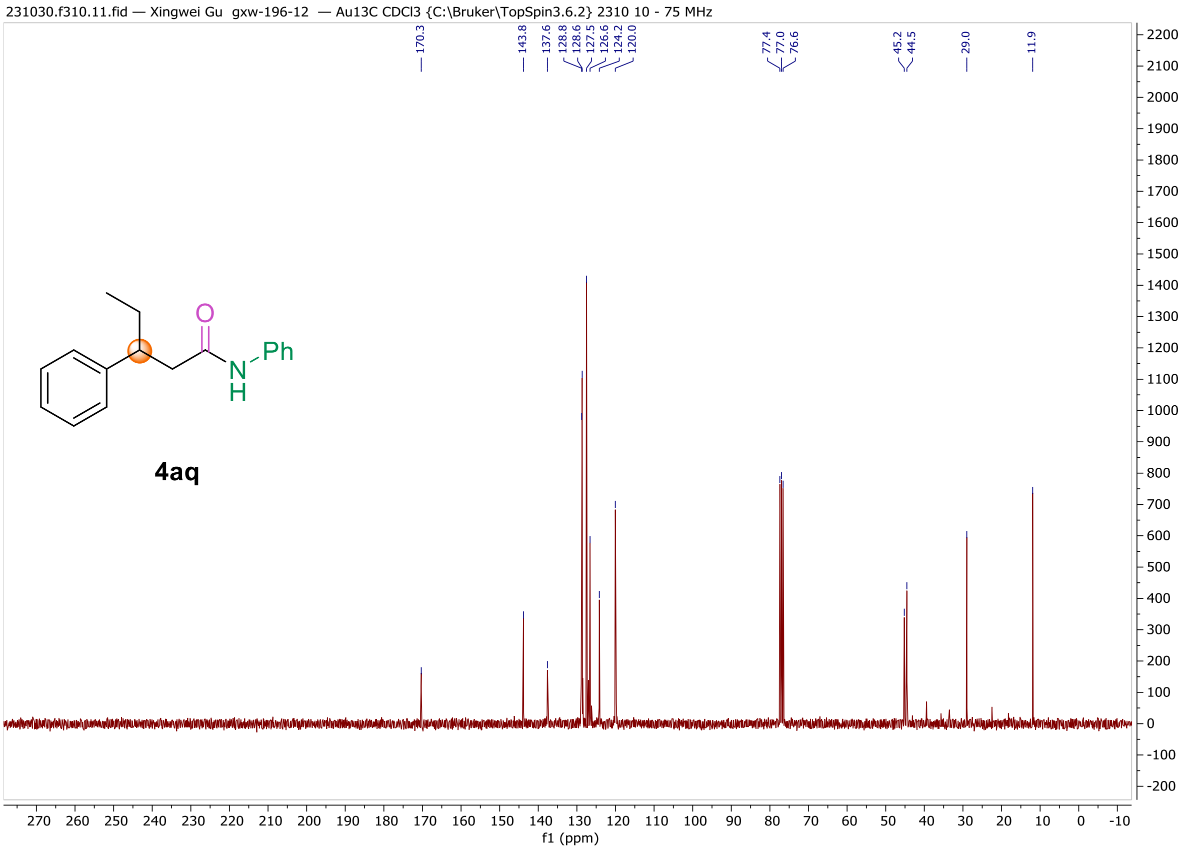
231030.f310.11.fid — Xingwei Gu gxw-196-12 — Au13C CDCl3 {C:\Bruker\TopSpin3.6.2} 2310 10 - 75 MHz
170.3 143.8 137.6 128.8 128.6 127.5 126.6 124.2 120.0 77.4 77.0 76.6 45.2 44.5 29.0 11.9
O
N
H
Ph
4aq
f1 (ppm)

231005.312.10.fid — Xingwei Gu gxw-188-2 — Au1H CDCl3 {C:\Bruker\TopSpin3.6.2} 2310 12 - 300 MHz

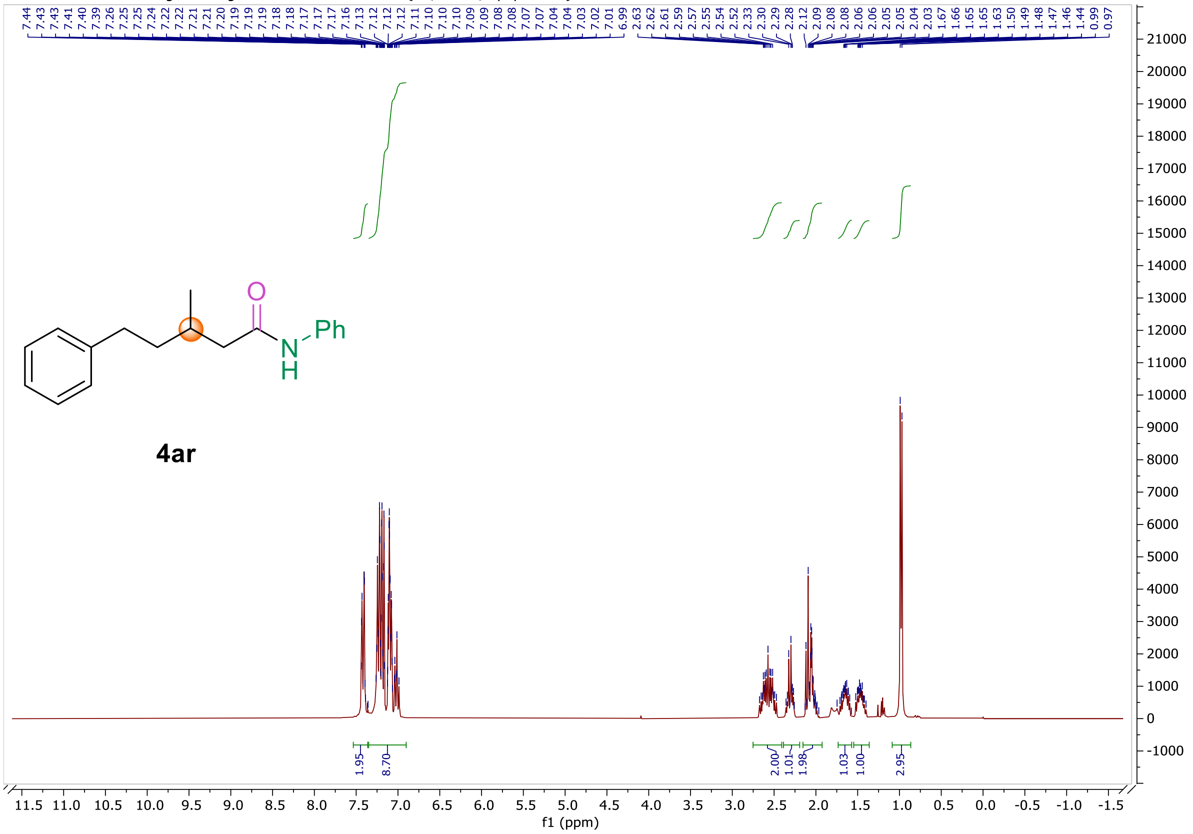


231001.304.11.fid — Xingwei Gu, gxw-188-2 — Au13C CDCl3 {C:\Bruker\TopSpin3.6.2} 2310 4 - 75 MHz

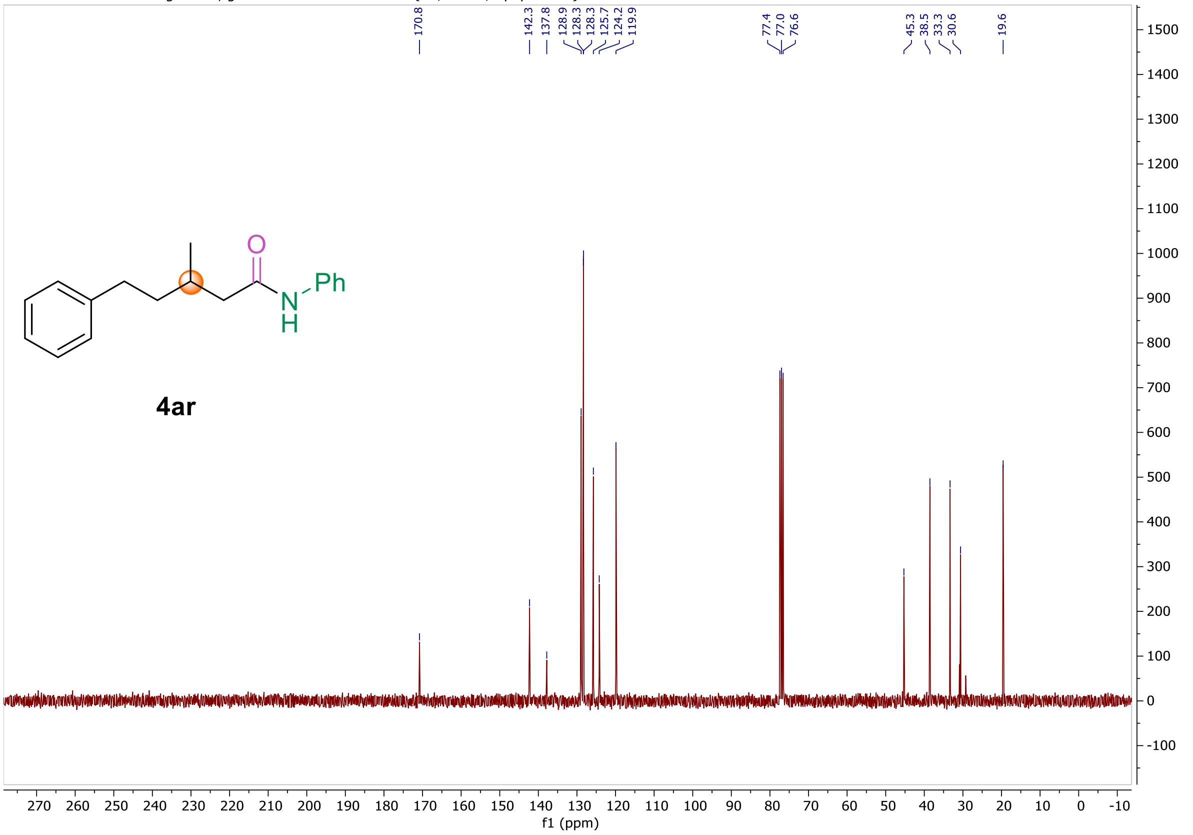

231005.309.10.fid — Xingwei Gu gxw-188-1 — Au1H CDCl3 {C:\Bruker\TopSpin3.6.2} 2310 9 - 300 MHz

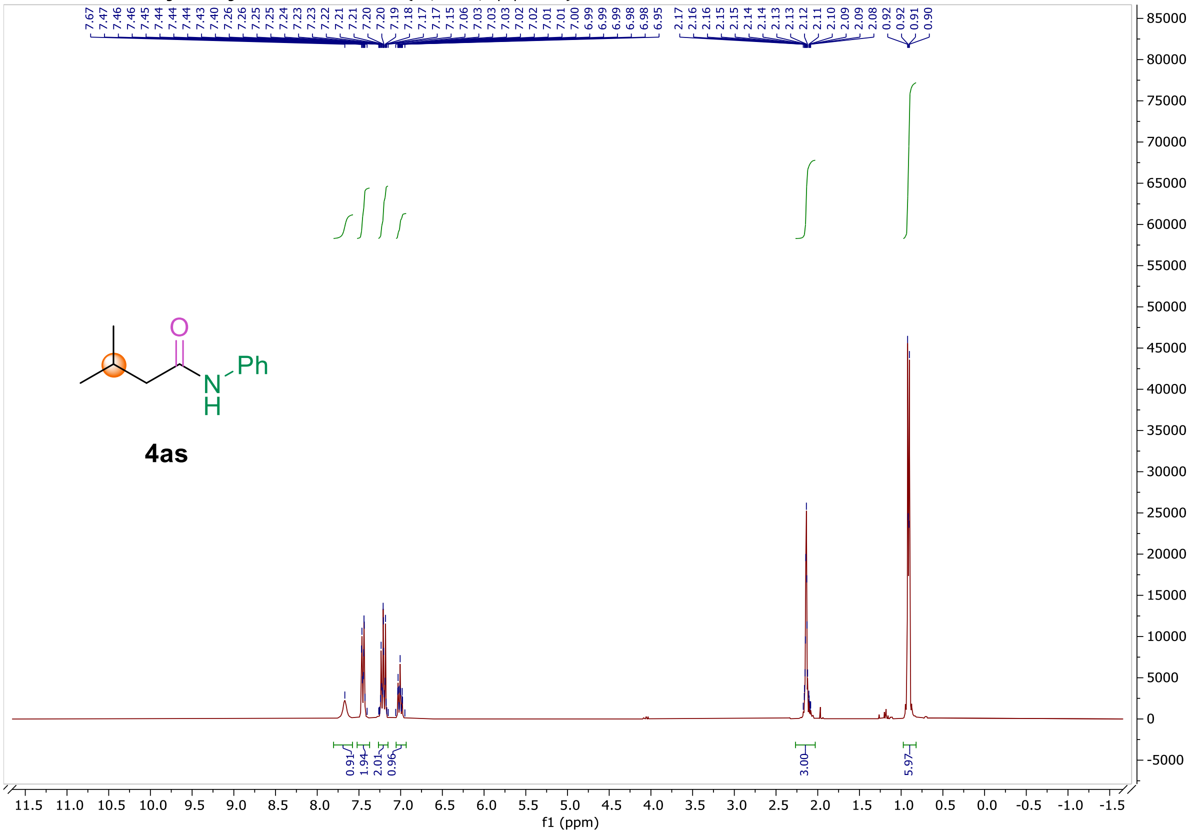


231005.309.11.fid — Xingwei Gu gxw-188-1 — Au13C CDCl3 {C:\Bruker\TopSpin3.6.2} 2310 9 - 75 MHz

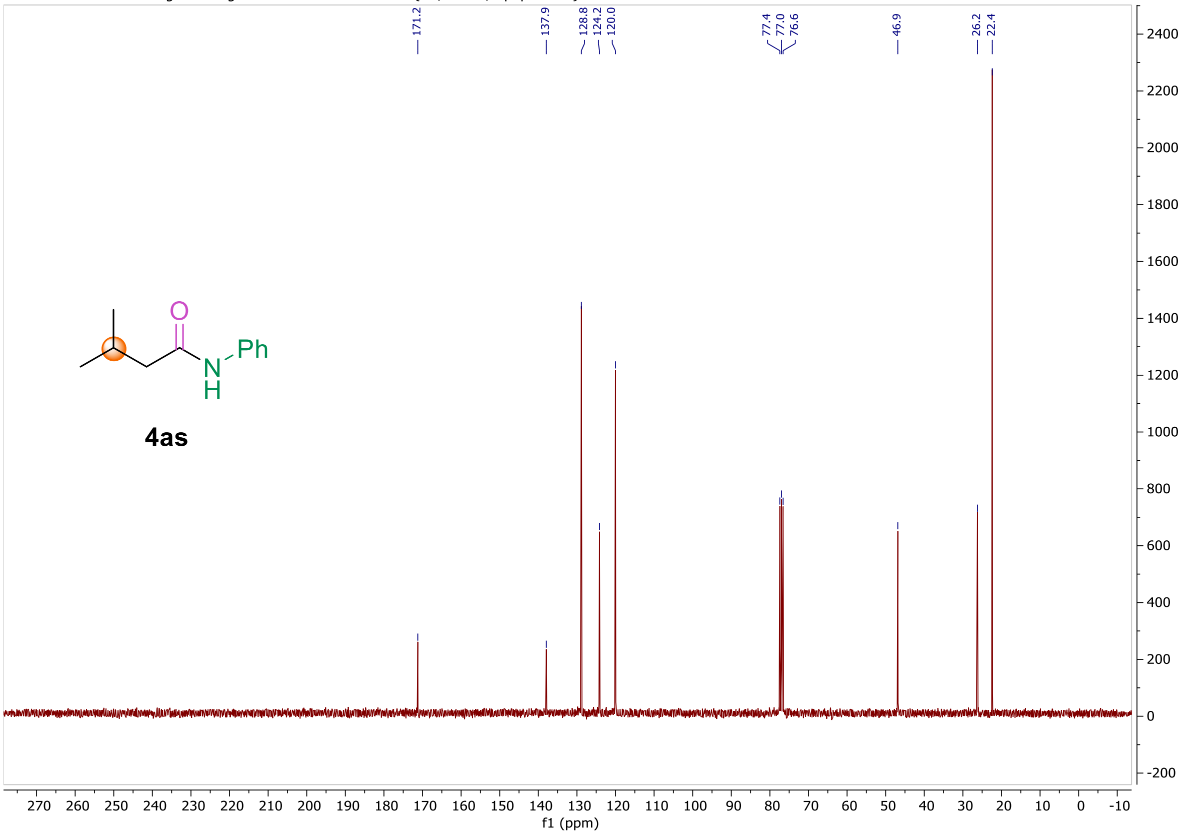

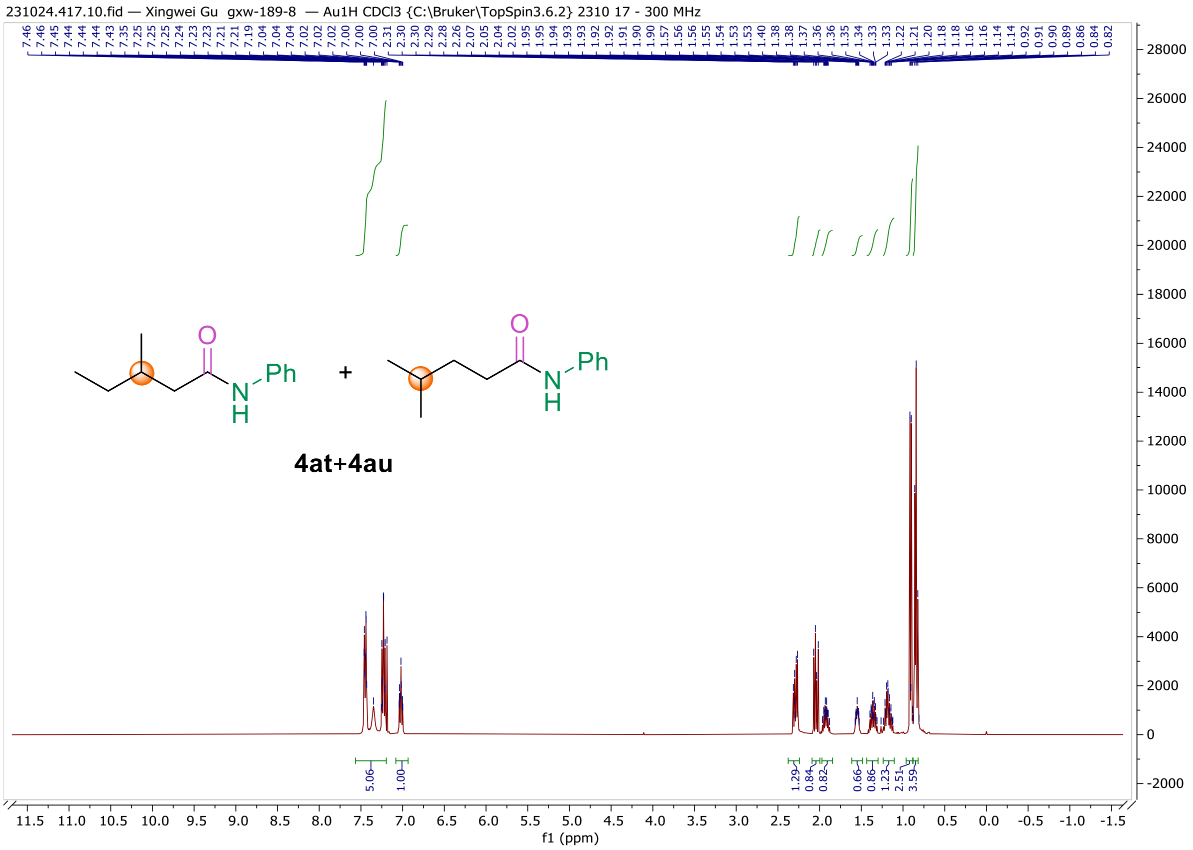
231024.417.10.fid — Xingwei Gu gxw-189-8 — Au1H CDCl3 {C:\Bruker\TopSpin3.6.2} 2310 17 - 300 MHz
7.46 7.46 7.45 7.44 7.44 7.44 7.43 7.35 7.25 7.25 7.25 7.24 7.23 7.23 7.23 7.21 7.21 7.19 7.04 7.04 7.04 7.02 7.02 7.02 7.00 7.00 7.00 2.31 2.30 2.30 2.29 2.28 2.26 2.07 2.05 2.04 2.02 1.95 1.95 1.94 1.93 1.93 1.92 1.92 1.91 1.91 1.90 1.90 1.57 1.56 1.56 1.55 1.54 1.53 1.53 1.40 1.38 1.38 1.37 1.36 1.36 1.35 1.34 1.33 1.33 1.22 1.21 1.20 1.18 1.16 1.16 1.14 1.14 0.92 0.91 0.90 0.89 0.86 0.84 0.82
O
N
H
Ph
+
4at+4au
5.06
1.00
1.29
0.84
0.82
0.66
0.86
1.23
2.51
3.59
f1 (ppm)

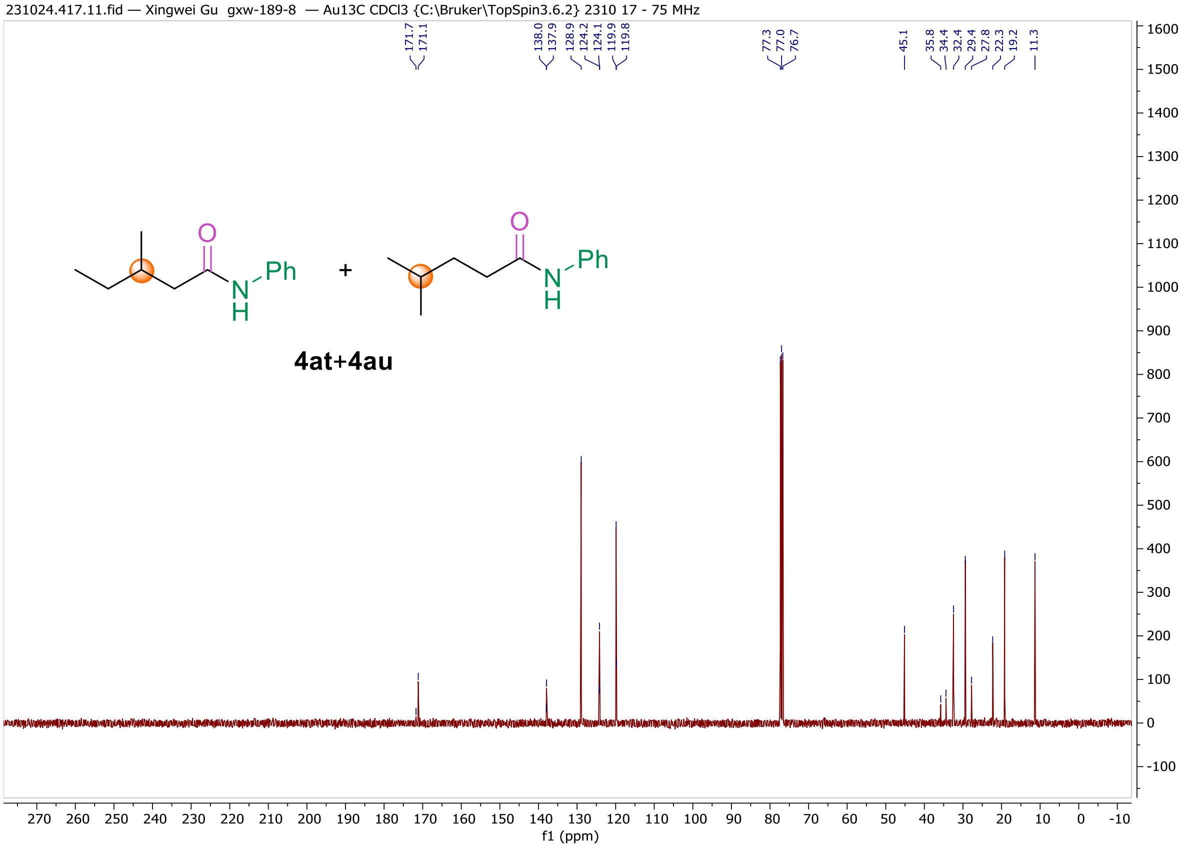
231024.417.11.fid — Xingwei Gu gxw-189-8 — Au13C CDCl3 {C:\Bruker\TopSpin3.6.2} 2310 17 - 75 MHz
171.7 171.1 138.0 137.9 128.9 124.2 124.1 119.9 119.8 77.3 77.0 76.7 45.1 35.8 34.4 32.4 29.4 27.8 22.3 19.2 11.3
O
N
H
Ph
+
4at+4au
f1 (ppm)

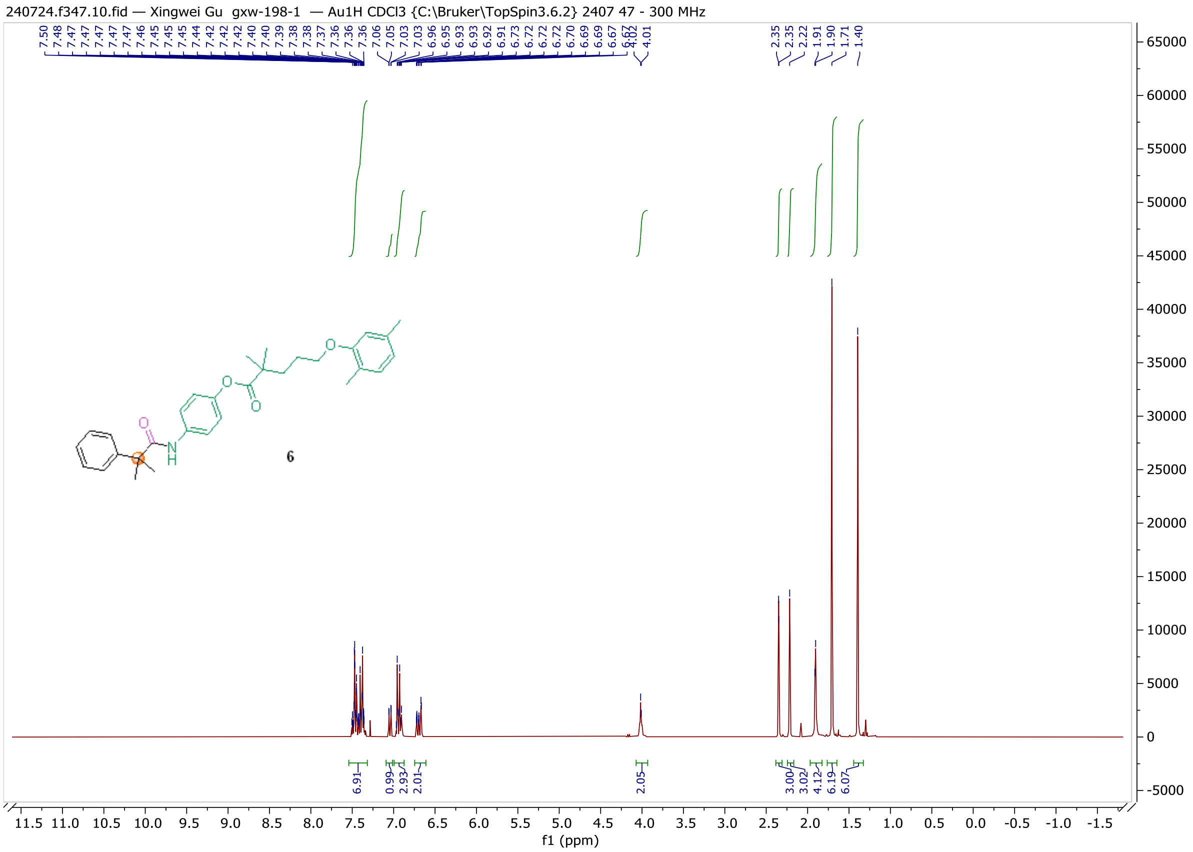
240724.f347.10.fid — Xingwei Gu gxw-198-1 — Au1H CDCl3 {C:\Bruker\TopSpin3.6.2} 2407 47 - 300 MHz
7.50 7.48 7.47 7.47 7.47 7.47 7.46 7.45 7.45 7.45 7.44 7.42 7.42 7.42 7.40 7.40 7.39 7.38 7.38 7.37 7.36 7.36 7.36 7.06 7.05 7.03 7.03 6.96 6.95 6.93 6.93 6.92 6.91 6.73 6.72 6.72 6.72 6.70 6.69 6.69 6.67 6.67 4.02 4.01
2.35 2.35 2.22 1.91 1.90 1.71 1.40
6
6.91 0.99 2.93 2.01 2.05 3.00 3.02 4.12 6.19 6.07
f1 (ppm)
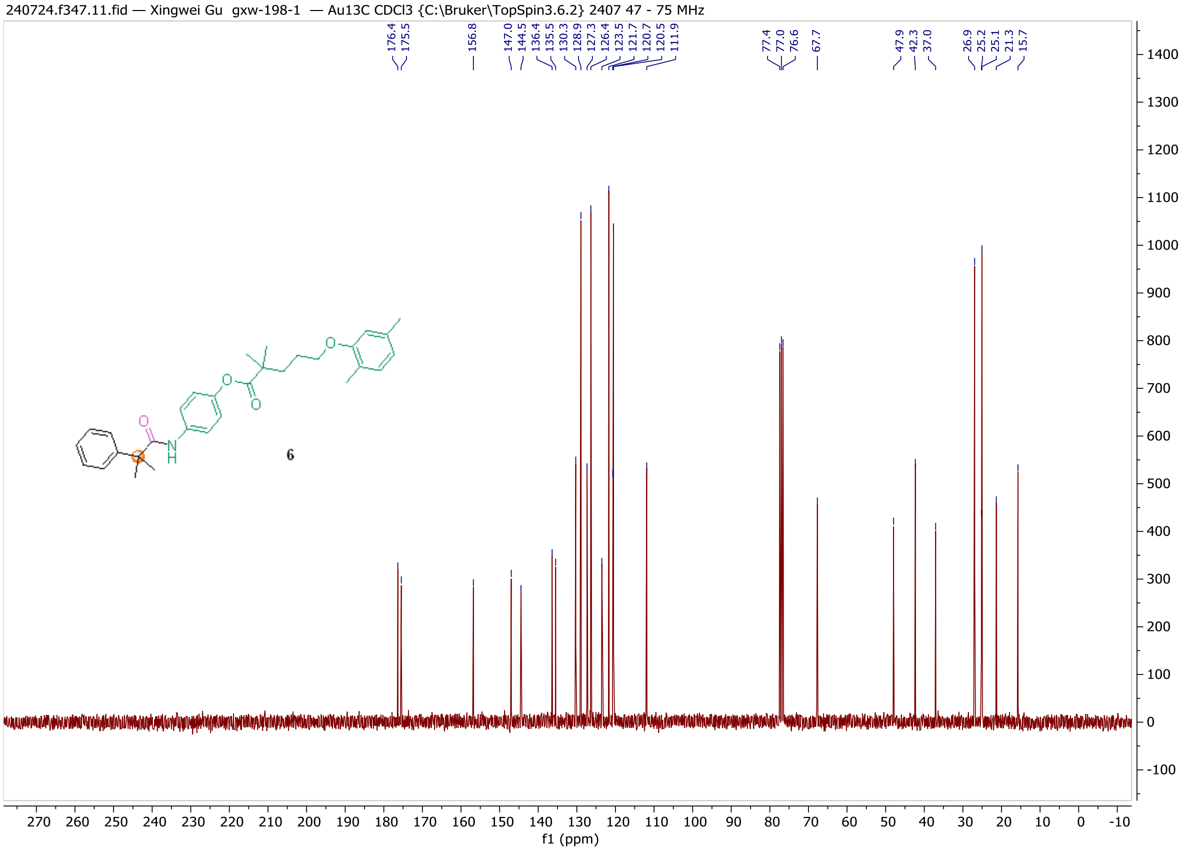
240724.f347.11.fid — Xingwei Gu gxw-198-1 — Au13C CDCl3 {C:\Bruker\TopSpin3.6.2} 2407 47 - 75 MHz
176.4 175.5 156.8 147.0 144.5 136.4 135.5 130.3 128.9 127.3 126.4 123.5 121.7 120.7 120.5 111.9
77.4 77.0 76.6 67.7 47.9 42.3 37.0 26.9 25.2 25.1 21.3 15.7
6
f1 (ppm)

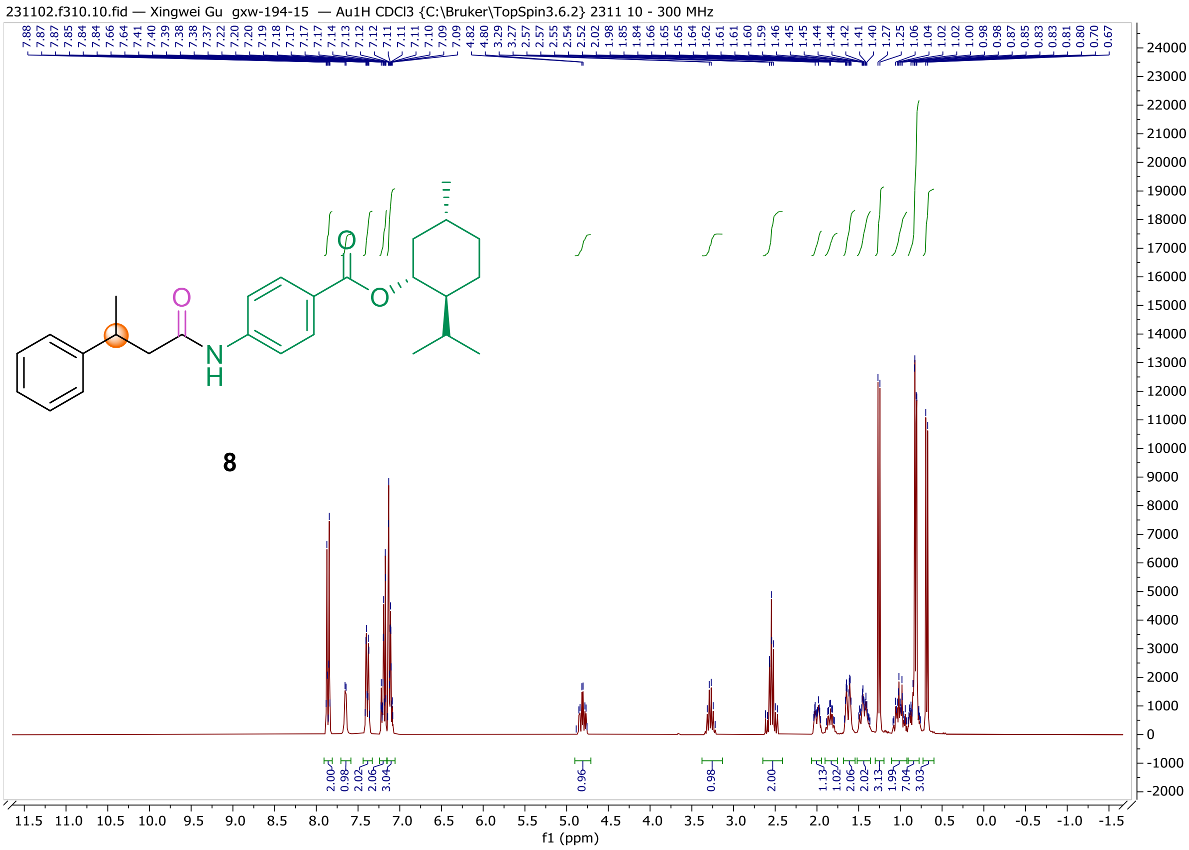
231102.f310.10.fid — Xingwei Gu gxw-194-15 — Au1H CDCl3 {C:\Bruker\TopSpin3.6.2} 2311 10 - 300 MHz
7.88 7.87 7.87 7.85 7.84 7.84 7.66 7.64 7.41 7.40 7.39 7.38 7.38 7.37 7.22 7.20 7.20 7.19 7.18 7.17 7.17 7.17 7.14 7.13 7.12 7.11 7.11 7.11 7.10 7.09 7.09 4.82 4.80 3.29 3.27 2.57 2.57 2.55 2.54 2.52 2.52 2.02 1.98 1.85 1.84 1.66 1.65 1.65 1.64 1.62 1.61 1.61 1.60 1.59 1.46 1.45 1.45 1.44 1.44 1.42 1.41 1.40 1.27 1.25 1.06 1.04 1.02 1.02 1.00 0.98 0.87 0.85 0.83 0.83 0.81 0.80 0.70 0.67
2.00 0.98 2.02 2.06 3.04 0.96 0.98 2.00 1.13 1.02 2.06 2.02 3.13 1.99 7.04 3.03
8
O
O
O
N
H
f1 (ppm)

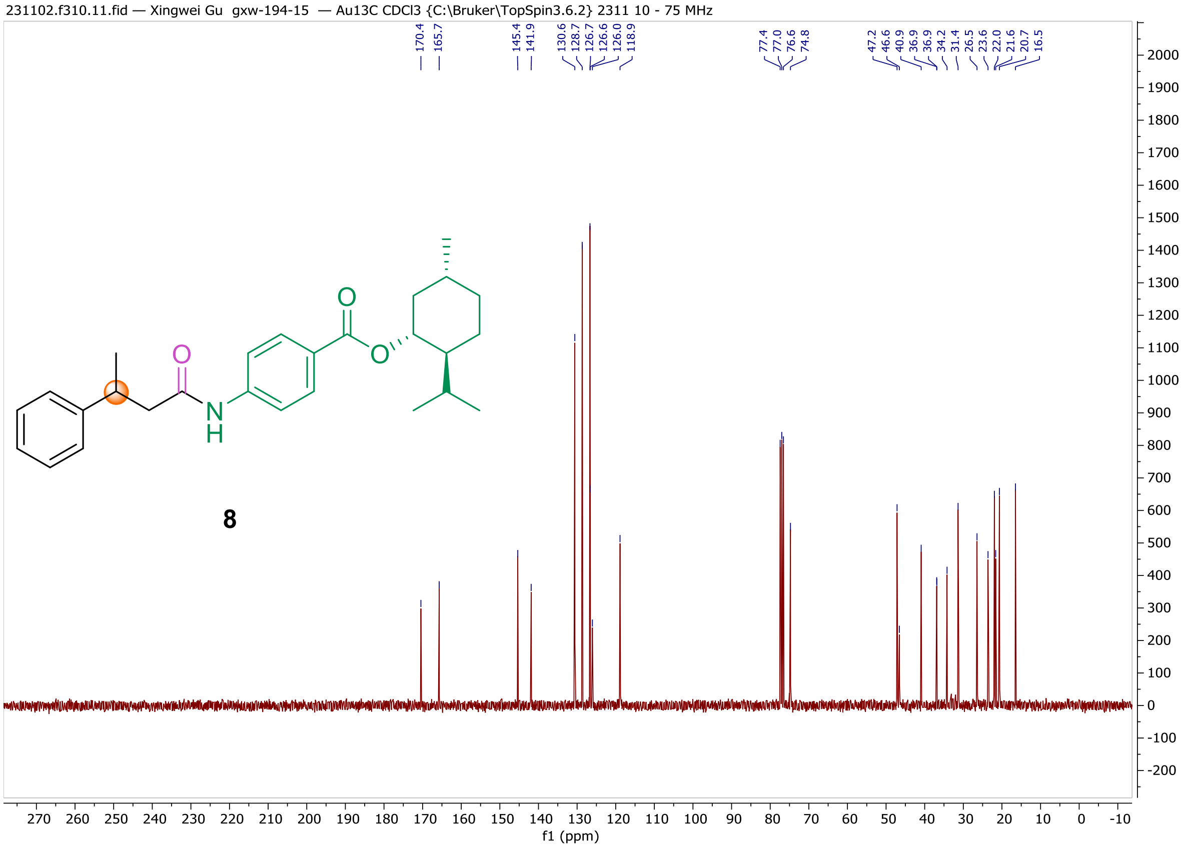
231102.f310.11.fid — Xingwei Gu gxw-194-15 — Au13C CDCl3 {C:\Bruker\TopSpin3.6.2} 2311 10 - 75 MHz
170.4 165.7 145.4 141.9 130.6 128.7 126.7 126.6 126.0 118.9 77.4 77.0 76.6 74.8 47.2 46.6 40.9 36.9 36.9 34.2 31.4 26.5 23.6 22.0 21.6 20.7 16.5
8
O
O
O
N
H
f1 (ppm)

240724.f348.10.fid — Xingwei Gu gxw-198-2 — Au1H CDCl3 {C:\Bruker\TopSpin3.6.2} 2407 48 - 300 MHz

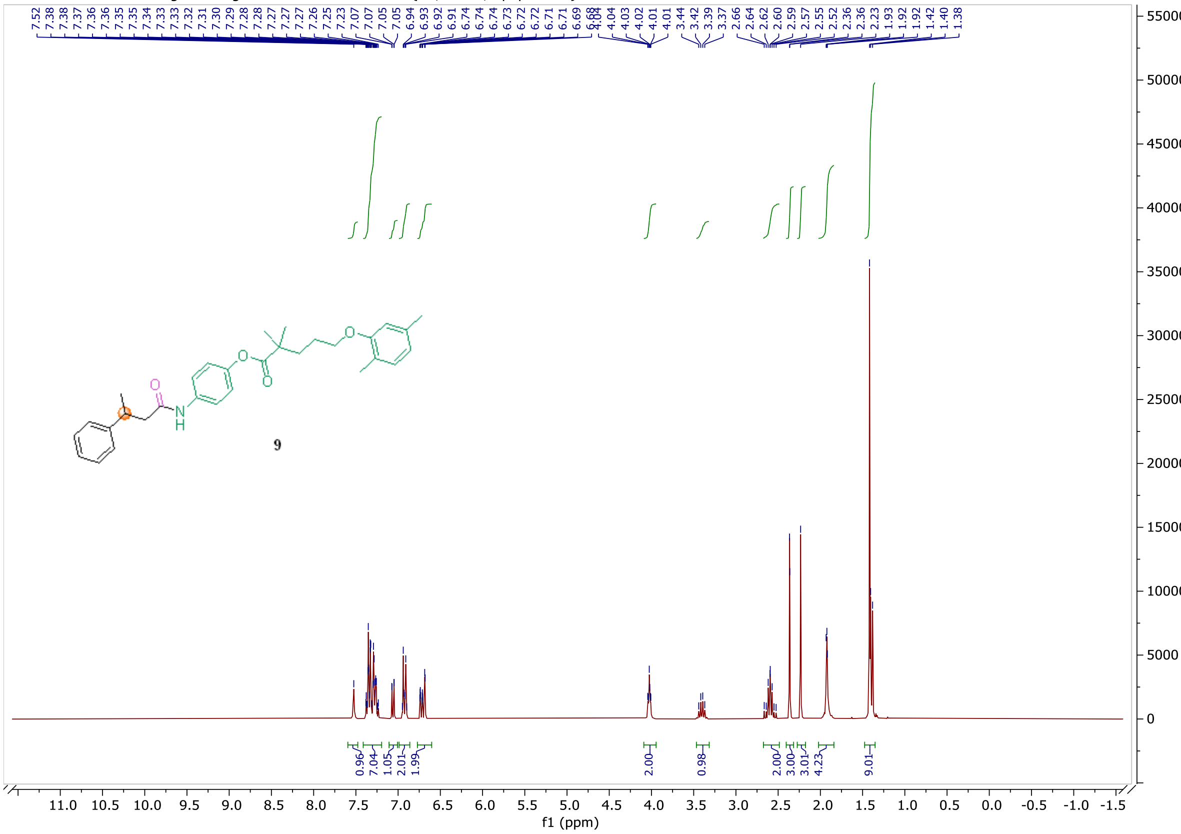

240724.f348.11.fid — Xingwei Gu gxw-198-2 — Au13C CDCl3 {C:\Bruker\TopSpin3.6.2} 2407 48 - 75 MHz

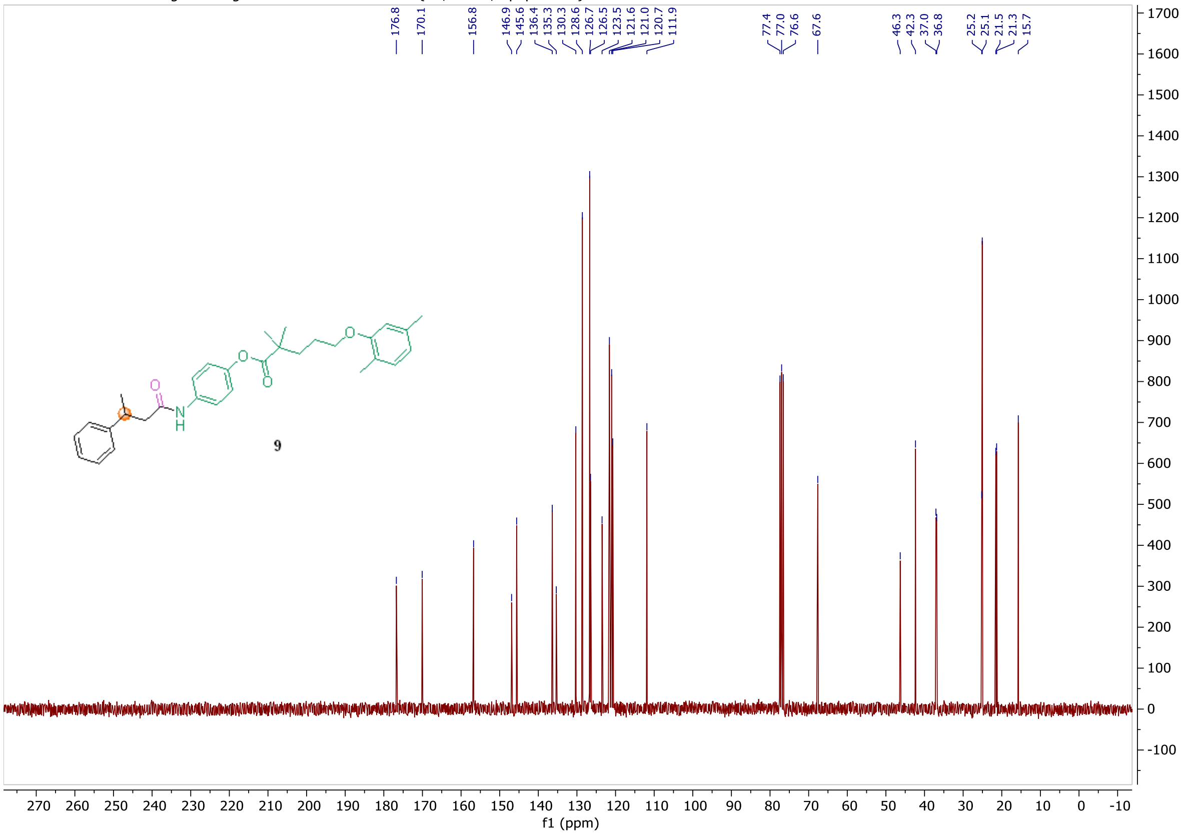

231108.f301.10.fid — Xingwei Gu gxw-194-20 — Au1H CDCl3 {C:\Bruker\TopSpin3.6.2} 2311 1 - 300 MHz

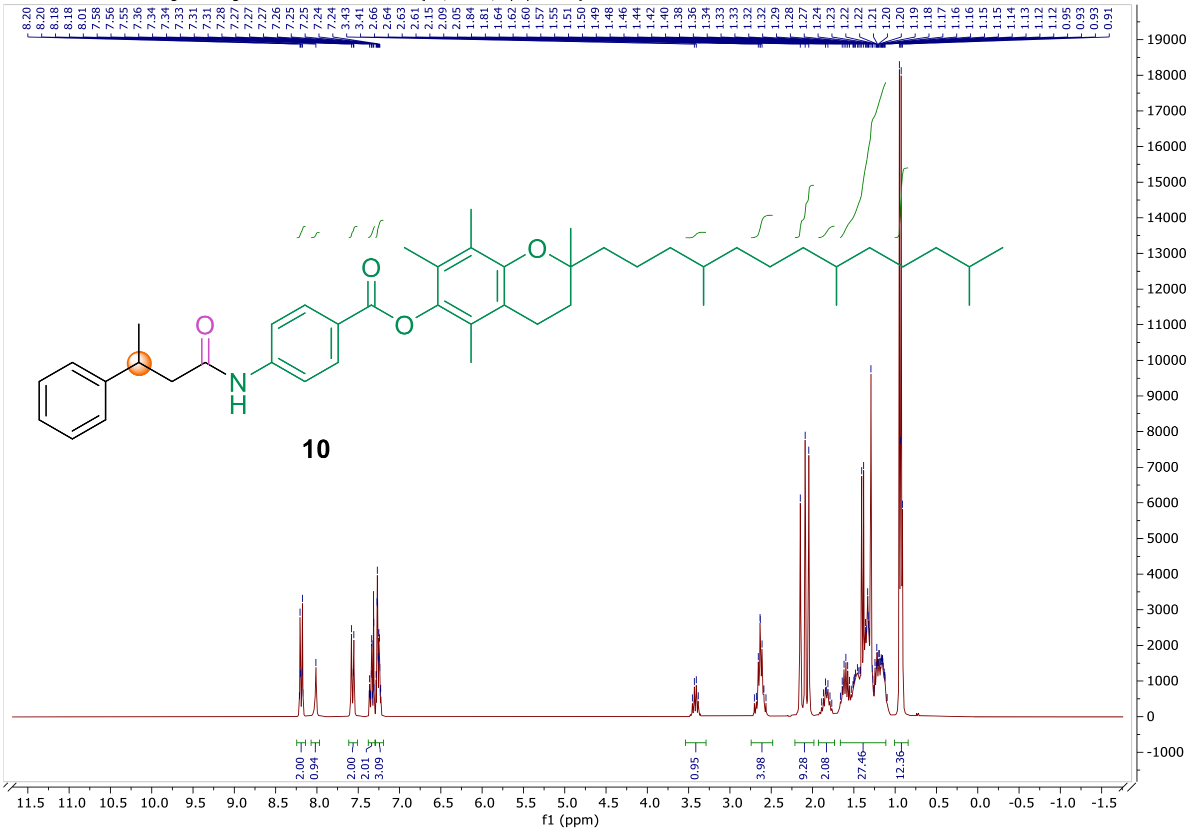


231108.f301.11.fid — Xingwei Gu gxw-194-20 — Au13C CDCl3 {C:\Bruker\TopSpin3.6.2} 2311 1 - 75 MHz

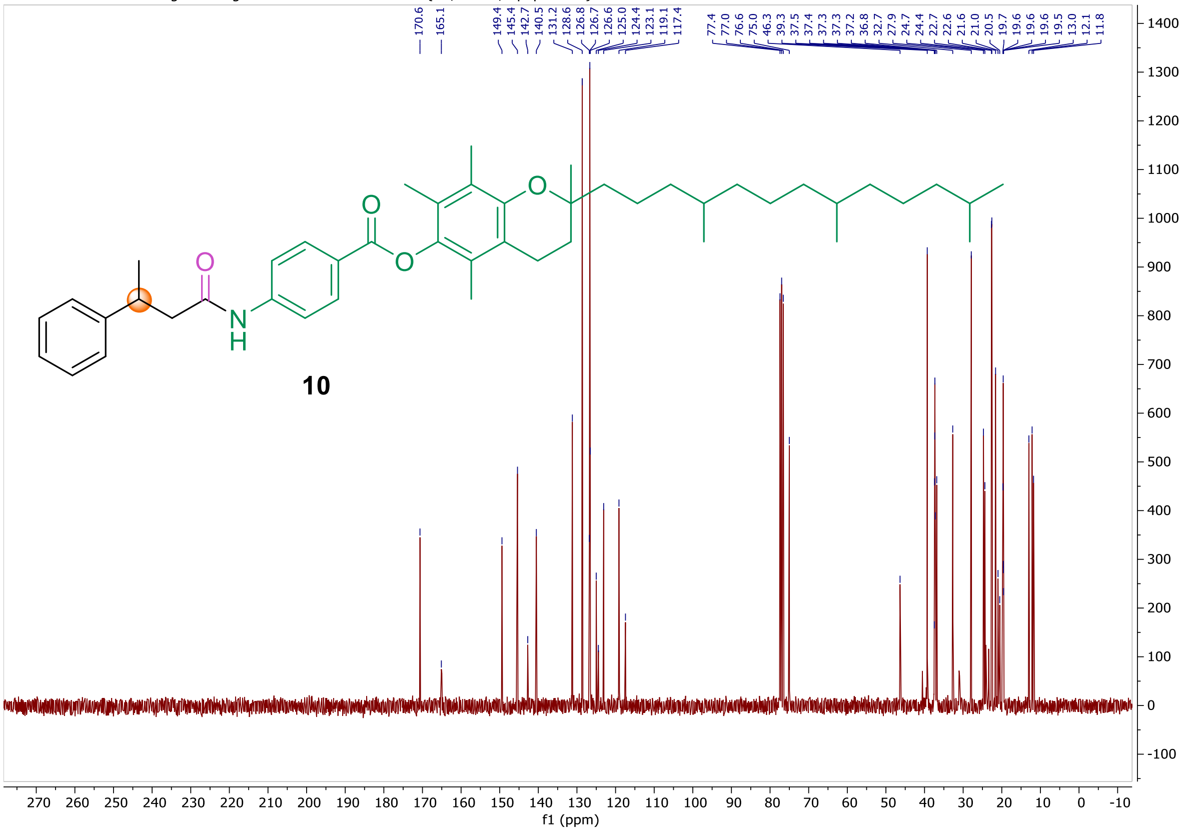

240724.f349.10.fid — Xingwei Gu gxw-198-3 — Au1H CDCl3 {C:\Bruker\TopSpin3.6.2} 2407 49 - 300 MHz

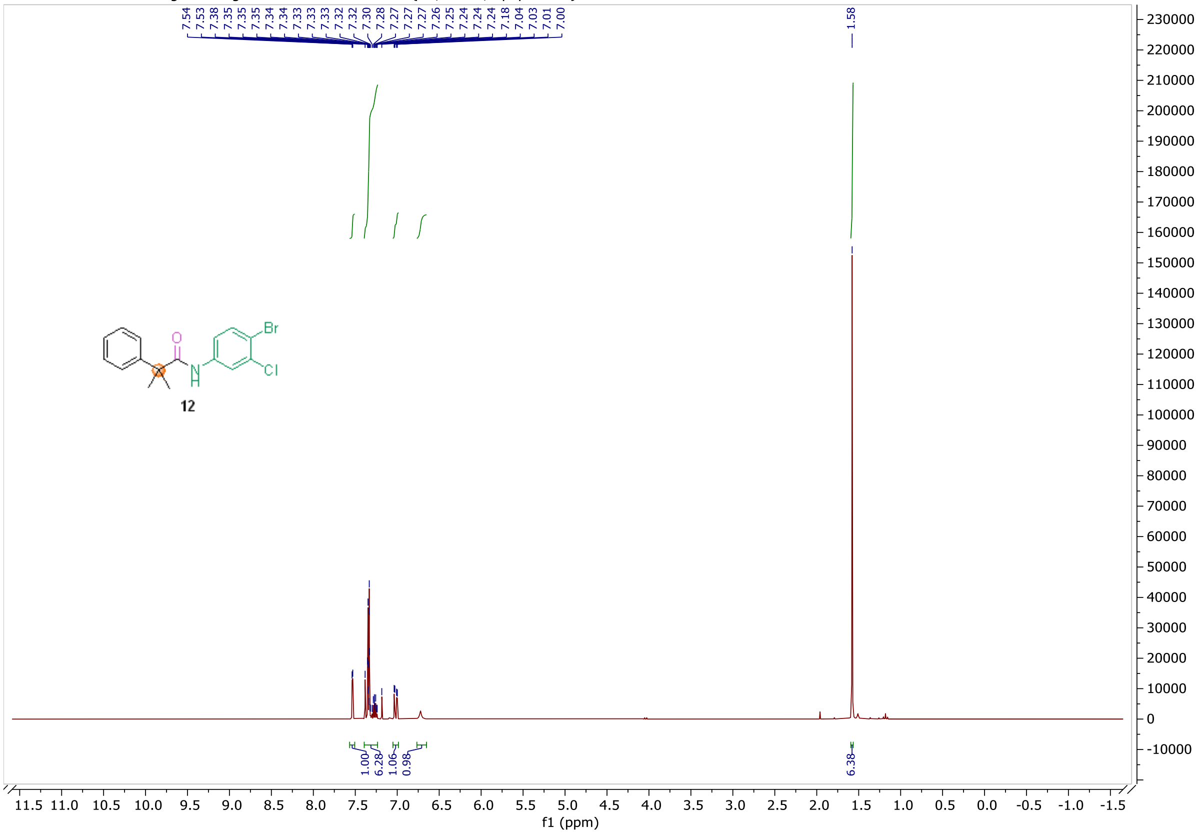


240724.f349.11.fid — Xingwei Gu gxw-198-3 — Au13C CDCl3 {C:\Bruker\TopSpin3.6.2} 2407 49 - 75 MHz

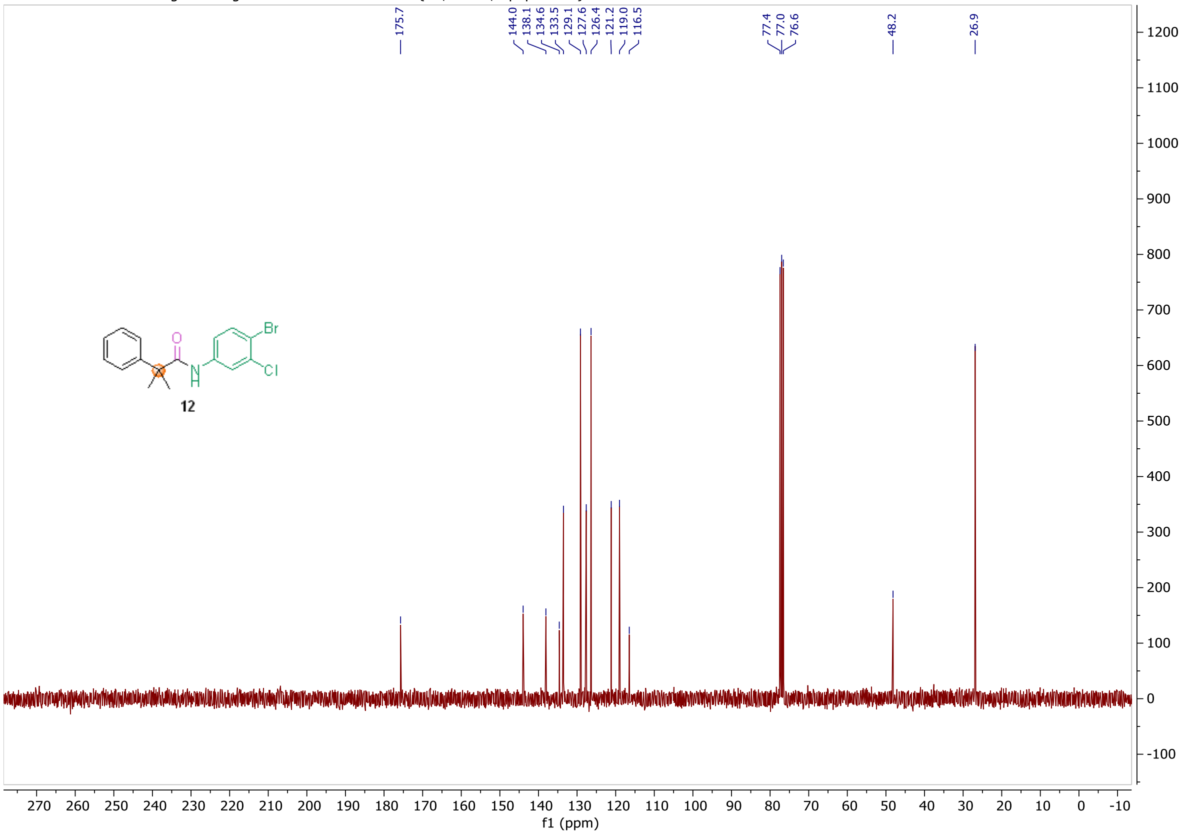

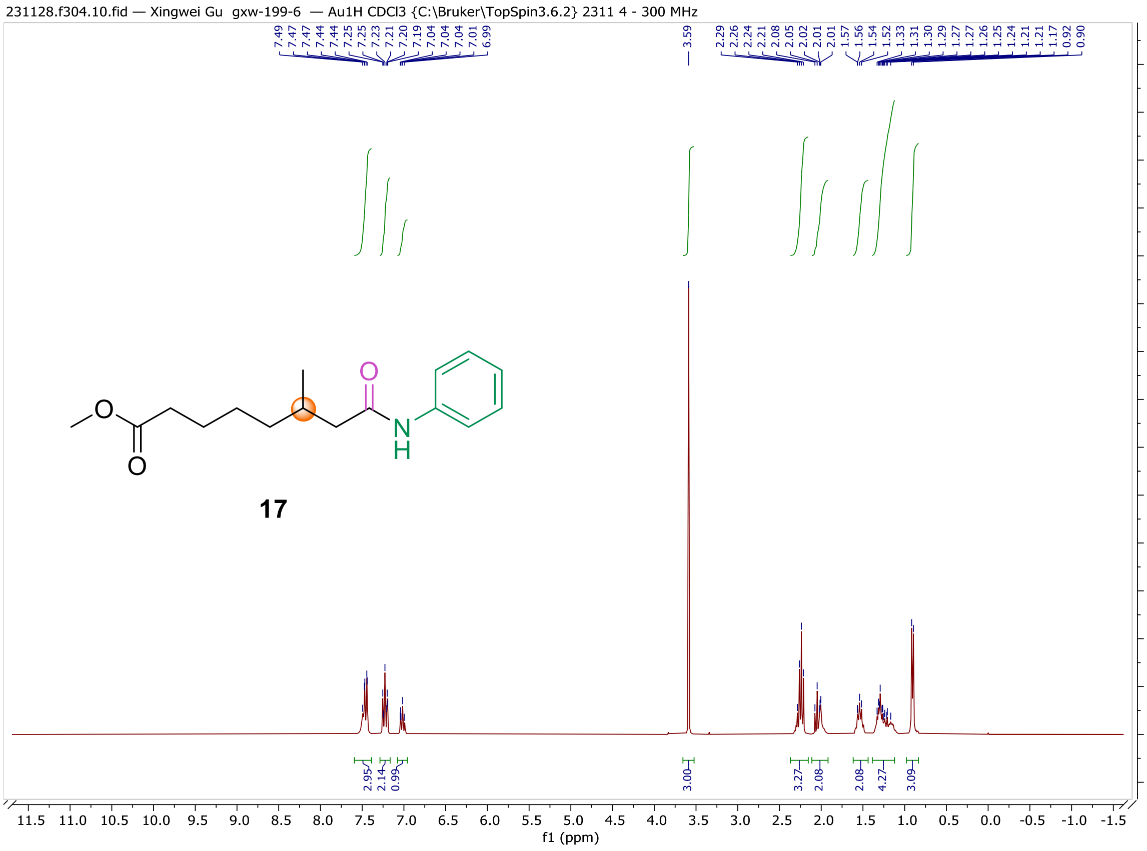
231128.f304.10.fid — Xingwei Gu gxw-199-6 — Au1H CDCl3 {C:\Bruker\TopSpin3.6.2} 2311 4 - 300 MHz
7.49 7.47 7.47 7.44 7.44 7.25 7.23 7.21 7.20 7.19 7.04 7.04 7.04 7.01 6.99
3.59
2.29 2.26 2.24 2.21 2.08 2.05 2.02 2.01 2.01 1.57 1.56 1.54 1.52 1.33 1.31 1.30 1.29 1.27 1.27 1.26 1.25 1.24 1.21 1.21 1.17 0.92 0.90
2.95 2.14 0.99 3.00 3.27 2.08 2.08 4.27 3.09
O
N
H
17
f1 (ppm)

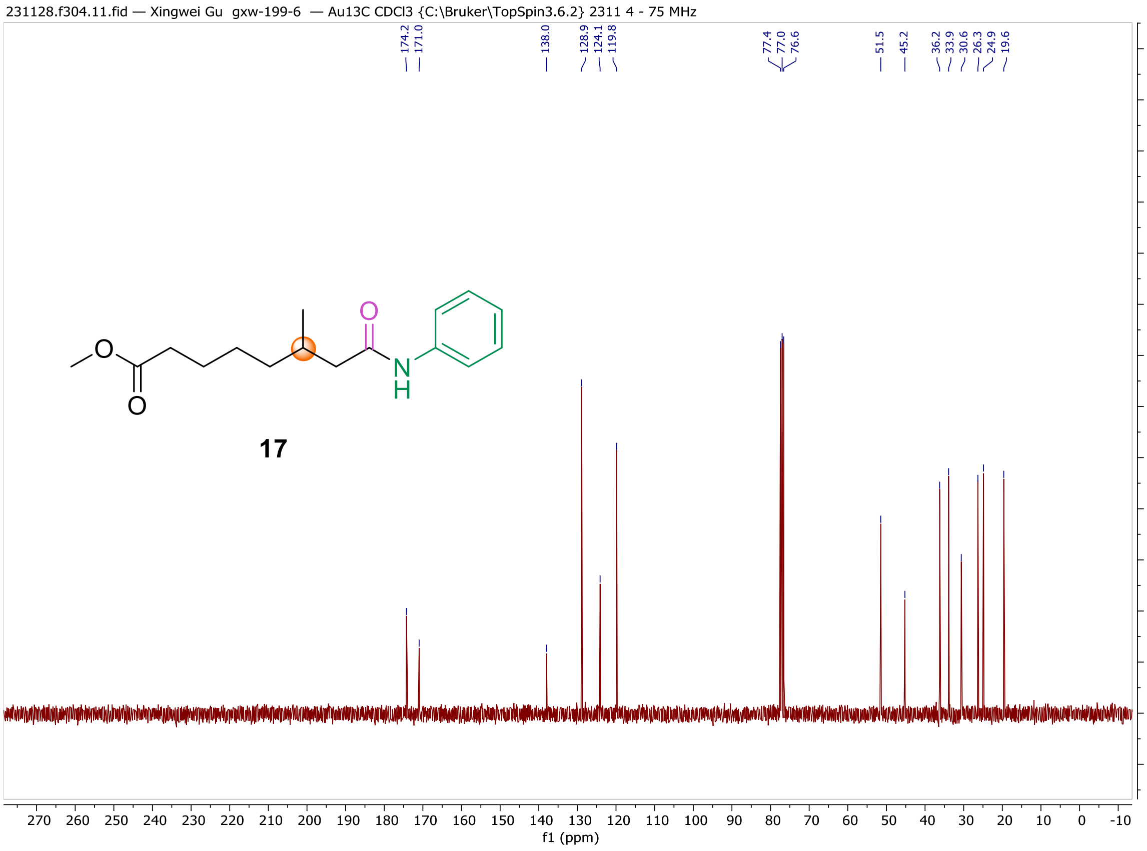
231128.f304.11.fid — Xingwei Gu gxw-199-6 — Au13C CDCl3 {C:\Bruker\TopSpin3.6.2} 2311 4 - 75 MHz
174.2 171.0 138.0 128.9 124.1 119.8 77.4 77.0 76.6 51.5 45.2 36.2 33.9 30.6 26.3 24.9 19.6
O
N
H
17
f1 (ppm)
